# 4D genetic networks reveal the genetic basis of metabolites and seed oil-related traits in 398 soybean RILs

**DOI:** 10.1186/s13068-022-02191-1

**Published:** 2022-09-09

**Authors:** Xu Han, Ya-Wen Zhang, Jin-Yang Liu, Jian-Fang Zuo, Ze-Chang Zhang, Liang Guo, Yuan-Ming Zhang

**Affiliations:** 1 College of Plant Science and Technology, Huazhong Agricultural University, Wuhan, 430070 China; 2 Institute of Industrial Crops, Jiangsu Academy of Agricultural Sciences, Nanjing, 210014 China

**Keywords:** Multi-dimension genetic network, Lipid, Metabolite, miRNA, Seed oil-related trait, Recombinant inbred line, Soybean

## Abstract

**Background:**

The yield and quality of soybean oil are determined by seed oil-related traits, and metabolites/lipids act as bridges between genes and traits. Although there are many studies on the mode of inheritance of metabolites or traits, studies on multi-dimensional genetic network (MDGN) are limited.

**Results:**

In this study, six seed oil-related traits, 59 metabolites, and 107 lipids in 398 recombinant inbred lines, along with their candidate genes and miRNAs, were used to construct an MDGN in soybean. Around 175 quantitative trait loci (QTLs), 36 QTL-by-environment interactions, and 302 metabolic QTL clusters, 70 and 181 candidate genes, including 46 and 70 known homologs, were previously reported to be associated with the traits and metabolites, respectively. Gene regulatory networks were constructed using co-expression, protein–protein interaction, and transcription factor binding site and miRNA target predictions between candidate genes and 26 key miRNAs. Using modern statistical methods, 463 metabolite–lipid, 62 trait–metabolite, and 89 trait–lipid associations were found to be significant. Integrating these associations into the above networks, an MDGN was constructed, and 128 sub-networks were extracted. Among these sub-networks, the gene–trait or gene–metabolite relationships in 38 sub-networks were in agreement with previous studies, e.g., oleic acid (trait)–*GmSEI*–*GmDGAT1a*–triacylglycerol (16:0/18:2/18:3), gene and metabolite in each of 64 sub-networks were predicted to be in the same pathway, e.g., oleic acid (trait)–*GmPHS*–d-glucose, and others were new, e.g., triacylglycerol (16:0/18:1/18:2)–*GmbZIP123*–*GmHD-ZIPIII-10*–miR166s–oil content.

**Conclusions:**

This study showed the advantages of MGDN in dissecting the genetic relationships between complex traits and metabolites. Using sub-networks in MGDN, 3D genetic sub-networks including pyruvate/threonine/citric acid revealed genetic relationships between carbohydrates, oil, and protein content, and 4D genetic sub-networks including *PLDs* revealed the relationships between oil-related traits and phospholipid metabolism likely influenced by the environment. This study will be helpful in soybean quality improvement and molecular biological research.

**Supplementary Information:**

The online version contains supplementary material available at 10.1186/s13068-022-02191-1.

## Background

Seed oil-related traits in soybean (*Glycine max*) are important traits, because soybean is the largest source of plant oil food and feed for millions of humans and live-stock [1]. Metabolites are essential to plants, affecting the diverse physiological and biochemical status of growth development in various environments [2]. It is widely known that metabolites act as bridges between genes and traits [3]. However, little is known about the genetic bases of trait-metabolite/lipid associations in soybean, especially with respect to miRNAs.

In recent decades, many associations of genes with oil-related traits in oil crops have been reported. Some associations have been used to elucidate the regulation of genes in carbon metabolism, such as oil content genes *GhPEPC1* in transgenic cotton [4], *GmSWEET10a* in transgenic soybean [5], and *AtPK* in transgenic *Arabidopsis* [6]. Meanwhile, lipids determine seed oil quality in dynamic metabolic pathways [7]. Thus, many associations of oil-related traits with genes in acyl-lipid pathways have been reported. Examples include stearic acid with *FAB2* [8], and oil content with *PDHC* [9] in fatty acid biosynthesis; fatty acids with *GmDGAT* [10], and oil content with *AtLPAAT* [11] and *GmPDAT* [12] in Kennedy pathways; fatty acids with *GmPLDα1* [13] and *AtPDCT* [14], oil content with *BnNPC6* [15], and fatty acids and oil content with *GmPLDγ* [16] in phospholipids pathways; oil content with *AtSEI1* [17] and *GmOLEO1* [18] in lipid droplet biogenesis; and fatty acids with *MDH2* [19] in fatty acid β-oxidation. In addition, some associations of oil-related traits with transcription factors (TFs) have been identified [20], e.g., *AtFUS3* [21], *GmbZIP123* [22], *BnLEC1a* [23], *GmDREBL* [24], *GmZF351* [25], and *GmDof11* [26]. However, these associations are limited as regards the genetic dissection of seed oil-related traits.

The genetic basis of metabolite–trait associations has attracted much attention in plant trait studies, e.g., phosphatidylinositol and phosphatidylinositol monophosphate with fiber growth in cotton [27], β-alanine with starch-related trait in potato tubers [28], and pyruvate and asparagine with oil content in soybean [29, 30]. Metabolic quantitative trait loci (mQTL) mapping and genome-wide association studies also aid research on metabolite–gene associations [31]. Up to now, many genetic bases of primary and secondary metabolites have been reported, such as histidine with *CAT4* in *Arabidopsis* [32], and apigenin di-C-hexoside with *GRMZM2G063550* in maize [33]. In addition, the database ARALIP (http://aralip.plantbiology.msu.edu/) in *Arabidopsis thaliana* provides many advantages for investigating lipid–gene associations [34]. Owing to a huge number of metabolites and intricate metabolic pathways, it is time and effort consuming to validate their candidate genes, a consideration that reduces the further use of the metabolite data to dissect the genetic foundation of these oil-related traits and improve them in soybean breeding.

miRNAs control plant development and regulate important traits through post-transcriptional gene regulation [35]. There are many experimental biology studies on the associations of miRNA with genes and their regulation mechanisms, e.g., *GmNINa*–miR172c–NNC1, and miR167–*GmARF8* in soybean nodulation [36, 37], miR828–*GhMYB2* and miR858–*GhMYB2* in cotton fiber trait [38], and OsmiR397–LAC in rice grain yield trait [39]. In lipid studies, high-throughput sequencing was used to identify the miRNAs related to both lipid metabolism and oil-related traits in *Brassica napus* [40], *Hippophae rhamnoides* [41], and *Camellia oleifera* [42]. Zhang et al. [43] predicted that bna-miR169 determined the oil content difference between *Glycine max* and *Brassica napus*, while bna-miR156, along with SPL, affected seed oil content by influencing early embryo development [40, 44]. In *Camelina sativa*, miR167a–*CsARF8* mediates LAFL regulation network for *CsFAD3* suppression and decreases seed linolenic acid content [45]. Thus, there exists great potential to dissect the genetic basis of oil-related traits through miRNA regulation.

**Fig. 1:**
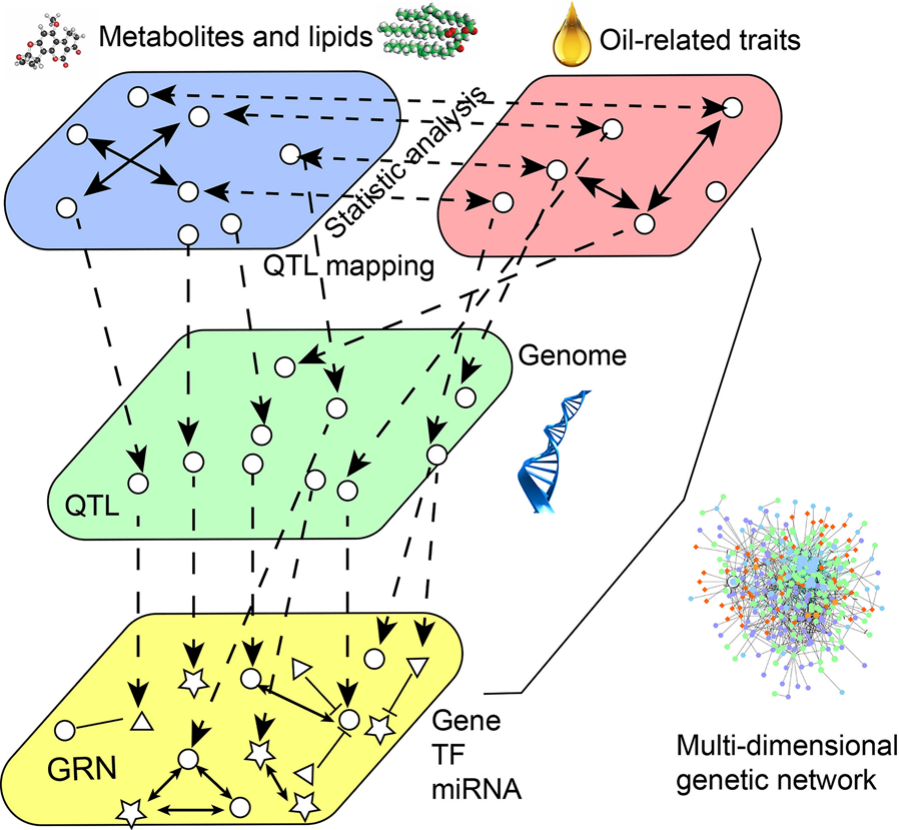
Overview of soybean multi-dimensional genetic network among genes, TFs, miRNAs, metabolites, lipids, and oil-related traits. This network includes three layers, namely, oil-related traits and metabolites/lipids layer, genome layer, and gene regulatory network layer

In this study, one MDGN was constructed using the associations among genes, TFs, miRNAs, metabolites/lipids, and seed oil-related traits (Fig. 1). In the oil-related trait and metabolite/lipid layer, the associations of seed oil-related traits with metabolites/lipids were obtained by modern statistical methods. In the genome layer, the associations of genes with seed oil-related traits or metabolites were obtained by quantitative trait locus (QTL) mapping. In the gene regulatory network (GRN) layer, the GRN among genes, TFs, and miRNAs was constructed using co-expression, protein–protein interaction (PPI), and TF binding site (TFBS) and miRNA target predictions. The first two associations were integrated into the GRN to construct the MDGN. Among the networks, hub nodes were mined. Thus, some important sub-networks containing hub nodes related to oil biosynthesis were identified. These findings will be useful for soybean oil quality improvement and identification of lipid metabolism regulation.

## Results

### Distribution of six seed oil-related traits, 59 metabolites, and 107 lipids in 398 soybean RILs

**Fig. 2:**
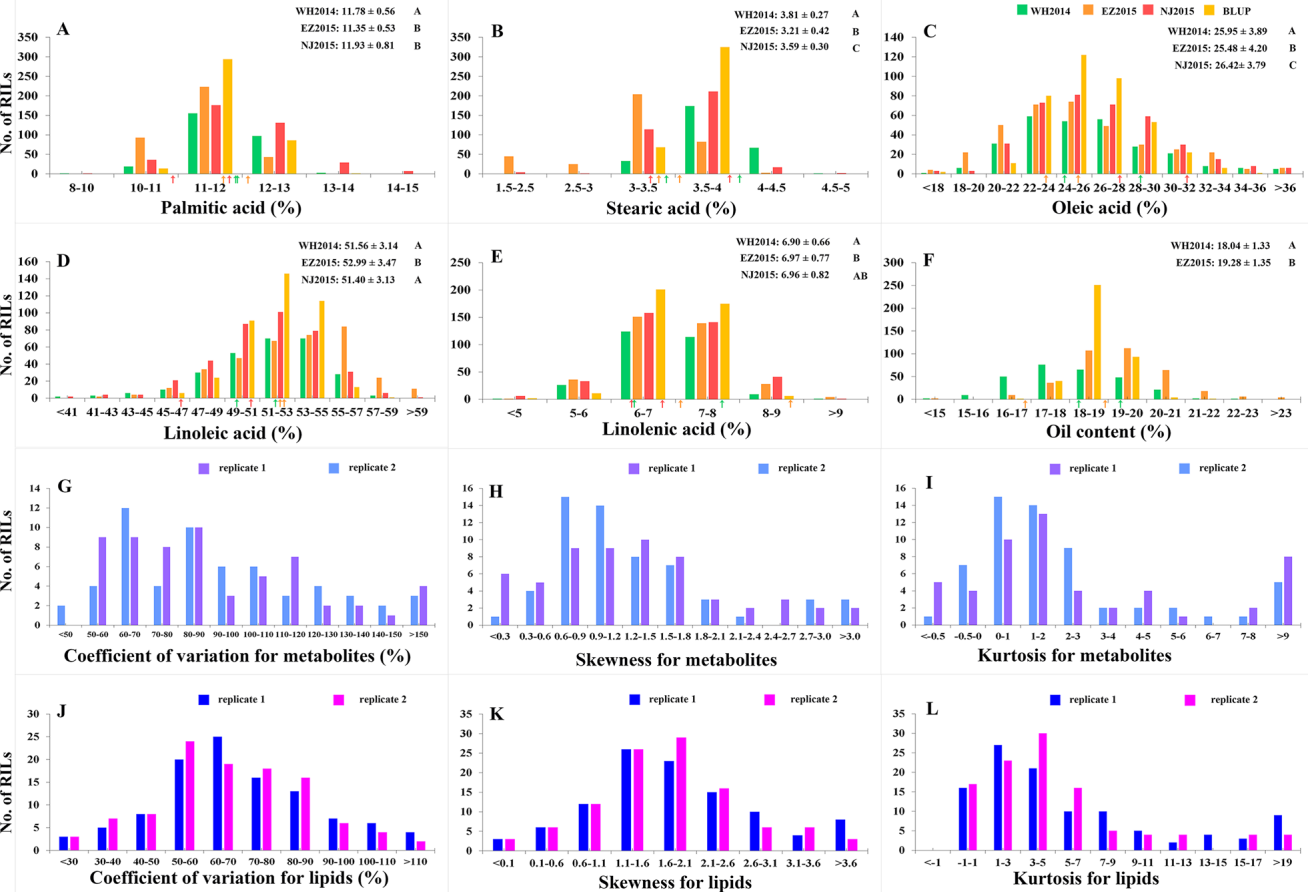
Frequency distributions for seed oil-related traits and variation characteristics of metabolites/lipids in 398 soybean RILs. **A**–**E** Seed fatty acid constituents. **F** Seed oil content. **G**, **J** Coefficients of variation. **H**, **K** Skewness. **I**, **L** Kurtosis. WH2014: Wuhan in 2014 (green); EZ2015: Ezhou in 2015 (orange); NJ2015: Nanjing in 2015 (red); BLUP: best linear unbiased prediction (yellow). The mean phenotypes of two parents for oil-related traits in each environment are indicated by arrows with different colors. LSD was used to test the significance of differences between various environments, and the significance was marked by different characters. All the data are indicated by mean ± standard deviation

In 398 recombinant inbred lines (RILs) of soybean, five seed oil constituents, including palmitic acid, stearic acid, oleic acid, linoleic acid, and linolenic acid, were measured in three environments (WH2014, EZ2015, and NJ2015; Fig. 2A–E), and seed oil content was measured in two environments (WH2014 and EZ2015; Fig. 2F). Frequency distributions of six seed oil-related traits in 398 RILs showed that they were typical quantitative traits with large variation, indicating the existence of large-effect genes for most traits other than stearic and linoleic acid content (Table 1; Additional file 1: Table S1; Fig. 2A–F).

**Table 1:** Overview of phenotypic characteristics and the numbers of QTLs/mQTLs for oil-related traits, metabolites, and lipids

Traits	No. of species	Phenotypic characteristics			Quantitative trait locus mapping		
		Coefficients of variation (%)	Skewness	Kurtosis	No. of QTL/mQTL	No. of candidate genes	No. of candidate miRNAs
Seed oil-related traits							
Stearic acid	1	4.06	0.1777	0.2377	32	16	0
Palmitic acid	1	7.15	1.9696	30.8676	22	5	0
Oleic acid	1	11.48	0.3230	1.8591	22	9	2
Linoleic acid	1	4.69	− 0.4509	0.8151	40	16	4
Linolenic acid	1	8.13	− 0.6409	4.3260	38	14	7
Oil content	1	4.79	0.4022	2.1282	21	8	4
Metabolites							
Carbohydrates	8	78.34 ± 25.26	1.9570	7.4024	11	9	3
Lipids	17	106. 03 ± 43.87	1.3740	4.2657	27	20	4
Organic acids	19	91.40 ± 34.23	1.9186	6.3696	31	26	6
Amino acids	15	90.89 ± 33.05	1.2199	2.1678	27	24	2
Lipids							
Fatty acids	10	121.01 ± 17.98	2.5373	8.1187	43	29	7
Glycerolipids	50	68.19 ± 26.30	2.0662	13.0418	123	83	7
Glycerolphospholipids	44	80.11 ± 20.09	1.7128	5.5052	64	39	6
Sphingolipids	3	60.11 ± 3.39	1.5809	3.2449	3	3	0

Fifty-five primary and four secondary metabolites were measured with two biological replicates in NJ2016 using the GC–TOF–MS method, and were classified into 19 organic acids, 15 amino acids, 17 lipids (2 sphingolipids and 15 fatty acids), and 8 carbohydrates (see Additional file 1: Table S2 for detail). Frequency distributions of coefficients of variation (CV), skewness, and kurtosis for 59 metabolites showed that they were typical quantitative traits with large variation, indicating the existence of large-effect genes for most metabolites (Table 1; Additional file 1: Table S2; Fig. 2G–I).

**Fig. 3:**
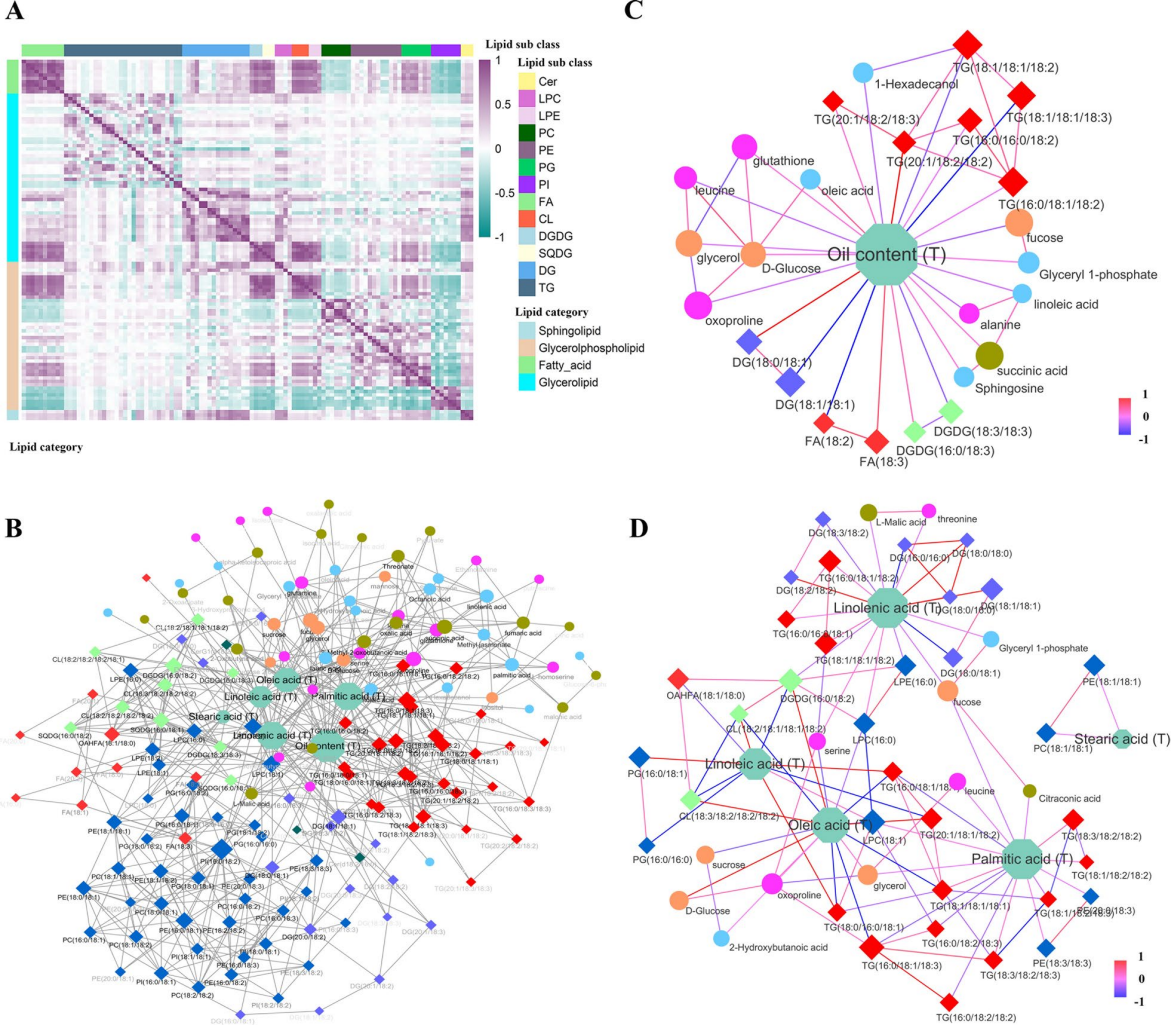
Statistical associations among six seed–oil-related traits, 59 metabolites, and 107 lipids in 398 soybean RILs. **A** Heatmap of genetic correlation for lipids. **B** Networks between metabolites/lipids and oil-related traits were constructed using the Gaussian graphical model, minimax concave penalty, and smoothly clipped absolute deviation methods. The sizes of nodes indicate their degrees in the network. **C**, **D** Cliques around seed oil content and seed fatty acids were extracted from the networks in **B**. The transparency of each label represents the connectivity of each node. Circle node: metabolite; diamond node: lipid; octagon node: seed oil-related trait; orange node: carbohydrate; red node: fatty acids and triglyceride; blue node: lipids measured by GC–TOF–MS; purple node: diglyceride; light green node: glycerolipid; brown: organic acid; pink node: amino acid; dark blue node: phospholipid; dark green node: sphingolipid; the color of line: the size of correlation coefficient

A total of 107 lipids were measured with two biological replicates in NJ2016 using the Q Exactive Orbitrap method. These lipids belong to 15 lipid sub-classes of four categories: fatty acids (10; CV: 104.94–165.28), glycerolipids (50; 24.00–123.48), glycerophospholipids (44; 50.90–122.92), and sphingolipids (3; 57.22–63.84) (Fig. 2J–L; Additional file 1: Table S3). Frequency distributions of CV, skewness, and kurtosis for 107 lipids showed that they were typical quantitative traits with large variation, indicating the existence of large-effect genes for most lipids (Table 1; Additional file 1: Table S3; Fig. 2J–L). Interestingly, in every lipid sub-class, each compound pair was highly correlated (Fig. 3A).

### Genetic relationships among six seed oil-related traits, 59 metabolites, and 107 lipids in 398 soybean RILs

To investigate the trait–metabolite and trait–lipid associations, the average/BLUP for each seed oil-related trait across various environments was used to identify the associations with 59 metabolites and 107 lipids in each biological replicate using minimax concave penalty (MCP) [46] and smoothly clipped absolute deviation penalty (SCAD) [47], as well as *t* test. As a result, 62 trait–metabolite associations in 36 metabolites and 89 trait–lipid associations in 54 lipids were found to be significant (Additional file 1: Tables S4, S5). To investigate the metabolite–metabolite, lipid–lipid, and metabolite–lipid associations, conditional pairwise Pearson correlation coefficients were calculated via Gaussian graphical modeling (GGM) [48]. As a result, 24 metabolite–lipid, 91 metabolite–metabolite, and 348 lipid–lipid associations were identified to be significant (Fig. 3B; Additional file 1: Table S6).

By connecting all the above associations among oil-related traits, metabolites, and lipids, a complex network was constructed. By extracting cliques from this network, 60 oil-related trait cliques were found, including 19 seed oil content, 12 palmitic acid, 1 stearic acid, 12 oleic acid, 7 linoleic acid, and 9 linolenic acid cliques. These cliques revealed the significant correlations between seed–oil-related traits and metabolites/lipids (Fig. 3C, D), e.g., “oil content, 1-Hexadecanol, and TG(18:1/18:1/18:2)” and “oil content, TG(18:1/18:1/18:3), TG(18:1/18:1/18:2), and TG(16:0/18:1/18:2)”.

### Mapping QTLs and QTL-by-environment interactions and predicting their candidate genes for seed oil-related traits in 398 soybean RILs

#### Detection of QTLs and their candidate genes for oil-related traits

**Fig. 4:**
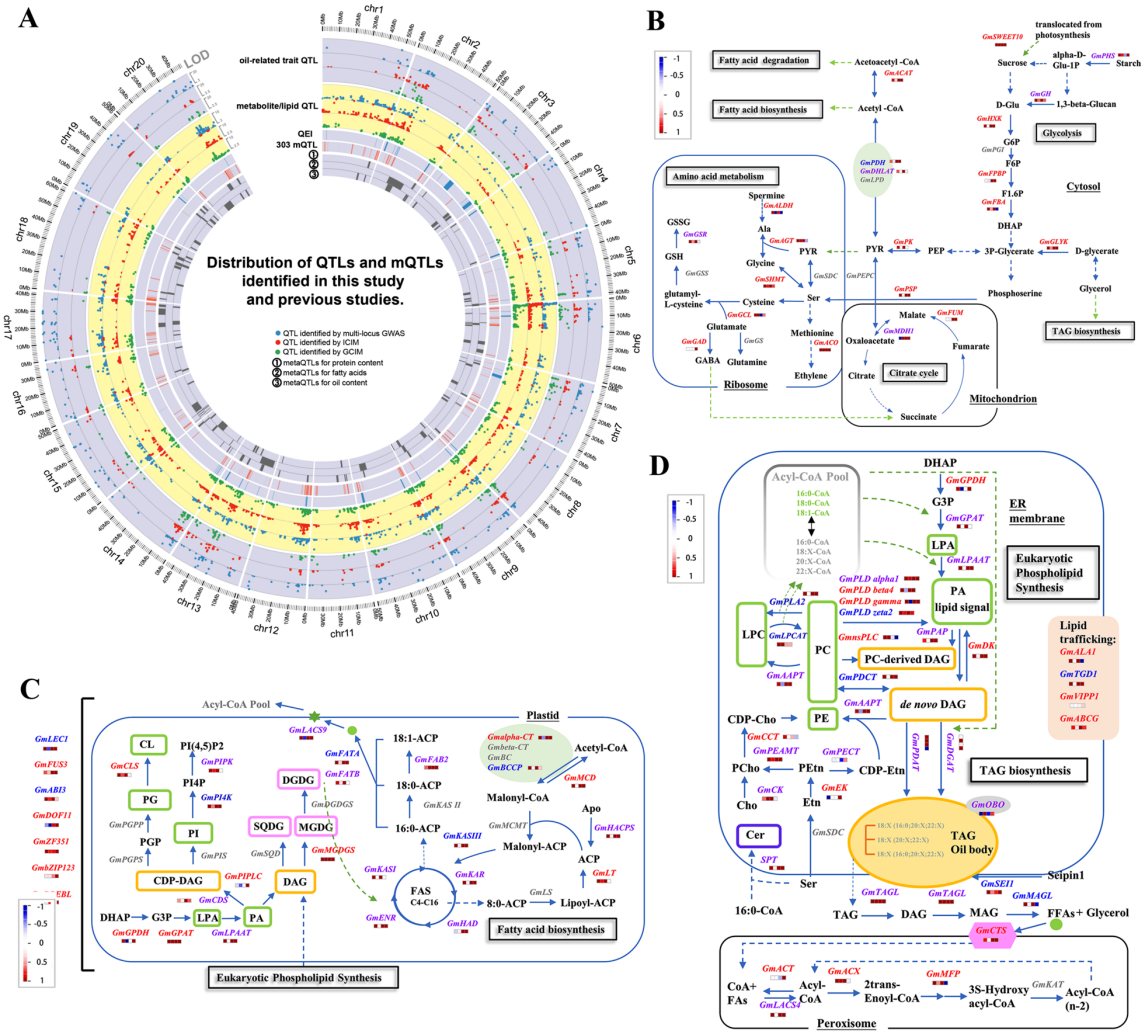
Genomic distribution of QTLs and primary metabolic pathways for metabolites/lipids and oil-related traits. **A** QTLs. Meta-QTLs were derived from Qi et al. [102]. In each circle, the dots with larger LOD score are closer to outer margin. **B** Glycolysis, citrate cycle, and amino acid metabolism. **C** Fatty acid biosynthesis. **D** TAG biosynthesis and eukaryotic phospholipid synthesis. blue: candidate genes for oil-related traits; red: candidate genes for metabolites/lipids; purple: candidate genes commonly for oil-related traits and metabolites/lipids; grey: genes derived from other studies. Four small blocks close to each gene represent log2 (Fold Change) transcript levels between high- and low-oil accessions at four stages (15, 25, 35, and 55 days after flowering). All the abbreviations can be found in Additional file 1: Table S15

To identify QTLs for seed oil-related traits, the phenotypes in each environment and their BLUP values across all the environments were used to associate with 11,846 molecular markers in 398 RILs using the software programs QTL.gCIMapping (GCIM) [49, 50], IciMapping (ICIM) [51], and mrMLM [52]. As a result, among 1222 QTLs for oil-related traits (Additional file 2: Table S7), 175 were identified by at least two approaches and/or in at least two environments (Additional file 1: Table S8; Fig. 4A), including 32 for palmitic acid, 21 for stearic acid, 23 for oleic acid, 40 for linoleic acid, 38 for linolenic acid, 21 for oil content, and 32 for pleiotropy.

To determine candidate genes for oil-related traits around the 175 above-mentioned QTLs, all the genes specifically expressed in seed were identified [53]. Furthermore, 1,390 differentially expressed genes (DEGs) between high- and low-oil accessions were used to prioritize candidate genes [54]. Finally, according to the annotations described in Liu et al. [55], along with Arabidopsis homologous information in ARALIP and soybean pathway annotation in SFGD, 70 candidate genes were mined (Additional file 1: Table S8). Among these genes, 9 soybean genes were confirmed by transgenic experiments in soybean (Table 2), i.e., *GmLEC1-b* [56], *GmFAB2* [9], *GmFatA* and *GmFatB1a* [57], *GmPLDα1* [13], *GmABI3b* [56], *GmDGAT1a* [10], *GmPDAT1* [12], and *GmSEI* [17]. 14 genes are homologs to those in Arabidopsis, which are confirmed by transgenic experiments, e.g., *Atα-PDHC* [58], *AtPLDζ* [59], *AtFAX1* [60], and *AtPDCT* [14]; 32 genes or their homologs in Arabidopsis have been predicted to participate in oil biosynthesis and lipid metabolism; and 15 genes were newly found in this study (Additional file 1: Table S8).

**Table 2:** Five new and ten known candidate genes around stable QTLs for oil-related traits in soybean

Candidate genes for oil-related traits		Quantitative trait locus mapping and genome-wide association studies						Comparative genomics analysis			
		Chr	Markers associated	Effect	LOD score	*r*^2^ (%)	Trait	Gene ID	*Arabidopsis* homologs	Pathway	References
*Gmα-PDHC*	New	3	Marker2405141, Marker2413638	0.07~0.15	3.12~7.15	1.83~6.86	Palmitic acids	*Glyma03g42190*	*At1g01090*	Fatty acid synthesis	Demeirleir et al. [57]
*GmSEI*	New	9	Marker501874, Marker411800	− 0.11~0.08	2.58~3.64	1.85~3.02	Linolenic acid	*Glyma09g38570*	*At5g16460*	Triacylglycerol biosynthesis	Lunn et al. [17]
*GmFAX1*	New	19	Marker1565978, Marker1460807	0~0.06	3.64~38.15	0~5.15	Stearic acid;	*Glyma19g31610*	*At3g57280*	Fatty acid transport	Tian et al. [60]
*GmPLDζ2*	New	15	Marker135457, Marker93680	− 0.22~0.12	2.60~6.11	0.64~3.17	Oil content	*Glyma15g16270*	*At3g16785*	Phospholipid synthesis	Yang et al. [59]
*GmPDCT*	New	7	Marker364973, Marker352632	− 0.79~ 0.46	3.43~10.08	2.24~5.67	Oleic acid; Linoleic acid; linoleic acid	*Glyma07g03350*	*At3g15820*	Triacylglycerol biosynthesis	Lu et al. [14]
*GmLEC1-b*	Known	17	Marker226711, Marker169904	− 0.15~0.07	3.13~3.59	3.06~6.12	Stearic acid	*Glyma17g00950*	*At5g47670*	Transcription factor	Zhang et al. [56],
*GmFAB2*	Known	2	Marker1161043, Marker1192262	− 0.07~ 0.12	5.59~8.85	2.36~4.97	Palmitic acids	*Glyma02g15600*	*At2g43710*	Fatty acid synthesis	Carrero-Colónet al. [8]
*GmFatB1b*	Known	4	Marker2230222, Marker2230222	0.25~0.52	2.75~5.90	1.26~1.90	Linoleic acid	*Glyma04g37420*	*At1g08510*	Fatty acid synthesis	Zhou et al. [57]
*GmFatB1a*	Known	5	Marker2100153, Marker2204980	0.07~0.16	3.45~5.00	1.66~7.47	Linolenic acid; linoleic acid	*Glyma05g08060*	*At1g08510*	Fatty acid synthesis	Zhou et al. [57]
*GmFatA*	Known	8	Marker673687, Marker674654	− 0.10~0.09	2.58~3.93	4.52~6.78	Stearic acid	*Glyma08g46360*	*At3g25110*	Fatty acid synthesis	Zhou et al. [57]
*GmPLDα1*	Known	6	Marker2029409, Marker1949300	0.09~0.16	3.28~6.51	2.07~4.24	Linolenic acid	*Glyma06g07230*	*At3g15730*	Phospholipid synthesis	Zhang et al. [13]
*GmABI3*	Known	8	Marker673687, Marker674654	− 0.10~0.09	2.58~3.93	4.52~6.78	Stearic acid	*Glyma08g47240*	*At3g24650*	Transcription factor	Zhang et al. [56]
*GmDGAT1a*	Known	13	Marker2798086, Marker2790748	0.09~0.12	3.26~19.94	5.29~9.33	Stearic acid; linoleic acid	*Glyma13g16560*	*At2g19450*	Triacylglycerol biosynthesis	Torabi et al. [10]
*GmPDAT1*	Known	13	Marker2850221, Marker2850221	− 0.50~− 0.43	2.84~4.03	1.83~1.97	Stearic acid; linoleic acid	*Glyma13g16790*	*At5g13640*	Triacylglycerol biosynthesis	Liu et al. [12]
*GmGA20OX*	Known	7	Marker288299, Marker366921	− 0.12~− 0.1	3.06~4.81	3.07~3.96	Linolenic acid	*Glyma07g08950*	*At5g51810*	Transcription factor	Lu et al. [108]

#### Detection of QTL-by-environment interactions (QEIs) and their candidate genes for oil-related traits

The above-mentioned multi-environment data sets were used to detect QEIs for oil-related traits using the ICIM method. As a result, a total of 36 significant QEIs were identified, including 7 for palmitic acid, 6 for stearic acid, 7 for oleic acid, 5 for linoleic acid, 9 for linolenic acid, and 2 for oil content (Fig. 4A; Additional file 1: Table S9). Using the same method as described above, 32 candidate genes were identified, including 10 genes in the phospholipid metabolism; for example, *GmPI3P* for palmitic acid, and *GmPIPK-IB* and *GmSac-PIP* for linolenic acid in lipid phosphatidylinositol signaling pathways [61]; *GmPLDβ4* for linoleic acid, *GmPLDα6* for palmitic acid, and *GmPLDζ3* for linolenic acid in PLDs [62]; *GmLPP-ε2* for linoleic acid, *GmPAH1* for oil content, *GmPAH2* for oleic acid, and *GmPAP* for palmitic acid in PPs, which was reported to control the proportions of its substrate phosphatidic acid and diacylglycerol to respond to environmental stress in plants [63].

#### Prediction of candidate miRNAs for oil-related traits

Among 756 mature miRNAs in miRbase (version 22.1), 109 were found to be around the above 175 QTLs. Merging the results from at least two miRNA target prediction methods (psRNAtarget, Target Finder, and psRobot) and co-expression validation, four miRNAs were predicted to directly regulate four candidate oil-related trait genes (Additional file 1: Tables S10, S11). Based on the FIMO results of putative TFBS, 16 miRNAs were predicted to indirectly regulate 37 candidate oil-related trait genes through 10 TFs (Additional file 1: Tables S10–S12). Among these miRNA families, some were reported to be associated with lipid metabolism, e.g., gma-miR156t, gma-miR156i, gma-miR156l, and gma-miR156q in the miR156 family [40], and gma-miR167b in the miR167 family [37, 45]. Among 10 TFs, *Glyma13g29160* (*GmTCP*) was located around oil-related trait QTLs (Additional file 1: Table S8), ARF has been reported to be regulated by miR167 in some crops [37, 45], and *SPL* has been reported to be under the regulation of miR156 in soybean [64].

**Fig. 5:**
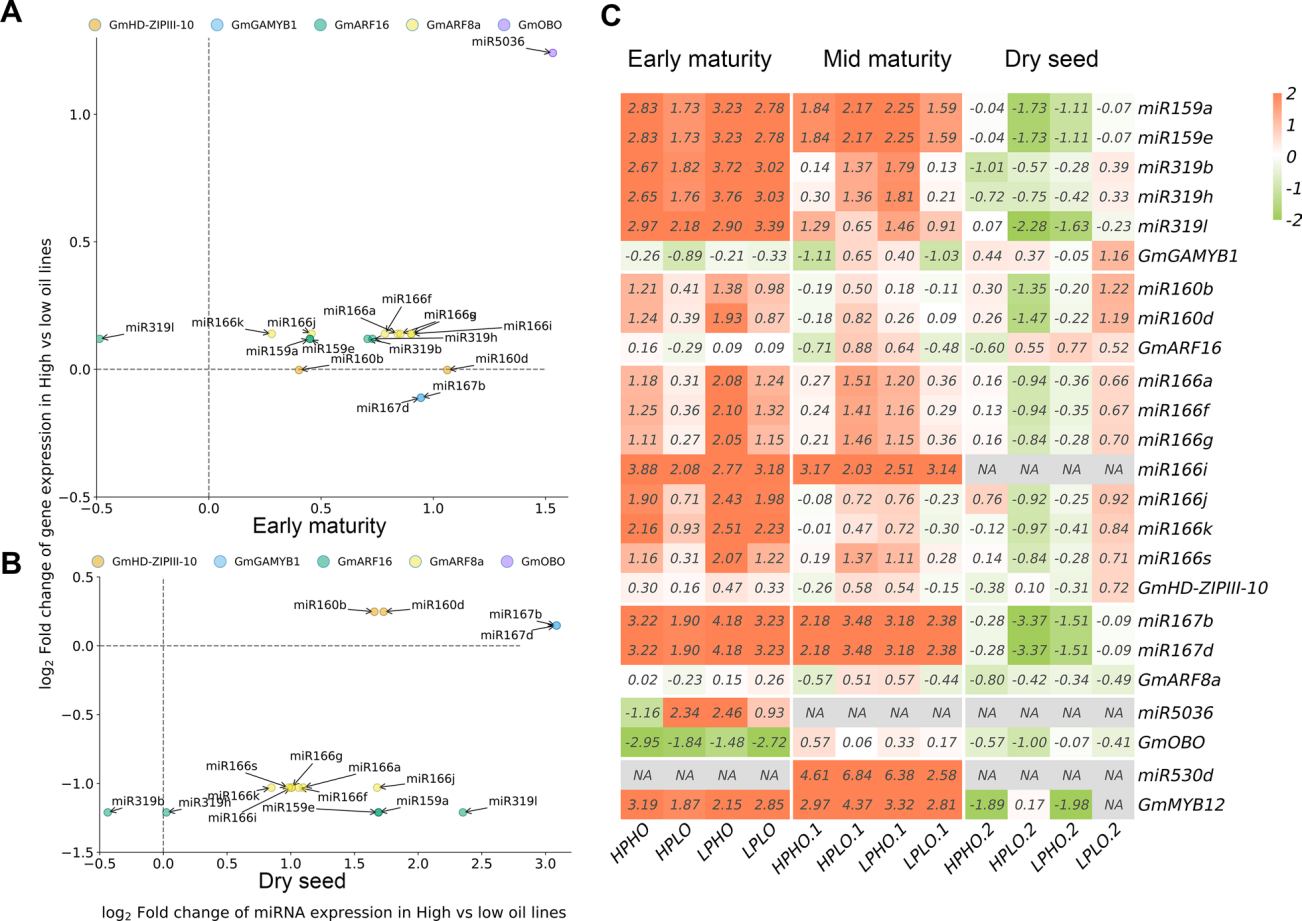
Opposite expression patterns of candidate miRNAs and their target genes in high- and low-oil CSSLs. The log2 (Fold Change) transcript levels between high- and low-oil CSSLs for candidate miRNAs and their target genes at different development stages were shown. **A** Early seed maturity stage. **B** Middle seed maturity stage. Each dot represents the regulation of miRNA and its target gene. The miRNAs are denoted by the arrow, and their target genes are denoted by the color of the dot. **C** Heatmap of expression patterns of CSSLs in different seed development stages. HPHO: log2 (Fold Change) transcript levels between high protein and oil CSSL and control line; HPLO: log2 (Fold Change) transcript levels between high- and low-oil CSSL and control line; LPHO: log2 (Fold Change) transcript levels between low protein and high oil CSSL and control line; LPLO: log2 (Fold Change) transcript levels between low protein and low oil CSSL and control line

To further validate the regulation of miRNAs and their candidate genes at different seed development stages (early and middle maturity stages, and dry seed), expression patterns were inspected in four chromosome segment substitution lines (CSSL) with high or low seed oil content [65]. Between high and low seed oil lines, miR167b and miR167d were found to have negative expression patterns with *Glyma02g40650* (*GmARF8a*) at early seed maturity stages (Fig. 5A), miR159a and miR159e were found to have opposite expression patterns with *Glyma13g25716* (*GmGAMYB1*) in dry seed, and miR319l was found to have negative expression patterns with *GmGAMYB1* in early maturity and dry seed stages (Fig. 5A, B). However, no negative regulations were found in middle maturity stage. As shown in Fig. 5C, four CSSL lines were compared with control lines to exhibit dynamic regulations during seed development.

### Identification of mQTLs and their candidate genes for 59 metabolites and 107 lipids

#### Detection of mQTLs and their candidate genes for metabolites and lipids

To identify mQTLs for seed metabolites and lipids in soybean, their measurements in 2016 were used to associate with SNP markers in 398 RILs using the software programs QTL.gCIMapping [49, 50], IciMapping [51], and mrMLM [52]. As a result, 470 mQTLs were found to be associated with 59 metabolites, including 52 for carbohydrates, 120 for lipids (10 for sphingolipids and 110 for fatty acids), 148 for organic acids, and 150 for amino acids, while 1,306 mQTLs were found to be associated with 107 lipids, including six for sphingolipids, 108 for fatty acids, 370 for glycerophospholipids, and 822 for glycerolipids (Fig. 4A; Additional file 3: Table S13; Additional file 4: Table S14). Moreover, mQTLs found using at least two methods to be associated with metabolites/lipids in the same compound categories were merged into mQTL clusters. As a result, 302 mQTL clusters were identified, including 11 for carbohydrates, 27 for amino acids, 31 for organic acids, three for sphingolipids, 43 for fatty acids, 64 for glycerophospholipids, and 123 for glycerolipids (Additional file 1: Table S15).

Around 302 mQTL clusters, gene annotations and expression levels at 55 DAF were used to mine candidate genes. As a result, 9, 24, 27, 3, 28, 84, and 39 candidate genes were found to be around carbohydrate, amino acid, organic acid, sphingolipid, fatty acid, glycerophospholipid, and glycerolipid QTLs, respectively (Additional file 1: Table S15), while 5, 6, 4, 1, 10, 28, and 16 candidate genes, along with their corresponding metabolites, were predicted to be in the same pathways (Fig. 4B–D). Among the 181 candidate genes, more importantly, 16 candidate genes were confirmed in previous studies, e.g., *Glyma06g12010* (*GmALDH2*) was found to be associated with β-alanine (gmx00260), and *Glyma13g16440* (*GmMDH1*) was found to be associated with isocitric, oxalic, succinic, and citric acids (gmx00020 and gmx00620; Table 3).

**Table 3:** Twelve new and sixteen known candidate genes around mQTLs clusters for metabolites and lipids in soybean

Candidate gene for metabolites and lipids		Quantitative trait locus mapping								Comparative genomics analysis			
		mQTL cluster	Chr	Markers associated	Effect	LOD score	*r*^2^ (%)	Metabolite class	Metabolites and lipids	Gene-name 1.1	*Arabidopsis* homologs	KEGG pathway	References
*GmPHS*	Known	mQTL-C6	8	Marker706158, Marker750703	− 0.97~ 0.52	3.14~3.63	2.48~8.16	Carbohydrates	d-Glucose	*Glyma08g45210*	*AT3G46970*	gmx00500	Satoh et al. [69]
*GmFPBP*	New	mQTL-C4	7	Marker399016, Marker384918	− 0.72~0.69	2.60~2.82	7.33~7.40		Mannose	*Glyma07g17180*	*AT3G54050*	gmx00051	Strand et al. [109]
*GmFBA*	Known	mQTL-C11	13	Marker2849746, Marker2767659	0.45~ 0.76	2.56~2.74	3.06~3.14		Mannose, d-fructose 2,6-bisphosphate	*Glyma13g21540*	*AT2G36460*	gmx00051	Carrera et al. [110]
*GmZF351*	Known	mQTL-F17	6	Marker1996457, Marker2044143	0.27~ 0.35	2.59~3.25	3.47~4.34	Fatty acids	FA(18:0), FA(20:0), FA(22:1)	*Glyma06g44440*	*AT1G03790*		Li et al. [25]
*GmPLDγ*	Known	mQTL-G115	1	Marker1801142, Marker1822692	− 0.44~ 0.46	2.62~4.71	5.92~10.87	Glycerolipids	DG(16:0/16:0), DG(20:0/18:2), DG(20:0/18:3), TG(18:1/18:2/18:3), DG(18:0/16:0), DG(18:0/18:0), DG(18:1/18:1)	*Glyma01g42420*	*AT2G42010*	gmx04144	Bai et al. [16]
*GmPLDε1*	Known	mQTL-G109	15	Marker29446, Marker5035	− 0.27~ 0.26	2.56~5.36	0.24~5.73		DG(16:0/16:0), DG(18:0/16:0), DG(18:0/18:0), DG(18:3/18:3)	*Glyma15g02710*	*AT1G55180*	gmx04144	Yang et al. [59]
*GmPECT1*	Known	mQTL-G21	18	Marker920733, Marker926627	− 0.45~ 0.41	2.72~4.45	5.97~11.39		DG(18:0/18:1), DG(16:0/18:1), DG(18:1/18:1), DG(18:0/18:0), DG(16:0/16:0)	*Glyma18g45210*	*AT2G38670*	gmx00564	Mizoi et al. [111]
*GmnsPLC*	New	mQTL-G40	3	Marker2485779, Marker2406610	− 0.21~ 1.44	3.24~31.92	3.49~10.22		DGDG(16:0/18:2)	*Glyma03g22860*	*AT3G03520*	gmx00564	Cai et al.[15]
*GmDREBL*	New	mQTL-G94	12	Marker2668097, Marker2705284	− 0.12~ 0.05	3.07~4.57	0.46~3.16		DG(20:1/18:2), TG(18:0/16:0/18:1), DG(18:3/18:3), LPC(16:0), LPC(18:0)	*Glyma12g11150*	*AT2G40340*		Zhang et al. [24]
*GmLPAAT4*	Known	mQTL-G9	17	Marker182106, Marker181452	− 0.23~ 0.22	2.51~5.41	0.10~11.01		TG(16:0/16:0/18:2), DG(18:2/18:2), TG(16:0/18:2/18:3), TG(16:0/16:0/18:1), TG(16:0/18:1/18:1), TG(18:3/18:2/18:2), TG(18:1/18:2/18:2), TG(18:0/16:0/18:1), TG(20:0/18:1/18:2), TG(16:0/16:0/18:3)	*Glyma17g36670*	*AT1G75020*	gmx00561	Kim et al. [112]
*GmDGAT1a*	Known	mQTL-G100	13	Marker2790748, Marker2850221	− 0.39~ 0.32	2.83~5.40	0.02~10.08		TG(16:0/18:2/18:3), TG(20:1/18:3/18:3), TG(18:3/18:3/18:3), TG(20:2/18:2/18:2)	*Glyma13g16560*	*AT2G19450*	gmx00561	Torabi et al. [10]
*GmbZIP123*	Known	mQTL-G51	6	Marker1991901, Marker1969292	− 0.33~ 0.22	2.62~4.27	1.46~8.53		TG(18:1/18:1/18:2), TG(20:1/18:1/18:2), TG(16:0/18:1/18:2), SQDG(16:0/18:2)	*Glyma06g01240*	*AT4G34590*		Song et al. [22]
*GmSWEET10a*	Known	mQTL-G112	15	Marker107799, Marker23766	− 0.19~ 0.39	2.81~17.32	0.10~6.87		DG(16:0/18:1), DG(16:0/18:2), DG(18:1/18:2), DG(20:1/18:2), TG(18:1/18:2/18:3)	*Glyma15g05470*	*AT5G13170*	gmx00500	Wang et al. [5]
*GmCK*	Known	mQTL-G33	20	Marker1374027, Marker1406581	− 2.96~ 1.16	2.58~7.15	1.44~7.58	Glycerophospholipids	PC(16:0/18:3), PE(16:0/18:3), PE(18:3/18:2), PE(18:3/18:3), PE(20:0/18:3)	*Glyma20g31030*	*AT1G74320*	gmx00564	Lin et al. [113]
*GmDof11*	Known	mQTL-GP54	13	Marker2818991, Marker2827481	− 1.08~1.02	2.53~3.34	0.22~10.26		PE(16:0/18:1), PE(16:0/18:2), PE(16:0/18:3), PE(18:3/18:2)	*Glyma13g40420*	*AT5G60200*		Wang et al. [26]
*GmFBA*	New	mQTL-O25	14	Marker1738741, Marker1763128	− 0.43~0.45	2.82~4.05	2.70~3.80	Organic acid	d-Glyceric acid	*Glyma14g36850*	*AT2G36460*	gmx01230	Carrera et al. [110]
*GmMDH1*	Known	mQTL-O19	13	Marker2842700, Marker2759137	− 0.90~0.94	2.57~3.68	4.93~9.05		Isocitric acid, oxalic acid, succinic acid, citric acid	*Glyma13g16440*	*AT1G04410*	gmx00020	Kong et al. [19]
*GmGLYK*	New	mQTL-O26	15	Marker114116, Marker114116	0.36	3.17	1.35		l-Malic acid	*Glyma15g01540*	*AT1G80380*	gmx00260	Usuda and Edwards [114]
*GmHXK*	New	mQTL-O30	17	Marker169306, Marker169306	1.34	2.58	6.82		Oxalacetic acid	*Glyma17g37720*	*AT1G47840*	gmx00500	Troncoso-Ponce et al. [115]
*GmAAPT*	New	mQTL-O2	2	Marker1193792, Marker1193792	− 0.91~ − 0.70	4.32~6.56	4.85~7.30		Pyruvate	*Glyma02g14211*	*AT1G13560*	gmx00564	Bai et al. [16]
*GmFUM*	Known	mQTL-O14	9	Marker429142, Marker482975	− 0.54~ − 0.37	2.88~3.94	1.74~3.17		Pyruvate	*Glyma10g02040*	*AT2G47510*	gmx00020	Behal and Oliver [116]
*GmGAD*	New	mQTL-O5	5	Marker2187077, Marker2178818	− 0.62~ 0.54	2.58~5.18	2.49~4.45		Succinic acid	*Glyma05g26660*	*AT2G02010*	gmx00250	Matsuyama et al. [117]
*GmALDH2*	New	mQTL-P8	6	Marker1993733, Marker2000797	− 0.55~ − 0.32	2.87~4.51	3.06~5.77	Amino acid	beta-Alanine	*Glyma06g12010*	*AT1G44170*	gmx00410	Shin et al. [118]
*GmSD*	Known	mQTL-P21	15	Marker12570, Marker12570	− 0.77~0.68	2.87~4.55	2.24~4.15		Ethanolamine	*Glyma15g05630*	*AT1G43710*	gmx00340	Yunus et al. [119]
*GmFUS3*	New	mQTL-P22	16	Marker2528893, Marker2589580	− 0.77~ − 0.50	3.97~4.10	1.94~8.71		Ethanolamine, isoleucine	*Glyma16g05480*	*AT3G26790*		Zhang et al. [24]
*GmPK*	New	mQTL-P26	20	Marker1324457, Marker1324457	− 0.40~ − 0.39	3.14~3.14	1.21~1.29		Leucine	*Glyma20g02980*	*AT5G56350*	gmx00620	Andre et al. [6]
*GmGCL*	New	mQTL-P1	1	Marker1898999, Marker1898999	− 0.74~ 0.70	4.05~6.02	2.62~4.38		Serine	*Glyma01g42900*	*AT4G23100*	gmx00270	Franklin et al. [120]
*GmSTYK*	Known	mQTL-P3	2	Marker1188545, Marker1243816	− 1.15~− 0.70	2.67~2.81	3.08~9.01		Threonine	*Glyma02g43650*	*AT4G08850*		Ramachandiran et al. [76]

#### Co-located QTLs and their candidate genes for oil-related traits and metabolites/lipids

To investigate the genetic basis of correlation between traits and metabolites/lipids, some co-located QTLs were found to be associated with both oil-related traits and metabolites/lipids. As a result, there were 47 common QTLs and 18 common QEIs (Additional file 1: Table S16). Among these common loci, 11 QTLs and 7 QEIs were further identified via MCP and SCAD (Fig. 3B). Around these common loci, 36 and 33 candidate genes were further identified using seed-specific and differential/high expression analyses, respectively (Additional file 1: Table S16). These results were used to construct 3D networks among traits, metabolites/lipids, and their candidate genes.

#### Prediction of candidate miRNAs for metabolites and lipids

Among 756 mature miRNAs in miRbase (version 22.1), 214 were found to be around 302 mQTL clusters. As described in the prediction of candidate miRNAs for oil-related traits, 12 out of 214 miRNAs were predicted to directly regulate 10 candidate genes (Additional file 1: Tables S11, S17, S18), and the *Glyma04g04060* (*GmPAH*)–miR172j and *Glyma17g13120* (*GmOBO*)–miR5036 regulations were consistent with the prediction of Ye et al. [66]. Meanwhile, 46 out of 214 miRNAs were predicted to indirectly regulate 46 candidate genes via 17 TFs (Additional file 1: Tables S11, S17, S18), in which three TFs (*Glyma12g04440*/*GmbZIP44*, *Glyma02g42960*/*GmERF*, and *Glyma04g04060*/*GmPAH2*) were found to be located around mQTL clusters (Additional file 1: Table S15). Among the above 214 miRNA families, 22 were reported to be associated with lipid metabolism, e.g., 16 miRNAs in the miR156 family [40], gma-miR167b, gma-miR167d, gma-miR167k, and gma-miR167l in the miR167 family [37, 45], and gma-miR172j, and gma-miR172f in the miR172 family [40]. Between high and low oil lines, miR167 (miR167b and miR167d) and miR160 (miR160b and miR160d) were found to have opposite expression patterns with *Glyma02g40650* (*GmARF8a*) and *Glyma10g06080* (*GmARF16*), respectively, at early seed maturity stage (Fig. 5A). miR166i was identified to have negative expression patterns with *Glyma08g21610* (*GmHD-ZIPIII-10*) in dry seed (Fig. 5B).

### Construction of GRN and multi-dimensional genetic networks with metabolites, lipids, oil-related traits, candidate genes, and miRNAs

#### GRN for candidate genes, TFs, and miRNAs

**Fig. 6:**
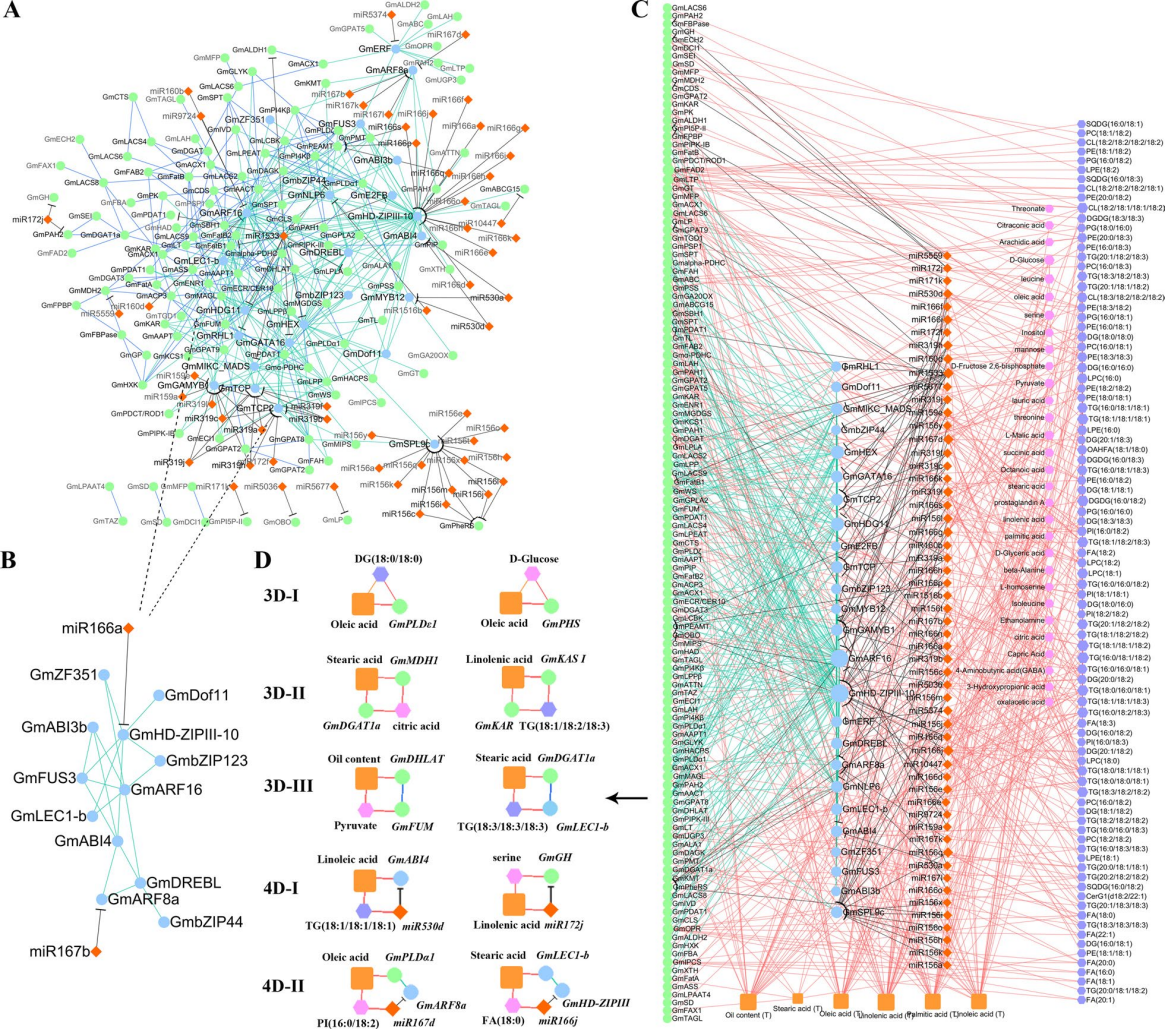
Gene regulatory network and 3D and 4D sub-networks in multi-dimensional genetic network. **A** Gene regulatory network. **B** TF regulatory module. **C** Multi-dimensional genetic network. **D** Examples for 3D and 4D sub-networks among candidate genes (green), TFs (blue), miRNAs (red), metabolites (pink)/lipids (purple), and oil-related traits (orange). Black line: associations between miRNAs and genes; green line: associations between TFs and genes; blue line: associations between a pair of genes; orange line: associations between metabolites/lipids and traits; red line: associations between miRNA/gene/TF and trait/metabolite/lipid

Seed storage accumulation is synchronized through a complex GRN in which TFs act as master regulators. To construct a GRN including candidate genes, TFs, and miRNAs, PPI, TFBS, and miRNA targets were predicted. As a result, the GRN nodes included 56 miRNAs, 25 TFs, and 123 genes, while the edges included 88 miRNA-genes (Fig. 6A; Additional file 1: Tables S10, S17), 241 TF-genes (Additional file 1: Tables S12, S18), and 147 PPIs (Additional file 1: Table S19), which were validated by co-expression analysis (*r*_pcc_ > 0.8; Additional file 1: Table S11).

In this GRN network, some regulations are in agreement with previous studies (Fig. 6B), e.g., miR167 with *ARF8a* [45], *miR166* with *HD-ZIPIII10* [67], miR156 with *SPL9* [64], and the regulation through LAFL transcriptional regulators (*GmLEC1-b*, *GmABI3b*, and *GmFUS3*) [68]. Some regulations were consistent with the predictions of Ye et al. [66], e.g., *GmOBO* with miR5036, and *GmPAH2* with miR172j. More importantly, some regulations were newly identified, e.g., *GmARF16* with miR160b, *GmGAMYB1* with miR159e, *GmARF16* with *GmLEC1-b*, *GmHD-ZIPIII10* with *GmZF351*, and *GmHD-ZIPIII10* with *GmbZIP123*.

#### Construction and validation of the multi-dimensional genetic networks

The metabolite–gene–trait associations obtained in the above genetic analyses, such as metabolite (or lipid) with gene, trait with gene, and trait with metabolite (or lipid), were integrated in the above GRNs to construct an MDGN. As a result, 6 oil-related traits, 30 metabolites, 89 lipids, 56 miRNAs in 17 miRNA families, 25 TFs, and 122 candidate genes were included in the MDGN (Fig. 6C).

In this MDGN, the MCC score of each node along with its topologic characteristics was calculated by CytoHubba (Additional file 1: Table S20). Thus, the hub nodes could be determined, and the sub-networks around the cliques and circuits of these hub nodes caught our attention. As a result, 47 three-dimensional (3D) circulating sub-networks were extracted. In each sub-network, there were three or four nodes that include an oil-related trait, a metabolite/lipid, and a gene (Fig. 6D; Table 3; Additional file 1: Table S21). Some sub-networks were constructed by a commonly associated gene, and significant trait–metabolite/lipid or trait–gene associations, such as oleic acid (Trait)–*GmPLDε1*–DG(18:0/18:0), oleic acid (T)–*GmPHS*–d-glucose, stearic acid (T)–*GmMDH1*–citric acid–*GmDGAT1a*, and linolenic acid (T)–*GmKASI*–TG(18:1/18:2/18:3)–*GmKAR*. Some sub-networks were constructed by PPIs and significant trait–metabolite/lipid associations, such as oil content (T)–*GmDHLAT*–*GmFUM*–pyruvate, and stearic acid (T)–*GmLEC1-b*–*GmDGAT1a*–TG(18:3/18:3/18:3). Among these 3D sub-networks, 35 trait–gene and metabolite/lipid–gene associations were reported in previous studies, such as *GmFAB2–*stearic acid [8], and *GmPHS*–D-glucose [69], and 24 metabolites/lipids–genes were predicted to be in the same pathways, such as *GmFUM*–Pyruvate, *GmPLDε1*–DG(16:0/16:0), and *GmLPAAT5*–TG(18:0/16:0/18:1) (Additional file 1: Table S21).

More importantly, 81 four-dimensional (4D) circulating sub-networks were extracted. In each sub-network, there were four or five nodes that included an oil-related trait, a metabolite/lipid, a miRNA, and a gene (Fig. 6D; Table 4; Additional file 1: Table S21). In these sub-networks, some miRNAs directly regulated candidate genes, such as linoleic acid (T)–*GmABI4*–miR530d–TG(18:1/18:1/18:1), and serine–*GmGH*–miR172j–linolenic acid (T). Some miRNAs targeted TFs that regulated candidate genes, such as FA(18:0)–*GmABI4*–*GmARF8a*–miR167b–oil content (T), and stearic acid (T)–*GmLEC1-b*–*GmHD-ZIPIII-10*–miR166j–FA(18:0). Among these 4D genetic networks, 62 trait–gene and metabolite/lipid–gene associations were reported in previous studies, such as *Glyma06g01240* (*GmbZIP123*)–oil content [22], and *Glyma18g50580* (*GmKASI*)–oil content [70], and 40 metabolites/lipids–genes were predicted to be in the same pathways, such as *GmTAGL–*TG(18:1/18:1/18:3), and *GmLPEAT–*PI(16:0/18:2) (Additional file 1: Table S21).

**Table 4:** Thirty-eight genetic sub-networks that were partly validated by previous molecular biology studies

Subnetworks constructed in this study				Evidences from previous studies	Subnetworks constructed in this study				Evidences from previous studies
No.^a^	Class	Known^b^	Sub-network		No.^a^	Class	Known^b^	Sub-network	
3	3D-I	New	Oil content (T)-*GmDHLAT*-DG(18:0/18:0)	*GPDH*-oil content [9]	60	4D-II	New	DG(18:0/16:0) -*GmPLDγ*-*GmARF16*-miR160b-linolenic acid (T)	*GmPLDγ*-oil content [16]
4	3D-I	New	Oil content (T)-*GmDHLAT*-DG(16:0/16:0)	*GPDH*-oil content [9]	61	4D-II	New	DG(16:0/16:0) -*GmPLDγ*-*GmARF16*-miR160b-linolenic acid (T)	*GmPLDγ*-oil content [16]
7	3D-I	Known	Linolenic acid (T)-*GmCK*-PC(16:0/18:3)	*CK*-PC [72]	62	4D-II	New	DG(18:0/18:0) -*GmPLDγ*-*GmARF16*-miR160b-linolenic acid (T)	*GmPLDγ*-oil content [16]
11	3D-I	New	Oleic acid (T)-*GmPHS*-d-glucose	*GmPHS-*d-glucose [69]	64	4D-II	Known	Oleic acid (T) -*GmPLDα1*-*GmARF8a*-miR167d-PI(16:0/18:2)	*GmPLDα1*-oleic acid [13]
19	3D-II	New	Stearic acid (T)-*GmMDH1*-succinic acid-*GmDGAT1a*	*ChMDH2*-oil content [19], *GmDGAT1*-oil content [10]	65	4D-II	Known	FA(18:0)-*GmABI4*-*GmARF8a*-miR167b-oil content (T)	*ABI3*-oil content [56], miR167-*ARF8a* [45]
20	3D-II	New	Stearic acid (T)-*GmMDH1*-citric acid-*GmDGAT1a*	*ChMDH2*-oil content [19], *GmDGAT1*-oil content [10]	78	4D-II	New	Palmitic acid (T)-*GmPLDα1*-*GmGAMYB1*-miR159a-pyruvate	*GmPLDα1*-oleic acid [13]
22	3D-II	New	Palmitic acids (T)-*GmSPT*-oleic acid-*GmHAD*	*AtHAD*-fatty acids [121]	84	4D-II	Known	TG(16:0/18:1/18:2)-*GmbZIP123*-*GmHD-ZIPIII-10*-miR166s-oil content (T)	*GmbZIP123*-lipid content [22], miR166-*HD-ZIPIII10* [67]
24	3D-II	Known	Linolenic acid (T)-*GmKASI*-TG(18:1/18:2/18:3)-*GmKAR*	*AtKAS*-fatty acids [121]	86	4D-II	Known	TG(18:1/18:1/18:3)-*GmTAGL*-*GmHD-ZIPIII-10*-miR166s-oil content (T)	miR166-*HD-ZIPIII10* [67]
35	3D-III	Known	Oil content (T)-*GmDHLAT*-*GmFUM*-Pyruvate	*GPDH*-palmitic acid [9]	90	4D-II	Known	TG(18:3/18:2/18:2)-*GmFatB1*-*GmHD-ZIPIII-10*-miR166f-oleic acid (T)	*GmFatB*-fatty acid [57], miR166-*HD-ZIPIII10* [67]
37	3D-III	Known	Oleic acid (T)-*GmSEI*-*GmDGAT1a*-TG(16:0/18:2/18:3)	*GmDGAT1*-linolenic acid [9]	100	4D-II	Known	Linolenic acid (T)*-GmPI4Kβ-GmHD-ZIPIII-10-*miR166g-PI(16:0/18:2)	miR166-*HD-ZIPIII10* [67]
38	3D-III	Known	Linoleic acid (T)-*GmPDAT1*-*GmDGAT1a*-TG(16:0/18:2/18:3)	*GmDGAT1*-linolenic acid [9], *GmPDAT*-stearic acid [12]	102	4D-II	Known	Oil content (T)-*GmPLDζ2-GmHD-ZIPIII-10*-miR166j-CerG1(d18:2/22:1)	*GmPLDζ2*-oil content [59], miR166-*HD-ZIPIII10* [67]
39	3D-III	Known	Oil content (T)-*GmPLDζ2*-*GmDAGK*-TG(18:0/16:0/18:1)	*GmPLDζ2*-oil content [59]	105	4D-II	Known	Stearic acid (T)-*GmLEC1-b-GmHD-ZIPIII-10*-miR166j-FA(18:0)	*AtLEC1*-stearic acid [58], miR166-*HD-ZIPIII10* [67]
40	3D-III	Known	Stearic acid (T)-*GmLEC1-b*-*GmDGAT1a*-TG(18:3/18:3/18:3)	*AtLEC1*-stearic acid [56], *GmDGAT1*-linolenic acid [9]	114	4D-II	Known	FA(22:1)-*GmZF351*-*GmHD-ZIPIII-10*-miR166k-linoleic acid (T)	*GmZF351*-oil content [25], miR166-*HD-ZIPIII10* [67]
44	3D-III	New	Palmitic acid (T)-*GmBCCP1*-*GmFUS3*-ethanolamine	*AtFUS3*-palmitic acid [21]	118	4D-II	New	Stearic acid (T)-*GmABI3b*-*GmNLP6*-miR1516b-palmitic acid	*ABI3*-oil content [56]
45	3D-III	Known	Linolenic acid (T)-*GmKAR*-*GmPK*-leucine	*AtPK*-linoleic acid [6]	119	4D-II	New	Stearic acid (T)-*GmLEC1-b*-*GmNLP6*-miR1516b-palmitic acid	*AtLEC1*-stearic acid [56]
49	4D-I	New	Linoleic acid (T)-*GmABI4*-miR530d-TG(18:1/18:1/18:1)	*ABI3*-oil content [56]	121	4D-II	New	PE(16:0/18:1)-*GmDof11*-*GmNLP6*-miR1516b-palmitic acid (T)	*GmDof11*-lipid content [26]
53	4D-I	New	Linolenic acid (T)-*GmOBO*-miR5036-TG(16:0/16:0/18:1)	*OBO*-miR5036 [66]	125	4D-II	New	LPC(16:0)-*GmDREBL*-*GmTCP*-miR319h-linolenic acid (T)	*GmDREBL*-lipid content [24]
55	4D-II	New	Oleic acid (T)-*GmPLDα1*-*GmARF16*-miR160d-TG(18:0/16:0/18:1)	*GmPLDα1*-oleic acid [13]	127	4D-II	New	Linolenic acid (T)-*GmbZIP123*-*GmTCP*-miR319h-TG(16:0/18:1/18:2)	*GmbZIP123*-lipid content [22]
59	4D-II	New	Stearic acid (T)-*GmLEC1-b*-*GmARF16*-miR160d-TG(18:0/18:1/18:1)	*AtLEC1*-stearic acid [56]	128	4D-II	New	Oil content (T)-*GmDHLAT*-*GmTCP2*-miR319f-TG(18:1/18:1/18:3)	*GPDH*-oil content [9]

^a^The number of sub-networks in Additional file 1: Table S21

^b^‘Known’ subnetworks could be found at the KEGG PATHWAY website (https://www.kegg.jp/kegg/pathway.html) and ‘new’ subnetworks were constructed in this study

### Validation of sub-networks corresponding to the oil content and linolenic acid traits

**Fig. 7:**
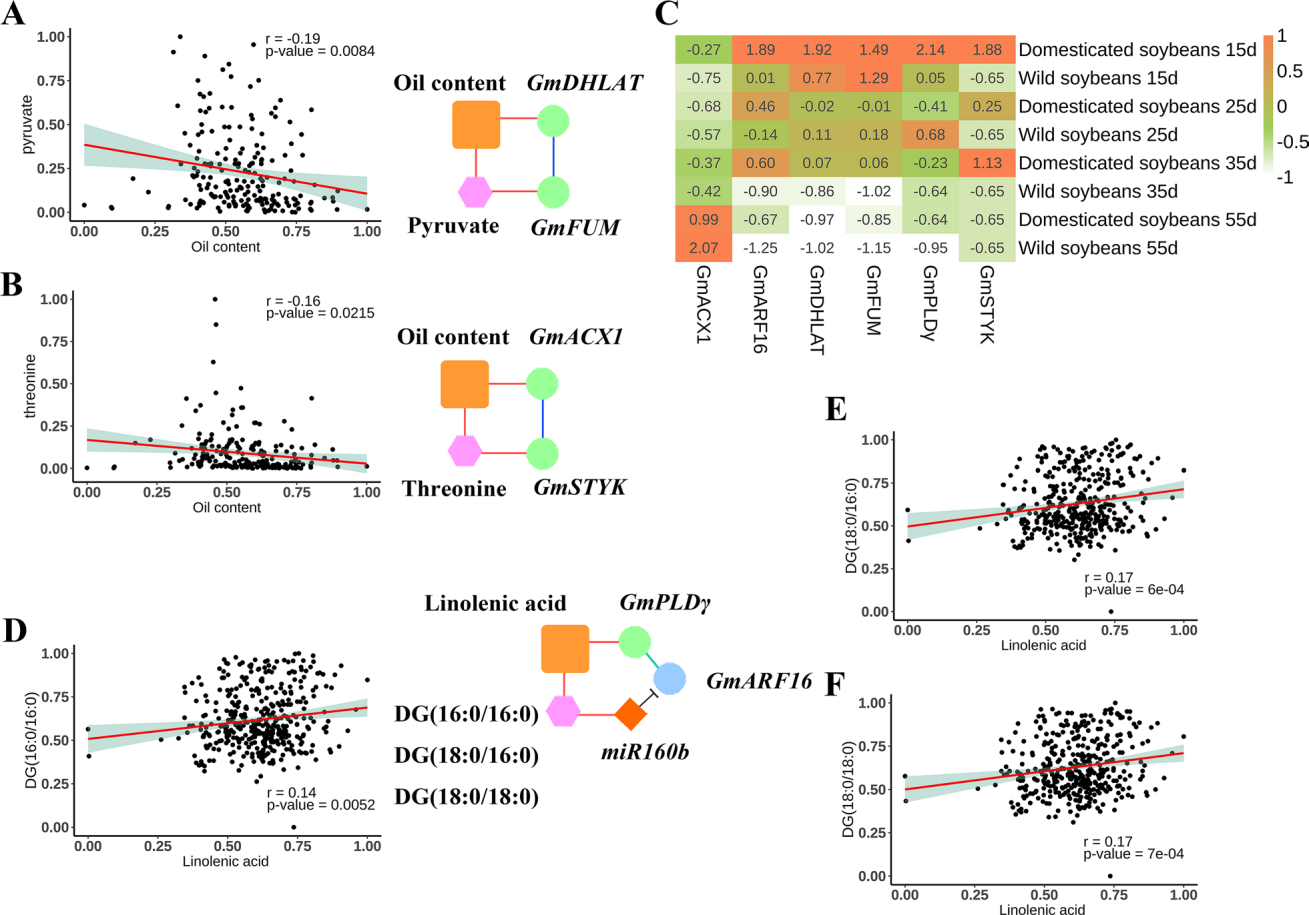
3D and 4D sub-networks of significant nodes of metabolites and genes in six soybean accessions. **A**, **B**, and **D**–**F** Pearson correlation analysis between one metabolite and one oil-related trait. **C** Heatmap of average RPKM values of six genes expressed in four domesticated soybeans with high seed oil content and two wild soybeans with low seed oil content at four seed development stages

To provide useful information for soybean breeding for oil content and fatty acid composition, we validated the sub-network in this study by combining the metabolites, oil-related traits, and expression profiling data in natural population of Liu et al. [55]. We found that 26 trait–gene associations in 133 3D sub-networks of Liu et al. [55] were also observed in this study. 11 metabolite nodes in sub-networks of this study were found to be significant in the hypothesis tests between five high-oil and five low-oil soybean accessions [55] (Additional file 1: Table S22). All the candidate gene expression profiling in seeds was found to be significant between domesticated and wild soybeans at four stages (15, 25, 35, and 55 days after flowering) (Fig. 4B–D). We found two 3D sub-networks, oil content (T)–*GmDHLAT*–*GmFUM*–pyruvate and oil content (T)–*GmACX1*–*GmSTYK*–threonine (Fig. 7A–C), and 4D sub-network DG(16:0/16:0)/DG(18:0/16:0)/DG(18:0/18:0)–*GmPLDγ–GmARF16*–miR160b–linolenic acid (T) (Fig. 7C–F), in which the metabolite and gene nodes were significant between domesticated and wild soybeans.

## Discussion

In this study, 175 QTLs for oil-related traits, 302 mQTL clusters for metabolites/lipids, and 62 trait–metabolite, 89 trait–lipid, 24 metabolite–lipid, 91 metabolite–metabolite, and 348 lipid–lipid associations were identified. Around these QTLs and mQTL clusters, 70 and 181 candidate genes, and 20 and 58 miRNAs, were, respectively, mined. Homologs of 46 and 70 genes for oil-related traits and metabolites were validated in previous molecular experiments. Using bioinformatics predictions, candidate genes, TFs, and miRNAs were used to construct a GRN. The above results of genetic analyses were integrated with the GRN to construct an MDGN. In this network, 47 3D and 81 4D circulating sub-networks were relatively reliable. The reasons are as follows. First, genes, metabolites, and lipids in 64 circulating sub-networks were found to participate in common pathways (Additional file 1: Table S21). In other words, these sub-networks are supported by prior knowledge. Then, 26 trait–gene associations in 133 3D sub-networks of Liu et al. [55] were obtained in this study (Additional file 1: Table S22). More importantly, this study is novel in three aspects. First, more metabolites/lipids (166) were measured in 398 RILs in this study than those (52) in 214 accessions in Liu et al. [55], e.g., glucose, fucose, ethanolamine, DAG, and TAG. Second, miRNAs and new regulations were included in MDGN in this study that were not in Liu et al. [55], e.g., miR167d–*GmARF8a*, miR160b–*GmARF16*, and miR166s–*GmHD-ZIPIII-10*. Finally, all the 47 3D and 81 4D circular sub-networks were more reliable, because all the edges in sub-networks were found to be significant.

### Candidate genes newly discovered for metabolites/lipids and oil-related traits

To address the genetic basis of metabolites/lipids in soybean, 5, 6, 4, 10, 28, and 16 candidate genes for carbohydrate, amino acid, organic acid, fatty acid, glycerophospholipid, and glycerolipid were found to participate in common metabolic pathways using mQTL mapping, seed-specific expression profiling, high/low oil differential expression, and information from the model species Arabidopsis (Table 3). Using the co-located QTLs via modern statistical methods, some metabolite/lipid candidate genes were found to be associated with oil biosynthesis, such as *GmSWEET10a* [5], *GmPLDγ* [16], and *GmPDAT1* [12], and some homologs were also found to be associated with oil biosynthesis, such as *GmMDH1* [19], *GmPK* [6], and *GmnsPLC* [15].

### Key regulations associated with oil-related traits and lipid metabolism

According to molecular biology research, genes are regulated by other genes, TFs, and miRNAs. In this study, candidate genes of both oil-related traits and metabolites/lipids and predicted TFs and miRNAs were used to construct a GRN (Fig. 6A). In the GRN, some TFs were identified in previous studies, e.g., *GmLEC1-b*, *GmABI4*, *GmABI3b* [56], and *GmFUS3* [21] in the LAFL network of Lepiniec et al. [68]. More known oil-biosynthesis TFs were found in mQTL mapping of metabolites/lipids than in QTL mapping of oil-related traits, e.g., *GmZF351*, *GmDREBL*, *GmDof11*, and *GmbZIP123* [22, 24–26]. More importantly, some new TFs which were validated to regulate development were predicted to regulate oil-biosynthesis in this study, e.g., *GmHD–ZIPIII10*, *GmARF16*, *GmARF8a*, and *GmGAMYB1*, in which some interacted with LAFL and other known oil synthesis genes, e.g., *GmHD–ZIPIII10* interacted with *GmFatB1* and *GmPLDζ2*, and *GmARF16* interacted with *GmbZIP123*, *GmZF351*, *GmPLDα1*, and *GmLEC1-b* (Fig. 6B; Additional file 1: Table S21).

In the GRN, some miRNAs and their regulations were identified in previous studies, e.g., miR166 targeted *GmHD–ZIPIII-10* [67], miR167 targeted *GmARF8a* [37, 45], and miR156 targeted *GmSPL9* [64]. Meanwhile, mR156, miR166, and miR167, along with their targeted genes, have been proved to regulate the accumulation of storage compounds during seed maturation in the miRNA–LAFL mediated network of Tang et al. [71] and Lepiniec et al. [68]. Moreover, some new miRNAs and their regulations were predicted in this study, e.g., miR160b and miR160d targeted *GmARF16*, which subsequently regulated *GmLEC1-b*, *GmABI3b*, and *GmFUS3* that are involved in the LAFL network (Fig. 6B), and miR319h targeted *GmTCP*, which subsequently regulated *GmDREBL*, *GmLEC1-b*, and *GmbZIP123* (Fig. 6A). Of course, these regulations should be further validated via molecular biology experiments, because the transcriptional levels for these candidate genes, predicted TFs, and miRNAs are dynamic and tissue-specific.

The above results are frequently found in the single dimensional genetic analyses and molecular biology research of both oil-related traits and metabolites. However, studies on the network analysis of oil-related traits, metabolites, genes, TFs, and miRNAs are limited. To address this issue, we constructed the MDGN in this study.

### Dissection of genetic basis for oil-related traits using multi-dimension genetic network

Metabolites bridge genes and complex traits [2]. Recently, Shi et al. [72] reported the genetic relationships between 4-indolecarbaldehyde/tryptophan and the number of grains per spike in wheat, and Liu et al. [55] constructed 3D genetic networks, revealing the genetic relationships between oil-related traits and acyl-lipid-related metabolites. In this study, we extended 3D genetic network into MDGN and found two types of sub-networks, which are used to reveal the potential genetic basis for both oil-related traits and metabolites/lipids. One was 3D sub-networks based on candidate genes that were commonly identified to be associated with both oil-related traits and metabolites/lipids, while another was 4D sub-networks based on indirect interactions of candidate genes, TFs, and miRNAs (Additional file 1: Table S21). Two examples are described below.

#### 3D genetic sub-networks revealed genetic relationships between seed carbohydrates, oil, and protein content

Soybean is not only one of the largest sources of oil for food and feed but also the protein source of the animal feed in which the level of essential amino acids in feed rations can impact meat qualities. Genetic engineering of genes encoding enzymes related to the flow of carbon into seed oil has led to significant increases in seed oil and protein content [4–6]. In our MDGN, there are three 3D circulating sub-networks, including oil content (T)–*GmDHLAT*–*GmFUM*–pyruvate, oil content (T)–*GmACX1*–*GmSTYK*–threonine, and stearic acid (T)–*GmMDH1*–succinic acid/citric acid–*GmDGAT1a* (Table 4). There has been some evidence to validate these sub-networks. In metabolites, first, pyruvate, oxaloacetate, succinic acid, and citric acid are involved in the citrate cycle (gmx00020). The phosphoenolpyruvate–pyruvate–oxaloacetate node is known as the switch point for carbon flux distribution [73], and pyruvate is the main precursor in fatty acid synthesis [74]. Threonine is considered as the most limiting essential amino acid in the aspartate family pathway with regulatory metabolic link of TCA cycle [75]. In oil synthesis-related genes, second, *GmDHLAT*, *GmMDH*, and *GmFUM* participated in the citrate cycle to catalyze pyruvate, oxaloacetate, and malate, respectively [9], and these genes were found to have higher expression at middle seed maturity stage than at other stages (Fig. 4B). The MDH activity in isolated embryos was reported to correlate with embryo oil and knocking out the peroxisome-located *MDH2* in Chlamydomonas results in alterations in fatty acid metabolism [19]. *STYK* can phosphorylate oil body proteins and regulate the oil content in Arabidopsis seeds [76]. Based on the above information, we deduce that threonine, pyruvate, oxaloacetate, and malate may play important roles in the flow of carbon into seed storage oil and protein content through the action of *GmMDH*, *GmFUM*, *GmDHLAT*, and *GmSTYK*.

#### 4D genetic sub-networks around PLDs revealed the effect of phospholipid metabolism on oil-related traits

Recent studies showed that acyl editing and phospholipid turnover influenced storage lipid production and oil-related traits [13, 16, 59]. In previous studies, PLD enzymes were found not only to determine seed viability and respond to environments but also to alter oil quality [13, 16, 62]. However, the regulations behind phospholipid metabolism are still unclear [15]. In this study, three 4D sub-networks with three PLDs may be helpful to solve this problem, i.e., DG(16:0/16:0)/DG(18:0/16:0)/DG(18:0/18:0)–*GmPLDγ–GmARF16*–miR160b–linolenic acid (T), palmitic acid (T)–*GmPLDα6*–*GmGAMYB1*–miR319l–PE(18:3/18:3), and oleic acid (T)–*GmPLDα1*–*GmARF8a*–miR167d–PI(16:0/18:2) (Table 4). These sub-networks are reliable. First, the nodes in each sub-network were found in this study to be significantly associated with their adjacent node to form a circulating sub-network. Interestingly, *GmPLDα6* was found to be significantly associated with palmitic acid in QEI detections (Additional file 1: Table S9), which may indicate the influence of environmental factors. Second, all the metabolites and genes in the above nodes participate in phospholipid metabolism (Additional file 1: Table S21). The DG pool is a key branch point in acyl editing, while PE is one of the substrates of the PLD enzyme [62]. Finally, some relationships in these sub-networks are consistent with previous studies. *PLDα1*-knockdown soybean seeds increased TAG unsaturation and modified PE content [13]; *GmPLDγ* influenced seed oil content and fatty acid composition in transgenic Arabidopsis [16]; miR167a mediated LAFL module through *CsARF8* in Camelina sativa seed [45]. Interestingly, miR167 and *GmARF8a* exhibit opposite expression patterns during early seed development stage (Fig. 5A, C), while miR160b and *GmARF16* exhibit more dynamic expression during seed development (Fig. 5A, C). Thus, these 4D sub-networks can aid to guide molecular experiments in the future to unveil the regulation mechanisms between oil-related traits and phospholipid metabolism.

In this study, not only linkage analysis (GCIM [50, 51] and ICIM [52]) but also genome-wide association studies (mrMLM [53]) were used to identify QTNs for oil-related traits and metabolites/lipids in 398 RILs. Although GCIM can detect more small-effect and linked QTNs than ICIM [50, 77], and genome-wide association studies can detect more small-effect QTNs than linkage analysis [78], especially, each method can identify some method-specific QTNs. In other words, these methods are complementary to each other. Thus, these methods were simultaneously adopted in our study.

In this study, three various data sets were used to construct microRNA/gene expression networks. Ideally, all the three data sets should be the same as regards varieties, sampling times, and environments. However, various data sets are also used to construct networks in previous studies. For example, Yang et al. [79] constructed a metabolic regulatory network using metabolome and transcriptome data sets, which were collected from different environments and years [79, 80]. Chen et al. [81] constructed a GRN controlling flower development in *Arabidopsis thaliana* using 85 data sets from 15 previous studies. In this study, all the relationships in the MGDNs were obtained using commonly used approaches. First, the relationships between oil-related traits and metabolites/lipids were obtained from MCP [46], SCAD [47], and GGM [48]. Candidate genes for oil-related traits in linkage and association analyses were related to lipid-metabolism [34], highly expressed in seed, and differentially expressed between high- and low-oil accessions. Candidate genes for lipids/metabolites in linkage and association analyses were obtained from lipid/metabolite metabolism pathways (https://www.kegg.jp/kegg/pathway.html). Then, the relationships between candidate genes and TFs were obtained from co-expression analysis, PPI (https://string-db.org/cgi/input.pl), and TFBS predictions [82]. Here 8 out of 17 TFs were previously reported to be associated with oil biosynthesis (Additional file 1: Table S15). Finally, the relationships between candidate genes and miRNAs were obtained from three miRNA target predictions [83–85] and expression pattern analyses of Yu et al. [65]. Here 12 out of 26 relationships were supported by the literature in Additional file 1: Table S21. Therefore, the results in this study are relatively reliable. More importantly, we proposed a novel method of constructing 4D networks in this study. In this sense, the present study is valuable.

## Conclusions

In this study, 70 candidate genes around 175 trait QTLs, 32 candidate genes around 36 QEIs, and 181 candidate genes around 326 mQTLs clusters were identified, including 46 and 70 known homologs identified to be associated with the traits and metabolites, respectively. Among these candidate genes, 15 trait genes and 27 metabolite/lipid genes were previously reported. Based on all the candidate genes, the PPI, co-expression analysis, and TFBS and miRNA target predictions were used to construct GRNs, in which some TFs and miRNAs were newly identified, e.g., *GmHD–ZIPIII10*, *GmARF16*, *GmARF8a*, *GmGAMYB1*, mR156, miR166, and miR167. All the genetic analysis results were integrated with GRNs to construct MDGNs, in which 47 3D and 81 4D circulating sub-networks might reveal the genetic relationships between metabolites/lipids and oil-related traits. Among the 128 sub-networks, 64 were consistent with previous studies, such as oil content (T)–*GmDHLAT*–*GmFUM*–pyruvate, and the others were newly identified, such as FA(18:0)–*GmABI4*–*GmARF8a*–miR167b–oil content. This study provides an example of system network analyses, and the genetic foundations of metabolites/lipids and oil-related traits.

## Materials and methods

### Recombinant inbred lines (RILs) for trait and metabolic QTL mapping

As described in Zuo et al. [86], 398 RILs derived from orthogonal (171, OC) and reciprocal crosses (227, RC) between two parents LSZZH (P1) and NN4931 (P2) in soybean (*Glycine max*) were planted at Jiangpu (E 118° 22′, N 31° 14′) experimental station of Nanjing Agricultural University in 2015 (NJ2015) and 2016 (NJ2016), and Wuhan (E 114° 21′, N 30° 29′) and Ezhou (E 114° 54′, N 30° 23′) experimental stations of Huazhong Agricultural University, respectively, in 2014 (WH2014) and 2015 (EZ2015). Detailed information was described in previous reports [54, 86]. Seeds for five plants in the middle row for each RIL were randomly harvested at 55 days after flowering (DAF), and the mixture with at least three pods each from different plants was stored at − 80 °C before extraction for the measurements of metabolites and lipids. The mixture of dry seeds for each RIL was used for the measurements of six seed oil-related traits.

### Measurements for six oil-related traits in 398 RILs

As described in Zhou et al. [54], the method of Baydar and Akkurt [87] was used to measure seed oil content, palmitic acid, stearic acid, oleic acid, linoleic acid, and linolenic acid. 10 g of seeds collected from each RIL were ground, and the seed powder was filtered. 30 mg seed powder was used to measure six oil-related traits by gas chromatography with a flame ionization detector and a Permabond FFAP stainless steel column (50 m × 0.2 mm × 0.33 µm, Thermo Fisher Scientific, Waltham, MA) at the Wuhan Research Branch of the National Rapeseed Genetic Improvement Center in 2014 and 2015, and at the State Key Laboratory of Crop Genetics and Germplasm Enhancement of Nanjing Agricultural University in 2015.

### Metabolites and lipids extraction

Metabolites and lipids were extracted from seeds planted in Nanjing (NJ2016) according to a protocol adapted from Bligh and Dyer [88] and Lisec et al. [89]. Seed powder was used to measure the metabolites and lipids at Biotree Biotech Co., Ltd (Shanghai, China, http://www.biotree.cn/). 200 mg ± 1 mg seed powder for each sample was placed in 2 mL EP tubes, and 0.4 mL dH2O and 0.96 mL extraction liquid (*V*_MTBE_:*V*_methanol_ = 5:1, methyl tertbutyl ether) were added. Subsequently, the samples were homogenized in a ball mill for 4 min at 45 Hz and ultrasound treated for 5 min (incubated in ice water). Then, centrifugation was conducted for 15 min at 16,200 g^−1^ at 4 °C, followed by incubation for 1 h at − 20 °C. Pooling the organic phase from the two parallel samples, the extraction was dried at room temperature under a gentle stream of nitrogen gas. The dry extraction was reconstituted with 900 μL MTBE (methyl tertbutyl ether). The lipid profiling and gas chromatography tandem time-of-flight mass spectrometry (GC–TOF–MS) profiling was conducted by transferring 200 μL samples into 1.5 mL EP tube vials, respectively. A QC sample was pooled by taking 100 μL MTBE reconstitution from each sample, which was divided into 30 aliquots for lipid profiling and 80 aliquots for GC–TOF–MS profiling, with an average volume of 200 μL. The reconstitution of lipid profiling and GC–TOF–MS profiling was conducted with 200 μL extraction liquid (*V*_dichloromethane_:*V*_methanol_ = 2:1).

### Measurement for metabolites using GC–TOF–MS

The metabolites in each sample were measured by GC–TOF–MS. Metabolite derivatization was conducted as follows: first, samples were dried in a vacuum concentrator without heating, then 30 μL of methoxy amine hydrochloride (20 mg/mL in pyridine) was added into the metabolite samples by incubating for 30 min at 80 °C. Subsequently, 40 μL of *N*,*O*-Bis(trimethylsilyl)trifluoroacetamide (BSTFA) regent was added to the sample aliquots by incubating for 1.5 h at 70 °C. 5 μL FAMEs (standard mixture of fatty acid methyl esters, C8–C16:1 mg/mL, C18–C24:0.5 mg/mL in chloroform) was added to the QC sample. For the GC–TOF–MS pipeline, analysis was performed using an Agilent 7890 gas chromatograph system coupled with a Pegasus HT time-of-flight mass spectrometer. The system utilized a DB-5MS capillary column coated with 5% diphenyl cross-linked with 95% dimethylpolysiloxane (30 m × 250 μm inner diameter, 0.25 μm film thickness; J&W Scientific, Folsom, CA, USA). A 1 μL aliquot of the analyte was injected in a splitless mode. Helium was used as the carrier gas, the front inlet purge flow was 3 mL min^−1^, and the gas flow rate through the column was 1 mL min^−1^. The initial temperature was kept at 50 °C for 1 min, then raised to 310 °C at a rate of 10 °C min^−1^, then kept for 5 min at 310 °C. The injection, transfer line, and ion source temperatures were 280, 270, and 220 °C, respectively. The energy was 70 eV in electron impact mode. The mass spectrometry data were acquired in full-scan mode with the *m*/*z* range of 50–500 at a rate of 20 spectra per second after a solvent delay of 6.1 min.

Chroma TOF 4.3X software of LECO Corporation and LECO-Fiehn Rtx5 database was used for the exacting of raw peaks, filtering, and calibration of the data baselines, peak alignment, deconvolution analysis, peak identification, and integration of the peak area [90]. Metabolic features detected < 50% of QC samples were removed [91]. The number of biological replicates for each line was two.

### Measurement for lipids using Q Exactive Orbitrap LC–MS/MS

Lipidomic data were obtained using a Q Exactive Orbitrap LC–MS/MS (Thermo Fisher Scientific, USA) system. The extracted lipid was redissolved by chloroform just before analysis. The experimental procedures were carried out according to Tang et al. [92]. In the HPLC (High-Performance Liquid Chromatography) methods, reverse phase chromatography Cortecs C18 column (2.1 × 100 mm, Waters) was connected to a Thermo Fisher Scientific Autosampler and to a UPLC pump. 1 μL supernatant was loaded on a normal phase chromatography column, then the sample was eluted to an orbitrap mass spectrometer with IPA:CAN = 90:10 as eluent. Positive–negative ion switching mode was performed for sample data acquisition. The QC data were acquired at positive ion and negative ion mode separately using data dependent MS/MS acquisition. The full scan and fragment spectra were collected with a resolution of 70,000 and 17,500, respectively. The source parameters were as follows: spray voltage: 3000 V; capillary temperature: 320 °C; heater temperature: 300 °C; sheath gas flow rate (Arb): 35; auxiliary gas flow rate (Arb): 10.

Lipidomics identification was performed using the analytical software *LipidSearch* (Thermo Fisher, CA). Mass tolerance for precursor and fragment was set to 8 ppm and 15 ppm, respectively. Adducts of H+, NH4+ were applied for positive mode search, and H−, CH3COO+ were selected for negative mode, since ammonium acetate was used in the mobile phases [93]. The number of biological replicates for each line was two. As such, triglycerides (TG), diglycerides (DG), ceramide (Cer), and galactosylcerebroside (CerG) displayed better responses under positive ion mode, whereas lysophosphatidylethanolamine (LPE), lysophosphatidylcholine (LPC), fatty acids (FA) ω-hydroxy fatty acid (OAHFA), digalactosyldiacylglycerol (DGDG), sulfoquinovosyldiacylglycerols (SQDG), phosphatidylcholines (PC), phosphatidylethanolamine (PE), phosphatidylinositol (PI), phosphatidylglycerols (PG), and cardiolipin (CL) were detected under negative ion mode.

### Statistical analysis and variable selection among oil-related traits and metabolites

The metabolites and lipids data were log_2_-transformed for statistical analysis as usual [94]. MCP [46] and SCAD [47] along with *t* test were used to determine the genetic associations of oil traits with metabolites (or lipids). Statistical significance was computed using *F* test for the total regression of each oil-related trait on several metabolites (or lipids) and *t* test for the regression of each oil-related trait on each metabolite. The ‘ncvreg’ R package (from the CRAN, http://www.cran.r-project.org/) was used to implement the SCAD and MCP methods [95].

The genetic correlation coefficients ($r_{G(i,j)}$) were calculated by

$$r_{G(i,j)} = {{{\text{COV}}_{G(i,j)} } \mathord{\left/ {\vphantom {{{\text{COV}}_{G(i,j)} } {\sqrt {\sigma_{G(i)}^{2} \sigma_{G(j)}^{2} } }}} \right. \kern-\nulldelimiterspace} {\sqrt {\sigma_{G(i)}^{2} \sigma_{G(j)}^{2} } }}$$

where ${\text{COV}}_{G(i,j)}$ is the covariance between metabolites *i* and *j*, and $\sigma_{G(i)}^{2}$ is the variance for metabolite *i*. Two-way ANOVA was conducted in R.

GGM is an undirected probabilistic graphical model based on pairwise Pearson correlation coefficients conditioned against the correlation with all other metabolites [48]. GGM and the Bonferroni correction were used to identify the associations between metabolites and lipids. The ‘GeneNet’ package 1.2.8 [96] (from the CRAN, http://www.cran.r-project.org/) was used to estimate the Pearson correlation. A significant *P* value < 4.19E−07 (0.05/119,316) was applied to filter the results. The BLUPs of all the RILs for each seed oil-related trait across various environments were calculated by R with ‘lme4’ package.

### QTL mapping for oil-related traits, metabolites, and lipids

Using the high-density genetic maps constructed in 398 RILs by Zuo et al. [86], GCIM [50] (genome-wide composite interval mapping) and ICIM [51] (inclusive CIM) were used to detect QTLs for oil-related traits, metabolites, and lipids, implemented by the QTL.gCIMapping (https://cran.r-project.org/web/packages/QTL.gCIMapping.GUI/index.html) and QTL IciMapping V4.1 (http://www.isbreeding.net) software packages. In the OC and RC joint analysis, RC and OC were viewed as covariate. The walk speed for genome-wide scanning was set at 1 cM, and the threshold for significant QTL was set as LOD ≥ 2.5 [51]. The trait, metabolite, and lipid data sets from all the lines were reanalyzed by multi-locus GWAS methods using the mrMLM v4.0.2 software [53] (https://cran.r-project.org/web/packages/mrMLM.GUI/index.html). In the software, there are six methods: mrMLM [97], ISIS EM-BLASSO [98], pKWmEB [99], pLARmEB [100], FASTmrMLM [52], and FASTmrEMMA [101]. The threshold for significant QTL was set as LOD ≥ 3.0 [97]. All the mQTLs were obtained from each biological replicate. QTL-by-environment interactions (QEIs) for oil-related traits were identified using the QTL IciMapping V4.1 software with ‘ICIM-EPI’ parameter, and the significant LOD thresholds for QEIs were set as 5.0 [51]. Here, stable QTLs were defined as those identified by at least two approaches and/or in at least two environments.

### Identification of candidate genes and target miRNAs and analysis of gene expression levels

Molecular markers in the overlapped region of QTLs were used to identify the genomic sequence by the assembly of soybean genome available at Soybase (release Wm82.a1.v1; https://www.soybase.org/). Known QTLs for oil-related traits were compared with metaQTLs identified in Qi et al. [102]. Candidate genes for each oil-related trait, metabolites, and lipids were mined according to the below rules: (i) genes were extracted between the 200 kb upstream and downstream regions for each significant QTL or QTL cluster [103]; (ii) candidate oil-related genes were chosen the genes specifically expressed in seed and differential expressed between high- and low-oil soybean accessions, and candidate metabolites/lipids genes were chosen the genes specifically expressed in seed and high expression genes at 55 DAF; (iii) based on the annotations from SFGD (http://bioinformatics.cau.edu.cn/SFGD/) and of Arabidopsis homologs from ARALIP (http://aralip.plantbiology.msu.edu/). Here candidate genes truly associated with traits or lipids/metabolites were defined as ones previously reported via their biological function identification.

Candidate miRNAs were extracted between the 200 kb upstream and downstream regions for each significant QTL or QTL cluster, according to soybean mature miRNA and miRNA hairpin sequences downloaded from miRBase (Release 22.1, http://www.mirbase.org/). Candidate miRNAs were further chosen the miRNAs targeted the candidate genes or any TFs. The reference annotations of miRNAs were transformed from Wm82.a2.v1 to Wm82.a1.v1 using Assembly Converter (http://plants.ensembl.org/Glycine_max/Tools/AssemblyConverter?db=core). The miRNA target predictions were conducted by psRNATarget [83] (http://plantgrn.noble.org/ psRNATarget), Target Finder [84], and psRobot [85] with default parameters. The sequencing data of small RNA for four CSSLs with high/low oil content were collected from Yu et al. [65].

Two RNA-seq data sets were used in this study. Seed-specific expressed genes were detected using data set I, downloaded from RNA Seq-Atlas in Soybase (https://www.soybase.org/soyseq), including young leaf, flower, pod, pod shell, root, nodule, and seed tissues [53]. DEGs were detected using data set II in Zhou et al. [54]. DEGs between high- (four domesticated soybeans: No. 101, 236, 257, and 276) and low-oil (two wild soybeans: No. 265 and 272) accessions [55] were detected using R with ‘DEGseq’ package at a 0.05 significant level [104]. Genes with at least a one-time expression level in 55 DAF than average expression level were viewed as high expression genes. Genes with FPKM value of expression level < 1 in all the tissues and with missing values exceeding 20% of the total number of samples were discarded. Two RNA-seq data sets were used to conduct co-expression analyses for candidate genes and gene pairs with correlation coefficient greater than 0.8 were retained.

### Construction and visualization of the GRN and MDGN

GRN was constructed by co-expression analysis, PPI, TFBS, and miRNA target predictions among candidate TFs, genes, and miRNAs. Significant PPIs were predicted (the predicted scores > 0.40) using STRING [105] (https://string-db.org/cgi/input.pl). Significant co-expression interactions (*r*_pcc_ > 0.8) were calculated at five stages during seed development using the data set of Zhou et al. [54]. TFBS predictions of candidate genes were conducted by FIMO software with the threshold of 1.0E−4 [82]. All the above relationships were used to construct MDGN, and this MDGN was classified as three layers (Fig. 1). In the first layer, the relationships of seed oil-related traits with metabolites/lipids were constructed using modern statistical methods. In the second layer, seed oil-related traits and metabolites/lipids were associated with SNP markers in the genome via QTL mapping approaches to identify QTLs and mQTLs, respectively. In third layer, all the TFs and miRNAs were targeted with all the candidate genes to construct the GRN. All the above relationships were integrated as one MDGN. In this MDGN, the circuit concept in graph theory was used to extract circular sub-networks. Regardless of the number of nodes, 3D sub-network was defined as ones that must contain one gene, one oil-related trait, and one metabolite/lipid, while 4D sub-network was defined as ones that must contain one additional miRNA as compared as 3D sub-network.

Network visualization was implemented with the Cytoscape package [106]. The network centrality parameters were detected using the Cytoscape plug-in NetworkAnalyzer. MCC scores were calculated by Cytoscape plug-in CytoHubba [107] and the top 10% nodes in the MCC score distributions were defined as hub nodes.

## Supplementary Information

**Additional file 1: Table S1.** Phenotypic characteristics for seed oil-related traits in 398 soybean RILs. **Table S2.** Phenotypic characteristics for 59 metabolites in 398 soybean RILs. **Table S3.** Phenotypic characteristics for 107 lipids in 398 soybean RILs. **Table S4.** Associations between oil-related traits and metabolites in 398 soybean RILs identified using the minimax concave penalty and smoothly clipped absolute deviation methods. **Table S5.** Associations between oil-related traits and lipids in 398 soybean RILs identified using the minimax concave penalty and smoothly clipped absolute deviation methods. **Table S6.** Associations between metabolites and metabolites, between metabolites and lipids, and between lipids and lipids in 398 soybean RILs identified using the Gaussian graphical model. **Table S8.** 175 QTLs for seed oil-related traits, their candidate genes, and miRNAs identified using multiple methods or across multiple environments. **Table S9.** 36 significant QTL-by-environment interactions for seed oil-related traits and their candidate genes. **Table S10.** miRNAs and their targeted acyl-lipid genes, predicted via psRNAtarget, Target Finder, and psRobot, around QTLs for seed oil-related traits. **Table S11.** Co-expression Pearson correlation coefficient among all the candidate genes in GRN. **Table S12.** Candidate genes for seed oil-related traits and their promoter sequences matched to motifs of miRNA-targeted TFs predicted via software FIMO. **Table S15.** 302 mQTL clusters for metabolites and lipids, their candidate genes, and miRNAs. **Table S16.** Co-located QTLs and their candidate genes for oil-related traits and metabolites/lipids. **Table S17** miRNAs and their targeted acyl-lipid genes, predicted via psRNAtarget, Target Finder, and psRobot, around mQTLs for metabolites and lipids. **Table S18.** Candidate genes for metabolites/lipids and their promoter sequences matched to motif of miRNA-targeted TFs predicted via software FIMO. **Table S19.** 147 significant PPIs among candidate genes for seed oil-related traits, metabolites, and lipids. **Table S20.** Topologic characteristics of nodes in multi-dimensional genetic networks. **Table S21.** 128 3D and 4D sub-networks for seed oil-related traits, metabolites, lipids, candidate genes, TFs, and miRNAs. **Table S22.** Common metabolites, candidate genes, trait–gene associations in this and previous studies [55].

**Additional file 2: Table S7.** Quantitative trait loci (QTLs) for seed oil-related traits in soybean seed using genome-wide composite interval mapping, inclusive composite interval mapping, and multi-locus GWAS methods.

**Additional file 3: Table S13.** mQTLs for metabolites in soybean seed using genome-wide composite interval mapping (GCIM), inclusive composite interval mapping (ICIM), and multi-locus GWAS.

**Additional file 4: Table S14.** mQTLs for lipids in soybean seed using genome-wide composite interval mapping (GCIM), inclusive composite interval mapping (ICIM), and multi-locus GWAS.

## Data Availability

The additional data generated during this study are available in the Additional files.

## Abbreviations

3D: Three dimension
4D: Four dimension
BLUP: Best linear unbiased prediction
CSSL: Chromosome segment substitution line
CV: Coefficients of variation
DAF: Days after flowering
DEG: Differentially expressed gene
GCIM: Genome-wide composite interval mapping
GGM: Gaussian graphical modeling
GRN: Gene regulatory network
ICIM: Inclusive composite interval mapping
MCP: Minimax concave penalty
MDGN: Multi-dimensional genetic network
PPI: Protein–protein interaction
QEI: QTL-by-environment interaction
QTL: Quantitative trait locus
mQTL: Metabolic QTL
mrMLM: Multi-locus random-SNP-effect mixed linear model
RIL: Recombinant inbred line
SCAD: Smoothly clipped absolute deviation penalty
TF: Transcription factor
TFBS: TF binding site
