# Supplementary material for: 4D genetic networks reveal the genetic basis of metabolites and seed oil-related traits in 398 soybean RILs
Source: Biotechnol Biofuels Bioprod. 2022 Sep 9;15:92. doi: 10.1186/s13068-022-02191-1 (PMC9461130; doi:10.1186/s13068-022-02191-1)
Supplement: Supplementary file 1 — Additional file 1: Table S1. Phenotypic characteristics for seed oil-related traits in 398 soybean RILs. Table S2. Phenotypic characteristics for 59 metabolites in 398 soybean RILs. Table S3. Phenotypic characteristics for 107 lipids in 398 soybean RILs. Table S4. Associations between oil-related traits and metabolites in 398 soybean RILs identified using the minimax concave penalty and smoothly clipped absolute deviation methods. Table S5. Associations between oil-related traits and lipids in 398 soybean RILs identified using the minimax concave penalty and smoothly clipped absolute deviation methods. Table S6. Associations between metabolites and metabolites, between metabolites and lipids, and between lipids and lipids in 398 soybean RILs identified using the Gaussian graphical model. Table S8. 175 QTLs for seed oil-related traits, their candidate genes, and miRNAs identified using multiple methods or across multiple environments. Table S9. 36 significant QTL-by-environment interactions for seed oil-related traits and their candidate genes. Table S10. miRNAs and their targeted acyl-lipid genes, predicted via psRNAtarget, Target Finder, and psRobot, around QTLs for seed oil-related traits. Table S11. Co-expression Pearson correlation coefficient among all the candidate genes in GRN. Table S12. Candidate genes for seed oil-related traits and their promoter sequences matched to motifs of miRNA-targeted TFs predicted via software FIMO. Table S15. 302 mQTL clusters for metabolites and lipids, their candidate genes, and miRNAs. Table S16. Co-located QTLs and their candidate genes for oil-related traits and metabolites/lipids. Table S17 miRNAs and their targeted acyl-lipid genes, predicted via psRNAtarget, Target Finder, and psRobot, around mQTLs for metabolites and lipids. Table S18. Candidate genes for metabolites/lipids and their promoter sequences matched to motif of miRNA-targeted TFs predicted via software FIMO. Table S19. 147 significant PPIs among candidate gene [file 13068_2022_2191_MOESM1_ESM.docx]

**Additional file 1**

**Table S1** Phenotypic characteristics for seed oil related traits in 398 soybean RILs

| **Traits (%)** | **Environment ^a^** | **Mean** | **Std Dev** | **Minimum** | **Maximum** | **CV ^b^ (%)** | **Skewness** | **Kurtosis** |
| --- | --- | --- | --- | --- | --- | --- | --- | --- |
| Stearic acid | WH2014 | 11.7841 | 0.5580 | 9.9250 | 13.5900 | 4.7435 | -0.0413 | 0.0084 |
|  | EZ2015 | 11.3537 | 0.5275 | 10.0360 | 12.9090 | 4.6525 | 0.0993 | -0.3019 |
|  | NJ2015 | 11.9319 | 0.8120 | 8.6179 | 14.8356 | 6.8142 | 0.4370 | 1.1477 |
|  | BLUP | 11.6896 | 0.3984 | 10.6416 | 13.1326 | 0.0341 | 0.2156 | 0.0966 |
| Palmitic acid | WH2014 | 3.8134 | 0.2653 | 3.1630 | 4.7450 | 6.9695 | -0.0127 | -0.0175 |
|  | EZ2015 | 3.2078 | 0.4214 | 1.9240 | 4.1950 | 13.1568 | -0.9365 | 0.4553 |
|  | NJ2015 | 3.5894 | 0.3030 | 2.1718 | 4.7552 | 8.4528 | -0.7670 | 5.2820 |
|  | BLUP | 3.5693 | 0.1289 | 3.3303 | 5.2011 | 0.0361 | 9.5946 | 117.7507 |
| Oleic acid | WH2014 | 25.9470 | 3.8816 | 17.6650 | 40.7450 | 14.9869 | 0.8305 | 0.9271 |
|  | EZ2015 | 25.4789 | 4.1961 | 17.0790 | 39.3250 | 16.4920 | 0.5891 | -0.0811 |
|  | NJ2015 | 26.3309 | 4.1467 | 14.5010 | 41.1424 | 14.3469 | -0.5265 | 6.0839 |
|  | BLUP | 25.9676 | 2.6092 | 16.7390 | 35.1944 | 0.1005 | 0.3988 | 0.5063 |
| Linoleic acid | WH2014 | 51.5589 | 3.1339 | 40.6400 | 57.5820 | 6.0893 | -0.7938 | 0.7536 |
|  | EZ2015 | 52.9927 | 3.4658 | 41.8360 | 61.8570 | 6.5492 | -0.3769 | 0.0592 |
|  | NJ2015 | 51.3981 | 3.1272 | 39.8677 | 67.7668 | 6.0923 | -0.2270 | 2.2692 |
|  | BLUP | 51.9697 | 2.0345 | 45.1902 | 58.5099 | 0.0391 | -0.4060 | 0.1784 |
| Linolenic acid | WH2014 | 6.8966 | 0.6610 | 4.9500 | 9.1560 | 9.6012 | -0.0253 | 0.1290 |
|  | EZ2015 | 6.9669 | 0.7723 | 4.9360 | 9.1800 | 11.1008 | 0.0932 | 0.0173 |
|  | NJ2015 | 6.9421 | 0.9228 | 3.1945 | 9.1143 | 11.7340 | -2.2539 | 15.7271 |
|  | BLUP | 6.9346 | 0.5121 | 4.6172 | 8.3995 | 0.0738 | -0.3777 | 1.4304 |
| Oil content | WH2014 | 18.0442 | 1.3277 | 13.3400 | 22.3290 | 7.3714 | 0.0541 | 0.4570 |
|  | NJ2015 | 19.2756 | 1.3437 | 14.4310 | 27.8490 | 6.9805 | 0.8304 | 5.0334 |
|  | BLUP | 18.6689 | 0.5648 | 16.9122 | 21.1096 | 0.0303 | 0.3222 | 0.8942 |

^a^ BLUP: best linear unbiased prediction; EZ: Ezhou; NJ: Nanjing; WH: Wuhan. ^b^ CV: coefficient of variation.

**Table S2** Phenotypic characteristics for 59 metabolites in 398 soybean RILs

| **Metabolite name** | **Class** | **KEGG ID ^a^** | **PubChem CID ^b^** | **Sub-class** | **Mean** | **Std Dev^c^** | **Minimum** | **Maximum** | **Skewness** | **Kurtosis** | **Coefficient of variation (%)** |
| --- | --- | --- | --- | --- | --- | --- | --- | --- | --- | --- | --- |
| D-fructose 2,6-bisphosphate | Carbohydrates | C00665 | 105021 | primary metabolites | 2.0166E-04 | 2.3572E-04 | 3.1000E-07 | 1.1387E-03 | 1.3241 | 1.1594 | 75.41 |
| fucose | Carbohydrates | C01019 | 17106 | primary metabolites | 4.4579E-04 | 3.1940E-04 | 2.9100E-07 | 1.9299E-03 | 0.7477 | 0.4630 | 55.34 |
| D-glucose | Carbohydrates | C00031 | 5793 | primary metabolites | 8.6875E-05 | 1.0494E-04 | 2.7500E-07 | 9.3456E-04 | 2.5002 | 10.9761 | 52.27 |
| glycerol | Carbohydrates | C00116 | 753 | primary metabolites | 1.5941E-03 | 1.3480E-03 | 2.7800E-07 | 9.4180E-03 | 1.5945 | 3.2339 | 102.63 |
| mannose | Carbohydrates | C00159 | 18950 | primary metabolites | 1.7492E-04 | 1.4883E-04 | 2.7300E-07 | 1.1619E-03 | 1.8809 | 6.4669 | 59.55 |
| inositol | Carbohydrates | C00137 | 892 | primary metabolites | 3.2518E-04 | 4.2279E-04 | 2.6800E-07 | 3.5314E-03 | 2.6854 | 10.5992 | 84.46 |
| sucrose | Carbohydrates | C00089 | 5988 | primary metabolites | 3.3148E-04 | 4.8913E-04 | 2.7300E-07 | 5.1881E-03 | 4.0495 | 25.4619 | 118.72 |
| gycerol 1-phosphate | Carbohydrates | C00093 | 439162 | primary metabolites | 8.2018E-04 | 6.4412E-04 | 2.7300E-07 | 3.6273E-03 | 0.8735 | 0.8590 | 119.62 |
| 1-hexadecanol | Lipids | C00823 | 2682 | primary metabolites | 1.5771E-02 | 1.3320E-02 | 3.4000E-07 | 9.4090E-02 | 0.5709 | 0.7579 | 84.56 |
| 2-hydroxybutanoic acid | Lipids | C05984 | 11266 | primary metabolites | 1.4003E-04 | 1.4959E-04 | 2.8600E-07 | 1.0982E-03 | 2.5451 | 9.7019 | 89.60 |
| arachidic acid | Lipids | C06425 | 10467 | primary metabolites | 3.1239E-04 | 2.0722E-04 | 3.3200E-07 | 1.0916E-03 | 0.5554 | 0.1030 | 205.58 |
| capric acid | Lipids | C01571 | 2969 | primary metabolites | 8.6424E-04 | 5.1047E-04 | 2.8300E-07 | 3.9819E-03 | 1.2264 | 2.7222 | 216.29 |
| octanoic acid | Lipids | C06423 | 379 | primary metabolites | 6.4987E-04 | 7.8762E-04 | 2.8300E-07 | 5.4359E-03 | 2.0056 | 6.2247 | 61.87 |
| sphingosine | Lipids | C00319 | 5280335 | primary metabolites | 1.2611E-03 | 1.1623E-03 | 2.9200E-07 | 6.8683E-03 | 1.1970 | 1.8477 | 86.25 |
| 2-aminooctadecane-1,3-diol | Lipids | C00836 | 3126 | primary metabolites | 1.6358E-04 | 1.7537E-04 | 2.8400E-07 | 9.4499E-04 | 1.6141 | 2.2184 | 106.27 |
| lauric acid | Lipids | C02679 | 3893 | primary metabolites | 8.0452E-05 | 9.5285E-05 | 2.6800E-07 | 1.0601E-03 | 4.1199 | 32.9775 | 94.37 |
| linoleic acid | Lipids | C01595 | 5280450 | primary metabolites | 3.4851E-03 | 1.7294E-03 | 3.1600E-07 | 1.2783E-02 | 0.4407 | 0.9695 | 96.98 |
| linolenic acid | Lipids | C06427 | 5280934 | primary metabolites | 2.0741E-04 | 2.2367E-04 | 2.7900E-07 | 1.3370E-03 | 2.0682 | 4.4310 | 47.14 |
| methyl jasmonate | Lipids | C11512 | 5281929 | secondary metabolites | 7.2623E-05 | 8.5422E-05 | 2.8400E-07 | 5.9188E-04 | 1.5073 | 3.4351 | 126.66 |
| myristic acid | Lipids | C06424 | 11005 | primary metabolites | 1.4564E-03 | 6.8649E-04 | 2.9400E-07 | 5.0294E-03 | 0.6059 | 1.9632 | 111.27 |
| oleic acid | Lipids | C00712 | 445639 | primary metabolites | 2.5739E-03 | 3.0375E-03 | 2.9600E-07 | 1.4201E-02 | 0.9922 | -0.2178 | 63.57 |
| palmitic acid | Lipids | C00249 | 985 | primary metabolites | 6.8264E-02 | 3.3476E-02 | 5.1600E-07 | 1.9378E-01 | 0.9581 | 0.7600 | 118.01 |
| palmitoleic acid | Lipids | C08362 | 445638 | primary metabolites | 3.5473E-04 | 2.4561E-04 | 2.6800E-07 | 1.4400E-03 | 0.7527 | 0.3958 | 87.42 |
| prostaglandin A | Lipids | C05953 | 5280880 | secondary metabolites | 3.9953E-05 | 6.1747E-05 | 2.7400E-07 | 4.2996E-04 | 1.9664 | 4.8059 | 76.70 |
| stearic acid | Lipids | C01530 | 5281 | primary metabolites | 7.1377E-02 | 4.9960E-02 | 3.3200E-07 | 2.2345E-01 | 0.2319 | -0.5795 | 116.44 |
| 2-oxoadipate | Organic acids | C00322 | 71 | primary metabolites | 7.4277E-04 | 3.8827E-04 | 3.5300E-07 | 2.9086E-03 | 0.7811 | 1.9895 | 70.15 |
| 2-oxobutyric acid | Organic acids | C00109 | 58 | primary metabolites | 1.3022E-04 | 1.5148E-04 | 2.9400E-07 | 1.1173E-03 | 2.7685 | 9.8031 | 79.75 |
| 3-methyl-2-oxobutanoic acid | Organic acids | C00141 | 5204641 | primary metabolites | 3.6720E-04 | 3.1052E-04 | 2.9400E-07 | 2.0950E-03 | 1.5471 | 3.6515 | 128.71 |
| 3-hydroxypropionic acid | Organic acids | C01013 | 68152 | primary metabolites | 1.2799E-04 | 9.3538E-05 | 2.7500E-07 | 6.0298E-04 | 1.3412 | 2.3839 | 82.05 |
| alpha-ketoisocaproic acid | Organic acids | C00233 | 70 | secondary metabolites | 2.0068E-04 | 1.6261E-04 | 2.8800E-07 | 1.1249E-03 | 1.1805 | 3.5436 | 84.26 |
| citraconic acid | Organic acids | C02226 | 643798 | primary metabolites | 5.0317E-05 | 5.6999E-05 | 2.7300E-07 | 2.5742E-04 | 1.0642 | 0.5914 | 140.50 |
| citric acid | Organic acids | C00158 | 19782904 | primary metabolites | 3.2920E-04 | 3.1927E-04 | 2.7500E-07 | 1.9430E-03 | 1.4768 | 2.3528 | 127.02 |
| D-glyceric acid | Organic acids | C00258 | 439194 | primary metabolites | 1.2247E-04 | 1.1184E-04 | 2.8300E-07 | 1.1121E-03 | 3.3948 | 20.6848 | 71.56 |
| fumaric acid | Organic acids | C00122 | 21883788 | primary metabolites | 2.0533E-04 | 5.2801E-04 | 2.7400E-07 | 5.3103E-03 | 4.2537 | 22.4595 | 65.62 |
| glucose-1-phosphate | Organic acids | C00103 | 65533 | primary metabolites | 3.4376E-04 | 7.6115E-04 | 2.7300E-07 | 6.7316E-03 | 2.9941 | 10.7958 | 97.30 |
| glucose-6-phosphate | Organic acids | C00092 | 5958 | primary metabolites | 9.2447E-05 | 1.1443E-04 | 2.7400E-07 | 6.9788E-04 | 1.9617 | 4.6733 | 66.79 |
| isocitric acid | Organic acids | C00311 | 1198 | primary metabolites | 1.1602E-04 | 9.2345E-05 | 2.7300E-07 | 5.6898E-04 | 1.1852 | 1.9155 | 65.46 |
| L-malic acid | Organic acids | C00149 | 222656 | primary metabolites | 2.7707E-04 | 2.6878E-04 | 2.7800E-07 | 2.3834E-03 | 2.6792 | 11.4015 | 71.27 |
| malonic acid | Organic acids | C00383 | 23511544 | primary metabolites | 2.6119E-04 | 2.2348E-04 | 2.7800E-07 | 1.1918E-03 | 1.2764 | 1.4526 | 85.08 |
| oxalacetic acid | Organic acids | C00036 | 970 | primary metabolites | 1.8973E-03 | 4.1037E-03 | 2.7400E-07 | 3.7536E-02 | 3.3942 | 14.8040 | 187.91 |
| oxalic acid | Organic acids | C00209 | 18676629 | primary metabolites | 5.4463E-04 | 6.2018E-04 | 2.7400E-07 | 3.9191E-03 | 1.5413 | 2.6878 | 90.63 |
| pyruvate | Organic acids | C00022 | 1060 | primary metabolites | 5.2213E-03 | 5.7235E-03 | 3.5100E-07 | 2.3576E-02 | 0.9613 | 0.0128 | 113.08 |
| succinic acid | Organic acids | C00042 | 1110 | primary metabolites | 2.2771E-04 | 2.0695E-04 | 2.8300E-07 | 1.6787E-03 | 1.8882 | 5.4209 | 54.48 |
| threonate | Organic acids | C01620 | 151152 | primary metabolites | 1.1291E-03 | 7.7591E-04 | 2.6800E-07 | 4.5320E-03 | 0.7638 | 0.3976 | 54.96 |
| 4-aminobutyric acid (GABA) | Amino Acids | C00334 | 119 | primary metabolites | 1.2304E-04 | 9.5893E-05 | 2.7400E-07 | 8.0142E-04 | 1.6286 | 5.4412 | 79.59 |
| alanine | Amino Acids | C00041 | 5950 | primary metabolites | 6.7454E-05 | 7.8546E-05 | 2.7500E-07 | 4.4012E-04 | 1.2810 | 1.6332 | 147.02 |
| beta-Alanine | Amino Acids | C00099 | 239 | primary metabolites | 1.4779E-03 | 8.4495E-04 | 2.7300E-07 | 4.8120E-03 | 0.7725 | 0.3858 | 78.42 |
| citrulline | Amino Acids | C00327 | 6992098 | primary metabolites | 5.6857E-04 | 3.7221E-04 | 2.9400E-07 | 2.1902E-03 | 0.9778 | 1.0881 | 129.55 |
| ethanolamine | Amino Acids | C00189 | 700 | primary metabolites | 1.2743E-04 | 1.6186E-04 | 2.7300E-07 | 9.9765E-04 | 1.6193 | 2.9464 | 70.53 |
| glutamine | Amino Acids | C00064 | 5961 | primary metabolites | 7.2366E-04 | 1.8418E-03 | 3.0300E-07 | 1.0563E-02 | 3.1003 | 8.9594 | 94.59 |
| glutathione (GSH) | Amino Acids | C00051 | 124886 | primary metabolites | 9.8298E-03 | 5.9711E-03 | 3.5900E-07 | 3.1380E-02 | 0.7944 | 0.4738 | 61.23 |
| isoleucine | Amino Acids | C00407 | 6306 | primary metabolites | 6.4576E-04 | 7.5530E-04 | 2.7400E-07 | 4.9012E-03 | 1.1946 | 1.2761 | 65.05 |
| leucine | Amino Acids | C00123 | 6106 | primary metabolites | 4.0572E-03 | 1.9891E-03 | 3.3600E-07 | 1.3235E-02 | 0.4885 | 0.5240 | 62.09 |
| L-homoserine | Amino Acids | C00263 | 12647 | primary metabolites | 3.9651E-04 | 1.9663E-04 | 2.7500E-07 | 1.0851E-03 | 0.0965 | 0.1711 | 156.36 |
| oxoproline | Amino Acids | C01879 | 7405 | secondary metabolites | 4.9437E-04 | 5.2224E-04 | 3.0000E-07 | 3.0867E-03 | 1.3099 | 1.5486 | 58.63 |
| proline | Amino Acids | C00148 | 145742 | primary metabolites | 1.5779E-04 | 1.3651E-04 | 2.7800E-07 | 1.0255E-03 | 1.2860 | 2.9236 | 117.15 |
| putrescine | Amino Acids | C00134 | 1045 | primary metabolites | 1.3532E-03 | 1.2274E-03 | 2.6800E-07 | 7.6545E-03 | 1.3579 | 2.7231 | 54.34 |
| serine | Amino Acids | C00065 | 5951 | primary metabolites | 1.5626E-03 | 1.0769E-03 | 2.9100E-07 | 5.7842E-03 | 0.7847 | 0.7052 | 85.02 |
| threonine | Amino Acids | C00188 | 6288 | primary metabolites | 7.2284E-04 | 1.0557E-03 | 2.7300E-07 | 5.8730E-03 | 1.6065 | 1.7173 | 103.84 |

^a^ KEGG COMPOUND database: https://www.kegg.jp/kegg/compound/. ^b^ PubChem open chemistry database: https://pubchem.ncbi.nlm.nih.gov/. ^c^ Std Dev: standard deviation.

**Table S3** Phenotypic characteristics for 107 lipids in 398 soybean RILs

| **Name** | **Sub class** | **KEGG ID^a^** | **Lipid category^b^** | **Mean** | **Std Dev^c^** | **Minimum** | **Maximum** | **Skewness** | **Kurtosis** | **Coefficient of**  **variation (%)** |
| --- | --- | --- | --- | --- | --- | --- | --- | --- | --- | --- |
| FA(16:0) | FA | C00162 | Fatty acid | 2.9742E-02 | 3.1614E-02 | 5.7000E-05 | 2.3640E-01 | 2.2732 | 6.6069 | 106.29 |
| FA(18:0) | FA | C00162 | Fatty acid | 2.9656E-02 | 3.1121E-02 | 5.4700E-05 | 1.6282E-01 | 1.9868 | 3.7588 | 104.94 |
| FA(18:1) | FA | C00162 | Fatty acid | 2.4830E-02 | 2.8851E-02 | 5.6700E-05 | 2.2330E-01 | 2.6875 | 9.4447 | 116.19 |
| FA(18:2) | FA | C00162 | Fatty acid | 2.1952E-02 | 2.6144E-02 | 1.2555E-05 | 1.4778E-01 | 2.1872 | 5.3399 | 119.10 |
| FA(18:3) | FA | C00162 | Fatty acid | 1.2749E-02 | 1.7544E-02 | 4.9200E-06 | 1.5214E-01 | 3.2284 | 14.2287 | 137.61 |
| FA(20:0) | FA | C00162 | Fatty acid | 1.1472E-03 | 1.2902E-03 | 2.5000E-06 | 9.6467E-03 | 2.4886 | 8.1099 | 112.47 |
| FA(20:1) | FA | C00162 | Fatty acid | 3.5594E-04 | 4.1384E-04 | 7.1900E-07 | 2.5863E-03 | 2.4381 | 6.9342 | 116.27 |
| FA(20:2) | FA | C00162 | Fatty acid | 4.4473E-05 | 5.3130E-05 | 2.0000E-10 | 3.7005E-04 | 2.4360 | 7.2773 | 119.47 |
| FA(22:1) | FA | C00162 | Fatty acid | 4.0378E-05 | 6.6739E-05 | 7.0000E-10 | 4.6831E-04 | 3.0905 | 11.0615 | 165.28 |
| OAHFA(18:1/18:0) | OAHFA | C03547 | Fatty acid | 1.0439E-03 | 1.1741E-03 | 4.2000E-09 | 8.6366E-03 | 2.5564 | 8.4255 | 112.48 |
| DG(16:0/16:0) | DG | C00165 | Glycerolipid | 1.6733E-04 | 1.5389E-04 | 1.8200E-05 | 2.5971E-03 | 7.2439 | 99.1978 | 91.97 |
| DG(16:0/18:1) | DG | C00165 | Glycerolipid | 1.5318E-04 | 1.0416E-04 | 1.8000E-05 | 7.0501E-04 | 1.6085 | 3.2265 | 68.00 |
| DG(16:0/18:2) | DG | C00165 | Glycerolipid | 4.6939E-04 | 2.8190E-04 | 5.5100E-05 | 1.8475E-03 | 1.4014 | 2.4952 | 60.06 |
| DG(16:0/18:3) | DG | C00165 | Glycerolipid | 3.9982E-04 | 4.2661E-04 | 1.7300E-05 | 2.9652E-03 | 2.3200 | 6.3010 | 106.70 |
| DG(18:0/16:0) | DG | C00165 | Glycerolipid | 2.3329E-04 | 2.7194E-04 | 1.7200E-05 | 5.2155E-03 | 10.9515 | 180.9145 | 116.56 |
| DG(18:0/18:0) | DG | C00165 | Glycerolipid | 1.3355E-04 | 1.2903E-04 | 1.3900E-05 | 2.3836E-03 | 9.0122 | 139.6797 | 96.62 |
| DG(18:0/18:1) | DG | C00165 | Glycerolipid | 2.4255E-03 | 1.9040E-03 | 1.2521E-04 | 1.1783E-02 | 1.8063 | 4.3544 | 78.50 |
| DG(18:1/18:1) | DG | C00165 | Glycerolipid | 2.9690E-04 | 2.5807E-04 | 1.4500E-05 | 1.4430E-03 | 1.6251 | 3.0612 | 86.92 |
| DG(18:1/18:2) | DG | C00165 | Glycerolipid | 7.9929E-04 | 4.3607E-04 | 1.3848E-04 | 2.9372E-03 | 1.3605 | 2.1706 | 54.56 |
| DG(18:2/18:2) | DG | C00165 | Glycerolipid | 1.5628E-03 | 1.0091E-03 | 2.6676E-04 | 6.3140E-03 | 1.5801 | 3.0181 | 64.57 |
| DG(18:3/18:2) | DG | C00165 | Glycerolipid | 5.5504E-04 | 3.5441E-04 | 6.3400E-06 | 2.1964E-03 | 1.4567 | 2.7144 | 63.85 |
| DG(18:3/18:3) | DG | C00165 | Glycerolipid | 2.6690E-04 | 1.5718E-04 | 6.9400E-06 | 1.0914E-03 | 1.3701 | 2.5879 | 58.89 |
| DG(20:0/18:2) | DG | C00165 | Glycerolipid | 2.3831E-04 | 1.7011E-04 | 1.5400E-05 | 1.2564E-03 | 1.7160 | 4.3517 | 71.38 |
| DG(20:0/18:3) | DG | C00165 | Glycerolipid | 1.8273E-04 | 1.3168E-04 | 9.7000E-06 | 1.2479E-03 | 2.1900 | 8.9601 | 72.07 |
| DG(20:1/18:2) | DG | C00165 | Glycerolipid | 3.9355E-04 | 2.2525E-04 | 6.9000E-05 | 1.4526E-03 | 1.3635 | 2.1656 | 57.23 |
| DG(20:1/18:3) | DG | C00165 | Glycerolipid | 2.5158E-04 | 1.5528E-04 | 4.1600E-05 | 1.0654E-03 | 1.6165 | 3.4382 | 61.72 |
| DGDG(16:0/18:2) | DGDG | C06037 | Glycerolipid | 7.2337E-04 | 8.9325E-04 | 9.5200E-07 | 7.1095E-03 | 3.4585 | 15.7223 | 123.48 |
| DGDG(16:0/18:3) | DGDG | C06037 | Glycerolipid | 4.7289E-04 | 4.7179E-04 | 3.0000E-10 | 2.5761E-03 | 1.7514 | 3.1609 | 99.77 |
| DGDG(18:3/18:3) | DGDG | C06037 | Glycerolipid | 1.1052E-03 | 1.1587E-03 | 3.0000E-10 | 5.8827E-03 | 1.8948 | 3.7381 | 104.83 |
| TG(16:0/16:0/18:1) | TG | C00422 | Glycerolipid | 3.2649E-04 | 1.6463E-04 | 6.4000E-05 | 1.0070E-03 | 1.1107 | 1.1540 | 50.42 |
| TG(16:0/16:0/18:2) | TG | C00422 | Glycerolipid | 4.6879E-04 | 1.7716E-04 | 1.2276E-04 | 1.5343E-03 | 1.4108 | 3.3499 | 37.79 |
| TG(16:0/16:0/18:3) | TG | C00422 | Glycerolipid | 1.1233E-04 | 5.5743E-05 | 2.4300E-05 | 4.0952E-04 | 1.5817 | 3.7192 | 49.62 |
| TG(16:0/18:1/18:1) | TG | C00422 | Glycerolipid | 3.7139E-03 | 2.7360E-03 | 3.8003E-04 | 3.4364E-02 | 3.3014 | 23.4902 | 73.67 |
| TG(16:0/18:1/18:2) | TG | C00422 | Glycerolipid | 1.2589E-05 | 6.4739E-06 | 6.5100E-07 | 4.2500E-05 | 0.9990 | 1.1807 | 51.42 |
| TG(16:0/18:1/18:3) | TG | C00422 | Glycerolipid | 3.1419E-05 | 1.8167E-05 | 2.5600E-06 | 1.0362E-04 | 1.0554 | 1.0693 | 57.82 |
| TG(16:0/18:2/18:2) | TG | C00422 | Glycerolipid | 1.2675E-02 | 6.4414E-03 | 2.0453E-04 | 4.3860E-02 | -0.0296 | 0.7307 | 50.82 |
| TG(16:0/18:2/18:3) | TG | C00422 | Glycerolipid | 4.5638E-03 | 2.3206E-03 | 1.1297E-03 | 2.0138E-02 | 1.8895 | 5.7825 | 50.85 |
| TG(16:0/18:3/18:3) | TG | C00422 | Glycerolipid | 8.3713E-04 | 4.5222E-04 | 1.6277E-04 | 4.0736E-03 | 2.2472 | 8.1667 | 54.02 |
| TG(18:0/16:0/18:1) | TG | C00422 | Glycerolipid | 6.6605E-04 | 2.9428E-04 | 1.2467E-04 | 1.9089E-03 | 0.9027 | 0.9198 | 44.18 |
| TG(18:0/18:0/18:1) | TG | C00422 | Glycerolipid | 3.6384E-04 | 1.9226E-04 | 4.6000E-05 | 1.2610E-03 | 1.0656 | 1.3692 | 52.84 |
| TG(18:0/18:1/18:1) | TG | C00422 | Glycerolipid | 3.3202E-03 | 2.1529E-03 | 3.4277E-04 | 1.6643E-02 | 1.4583 | 3.5699 | 64.84 |
| TG(18:1/18:1/18:1) | TG | C00422 | Glycerolipid | 8.7062E-03 | 6.8667E-03 | 5.1028E-04 | 8.3656E-02 | 3.3360 | 21.8179 | 78.87 |
| TG(18:1/18:1/18:2) | TG | C00422 | Glycerolipid | 3.2362E-05 | 2.2241E-05 | 3.6030E-06 | 1.6108E-04 | 1.9669 | 5.4007 | 68.72 |
| TG(18:1/18:1/18:3) | TG | C00422 | Glycerolipid | 1.1354E-04 | 7.0235E-05 | 1.5600E-05 | 7.2051E-04 | 2.5966 | 13.0441 | 61.86 |
| TG(18:1/18:2/18:2) | TG | C00422 | Glycerolipid | 5.9521E-02 | 1.5818E-02 | 1.2751E-02 | 1.1034E-01 | 0.1948 | 0.0484 | 26.57 |
| TG(18:1/18:2/18:3) | TG | C00422 | Glycerolipid | 1.6125E-01 | 3.8739E-02 | 4.2290E-02 | 2.8340E-01 | -0.3624 | -0.1895 | 24.02 |
| TG(18:2/18:2/18:2) | TG | C00422 | Glycerolipid | 1.2375E-05 | 4.6619E-06 | 3.6750E-06 | 3.0400E-05 | 0.2588 | -0.2356 | 37.67 |
| TG(18:3/18:2/18:2) | TG | C00422 | Glycerolipid | 1.2264E-05 | 3.8540E-06 | 3.3200E-06 | 2.5200E-05 | 0.6987 | 0.0858 | 31.42 |
| TG(18:3/18:2/18:3) | TG | C00422 | Glycerolipid | 1.5052E-05 | 8.9760E-06 | 2.3300E-06 | 7.1400E-05 | 1.6259 | 4.2404 | 59.64 |
| TG(18:3/18:3/18:3) | TG | C00422 | Glycerolipid | 2.0976E-03 | 2.4559E-03 | 1.7386E-04 | 2.6676E-02 | 4.1504 | 25.0865 | 117.08 |
| TG(20:0/18:1/18:1) | TG | C00422 | Glycerolipid | 3.9016E-04 | 1.4724E-04 | 6.2400E-05 | 9.5598E-04 | 0.4667 | 0.3121 | 37.74 |
| TG(20:0/18:1/18:2) | TG | C00422 | Glycerolipid | 2.2463E-04 | 7.9410E-05 | 3.4600E-05 | 6.3953E-04 | 0.5273 | 1.0299 | 35.35 |
| TG(20:1/18:1/18:2) | TG | C00422 | Glycerolipid | 7.6275E-05 | 2.8016E-05 | 2.2200E-05 | 1.8332E-04 | 0.8638 | 0.6996 | 36.73 |
| TG(20:1/18:2/18:2) | TG | C00422 | Glycerolipid | 5.1620E-04 | 3.7370E-04 | 2.1000E-05 | 2.5565E-03 | 2.1319 | 5.9878 | 72.39 |
| TG(20:1/18:2/18:3) | TG | C00422 | Glycerolipid | 1.4951E-05 | 5.3450E-06 | 4.8000E-06 | 4.3000E-05 | 1.8527 | 5.4227 | 35.75 |
| TG(20:1/18:3/18:3) | TG | C00422 | Glycerolipid | 3.5809E-03 | 3.9491E-03 | 1.6685E-04 | 2.3404E-02 | 2.1631 | 5.0967 | 110.28 |
| TG(20:2/18:2/18:2) | TG | C00422 | Glycerolipid | 1.9912E-03 | 2.0718E-03 | 2.2650E-04 | 1.3099E-02 | 2.2039 | 5.0206 | 104.05 |
| SQDG(16:0/18:1) | SQDG | C13508 | Glycerolipid | 1.0249E-03 | 1.0930E-03 | 1.2200E-06 | 7.0116E-03 | 2.1376 | 5.1021 | 106.65 |
| SQDG(16:0/18:2) | SQDG | C13508 | Glycerolipid | 7.1227E-04 | 7.1839E-04 | 8.7900E-07 | 3.7944E-03 | 1.8649 | 3.5160 | 100.86 |
| SQDG(16:0/18:3) | SQDG | C13508 | Glycerolipid | 5.6432E-04 | 4.9624E-04 | 9.3100E-07 | 3.5015E-03 | 1.9110 | 4.8436 | 87.94 |
| CL(18:2/18:1/18:1/18:2) | CL | C05980 | Glycerolphospholipid | 4.3571E-04 | 4.5486E-04 | 4.2800E-07 | 4.2009E-03 | 2.9895 | 14.9863 | 104.39 |
| CL(18:2/18:2/18:2/18:1) | CL | C05980 | Glycerolphospholipid | 6.4865E-04 | 6.1405E-04 | 8.4000E-07 | 4.1637E-03 | 1.9789 | 5.0907 | 94.67 |
| CL(18:2/18:2/18:2/18:2) | CL | C05980 | Glycerolphospholipid | 7.9775E-04 | 7.5738E-04 | 1.2680E-06 | 4.9657E-03 | 1.8338 | 3.7692 | 94.94 |
| CL(18:3/18:2/18:2/18:2) | CL | C05980 | Glycerolphospholipid | 4.1938E-04 | 4.5286E-04 | 2.0000E-10 | 2.9515E-03 | 2.1836 | 5.4469 | 107.98 |
| LPC(16:0) | LPC | C04230 | Glycerolphospholipid | 1.8697E-05 | 1.0434E-05 | 3.7000E-06 | 6.5900E-05 | 1.3965 | 2.3011 | 55.80 |
| LPC(18:0) | LPC | C04230 | Glycerolphospholipid | 7.1093E-06 | 3.6651E-06 | 1.2600E-06 | 2.5900E-05 | 1.2157 | 1.8409 | 51.55 |
| LPC(18:1) | LPC | C04230 | Glycerolphospholipid | 2.9189E-05 | 1.8618E-05 | 2.0900E-06 | 1.1797E-04 | 1.5816 | 2.9045 | 63.78 |
| LPC(18:2) | LPC | C04230 | Glycerolphospholipid | 5.3186E-06 | 5.5303E-06 | 3.0000E-10 | 5.0700E-05 | 2.8647 | 12.7502 | 103.98 |
| LPE(16:0) | LPE | C04438 | Glycerolphospholipid | 1.0607E-03 | 1.1966E-03 | 1.1800E-06 | 8.7072E-03 | 2.6200 | 9.1317 | 112.82 |
| LPE(18:1) | LPE | C04438 | Glycerolphospholipid | 6.7498E-04 | 8.0571E-04 | 1.3700E-06 | 6.1687E-03 | 2.9024 | 11.3213 | 119.37 |
| LPE(18:2) | LPE | C04438 | Glycerolphospholipid | 5.6588E-04 | 6.0516E-04 | 6.5000E-09 | 4.3621E-03 | 2.0887 | 5.4271 | 106.94 |
| PC(16:0/18:1) | PC | C00157 | Glycerolphospholipid | 3.2021E-02 | 2.6378E-02 | 2.2500E-05 | 1.6332E-01 | 1.0957 | 1.2947 | 82.38 |
| PC(16:0/18:2) | PC | C00157 | Glycerolphospholipid | 2.9096E-02 | 2.6697E-02 | 8.1500E-05 | 2.6443E-01 | 2.6991 | 12.7543 | 91.76 |
| PC(16:0/18:3) | PC | C00157 | Glycerolphospholipid | 3.4409E-03 | 2.8769E-03 | 2.8000E-09 | 3.6861E-02 | 3.3203 | 28.3990 | 83.61 |
| PC(18:0/18:1) | PC | C00157 | Glycerolphospholipid | 1.7046E-02 | 1.6813E-02 | 3.7665E-05 | 1.2318E-01 | 1.8408 | 4.8273 | 98.63 |
| PC(18:1/18:1) | PC | C00157 | Glycerolphospholipid | 4.8753E-02 | 4.4478E-02 | 3.3776E-04 | 3.7467E-01 | 2.0034 | 6.5367 | 91.23 |
| PC(18:1/18:2) | PC | C00157 | Glycerolphospholipid | 2.8377E-02 | 2.4689E-02 | 3.8100E-05 | 2.3526E-01 | 2.0848 | 9.8103 | 87.00 |
| PC(18:2/18:2) | PC | C00157 | Glycerolphospholipid | 1.4199E-02 | 1.5086E-02 | 2.8000E-09 | 1.1309E-01 | 2.5306 | 8.5812 | 106.25 |
| PE(16:0/18:1) | PE | C00350 | Glycerolphospholipid | 4.4217E-02 | 2.4319E-02 | 2.5093E-04 | 1.3244E-01 | 0.6195 | 0.0890 | 55.00 |
| PE(16:0/18:2) | PE | C00350 | Glycerolphospholipid | 6.1609E-02 | 3.6203E-02 | 1.0000E-09 | 1.9395E-01 | 0.8143 | 0.4181 | 58.76 |
| PE(16:0/18:3) | PE | C00350 | Glycerolphospholipid | 6.1192E-03 | 4.0051E-03 | 5.0000E-10 | 2.5434E-02 | 1.1908 | 2.5852 | 65.45 |
| PE(18:0/18:1) | PE | C00350 | Glycerolphospholipid | 6.1310E-03 | 4.4443E-03 | 5.6800E-05 | 3.2296E-02 | 1.4177 | 3.0896 | 72.49 |
| PE(18:1/18:1) | PE | C00350 | Glycerolphospholipid | 2.0256E-02 | 1.3311E-02 | 2.0000E-10 | 9.1931E-02 | 1.3970 | 3.4604 | 65.71 |
| PE(18:1/18:2) | PE | C00350 | Glycerolphospholipid | 1.4914E-02 | 9.8928E-03 | 1.4584E-04 | 5.6499E-02 | 1.0857 | 1.0938 | 66.33 |
| PE(18:2/18:2) | PE | C00350 | Glycerolphospholipid | 1.3872E-03 | 1.6084E-03 | 2.7000E-09 | 1.2479E-02 | 3.1118 | 12.7468 | 115.94 |
| PE(18:3/18:2) | PE | C00350 | Glycerolphospholipid | 3.2587E-03 | 2.4881E-03 | 7.0000E-10 | 1.3311E-02 | 0.9773 | 1.2485 | 76.35 |
| PE(18:3/18:3) | PE | C00350 | Glycerolphospholipid | 7.1105E-04 | 6.1160E-04 | 7.0000E-10 | 4.0145E-03 | 1.3495 | 2.9128 | 86.01 |
| PE(20:0/18:1) | PE | C00350 | Glycerolphospholipid | 8.0612E-04 | 5.1928E-04 | 7.0000E-10 | 3.2408E-03 | 1.2911 | 2.2090 | 64.42 |
| PE(20:0/18:2) | PE | C00350 | Glycerolphospholipid | 2.5123E-03 | 1.3290E-03 | 7.9600E-06 | 8.2761E-03 | 1.1587 | 1.5687 | 52.90 |
| PE(20:0/18:3) | PE | C00350 | Glycerolphospholipid | 9.1821E-04 | 6.4162E-04 | 1.8000E-09 | 4.7435E-03 | 1.5284 | 4.4441 | 69.88 |
| PG(16:0/16:0) | PG | C00344 | Glycerolphospholipid | 8.1373E-03 | 6.5056E-03 | 1.0800E-05 | 3.8664E-02 | 1.7517 | 3.0057 | 79.95 |
| PG(16:0/18:1) | PG | C00344 | Glycerolphospholipid | 9.8152E-03 | 7.3448E-03 | 2.2700E-05 | 5.7924E-02 | 1.8755 | 5.1212 | 74.83 |
| PG(16:0/18:2) | PG | C00344 | Glycerolphospholipid | 8.3500E-03 | 5.3240E-03 | 1.6100E-05 | 3.4657E-02 | 1.5660 | 2.7601 | 63.76 |
| PG(18:0/16:0) | PG | C00344 | Glycerolphospholipid | 3.4812E-03 | 2.8262E-03 | 7.3800E-06 | 1.8593E-02 | 1.9446 | 4.4613 | 81.18 |
| PG(18:0/18:1) | PG | C00344 | Glycerolphospholipid | 7.6708E-04 | 9.4289E-04 | 1.1000E-09 | 9.8282E-03 | 3.7397 | 22.5415 | 122.92 |
| PG(18:0/18:2) | PG | C00344 | Glycerolphospholipid | 1.6522E-03 | 1.1648E-03 | 6.1500E-06 | 1.0419E-02 | 2.3509 | 9.3368 | 70.50 |
| PG(18:1/18:2) | PG | C00344 | Glycerolphospholipid | 4.7533E-04 | 3.9719E-04 | 3.2000E-09 | 2.5993E-03 | 1.9571 | 4.7865 | 83.56 |
| PI(16:0/18:1) | PI | C00626 | Glycerolphospholipid | 6.0137E-02 | 4.1633E-02 | 1.7957E-04 | 2.0349E-01 | 0.8255 | 0.3039 | 69.23 |
| PI(16:0/18:2) | PI | C00626 | Glycerolphospholipid | 1.6409E-01 | 1.0766E-01 | 1.9000E-09 | 5.0589E-01 | 0.4032 | -0.3423 | 65.61 |
| PI(16:0/18:3) | PI | C00626 | Glycerolphospholipid | 3.9205E-02 | 2.5476E-02 | 5.7257E-05 | 1.7392E-01 | 1.3337 | 3.7183 | 64.98 |
| PI(18:0/18:1) | PI | C00626 | Glycerolphospholipid | 2.9332E-02 | 1.9783E-02 | 1.8709E-04 | 1.3100E-01 | 1.2612 | 2.7445 | 67.45 |
| PI(18:1/18:1) | PI | C00626 | Glycerolphospholipid | 9.2096E-02 | 4.6874E-02 | 5.1791E-04 | 2.3001E-01 | 0.0793 | -0.4964 | 50.90 |
| PI(18:1/18:2) | PI | C00626 | Glycerolphospholipid | 4.0725E-02 | 2.2552E-02 | 1.9269E-04 | 1.2376E-01 | 0.3001 | -0.0841 | 55.37 |
| PI(18:2/18:2) | PI | C00626 | Glycerolphospholipid | 1.5436E-02 | 9.3388E-03 | 6.9400E-05 | 5.7926E-02 | 0.8007 | 1.3327 | 60.50 |
| Cer(d18:2/16:0) | Cer | C00195 | Sphingolipid | 1.4611E-05 | 8.6607E-06 | 2.0000E-10 | 6.6200E-05 | 1.5025 | 3.0281 | 59.27 |
| CerG1(d18:2/16:0) | CerG | C01190 | Sphingolipid | 5.0696E-04 | 2.9008E-04 | 7.2400E-05 | 1.6485E-03 | 1.3210 | 1.7699 | 57.22 |
| CerG1(d18:2/22:1) | CerG | C01190 | Sphingolipid | 8.9255E-06 | 5.6978E-06 | 1.5500E-06 | 3.9400E-05 | 1.9190 | 4.9367 | 63.8369 |

^a^ KEGG COMPOUND database: https://www.kegg.jp/kegg/compound/. ^b^ Lipid category was defined in the LIPID MAPS database (lipidmaps.org/). ^c^ Std Dev: standard deviation.

**Table S4.** Associations between oil-related traits and metabolites in 398 soybean RILs identified using the minimax concave penalty and smoothly clipped absolute deviation methods

| **Oil-related traits** | **Metabolites** | **Partial regression coefficient** | **P-value** | **Class** |
| --- | --- | --- | --- | --- |
| Palmitic acid | fucose | -0.0574 | 3.7700E-04 | Carbohydrates |
|  | D-Fructose 2,6-bisphosphate | 0.0038 | 4.3966E-01 | Carbohydrates |
|  | Inositol | 0.0062 | 5.7541E-01 | Carbohydrates |
|  | sucrose | 0.0111 | 5.4513E-01 | Carbohydrates |
|  | glycerol | -0.0089 | 2.1886E-01 | Carbohydrates |
|  | mannose | 0.0146 | 2.8171E-01 | Carbohydrates |
|  | myristic acid | -0.0216 | 2.8600E-02 | Lipids |
|  | Pyruvate | -0.0316 | 1.1650E-02 | Organic acids |
|  | Citraconic acid | -0.0319 | 5.4900E-03 | Organic acids |
|  | Ethanolamine | -0.0110 | 1.5430E-02 | Amino acids |
|  | leucine | -0.0138 | 4.1860E-02 | Amino acids |
|  | alanine | 0.0098 | 1.4750E-02 | Amino acids |
|  | threonine | 0.0576 | 1.3900E-03 | Amino acids |
| Stearic acid | glutamine | 0.0341 | 4.3200E-02 | Amino acids |
| Oleic acid | glycerol | 0.0680 | 2.1870E-01 | Carbohydrates |
|  | sucrose | -0.1660 | 9.8900E-02 | Carbohydrates |
|  | fucose | 0.2178 | 5.1700E-02 | Carbohydrates |
|  | D-Glucose | 0.7853 | 2.7900E-06 | Carbohydrates |
|  | 2-Hydroxybutanoic acid | 0.0229 | 4.2100E-02 | Lipids |
|  | 2-Oxobutyric acid | -0.0625 | 1.4600E-02 | Organic acids |
|  | 3-Hydroxypropionic acid | -0.0165 | 3.6600E-02 | Organic acids |
|  | oxalic acid | 0.0958 | 4.4100E-02 | Organic acids |
|  | succinic acid | 0.0187 | 3.9100E-02 | Organic acids |
|  | oxoproline | 0.0906 | 4.6940E-02 | Amino acids |
|  | serine | -0.1828 | 6.0100E-03 | Amino acids |
| Linoleic acid | glycerol | -0.0972 | 1.2600E-01 | Carbohydrates |
|  | sucrose | 0.0610 | 1.6100E-01 | Carbohydrates |
|  | fucose | -0.0253 | 2.2700E-01 | Carbohydrates |
|  | D-Glucose | -0.5885 | 4.7900E-06 | Carbohydrates |
|  | palmitoleic acid | 0.1507 | 2.6200E-02 | Lipids |
|  | stearic acid | -0.2181 | 1.7900E-03 | Lipids |
|  | 4-Aminobutyric acid(GABA) | -0.0391 | 3.6421E-02 | Amino acids |
|  | serine | 0.2258 | 1.4600E-04 | Amino acids |
| Linolenic acid | fucose | -0.0429 | 2.9300E-02 | Carbohydrates |
|  | D-Glucose | -0.1550 | 8.4600E-07 | Carbohydrates |
|  | 2-Hydroxybutanoic acid | -0.0288 | 9.4700E-03 | Lipids |
|  | Glyceryl 1-phosphate | -0.0172 | 3.5060E-02 | Lipids |
|  | prostaglandin A | -0.0242 | 1.2590E-02 | Lipids |
|  | 2-Oxobutyric acid | 0.0187 | 2.2330E-02 | Organic acids |
|  | Glucose-1-phosphate | -0.0062 | 2.7860E-02 | Organic acids |
|  | oxalic acid | -0.0233 | 1.6440E-02 | Organic acids |
|  | 3-Methyl-2-oxobutanoic acid | 0.0281 | 7.8200E-03 | Organic acids |
|  | 3-Hydroxypropionic acid | 0.0380 | 4.2300E-03 | Organic acids |
|  | L-Malic acid | -0.0353 | 3.8200E-03 | Organic acids |
|  | threonine | -0.0253 | 4.2160E-02 | Amino acids |
|  | serine | 0.0595 | 8.1000E-03 | Amino acids |
| Oil content | D-Fructose 2,6-bisphosphate | 0.0006 | 2.1869E-01 | Carbohydrates |
|  | D-Glucose | 0.0629 | 6.7590E-02 | Carbohydrates |
|  | glycerol | -0.0336 | 5.7900E-02 | Carbohydrates |
|  | fucose | -0.1106 | 5.0600E-03 | Carbohydrates |
|  | sucrose | 0.0074 | 6.1880E-01 | Carbohydrates |
|  | linoleic acid | -0.0432 | 1.7571E-02 | Lipids |
|  | oleic acid | 0.1482 | 2.3700E-04 | Lipids |
|  | 1-Hexadecanol | -0.0805 | 5.5800E-03 | Lipids |
|  | 2-Aminooctadecane-1,3-diol | 0.1007 | 3.5060E-02 | Lipids |
|  | Glyceryl 1-phosphate | 0.1334 | 1.2590E-02 | Lipids |
|  | Sphingosine | 0.0612 | 2.9760E-02 | Lipids |
|  | succinic acid | 0.0633 | 3.5100E-04 | Organic acids |
|  | glutathione | 0.0584 | 1.8740E-02 | Amino acids |
|  | oxoproline | -0.0528 | 3.5730E-02 | Amino acids |
|  | leucine | -0.0691 | 2.7600E-02 | Amino acids |
|  | alanine | -0.0766 | 6.1900E-03 | Amino acids |

**Table S5.** Associations between oil-related traits and lipids in 398 soybean RILs identified using the minimax concave penalty and smoothly clipped absolute deviation methods

| **Oil-related traits** | **Lipids** | **Partial regression coefficient** | **P-value** | **Category** |
| --- | --- | --- | --- | --- |
| Palmitic acid | TG(18:1/18:2/18:2) | 0.0688 | 0.0311 | Glycerolipid |
|  | TG(18:3/18:2/18:3) | 0.0780 | 0.0208 | Glycerolipid |
|  | TG(16:0/18:1/18:3) | -0.1133 | 0.0002 | Glycerolipid |
|  | TG(16:0/18:2/18:2) | -0.0989 | 0.0012 | Glycerolipid |
|  | TG(16:0/18:2/18:3) | -0.1110 | 0.0031 | Glycerolipid |
|  | TG(18:1/18:1/18:1) | 0.1127 | 0.0082 | Glycerolipid |
|  | TG(18:1/18:2/18:3) | -0.0590 | 0.0062 | Glycerolipid |
|  | TG(18:3/18:2/18:2) | 0.0942 | 0.0024 | Glycerolipid |
|  | TG(20:0/18:1/18:1) | -0.0737 | 0.0027 | Glycerolipid |
|  | TG(20:1/18:2/18:3) | 0.0852 | 0.0023 | Glycerolipid |
|  | DG(18:0/16:0) | 0.1295 | 0.0000 | Glycerolipid |
|  | TG(16:0/18:1/18:1) | -0.1352 | 0.0001 | Glycerolipid |
|  | PE(20:0/18:3) | -0.0060 | 0.0395 | Glycerolphospholipid |
|  | PI(16:0/18:2) | 0.0107 | 0.0448 | Glycerolphospholipid |
|  | LPC(18:1) | 0.0207 | 0.0271 | Glycerolphospholipid |
|  | PE(18:3/18:3) | 0.0990 | 0.0002 | Glycerolphospholipid |
|  | CerG1(d18:2/16:0) | 0.0745 | 0.0002 | Sphingolipid |
| Stearic acid | FA(18:0) | -0.0331 | 0.0258 | Fatty acid |
|  | TG(16:0/16:0/18:1) | 0.0721 | 0.0001 | Glycerolipid |
|  | DG(18:0/18:0) | 0.0364 | 0.0000 | Glycerolipid |
|  | PC(18:1/18:1) | 0.0066 | 0.0265 | Glycerolphospholipid |
|  | LPC(18:0) | 0.0113 | 0.0064 | Glycerolphospholipid |
|  | PE(18:1/18:1) | -0.0560 | 0.0044 | Glycerolphospholipid |
|  | CerG1(d18:2/16:0) | 0.0095 | 0.0368 | Sphingolipid |
| Oleic acid | TG(16:0/18:1/18:1) | -0.8851 | 0.0000 | Glycerolipid |
|  | TG(16:0/18:1/18:3) | -0.3020 | 0.0248 | Glycerolipid |
|  | TG(18:3/18:2/18:2) | 0.7294 | 0.0035 | Glycerolipid |
|  | TG(18:1/18:1/18:1) | -0.4445 | 0.0003 | Glycerolipid |
|  | TG(20:1/18:1/18:2) | 0.9365 | 0.0001 | Glycerolipid |
|  | TG(18:0/16:0/18:1) | -0.9641 | 0.0000 | Glycerolipid |
|  | DGDG(16:0/18:2) | 1.1392 | 0.0000 | Glycerolipid |
|  | LPC(16:0) | -0.0994 | 0.0442 | Glycerolphospholipid |
|  | PI(16:0/18:2) | 0.1145 | 0.0240 | Glycerolphospholipid |
|  | LPC(18:1) | -0.2350 | 0.0024 | Glycerolphospholipid |
|  | CL(18:2/18:1/18:1/18:2) | -1.1006 | 0.0008 | Glycerolphospholipid |
|  | PG(16:0/18:1) | -0.8335 | 0.0001 | Glycerolphospholipid |
|  | CL(18:3/18:2/18:2/18:2) | 0.5960 | 0.0019 | Glycerolphospholipid |
|  | CerG1(d18:2/16:0) | -0.3400 | 0.0054 | Sphingolipid |
| Linoleic acid | FA(22:1) | 0.1554 | 0.0269 | Fatty acid |
|  | OAHFA(18:1/18:0) | -0.1519 | 0.0287 | Fatty acid |
|  | TG(16:0/18:1/18:1) | 0.6166 | 0.0000 | Glycerolipid |
|  | TG(18:0/16:0/18:1) | 0.6980 | 0.0000 | Glycerolipid |
|  | TG(18:3/18:2/18:2) | -0.4285 | 0.0018 | Glycerolipid |
|  | TG(20:1/18:1/18:2) | -0.4008 | 0.0005 | Glycerolipid |
|  | DGDG(16:0/18:2) | -0.8425 | 0.0000 | Glycerolipid |
|  | PG(16:0/16:0) | -0.5011 | 0.0264 | Glycerolphospholipid |
|  | LPC(18:1) | 0.0708 | 0.0275 | Glycerolphospholipid |
|  | PI(16:0/18:2) | -0.1246 | 0.0201 | Glycerolphospholipid |
|  | PG(16:0/18:1) | 0.7456 | 0.0001 | Glycerolphospholipid |
|  | CL(18:2/18:1/18:1/18:2) | 1.0331 | 0.0001 | Glycerolphospholipid |
|  | CL(18:3/18:2/18:2/18:2) | -0.3046 | 0.0271 | Glycerolphospholipid |
| Linolenic acid | FA(20:1) | -0.0383 | 0.0109 | Fatty acid |
|  | TG(16:0/16:0/18:1) | 0.0376 | 0.0195 | Glycerolipid |
|  | DG(18:0/18:0) | 0.6250 | 0.0004 | Glycerolipid |
|  | DG(18:2/18:2) | 0.3491 | 0.0213 | Glycerolipid |
|  | DG(18:3/18:2) | -0.1064 | 0.0480 | Glycerolipid |
|  | DGDG(16:0/18:2) | -0.0656 | 0.0005 | Glycerolipid |
|  | DG(18:0/16:0) | 0.6398 | 0.0151 | Glycerolipid |
|  | DG(16:0/16:0) | -0.4468 | 0.0067 | Glycerolipid |
|  | TG(16:0/18:1/18:2) | 0.0648 | 0.0064 | Glycerolipid |
|  | TG(18:1/18:1/18:2) | 0.1014 | 0.0025 | Glycerolipid |
|  | TG(18:3/18:2/18:3) | -0.0628 | 0.0021 | Glycerolipid |
|  | DG(18:0/18:1) | -0.5865 | 0.0001 | Glycerolipid |
|  | DG(18:1/18:1) | 0.7627 | 0.0000 | Glycerolipid |
|  | LPC(16:0) | 0.0692 | 0.0004 | Glycerolphospholipid |
|  | LPE(16:0) | -0.0732 | 0.0008 | Glycerolphospholipid |
|  | PI(16:0/18:2) | -0.0612 | 0.0009 | Glycerolphospholipid |
|  | CerG1(d18:2/16:0) | 0.0187 | 0.0145 | Sphingolipid |
| Oil content | FA(18:0) | 0.2595 | 0.0001 | Fatty acid |
|  | FA(18:2) | -0.2741 | 0.0005 | Fatty acid |
|  | FA(18:3) | 0.2298 | 0.0062 | Fatty acid |
|  | TG(16:0/16:0/18:2) | -0.0072 | 0.0421 | Glycerolipid |
|  | TG(16:0/18:1/18:2) | -0.0034 | 0.0348 | Glycerolipid |
|  | TG(18:1/18:1/18:2) | -0.1632 | 0.0202 | Glycerolipid |
|  | DGDG(18:3/18:3) | -0.1419 | 0.0006 | Glycerolipid |
|  | DGDG(16:0/18:3) | 0.1168 | 0.0064 | Glycerolipid |
|  | TG(18:1/18:1/18:3) | -0.2959 | 0.0000 | Glycerolipid |
|  | TG(20:1/18:2/18:2) | 0.3585 | 0.0000 | Glycerolipid |
|  | TG(20:1/18:2/18:3) | 0.0738 | 0.0003 | Glycerolipid |
|  | TG(16:0/18:2/18:2) | -0.2234 | 0.0000 | Glycerolipid |
|  | TG(18:3/18:2/18:3) | 0.2129 | 0.0002 | Glycerolipid |
|  | DG(18:0/18:1) | 0.6551 | 0.0000 | Glycerolipid |
|  | DG(18:1/18:1) | -0.7960 | 0.0000 | Glycerolipid |
|  | DG(16:0/16:0) | 0.3287 | 0.0000 | Glycerolipid |
|  | DG(16:0/18:2) | -0.1384 | 0.0423 | Glycerolipid |
|  | PG(18:0/16:0) | 0.0889 | 0.0070 | Glycerolphospholipid |
|  | PG(18:1/18:2) | 0.0503 | 0.0448 | Glycerolphospholipid |
|  | PI(18:1/18:2) | -0.2587 | 0.0000 | Glycerolphospholipid |
|  | CerG1(d18:2/22:1) | 0.0871 | 0.0017 | Sphingolipid |

**Table S6.** Associations between metabolites and metabolites, between metabolites and lipids, and between lipids and lipids in 398 soybean RILs identified using the Gaussian graphical model

| **Node 1** | **Node 2** | **CC** | **P-value** | **Q-value** | **PP** | **Node 1** | **Node 2** | **CC** | **P-value** | **Q-value** | **PP** |
| --- | --- | --- | --- | --- | --- | --- | --- | --- | --- | --- | --- |
| glutamine | glycerol | -0.1846 | 2.2200E-16 | 5.3200E-14 | 1.0000 | PE(18:0/18:1) | PE(18:1/18:2) | 0.1549 | 5.9200E-12 | 1.0200E-09 | 1.0000 |
| glutathione | glycerol | -0.1646 | 2.4900E-13 | 4.8800E-11 | 1.0000 | PE(18:0/18:1) | PE(18:1/22:0) | -0.0996 | 1.0500E-05 | 6.1182E-04 | 0.9953 |
| glutamine | leucine | -0.1408 | 4.2000E-10 | 5.7200E-08 | 1.0000 | PE(18:0/18:1) | PE(20:0/18:1) | 0.1781 | 2.2200E-15 | 5.1400E-13 | 1.0000 |
| mannose | succinic acid | -0.1310 | 6.2400E-09 | 7.3800E-07 | 1.0000 | PE(18:0/18:1) | PE(20:0/18:2) | 0.0942 | 3.0600E-05 | 1.5044E-03 | 0.9895 |
| glycerol | oxoproline | -0.1271 | 1.7600E-08 | 1.9200E-06 | 1.0000 | PE(18:0/18:1) | PG(16:0/18:1) | -0.1150 | 3.5300E-07 | 3.0600E-05 | 0.9997 |
| 3-Methyl-2-oxobutanoic acid | palmitoleic acid | -0.1149 | 3.5900E-07 | 3.1100E-05 | 0.9997 | PE(18:0/18:1) | PG(18:0/18:1) | 0.1494 | 3.3200E-11 | 5.3000E-09 | 1.0000 |
| palmitic acid | palmitoleic acid | -0.1146 | 3.8600E-07 | 3.3100E-05 | 0.9997 | PE(18:0/18:1) | PI(16:0/18:1) | -0.0974 | 1.6200E-05 | 8.8729E-04 | 0.9940 |
| beta-Alanine | sucrose | -0.1130 | 5.5500E-07 | 4.5800E-05 | 0.9995 | PE(18:1/18:1) | PE(18:1/18:2) | 0.5015 | 2.2200E-16 | 5.3200E-14 | 1.0000 |
| proline | sucrose | -0.1105 | 9.8400E-07 | 7.6600E-05 | 0.9994 | PE(18:1/18:1) | PE(18:1/22:0) | 0.1421 | 2.8200E-10 | 3.9600E-08 | 1.0000 |
| 3-Methyl-2-oxobutanoic acid | glutathione | -0.1081 | 1.7000E-06 | 1.2444E-04 | 0.9990 | PE(18:1/18:1) | PE(22:0/18:3) | -0.0906 | 6.0900E-05 | 2.6685E-03 | 0.9833 |
| 3-Methyl-2-oxobutanoic acid | isocitric acid | -0.1081 | 1.7100E-06 | 1.2508E-04 | 0.9990 | PE(18:1/18:1) | PG(16:0/18:1) | 0.0932 | 3.7100E-05 | 1.7694E-03 | 0.9895 |
| succinic acid | Threonate | -0.1046 | 3.6200E-06 | 2.4176E-04 | 0.9983 | PE(18:1/18:1) | SQDG(16:0/18:1) | 0.1072 | 2.0700E-06 | 1.4765E-04 | 0.9990 |
| palmitic acid | putrescine | -0.1045 | 3.7200E-06 | 2.4773E-04 | 0.9983 | PE(18:1/18:1) | SQDG(16:0/18:2) | -0.1238 | 4.1000E-08 | 4.2200E-06 | 0.9999 |
| oxoproline | Threonate | -0.1040 | 4.1200E-06 | 2.6965E-04 | 0.9983 | PE(18:1/18:1) | Abietic Acid | 0.1034 | 4.7100E-06 | 3.0359E-04 | 0.9975 |
| L-homoserine | Inositol | -0.1023 | 5.8900E-06 | 3.7021E-04 | 0.9967 | PE(18:1/18:1) | L-kynurenine | -0.1124 | 6.3500E-07 | 5.1800E-05 | 0.9994 |
| 2-Hydroxybutanoic acid | glutathione | -0.1004 | 8.8500E-06 | 5.2878E-04 | 0.9961 | PE(18:1/18:1) | Maleamate | 0.0975 | 1.5800E-05 | 8.7039E-04 | 0.9940 |
| linolenic acid | oxalic acid | -0.1003 | 8.9900E-06 | 5.3587E-04 | 0.9961 | PE(18:1/18:1) | PE(18:1/18:2) | 0.1772 | 3.1100E-15 | 7.1200E-13 | 1.0000 |
| Sphingosine | Pyruvate | -0.0993 | 1.1100E-05 | 6.4541E-04 | 0.9953 | PE(18:1/18:1) | PG(16:0/18:2) | -0.0934 | 3.5800E-05 | 1.7185E-03 | 0.9895 |
| glutamine | oxalacetic acid | -0.0985 | 1.3000E-05 | 7.3500E-04 | 0.9953 | PE(18:1/18:1) | PI(16:0/18:2) | -0.1329 | 3.7400E-09 | 4.5500E-07 | 1.0000 |
| oleic acid | oxalacetic acid | -0.0961 | 2.1200E-05 | 1.1184E-03 | 0.9933 | PE(18:1/18:2) | PE(18:2/18:2) | 0.5416 | 2.2200E-16 | 5.3200E-14 | 1.0000 |
| 3-Methyl-2-oxobutanoic acid | D-Fructose 2,6-bisphosphate | -0.0955 | 2.3900E-05 | 1.2322E-03 | 0.9933 | PE(18:1/18:2) | PG(16:0/18:1) | -0.0919 | 4.8100E-05 | 2.1975E-03 | 0.9865 |
| fumaric acid | succinic acid | -0.0945 | 2.8800E-05 | 1.4331E-03 | 0.9933 | PE(18:1/18:2) | PG(18:0/18:1) | -0.0930 | 3.8800E-05 | 1.8388E-03 | 0.9895 |
| alpha-ketoisocaproic acid | citrulline | -0.0944 | 2.9500E-05 | 1.4604E-03 | 0.9933 | PE(18:1/18:2) | PE(18:2/18:2) | 0.3107 | 2.2200E-16 | 5.3200E-14 | 1.0000 |
| 2-Oxoadipate | Arachidic acid | -0.0928 | 4.0400E-05 | 1.9006E-03 | 0.9895 | PE(18:1/18:2) | PG(18:1/18:2) | 0.2122 | 2.2200E-16 | 5.3200E-14 | 1.0000 |
| 3-Methyl-2-oxobutanoic acid | mannose | -0.0925 | 4.2800E-05 | 1.9895E-03 | 0.9881 | PE(18:1/18:2) | FA(28:4) | 0.0962 | 2.0700E-05 | 1.0950E-03 | 0.9933 |
| 2-Hydroxybutanoic acid | sucrose | -0.0925 | 4.2900E-05 | 1.9944E-03 | 0.9865 | PE(18:1/22:0) | PE(18:1/24:0) | 0.2939 | 2.2200E-16 | 5.3200E-14 | 1.0000 |
| linolenic acid | succinic acid | 0.0915 | 5.1100E-05 | 2.3106E-03 | 0.9865 | PE(18:1/22:0) | PE(20:0/18:1) | 0.4650 | 2.2200E-16 | 5.3200E-14 | 1.0000 |
| fumaric acid | glutathione | 0.0922 | 4.4800E-05 | 2.0709E-03 | 0.9865 | PE(18:1/22:0) | PE(20:0/18:2) | -0.0972 | 1.7000E-05 | 9.2840E-04 | 0.9933 |
| Glyceryl 1-phosphate | lauric acid | 0.0923 | 4.4100E-05 | 2.0441E-03 | 0.9865 | PE(18:1/22:0) | PE(22:0/18:2) | 0.1473 | 6.1000E-11 | 9.4200E-09 | 1.0000 |
| glutathione | Threonate | 0.0924 | 4.3400E-05 | 2.0144E-03 | 0.9865 | PE(18:1/22:0) | PE(22:0/18:3) | 0.1626 | 4.9000E-13 | 9.3900E-11 | 1.0000 |
| 2-Hydroxybutanoic acid | Octanoic acid | 0.0926 | 4.1900E-05 | 1.9578E-03 | 0.9895 | PE(18:1/22:0) | uracil | -0.1096 | 1.2100E-06 | 9.1900E-05 | 0.9991 |
| Methyl jasmonate | oxalic acid | 0.0945 | 2.9200E-05 | 1.4476E-03 | 0.9933 | PE(18:1/24:0) | PE(20:0/18:1) | 0.2517 | 2.2200E-16 | 5.3200E-14 | 1.0000 |
| 1-Hexadecanol | oxalic acid | 0.0946 | 2.8600E-05 | 1.4222E-03 | 0.9933 | PE(18:1/24:0) | PE(24:0/18:2) | 0.2436 | 2.2200E-16 | 5.3200E-14 | 1.0000 |
| linolenic acid | mannose | 0.0947 | 2.8000E-05 | 1.4018E-03 | 0.9933 | PE(18:1/24:0) | PE(24:0/18:3) | 0.1542 | 7.3100E-12 | 1.2500E-09 | 1.0000 |
| fucose | Isoleucine | 0.0948 | 2.7300E-05 | 1.3710E-03 | 0.9933 | PE(18:1/24:0) | PE(25:0/18:2) | 0.1392 | 6.5900E-10 | 8.7700E-08 | 1.0000 |
| 4-Aminobutyric acid(GABA) | lauric acid | 0.0948 | 2.7100E-05 | 1.3634E-03 | 0.9933 | PE(18:1/24:0) | SQDG(16:0/18:2) | -0.0932 | 3.7600E-05 | 1.7889E-03 | 0.9895 |
| malonic acid | Methyl jasmonate | 0.0954 | 2.4100E-05 | 1.2406E-03 | 0.9933 | PE(18:1/24:0) | phosphomycin | -0.1060 | 2.6900E-06 | 1.8634E-04 | 0.9984 |
| Capric Acid | Glucose-6-phosphate | 0.0973 | 1.6500E-05 | 9.0044E-04 | 0.9933 | PE(18:2/18:2) | PE(18:2/23:0) | 0.1139 | 4.5600E-07 | 3.8500E-05 | 0.9997 |
| linolenic acid | palmitic acid | 0.0975 | 1.5900E-05 | 8.7419E-04 | 0.9940 | PE(18:2/18:2) | PE(18:3/18:2) | 0.1006 | 8.5400E-06 | 5.1247E-04 | 0.9961 |
| oxoproline | palmitic acid | 0.0986 | 1.2700E-05 | 7.2148E-04 | 0.9953 | PE(18:2/18:2) | FA(28:4) | 0.2888 | 2.2200E-16 | 5.3200E-14 | 1.0000 |
| D-Fructose 2,6-bisphosphate | glutamine | 0.0990 | 1.1800E-05 | 6.7922E-04 | 0.9953 | PE(18:2/18:2) | PE(18:3/18:2) | 0.3051 | 2.2200E-16 | 5.3200E-14 | 1.0000 |
| glutamine | Threonate | 0.0999 | 9.7500E-06 | 5.7469E-04 | 0.9961 | PE(18:2/18:2) | PG(16:0/16:0) | -0.1255 | 2.6300E-08 | 2.7800E-06 | 0.9999 |
| L-Malic acid | proline | 0.1003 | 9.0300E-06 | 5.3801E-04 | 0.9961 | PE(18:2/18:2) | PI(16:0/18:2) | -0.1111 | 8.6600E-07 | 6.8500E-05 | 0.9994 |
| Octanoic acid | lauric acid | 0.1006 | 8.3800E-06 | 5.0444E-04 | 0.9961 | PE(18:2/18:2) | FA(18:3) | -0.0924 | 4.3600E-05 | 2.0231E-03 | 0.9865 |
| 1-Hexadecanol | fumaric acid | 0.1009 | 7.9400E-06 | 4.8112E-04 | 0.9961 | PE(18:2/23:0) | PE(18:3/23:0) | 0.1479 | 5.1900E-11 | 8.0900E-09 | 1.0000 |
| linoleic acid | succinic acid | 0.1013 | 7.3900E-06 | 4.5181E-04 | 0.9967 | PE(18:2/23:0) | PE(22:0/18:2) | 0.2353 | 2.2200E-16 | 5.3200E-14 | 1.0000 |
| oxoproline | palmitoleic acid | 0.1038 | 4.3600E-06 | 2.8367E-04 | 0.9975 | PE(18:2/23:0) | PE(24:0/18:2) | 0.2268 | 2.2200E-16 | 5.3200E-14 | 1.0000 |
| malonic acid | palmitic acid | 0.1039 | 4.1900E-06 | 2.7377E-04 | 0.9983 | PE(18:2/23:0) | PE(25:0/18:2) | 0.2853 | 2.2200E-16 | 5.3200E-14 | 1.0000 |
| D-Glyceric acid | L-Malic acid | 0.1040 | 4.1500E-06 | 2.7148E-04 | 0.9983 | PE(18:3/18:2) | PE(18:3/18:3) | 0.3673 | 2.2200E-16 | 5.3200E-14 | 1.0000 |
| alanine | linoleic acid | 0.1042 | 3.9800E-06 | 2.6230E-04 | 0.9983 | PE(18:3/18:2) | PE(18:3/23:0) | -0.1025 | 5.6600E-06 | 3.5734E-04 | 0.9967 |
| Octanoic acid | Methyl jasmonate | 0.1048 | 3.5100E-06 | 2.3537E-04 | 0.9983 | PE(18:3/18:2) | PE(22:0/18:3) | 0.1343 | 2.5900E-09 | 3.2100E-07 | 1.0000 |
| Ethanolamine | fumaric acid | 0.1049 | 3.4300E-06 | 2.3050E-04 | 0.9983 | PE(18:3/18:3) | PE(18:3/23:0) | 0.1595 | 1.3700E-12 | 2.5400E-10 | 1.0000 |
| oxalic acid | Pyruvate | 0.1052 | 3.2200E-06 | 2.1820E-04 | 0.9983 | PE(18:3/18:3) | PE(20:0/18:3) | 0.2163 | 2.2200E-16 | 5.3200E-14 | 1.0000 |
| L-homoserine | palmitic acid | 0.1076 | 1.9000E-06 | 1.3688E-04 | 0.9990 | PE(18:3/18:3) | SQDG(16:0/18:3) | -0.0997 | 1.0100E-05 | 5.9373E-04 | 0.9953 |
| fumaric acid | leucine | 0.1080 | 1.7300E-06 | 1.2640E-04 | 0.9990 | PE(18:3/23:0) | PE(20:0/18:3) | 0.1188 | 1.4200E-07 | 1.3300E-05 | 0.9999 |
| Citraconic acid | fucose | 0.1082 | 1.6600E-06 | 1.2180E-04 | 0.9990 | PE(18:3/23:0) | PE(24:0/18:3) | 0.3587 | 2.2200E-16 | 5.3200E-14 | 1.0000 |
| oxalic acid | Threonate | 0.1087 | 1.4800E-06 | 1.1018E-04 | 0.9991 | PE(18:3/23:0) | FA(21:6) | 0.1295 | 9.3200E-09 | 1.0700E-06 | 1.0000 |
| L-homoserine | oxoproline | 0.1104 | 1.0100E-06 | 7.8100E-05 | 0.9994 | PE(20:0/18:1) | PE(20:0/18:2) | 0.1450 | 1.2300E-10 | 1.8000E-08 | 1.0000 |
| glutamine | palmitoleic acid | 0.1115 | 7.8000E-07 | 6.2400E-05 | 0.9994 | PE(20:0/18:1) | PE(20:0/18:3) | 0.1302 | 7.7600E-09 | 9.0400E-07 | 1.0000 |
| oxoproline | serine | 0.1116 | 7.7200E-07 | 6.1900E-05 | 0.9994 | PE(20:0/18:2) | PE(20:0/18:3) | 0.2883 | 2.2200E-16 | 5.3200E-14 | 1.0000 |
| oleic acid | serine | 0.1130 | 5.6300E-07 | 4.6400E-05 | 0.9994 | PE(20:0/18:2) | PE(22:0/18:2) | 0.2465 | 2.2200E-16 | 5.3200E-14 | 1.0000 |
| Glyceryl 1-phosphate | mannose | 0.1131 | 5.4500E-07 | 4.5100E-05 | 0.9996 | PE(20:0/18:2) | PE(22:0/18:3) | 0.1187 | 1.4500E-07 | 1.3600E-05 | 0.9999 |
| Ethanolamine | Methyl jasmonate | 0.1141 | 4.3700E-07 | 3.7000E-05 | 0.9997 | PE(20:0/18:2) | PE(25:0/18:2) | 0.1453 | 1.1200E-10 | 1.6600E-08 | 1.0000 |
| D-Glucose | glutathione | 0.1146 | 3.8100E-07 | 3.2800E-05 | 0.9997 | PE(20:0/18:2) | PG(18:0/16:0) | 0.1474 | 6.0300E-11 | 9.3200E-09 | 1.0000 |
| 2-Oxoadipate | alpha-ketoisocaproic acid | 0.1166 | 2.4000E-07 | 2.1500E-05 | 0.9998 | PE(20:0/18:3) | PE(22:0/18:3) | 0.1835 | 4.4400E-16 | 1.0500E-13 | 1.0000 |
| D-Glucose | oleic acid | 0.1195 | 1.1800E-07 | 1.1300E-05 | 0.9999 | PE(20:0/18:3) | PG(18:0/16:0) | 0.1515 | 1.7300E-11 | 2.8600E-09 | 1.0000 |
| citric acid | fumaric acid | 0.1204 | 9.5600E-08 | 9.2500E-06 | 0.9999 | PE(22:0/18:2) | PE(22:0/18:3) | 0.2869 | 2.2200E-16 | 5.3200E-14 | 1.0000 |
| Sphingosine | succinic acid | 0.1211 | 7.9400E-08 | 7.7700E-06 | 0.9999 | PE(22:0/18:2) | PE(25:0/18:2) | -0.1550 | 5.7900E-12 | 9.9900E-10 | 1.0000 |
| 2-Oxobutyric acid | alpha-ketoisocaproic acid | 0.1212 | 7.7300E-08 | 7.5800E-06 | 0.9999 | PE(22:0/18:3) | PE(24:0/18:3) | 0.1408 | 4.2200E-10 | 5.7500E-08 | 1.0000 |
| linolenic acid | Inositol | 0.1216 | 7.0100E-08 | 6.9100E-06 | 0.9999 | PE(22:0/18:3) | PE(25:0/18:2) | -0.1284 | 1.2600E-08 | 1.4200E-06 | 1.0000 |
| glycerol | isocitric acid | 0.1247 | 3.2600E-08 | 3.4100E-06 | 0.9999 | PE(22:0/18:3) | FA(18:3) | 0.0966 | 1.9100E-05 | 1.0219E-03 | 0.9933 |
| citric acid | Inositol | 0.1273 | 1.6700E-08 | 1.8300E-06 | 1.0000 | PE(24:0/18:2) | PE(24:0/18:3) | 0.2136 | 2.2200E-16 | 5.3200E-14 | 1.0000 |
| glycerol | leucine | 0.1280 | 1.3900E-08 | 1.5500E-06 | 1.0000 | PE(24:0/18:2) | PE(25:0/18:2) | 0.3943 | 2.2200E-16 | 5.3200E-14 | 1.0000 |
| Octanoic acid | palmitoleic acid | 0.1313 | 5.7200E-09 | 6.8000E-07 | 1.0000 | PE(24:0/18:3) | PE(25:0/18:2) | 0.1014 | 7.2000E-06 | 4.4172E-04 | 0.9967 |
| D-Glucose | oxoproline | 0.1319 | 4.9900E-09 | 5.9800E-07 | 1.0000 | PG(16:0/16:0) | PG(16:0/18:1) | 0.1293 | 9.7900E-09 | 1.1200E-06 | 1.0000 |
| palmitic acid | succinic acid | 0.1332 | 3.4700E-09 | 4.2300E-07 | 1.0000 | PG(16:0/16:0) | PG(16:0/18:2) | 0.2006 | 2.2200E-16 | 5.3200E-14 | 1.0000 |
| L-Malic acid | threonine | 0.1342 | 2.6400E-09 | 3.2700E-07 | 1.0000 | PG(16:0/16:0) | PG(18:0/16:0) | 0.4800 | 2.2200E-16 | 5.3200E-14 | 1.0000 |
| Glucose-6-phosphate | malonic acid | 0.1351 | 2.0800E-09 | 2.6100E-07 | 1.0000 | PG(16:0/18:1) | PG(16:0/18:2) | 0.1432 | 2.0900E-10 | 2.9900E-08 | 1.0000 |
| Sphingosine | linolenic acid | 0.1387 | 7.5400E-10 | 9.9900E-08 | 1.0000 | PG(16:0/18:1) | PG(18:0/18:1) | 0.3254 | 2.2200E-16 | 5.3200E-14 | 1.0000 |
| fucose | Threonate | 0.1449 | 1.2700E-10 | 1.8600E-08 | 1.0000 | PG(16:0/18:1) | PG(18:0/18:2) | 0.1945 | 2.2200E-16 | 5.3200E-14 | 1.0000 |
| 2-Oxobutyric acid | lauric acid | 0.1450 | 1.2100E-10 | 1.7800E-08 | 1.0000 | PG(16:0/18:1) | PG(18:1/18:2) | 0.1510 | 2.0100E-11 | 3.2900E-09 | 1.0000 |
| 3-Hydroxypropionic acid | alanine | 0.1471 | 6.4900E-11 | 9.9800E-09 | 1.0000 | PG(16:0/18:2) | PG(18:0/18:1) | -0.1987 | 2.2200E-16 | 5.3200E-14 | 1.0000 |
| Glyceryl 1-phosphate | fucose | 0.1479 | 5.1700E-11 | 8.0500E-09 | 1.0000 | PG(16:0/18:2) | PG(18:0/18:2) | 0.2570 | 2.2200E-16 | 5.3200E-14 | 1.0000 |
| alpha-ketoisocaproic acid | lauric acid | 0.1503 | 2.4900E-11 | 4.0400E-09 | 1.0000 | PG(16:0/18:2) | PG(18:1/18:2) | 0.2930 | 2.2200E-16 | 5.3200E-14 | 1.0000 |
| linolenic acid | Threonate | 0.1570 | 3.0700E-12 | 5.4900E-10 | 1.0000 | PG(16:0/18:2) | FA(18:3) | 0.1172 | 2.0700E-07 | 1.8900E-05 | 0.9998 |
| putrescine | succinic acid | 0.1597 | 1.2600E-12 | 2.3300E-10 | 1.0000 | PG(16:0/18:2) | FA(29:0) | 0.0929 | 3.9600E-05 | 1.8675E-03 | 0.9895 |
| L-homoserine | Methyl jasmonate | 0.1799 | 1.3300E-15 | 3.1200E-13 | 1.0000 | PG(18:0/16:0) | PG(18:0/18:2) | 0.1127 | 6.0200E-07 | 4.9400E-05 | 0.9994 |
| Octanoic acid | glutathione | 0.1899 | 2.2200E-16 | 5.3200E-14 | 1.0000 | PG(18:0/18:1) | PG(18:0/18:2) | 0.3939 | 2.2200E-16 | 5.3200E-14 | 1.0000 |
| D-Glucose | leucine | 0.2034 | 2.2200E-16 | 5.3200E-14 | 1.0000 | PG(18:0/18:1) | PG(18:1/18:2) | 0.1099 | 1.1400E-06 | 8.6900E-05 | 0.9991 |
| D-Glyceric acid | fucose | 0.2269 | 2.2200E-16 | 5.3200E-14 | 1.0000 | PG(18:0/18:2) | PG(18:1/18:2) | 0.2316 | 2.2200E-16 | 5.3200E-14 | 1.0000 |
| isocitric acid | Isoleucine | 0.4602 | 2.2200E-16 | 5.3200E-14 | 1.0000 | PG(18:1/18:2) | FA(16:4) | -0.0940 | 3.1800E-05 | 1.5529E-03 | 0.9895 |
| TG(16:0/18:2/18:2) | palmitic acid | -0.1225 | 5.7300E-08 | 5.7500E-06 | 0.9999 | PI(16:0/18:1) | PI(16:0/18:2) | -0.2466 | 2.2200E-16 | 5.3200E-14 | 1.0000 |
| TG(18:1/18:1/18:2) | serine | -0.1203 | 9.7200E-08 | 9.4000E-06 | 0.9999 | PI(16:0/18:1) | PI(18:0/18:1) | 0.3673 | 2.2200E-16 | 5.3200E-14 | 1.0000 |
| TG(20:1/18:1/18:2) | proline | -0.1124 | 6.4600E-07 | 5.2700E-05 | 0.9994 | PI(16:0/18:1) | PI(18:1/18:1) | -0.2268 | 2.2200E-16 | 5.3200E-14 | 1.0000 |
| TG(16:0/18:2/18:2) | 3-Methyl-2-oxobutanoic acid | -0.1114 | 8.0800E-07 | 6.4400E-05 | 0.9994 | PI(16:0/18:1) | PI(18:2/18:2) | -0.2294 | 2.2200E-16 | 5.3200E-14 | 1.0000 |
| TG(16:0/18:1/18:1) | linolenic acid | -0.1004 | 8.8200E-06 | 5.2741E-04 | 0.9961 | PI(16:0/18:2) | PI(16:0/18:3) | -0.2215 | 2.2200E-16 | 5.3200E-14 | 1.0000 |
| LPC(18:2) | palmitoleic acid | -0.0994 | 1.0800E-05 | 6.2884E-04 | 0.9953 | PI(16:0/18:2) | PI(18:0/18:1) | -0.3690 | 2.2200E-16 | 5.3200E-14 | 1.0000 |
| TG(20:1/18:2/18:3) | L-Malic acid | -0.0960 | 2.1400E-05 | 1.1268E-03 | 0.9933 | PI(16:0/18:2) | PI(18:1/18:1) | -0.1388 | 7.4000E-10 | 9.8000E-08 | 1.0000 |
| TG(16:0/16:0/18:2) | 2-Oxobutyric acid | -0.0949 | 2.6900E-05 | 1.3579E-03 | 0.9933 | PI(16:0/18:2) | PI(18:1/18:2) | -0.1599 | 1.2000E-12 | 2.2300E-10 | 1.0000 |
| DG(18:3/18:2) | D-Glyceric acid | -0.0935 | 3.4800E-05 | 1.6782E-03 | 0.9895 | PI(16:0/18:2) | FA(18:1) | -0.1364 | 1.4300E-09 | 1.8400E-07 | 1.0000 |
| TG(16:0/18:1/18:3) | Octanoic acid | -0.0927 | 4.0700E-05 | 1.9122E-03 | 0.9895 | PI(16:0/18:2) | FA(18:2) | -0.1064 | 2.4500E-06 | 1.7174E-04 | 0.9984 |
| TG(18:1/18:1/18:2) | 1-Hexadecanol | -0.0917 | 4.9800E-05 | 2.2626E-03 | 0.9865 | PI(16:0/18:3) | PI(18:0/18:1) | -0.0925 | 4.2500E-05 | 1.9779E-03 | 0.9889 |
| TG(18:1/18:1/18:3) | Inositol | -0.0917 | 5.0000E-05 | 2.2693E-03 | 0.9865 | PI(16:0/18:3) | PI(18:2/18:2) | 0.4490 | 2.2200E-16 | 5.3200E-14 | 1.0000 |
| TG(16:0/18:3/18:3) | malonic acid | -0.0910 | 5.7000E-05 | 2.5276E-03 | 0.9865 | PI(18:0/18:1) | PI(18:1/18:1) | 0.3212 | 2.2200E-16 | 5.3200E-14 | 1.0000 |
| LPC(18:2) | D-Glyceric acid | 0.0920 | 4.6700E-05 | 2.1425E-03 | 0.9865 | PI(18:0/18:1) | PI(18:1/18:2) | 0.1013 | 7.3700E-06 | 4.5084E-04 | 0.9967 |
| TG(16:0/18:1/18:3) | 3-Methyl-2-oxobutanoic acid | 0.0929 | 3.9400E-05 | 1.8587E-03 | 0.9895 | PI(18:0/18:1) | PI(18:2/18:2) | -0.1171 | 2.1400E-07 | 1.9400E-05 | 0.9998 |
| TG(16:0/18:1/18:3) | oxoproline | 0.0947 | 2.7800E-05 | 1.3918E-03 | 0.9933 | PI(18:1/18:1) | PI(18:1/18:2) | 0.1966 | 2.2200E-16 | 5.3200E-14 | 1.0000 |
| TG(18:0/16:0/18:1) | L-Malic acid | 0.0959 | 2.1800E-05 | 1.1431E-03 | 0.9933 | PI(18:1/18:1) | PI(18:2/18:2) | 0.1101 | 1.0700E-06 | 8.2600E-05 | 0.9994 |
| LPC(18:2) | 2-Oxoadipate | 0.1003 | 9.0900E-06 | 5.4104E-04 | 0.9961 | PI(18:1/18:2) | PI(18:2/18:2) | 0.3101 | 2.2200E-16 | 5.3200E-14 | 1.0000 |
| TG(16:0/16:0/18:2) | 4-Aminobutyric acid(GABA) | 0.1014 | 7.2100E-06 | 4.4252E-04 | 0.9967 | SQDG(16:0/18:1) | SQDG(16:0/18:2) | -0.1007 | 8.2400E-06 | 4.9690E-04 | 0.9961 |
| TG(18:1/18:1/18:1) | lauric acid | 0.1014 | 7.1600E-06 | 4.3959E-04 | 0.9967 | SQDG(16:0/18:1) | SQDG(16:0/18:3) | -0.1740 | 9.7700E-15 | 2.1300E-12 | 1.0000 |
| TG(18:2/18:2/18:2) | Methyl jasmonate | 0.1035 | 4.6500E-06 | 3.0019E-04 | 0.9975 | SQDG(16:0/18:3) | FA(18:3) | 0.1034 | 4.6600E-06 | 3.0060E-04 | 0.9975 |
| TG(16:0/18:1/18:2) | lauric acid | 0.1075 | 1.9300E-06 | 1.3876E-04 | 0.9990 | SQDG(16:0/18:3) | FA(18:4) | 0.1140 | 4.4500E-07 | 3.7600E-05 | 0.9997 |
| TG(16:0/18:2/18:2) | fumaric acid | 0.1115 | 7.9600E-07 | 6.3600E-05 | 0.9994 | FA(13:0) | FA(14:1) | 0.1348 | 2.2700E-09 | 2.8400E-07 | 1.0000 |
| TG(16:0/18:2/18:2) | L-homoserine | 0.1131 | 5.4800E-07 | 4.5300E-05 | 0.9996 | FA(13:0) | FA(15:0) | 0.1550 | 5.8000E-12 | 1.0000E-09 | 1.0000 |
| DGDG(16:0/18:2) | DGDG(18:3/18:3) | -0.5977 | 2.2200E-16 | 5.3200E-14 | 1.0000 | FA(13:0) | FA(20:4) | 0.2158 | 2.2200E-16 | 5.3200E-14 | 1.0000 |
| PI(16:0/18:2) | PI(18:0/18:1) | -0.3690 | 2.2200E-16 | 5.3200E-14 | 1.0000 | FA(13:0) | FA(23:1) | 0.0997 | 1.0200E-05 | 5.9675E-04 | 0.9953 |
| LPE(16:0) | LPE(18:2) | -0.3067 | 2.2200E-16 | 5.3200E-14 | 1.0000 | FA(14:0) | FA(15:0) | 0.1335 | 3.1700E-09 | 3.8900E-07 | 1.0000 |
| TG(18:1/18:2/18:3) | TG(18:3/18:2/18:3) | -0.2866 | 2.2200E-16 | 5.3200E-14 | 1.0000 | FA(14:0) | FA(18:0) | 0.1301 | 8.0500E-09 | 9.3600E-07 | 1.0000 |
| CL(18:2/18:1/18:1/18:2) | CL(18:3/18:2/18:2/18:2) | -0.2796 | 2.2200E-16 | 5.3200E-14 | 1.0000 | FA(14:1) | FA(15:0) | 0.1188 | 1.4100E-07 | 1.3200E-05 | 0.9999 |
| PC(18:1/18:1) | PI(16:0/18:2) | -0.2729 | 2.2200E-16 | 5.3200E-14 | 1.0000 | FA(14:1) | FA(15:1) | 0.3405 | 2.2200E-16 | 5.3200E-14 | 1.0000 |
| LPC(16:0) | LPC(18:1) | -0.2689 | 2.2200E-16 | 5.3200E-14 | 1.0000 | FA(14:1) | FA(16:1) | 0.2828 | 2.2200E-16 | 5.3200E-14 | 1.0000 |
| LPE(16:0) | LPE(18:1) | -0.2526 | 2.2200E-16 | 5.3200E-14 | 1.0000 | FA(14:1) | FA(16:2) | 0.1295 | 9.4800E-09 | 1.0900E-06 | 1.0000 |
| TG(18:2/18:2/18:2) | TG(18:3/18:2/18:2) | -0.2487 | 2.2200E-16 | 5.3200E-14 | 1.0000 | FA(14:1) | FA(17:1) | 0.1197 | 1.1400E-07 | 1.0900E-05 | 0.9999 |
| PI(16:0/18:1) | PI(16:0/18:2) | -0.2466 | 2.2200E-16 | 5.3200E-14 | 1.0000 | FA(14:1) | 19-Hydroxyandrost-4-ene-3,17-dione | 0.1013 | 7.3500E-06 | 4.5007E-04 | 0.9967 |
| TG(18:1/18:2/18:3) | TG(18:3/18:2/18:2) | -0.2464 | 2.2200E-16 | 5.3200E-14 | 1.0000 | FA(15:0) | FA(16:1) | 0.1653 | 1.9700E-13 | 3.8900E-11 | 1.0000 |
| PI(16:0/18:1) | PI(18:2/18:2) | -0.2294 | 2.2200E-16 | 5.3200E-14 | 1.0000 | FA(15:0) | FA(17:0) | 0.1348 | 2.2200E-09 | 2.7800E-07 | 1.0000 |
| PI(16:0/18:1) | PI(18:1/18:1) | -0.2268 | 2.2200E-16 | 5.3200E-14 | 1.0000 | FA(15:0) | FA(17:1) | 0.1409 | 4.0700E-10 | 5.5700E-08 | 1.0000 |
| PI(16:0/18:2) | PI(16:0/18:3) | -0.2215 | 2.2200E-16 | 5.3200E-14 | 1.0000 | FA(15:1) | FA(16:1) | 0.2004 | 2.2200E-16 | 5.3200E-14 | 1.0000 |
| CL(18:2/18:1/18:1/18:2) | CL(18:2/18:2/18:2/18:2) | -0.2205 | 2.2200E-16 | 5.3200E-14 | 1.0000 | FA(15:1) | FA(16:2) | 0.1028 | 5.3000E-06 | 3.3693E-04 | 0.9967 |
| LPE(18:1) | LPE(18:2) | -0.2204 | 2.2200E-16 | 5.3200E-14 | 1.0000 | FA(15:1) | FA(17:1) | 0.1826 | 4.4400E-16 | 1.0500E-13 | 1.0000 |
| PC(16:0/18:1) | PI(16:0/18:2) | -0.2118 | 2.2200E-16 | 5.3200E-14 | 1.0000 | FA(15:4) | FA(16:4) | 0.4708 | 2.2200E-16 | 5.3200E-14 | 1.0000 |
| PC(18:1/18:2) | PI(16:0/18:2) | -0.2021 | 2.2200E-16 | 5.3200E-14 | 1.0000 | FA(15:4) | FA(23:1) | 0.1107 | 9.5100E-07 | 7.4400E-05 | 0.9994 |
| PG(16:0/18:2) | PG(18:0/18:1) | -0.1987 | 2.2200E-16 | 5.3200E-14 | 1.0000 | FA(16:0) | FA(17:2) | 0.1001 | 9.3300E-06 | 5.5342E-04 | 0.9961 |
| DG(16:0/18:3) | LPC(18:2) | -0.1963 | 2.2200E-16 | 5.3200E-14 | 1.0000 | FA(16:0) | FA(18:0) | 0.1420 | 2.9100E-10 | 4.0600E-08 | 1.0000 |
| TG(18:2/18:2/18:2) | TG(18:3/18:2/18:3) | -0.1788 | 1.7800E-15 | 4.1300E-13 | 1.0000 | FA(16:0) | FA(18:1) | 0.0914 | 5.2300E-05 | 2.3561E-03 | 0.9865 |
| PE(16:0/18:1) | PI(16:0/18:2) | -0.1778 | 2.4400E-15 | 5.6300E-13 | 1.0000 | FA(16:0) | FA(23:0) | 0.0944 | 2.9600E-05 | 1.4617E-03 | 0.9933 |
| PC(18:0/18:1) | PI(16:0/18:2) | -0.1754 | 5.7700E-15 | 1.2900E-12 | 1.0000 | FA(16:1) | FA(16:2) | 0.0947 | 2.7800E-05 | 1.3912E-03 | 0.9933 |
| SQDG(16:0/18:1) | SQDG(16:0/18:3) | -0.1740 | 9.7700E-15 | 2.1300E-12 | 1.0000 | FA(16:1) | FA(17:1) | 0.2351 | 2.2200E-16 | 5.3200E-14 | 1.0000 |
| TG(18:1/18:1/18:2) | TG(18:2/18:2/18:2) | -0.1731 | 1.3500E-14 | 2.9100E-12 | 1.0000 | FA(16:1) | FA(22:1) | 0.1027 | 5.4800E-06 | 3.4716E-04 | 0.9967 |
| PC(16:0/18:3) | PE(18:2/18:2) | -0.1697 | 4.4000E-14 | 9.1500E-12 | 1.0000 | FA(16:2) | FA(17:2) | 0.1666 | 1.3000E-13 | 2.5900E-11 | 1.0000 |
| LPC(18:0) | LPC(18:2) | -0.1693 | 5.0200E-14 | 1.0400E-11 | 1.0000 | FA(16:2) | 19-Hydroxyandrost-4-ene-3,17-dione | -0.0923 | 4.4600E-05 | 2.0628E-03 | 0.9865 |
| DG(18:1/18:1) | DG(20:0/18:2) | -0.1684 | 6.9700E-14 | 1.4300E-11 | 1.0000 | FA(16:4) | FA(18:0) | 0.1019 | 6.4400E-06 | 4.0111E-04 | 0.9967 |
| PC(16:0/18:2) | PI(16:0/18:2) | -0.1674 | 9.8400E-14 | 1.9900E-11 | 1.0000 | FA(16:4) | FA(20:2) | 0.1019 | 6.5200E-06 | 4.0509E-04 | 0.9967 |
| PE(16:0/18:2) | PI(16:0/18:2) | -0.1650 | 2.1900E-13 | 4.3200E-11 | 1.0000 | FA(17:0) | FA(23:1) | 0.1290 | 1.0800E-08 | 1.2300E-06 | 1.0000 |
| PI(16:0/18:2) | PI(18:1/18:2) | -0.1599 | 1.2000E-12 | 2.2300E-10 | 1.0000 | FA(17:1) | FA(19:1) | 0.1651 | 2.1400E-13 | 4.2200E-11 | 1.0000 |
| CL(18:2/18:2/18:2/18:1) | CL(18:3/18:2/18:2/18:2) | -0.1555 | 4.9500E-12 | 8.6300E-10 | 1.0000 | FA(17:2) | FA(18:2) | 0.3110 | 2.2200E-16 | 5.3200E-14 | 1.0000 |
| TG(16:0/18:2/18:2) | TG(18:2/18:2/18:2) | -0.1527 | 1.1700E-11 | 1.9700E-09 | 1.0000 | FA(17:2) | FA(19:1) | 0.0933 | 3.6600E-05 | 1.7518E-03 | 0.9895 |
| TG(16:0/18:1/18:2) | TG(18:1/18:2/18:3) | -0.1496 | 3.1100E-11 | 4.9800E-09 | 1.0000 | FA(17:2) | FA(23:0) | 0.0979 | 1.4800E-05 | 8.2322E-04 | 0.9940 |
| LPC(16:0) | LPC(18:2) | -0.1432 | 2.0700E-10 | 2.9600E-08 | 1.0000 | FA(17:6) | FA(18:4) | 0.1085 | 1.5400E-06 | 1.1407E-04 | 0.9991 |
| TG(16:0/16:0/18:3) | TG(20:1/18:1/18:2) | -0.1422 | 2.7600E-10 | 3.8800E-08 | 1.0000 | FA(17:6) | FA(21:6) | 0.2425 | 2.2200E-16 | 5.3200E-14 | 1.0000 |
| PC(18:0/18:1) | PC(18:2/18:2) | -0.1406 | 4.4700E-10 | 6.0500E-08 | 1.0000 | FA(17:6) | FA(28:4) | -0.1032 | 4.8600E-06 | 3.1219E-04 | 0.9975 |
| PC(18:1/18:2) | PE(18:0/18:1) | -0.1402 | 4.9100E-10 | 6.6300E-08 | 1.0000 | FA(17:6) | Aconitic Acid | 0.1040 | 4.1400E-06 | 2.7123E-04 | 0.9983 |
| PI(16:0/18:2) | PI(18:1/18:1) | -0.1388 | 7.4000E-10 | 9.8000E-08 | 1.0000 | FA(18:0) | FA(20:0) | 0.1461 | 8.7300E-11 | 1.3100E-08 | 1.0000 |
| DGDG(16:0/18:2) | DGDG(16:0/18:3) | -0.1377 | 1.0100E-09 | 1.3200E-07 | 1.0000 | FA(18:0) | FA(21:6) | 0.0926 | 4.2100E-05 | 1.9642E-03 | 0.9895 |
| TG(18:1/18:2/18:3) | DG(18:1/18:1) | -0.1371 | 1.2000E-09 | 1.5500E-07 | 1.0000 | FA(18:1) | FA(18:3) | -0.1159 | 2.8000E-07 | 2.4700E-05 | 0.9998 |
| PI(16:0/18:2) | FA(18:1) | -0.1364 | 1.4300E-09 | 1.8400E-07 | 1.0000 | FA(18:1) | FA(20:1) | 0.2623 | 2.2200E-16 | 5.3200E-14 | 1.0000 |
| LPC(16:0) | LPC(18:2) | -0.1364 | 1.4400E-09 | 1.8500E-07 | 1.0000 | FA(18:1) | FA(22:0) | 0.1308 | 6.5800E-09 | 7.7500E-07 | 1.0000 |
| PC(18:1/18:1) | PE(16:0/18:1) | -0.1358 | 1.7000E-09 | 2.1600E-07 | 1.0000 | FA(18:1) | FA(23:0) | 0.0935 | 3.5000E-05 | 1.6864E-03 | 0.9895 |
| TG(16:0/18:2/18:3) | LPC(18:2) | -0.1351 | 2.0400E-09 | 2.5700E-07 | 1.0000 | FA(18:1) | FA(29:0) | -0.1224 | 5.7900E-08 | 5.8100E-06 | 0.9999 |
| PC(18:1/18:2) | PI(16:0/18:1) | -0.1349 | 2.1800E-09 | 2.7300E-07 | 1.0000 | FA(18:1) | FA(30:0) | 0.0979 | 1.4600E-05 | 8.1177E-04 | 0.9940 |
| PE(18:1/18:1) | PI(16:0/18:2) | -0.1329 | 3.7400E-09 | 4.5500E-07 | 1.0000 | FA(18:2) | FA(18:3) | 0.2339 | 2.2200E-16 | 5.3200E-14 | 1.0000 |
| LPC(16:0) | LPE(18:1) | -0.1328 | 3.9200E-09 | 4.7500E-07 | 1.0000 | FA(18:2) | FA(20:2) | 0.1132 | 5.2700E-07 | 4.3800E-05 | 0.9996 |
| PC(16:0/18:2) | PE(18:3/18:3) | -0.1316 | 5.3600E-09 | 6.4000E-07 | 1.0000 | FA(18:2) | FA(21:6) | -0.1205 | 9.2000E-08 | 8.9200E-06 | 0.9999 |
| LPC(18:1) | LPC(18:2) | -0.1309 | 6.4200E-09 | 7.5700E-07 | 1.0000 | FA(18:3) | FA(18:4) | 0.1745 | 8.2200E-15 | 1.8100E-12 | 1.0000 |
| PC(18:1/18:2) | PE(18:1/18:1) | -0.1284 | 1.2500E-08 | 1.4000E-06 | 1.0000 | FA(18:3) | FA(20:2) | 0.1161 | 2.7000E-07 | 2.4000E-05 | 0.9998 |
| PE(18:2/18:2) | PG(16:0/16:0) | -0.1255 | 2.6300E-08 | 2.7800E-06 | 0.9999 | FA(18:3) | FA(20:3) | 0.3289 | 2.2200E-16 | 5.3200E-14 | 1.0000 |
| PE(18:1/18:1) | SQDG(16:0/18:2) | -0.1238 | 4.1000E-08 | 4.2200E-06 | 0.9999 | FA(18:4) | FA(20:1) | 0.0983 | 1.3700E-05 | 7.6962E-04 | 0.9949 |
| PC(18:1/18:1) | PI(16:0/18:1) | -0.1228 | 5.3100E-08 | 5.3600E-06 | 0.9999 | FA(18:4) | FA(20:2) | 0.1704 | 3.4400E-14 | 7.2300E-12 | 1.0000 |
| PC(18:2/18:2) | PI(16:0/18:2) | -0.1206 | 8.9900E-08 | 8.7300E-06 | 0.9999 | FA(18:4) | FA(20:3) | 0.1284 | 1.2500E-08 | 1.4100E-06 | 1.0000 |
| PC(18:0/18:1) | PI(16:0/18:1) | -0.1193 | 1.2500E-07 | 1.1800E-05 | 0.9999 | FA(19:0) | FA(21:0) | 0.1467 | 7.3000E-11 | 1.1100E-08 | 1.0000 |
| PC(18:1/18:2) | PE(16:0/18:3) | -0.1191 | 1.3100E-07 | 1.2400E-05 | 0.9999 | FA(19:0) | FA(23:1) | 0.2261 | 2.2200E-16 | 5.3200E-14 | 1.0000 |
| TG(16:0/18:1/18:1) | TG(18:2/18:2/18:2) | -0.1179 | 1.7500E-07 | 1.6100E-05 | 0.9998 | FA(19:1) | FA(20:1) | 0.1128 | 5.9000E-07 | 4.8400E-05 | 0.9994 |
| TG(18:0/18:1/18:1) | TG(20:1/18:1/18:2) | -0.1174 | 1.9600E-07 | 1.7900E-05 | 0.9998 | FA(19:1) | FA(23:1) | -0.0921 | 4.5700E-05 | 2.1059E-03 | 0.9865 |
| PI(18:0/18:1) | PI(18:2/18:2) | -0.1171 | 2.1400E-07 | 1.9400E-05 | 0.9998 | FA(20:0) | FA(20:1) | 0.0976 | 1.5700E-05 | 8.6570E-04 | 0.9940 |
| LPC(18:2) | PG(16:0/18:2) | -0.1165 | 2.4600E-07 | 2.2000E-05 | 0.9998 | FA(20:0) | FA(21:0) | 0.0956 | 2.3100E-05 | 1.2004E-03 | 0.9933 |
| FA(18:1) | FA(18:3) | -0.1159 | 2.8000E-07 | 2.4700E-05 | 0.9998 | FA(20:0) | FA(22:0) | 0.1705 | 3.4000E-14 | 7.1400E-12 | 1.0000 |
| PE(18:0/18:1) | PG(16:0/18:1) | -0.1150 | 3.5300E-07 | 3.0600E-05 | 0.9997 | FA(20:0) | FA(24:0) | 0.1117 | 7.4800E-07 | 6.0100E-05 | 0.9994 |
| TG(18:2/18:2/18:2) | TG(18:3/18:2/18:2) | -0.1134 | 5.1400E-07 | 4.2800E-05 | 0.9996 | FA(20:1) | FA(20:2) | 0.1042 | 3.9600E-06 | 2.6087E-04 | 0.9983 |
| PC(18:1/18:1) | PI(18:1/18:1) | -0.1131 | 5.4600E-07 | 4.5100E-05 | 0.9996 | FA(20:2) | FA(20:3) | 0.1548 | 6.1600E-12 | 1.0600E-09 | 1.0000 |
| PE(16:0/18:1) | PG(18:0/18:2) | -0.1116 | 7.7100E-07 | 6.1800E-05 | 0.9994 | FA(20:4) | FA(21:0) | 0.0926 | 4.1500E-05 | 1.9416E-03 | 0.9895 |
| PE(18:2/18:2) | PI(16:0/18:2) | -0.1111 | 8.6600E-07 | 6.8500E-05 | 0.9994 | FA(20:4) | FA(23:1) | 0.2203 | 2.2200E-16 | 5.3200E-14 | 1.0000 |
| TG(18:3/18:2/18:2) | TG(18:3/18:3/18:3) | -0.1110 | 8.7600E-07 | 6.9200E-05 | 0.9994 | FA(20:4) | 3-Hydroxyphenylacetic acid | -0.1054 | 3.0500E-06 | 2.0841E-04 | 0.9983 |
| DG(16:0/18:3) | SQDG(16:0/18:3) | -0.1098 | 1.1500E-06 | 8.8200E-05 | 0.9991 | FA(20:4) | serine | -0.1040 | 4.1100E-06 | 2.6951E-04 | 0.9983 |
| TG(18:1/18:1/18:1) | TG(18:1/18:2/18:3) | -0.1096 | 1.2100E-06 | 9.1800E-05 | 0.9991 | FA(20:6) | FA(21:6) | 0.3699 | 2.2200E-16 | 5.3200E-14 | 1.0000 |
| TG(18:3/18:2/18:2) | DG(18:1/18:1) | -0.1090 | 1.3800E-06 | 1.0362E-04 | 0.9991 | FA(21:0) | FA(22:0) | 0.1583 | 1.9900E-12 | 3.6100E-10 | 1.0000 |
| PE(16:0/18:2) | PE(18:1/18:2) | -0.1086 | 1.5200E-06 | 1.1237E-04 | 0.9991 | FA(21:0) | FA(23:1) | 0.3361 | 2.2200E-16 | 5.3200E-14 | 1.0000 |
| PC(18:2/18:2) | PE(16:0/18:1) | -0.1082 | 1.6400E-06 | 1.2046E-04 | 0.9990 | FA(21:6) | FA(28:4) | 0.0911 | 5.5500E-05 | 2.4751E-03 | 0.9865 |
| TG(18:2/18:2/18:2) | DG(18:1/18:1) | -0.1080 | 1.7500E-06 | 1.2739E-04 | 0.9990 | FA(21:6) | FA(29:0) | 0.0967 | 1.8600E-05 | 1.0023E-03 | 0.9933 |
| TG(18:3/18:2/18:2) | TG(20:1/18:3/18:3) | -0.1079 | 1.7700E-06 | 1.2893E-04 | 0.9990 | FA(21:6) | FA(30:0) | -0.1088 | 1.4400E-06 | 1.0729E-04 | 0.9991 |
| PC(16:0/18:2) | PE(18:1/18:2) | -0.1079 | 1.7700E-06 | 1.2905E-04 | 0.9990 | FA(22:0) | FA(23:0) | 0.2253 | 2.2200E-16 | 5.3200E-14 | 1.0000 |
| TG(16:0/18:2/18:3) | TG(20:1/18:1/18:2) | -0.1074 | 1.9800E-06 | 1.4199E-04 | 0.9990 | FA(22:0) | FA(23:1) | 0.1204 | 9.4400E-08 | 9.1400E-06 | 0.9999 |
| PC(16:0/18:1) | PI(16:0/18:1) | -0.1071 | 2.1200E-06 | 1.5052E-04 | 0.9984 | FA(22:0) | FA(24:0) | 0.1558 | 4.4500E-12 | 7.8100E-10 | 1.0000 |
| TG(18:1/18:1/18:3) | TG(18:3/18:2/18:2) | -0.1066 | 2.3400E-06 | 1.6470E-04 | 0.9984 | FA(22:1) | FA(27:0) | 0.1491 | 3.6100E-11 | 5.7200E-09 | 1.0000 |
| TG(16:0/18:1/18:3) | TG(18:2/18:2/18:2) | -0.1064 | 2.4500E-06 | 1.7153E-04 | 0.9984 | FA(23:0) | FA(24:0) | 0.2885 | 2.2200E-16 | 5.3200E-14 | 1.0000 |
| PI(16:0/18:2) | FA(18:2) | -0.1064 | 2.4500E-06 | 1.7174E-04 | 0.9984 | FA(23:0) | FA(25:0) | 0.2003 | 2.2200E-16 | 5.3200E-14 | 1.0000 |
| TG(18:1/18:2/18:2) | TG(18:3/18:2/18:2) | -0.1062 | 2.5700E-06 | 1.7876E-04 | 0.9984 | FA(24:0) | FA(25:0) | 0.2674 | 2.2200E-16 | 5.3200E-14 | 1.0000 |
| TG(18:2/18:2/18:2) | TG(18:3/18:2/18:2) | -0.1061 | 2.6400E-06 | 1.8328E-04 | 0.9984 | FA(24:0) | FA(26:0) | 0.1373 | 1.1300E-09 | 1.4700E-07 | 1.0000 |
| TG(20:1/18:1/18:2) | Cer(d18:2/16:0) | -0.1060 | 2.6900E-06 | 1.8650E-04 | 0.9984 | FA(25:0) | FA(26:0) | 0.1562 | 3.9500E-12 | 6.9800E-10 | 1.0000 |
| PC(18:0/18:1) | PE(18:1/18:1) | -0.1056 | 2.9200E-06 | 2.0072E-04 | 0.9983 | FA(25:0) | FA(27:0) | 0.1391 | 6.7600E-10 | 8.9800E-08 | 1.0000 |
| LPC(18:1) | LPC(18:2) | -0.1053 | 3.1600E-06 | 2.1464E-04 | 0.9983 | FA(26:0) | FA(27:0) | 0.2151 | 2.2200E-16 | 5.3200E-14 | 1.0000 |
| TG(16:0/18:2/18:3) | LPC(18:1) | -0.1050 | 3.3600E-06 | 2.2666E-04 | 0.9983 | FA(26:0) | FA(28:0) | 0.2276 | 2.2200E-16 | 5.3200E-14 | 1.0000 |
| PC(16:0/18:1) | PE(18:0/18:1) | -0.1045 | 3.7100E-06 | 2.4709E-04 | 0.9983 | FA(27:0) | FA(28:0) | 0.2946 | 2.2200E-16 | 5.3200E-14 | 1.0000 |
| PC(16:0/18:2) | PC(18:0/18:1) | -0.1040 | 4.1100E-06 | 2.6914E-04 | 0.9983 | FA(27:0) | FA(29:0) | 0.1871 | 2.2200E-16 | 5.3200E-14 | 1.0000 |
| DG(18:1/18:1) | Cer(d18:2/16:0) | -0.1020 | 6.2900E-06 | 3.9247E-04 | 0.9967 | FA(27:0) | FA(30:0) | -0.1262 | 2.2000E-08 | 2.3600E-06 | 1.0000 |
| SQDG(16:0/18:1) | SQDG(16:0/18:2) | -0.1007 | 8.2400E-06 | 4.9690E-04 | 0.9961 | FA(28:0) | FA(29:0) | 0.2565 | 2.2200E-16 | 5.3200E-14 | 1.0000 |
| TG(18:1/18:1/18:3) | TG(18:2/18:2/18:2) | -0.1000 | 9.5900E-06 | 5.6653E-04 | 0.9961 | FA(28:0) | FA(30:0) | 0.1305 | 7.1400E-09 | 8.3600E-07 | 1.0000 |
| DG(18:0/18:1) | PE(16:0/18:1) | -0.0999 | 9.7000E-06 | 5.7214E-04 | 0.9961 | FA(28:4) | L-kynurenine | 0.1080 | 1.7400E-06 | 1.2683E-04 | 0.9990 |
| PE(18:3/18:3) | SQDG(16:0/18:3) | -0.0997 | 1.0100E-05 | 5.9373E-04 | 0.9953 | FA(28:4) | Inositol | -0.0974 | 1.6200E-05 | 8.9020E-04 | 0.9940 |
| TG(18:2/18:2/18:2) | TG(18:3/18:3/18:3) | -0.0994 | 1.0800E-05 | 6.2727E-04 | 0.9953 | FA(28:4) | squalene | 0.0977 | 1.5200E-05 | 8.4348E-04 | 0.9940 |
| DG(20:0/18:3) | LPC(18:1) | -0.0982 | 1.3800E-05 | 7.7371E-04 | 0.9947 | FA(29:0) | FA(30:0) | 0.5897 | 2.2200E-16 | 5.3200E-14 | 1.0000 |
| DG(18:1/18:1) | LPC(18:2) | -0.0981 | 1.4100E-05 | 7.8788E-04 | 0.9947 | FA(30:0) | 19-Hydroxyandrost-4-ene-3,17-dione | 0.0950 | 2.6000E-05 | 1.3208E-03 | 0.9933 |
| PE(18:0/18:1) | PI(16:0/18:1) | -0.0974 | 1.6200E-05 | 8.8729E-04 | 0.9940 | FA(30:0) | dibenzofuran | 0.1189 | 1.3800E-07 | 1.3000E-05 | 0.9999 |
| LPC(18:0) | LPE(18:2) | -0.0974 | 1.6300E-05 | 8.9354E-04 | 0.9940 | β-Sitosterol | 2-Aminophenol | 0.2032 | 2.2200E-16 | 5.3200E-14 | 1.0000 |
| TG(18:1/18:1/18:1) | TG(18:3/18:2/18:3) | -0.0970 | 1.7700E-05 | 9.5721E-04 | 0.9933 | N-Methylhydantoin | 2-Aminophenol | 0.0964 | 1.9700E-05 | 1.0511E-03 | 0.9933 |
| DG(16:0/18:3) | DG(18:1/18:1) | -0.0946 | 2.8300E-05 | 1.4107E-03 | 0.9933 | L-2,4-Diaminobutanoate | 2-Aminophenol | -0.1595 | 1.3800E-12 | 2.5500E-10 | 1.0000 |
| TG(16:0/18:1/18:2) | TG(18:0/18:0/18:1) | -0.0944 | 2.9600E-05 | 1.4635E-03 | 0.9933 | N-Methylhydantoin | 2-Oxoadipate | 0.1329 | 3.7800E-09 | 4.6000E-07 | 1.0000 |
| PE(18:1/18:1) | PG(16:0/18:2) | -0.0934 | 3.5800E-05 | 1.7185E-03 | 0.9895 | N-Methylhydantoin | 3-Methyl-2-oxobutanoic acid | 0.1026 | 5.5700E-06 | 3.5224E-04 | 0.9967 |
| TG(18:1/18:2/18:2) | TG(18:2/18:2/18:2) | -0.0931 | 3.7800E-05 | 1.7985E-03 | 0.9895 | L-2,4-Diaminobutanoate | 3-Methyl-2-oxobutanoic acid | 0.2322 | 2.2200E-16 | 5.3200E-14 | 1.0000 |
| PE(18:1/18:2) | PG(18:0/18:1) | -0.0930 | 3.8800E-05 | 1.8388E-03 | 0.9895 | 3-hydroxybutyric acid | 3-hydroxy-L-proline | 0.1386 | 7.8700E-10 | 1.0400E-07 | 1.0000 |
| DG(20:0/18:2) | PG(18:0/18:1) | -0.0929 | 3.9600E-05 | 1.8672E-03 | 0.9895 | 3-hydroxy-L-proline | 3-Hydroxyphenylacetic acid | 0.1241 | 3.8200E-08 | 3.9500E-06 | 0.9999 |
| PC(16:0/18:3) | FA(18:3) | -0.0929 | 3.9800E-05 | 1.8746E-03 | 0.9895 | 2-Hydroxybutanoic acid | 4-Acetamidobutanoate | 0.1091 | 1.3700E-06 | 1.0259E-04 | 0.9991 |
| PE(16:0/18:1) | PI(18:0/18:1) | -0.0926 | 4.1700E-05 | 1.9504E-03 | 0.9895 | 3-Hydroxypropionic acid | 4-Acetamidobutanoate | 0.3770 | 2.2200E-16 | 5.3200E-14 | 1.0000 |
| TG(16:0/18:1/18:3) | TG(18:1/18:1/18:2) | -0.0926 | 4.1900E-05 | 1.9555E-03 | 0.9895 | 4-Acetamidobutanoate | 19-Hydroxyandrost-4-ene-3,17-dione | 0.0969 | 1.7800E-05 | 9.6437E-04 | 0.9933 |
| DG(16:0/18:2) | DG(20:0/18:2) | -0.0926 | 4.2100E-05 | 1.9645E-03 | 0.9895 | 3-Hydroxypropionic acid | 4-Hydroxybenzoic acid | 0.1274 | 1.6100E-08 | 1.7700E-06 | 1.0000 |
| PI(16:0/18:3) | PI(18:0/18:1) | -0.0925 | 4.2500E-05 | 1.9779E-03 | 0.9889 | 2-Oxoadipate | 4-Hydroxyphenylethanol | 0.1415 | 3.4000E-10 | 4.7100E-08 | 1.0000 |
| PE(18:2/18:2) | FA(18:3) | -0.0924 | 4.3600E-05 | 2.0231E-03 | 0.9865 | 3-Methylcatechol | 5-Amino-4-imidazolecarboxyamide | -0.1073 | 2.0200E-06 | 1.4475E-04 | 0.9990 |
| DGDG(16:0/18:3) | DGDG(18:3/18:3) | -0.0924 | 4.3700E-05 | 2.0274E-03 | 0.9865 | 3-Methylcatechol | 5-aminovaleric acid lactam | 0.0959 | 2.1800E-05 | 1.1433E-03 | 0.9933 |
| PC(16:0/18:3) | PE(16:0/18:2) | -0.0919 | 4.7900E-05 | 2.1889E-03 | 0.9865 | 3-Hydroxypropionic acid | 6-Hydroxynicotinic acid | 0.1035 | 4.6100E-06 | 2.9761E-04 | 0.9975 |
| PE(18:1/18:2) | PG(16:0/18:1) | -0.0919 | 4.8100E-05 | 2.1975E-03 | 0.9865 | 4-Aminobutyric acid(GABA) | 6-Hydroxynicotinic acid | 0.1246 | 3.3600E-08 | 3.5100E-06 | 0.9999 |
| PE(16:0/18:1) | PI(18:1/18:1) | -0.0915 | 5.1200E-05 | 2.3133E-03 | 0.9865 | 3-hydroxy-L-proline | 6-Phospho-D-gluconate | 0.1217 | 6.9700E-08 | 6.8800E-06 | 0.9999 |
| TG(16:0/18:3/18:3) | TG(18:1/18:1/18:2) | -0.0914 | 5.2100E-05 | 2.3501E-03 | 0.9865 | 3-Hydroxyphenylacetic acid | 6-Phospho-D-gluconate | 0.1111 | 8.5600E-07 | 6.7800E-05 | 0.9994 |
| TG(18:0/16:0/18:1) | TG(18:1/18:1/18:1) | -0.0908 | 5.9000E-05 | 2.5987E-03 | 0.9833 | 4-Acetamidobutanoate | 6-Phospho-D-gluconate | 0.1257 | 2.4900E-08 | 2.6400E-06 | 1.0000 |
| PC(18:0/18:1) | PE(18:1/18:2) | -0.0907 | 5.9700E-05 | 2.6235E-03 | 0.9833 | 6-Hydroxynicotinic acid | 6-Phospho-D-gluconate | 0.0932 | 3.6900E-05 | 1.7631E-03 | 0.9895 |
| Cer(d18:2/16:0) | PG(16:0/16:0) | 0.0909 | 5.8000E-05 | 2.5635E-03 | 0.9865 | 1-Hexadecanol | 9-Fluorenone | 0.1591 | 1.5500E-12 | 2.8400E-10 | 1.0000 |
| PE(16:0/18:1) | PE(20:0/18:1) | 0.0910 | 5.7000E-05 | 2.5259E-03 | 0.9865 | 2-Aminophenol | 9-Fluorenone | 0.1538 | 8.5000E-12 | 1.4400E-09 | 1.0000 |
| DGDG(16:0/18:3) | LPE(18:1) | 0.0910 | 5.6500E-05 | 2.5106E-03 | 0.9865 | 3-Methyl-2-oxobutanoic acid | 9-Fluorenone | 0.1406 | 4.4100E-10 | 5.9900E-08 | 1.0000 |
| FA(16:0) | FA(18:1) | 0.0914 | 5.2300E-05 | 2.3561E-03 | 0.9865 | 4-Hydroxyphenylethanol | 9-Fluorenone | 0.0981 | 1.4200E-05 | 7.9383E-04 | 0.9940 |
| LPC(18:1) | LPE(18:2) | 0.0915 | 5.1600E-05 | 2.3303E-03 | 0.9865 | 1-Hexadecanol | Abietic Acid | -0.1106 | 9.6700E-07 | 7.5400E-05 | 0.9994 |
| CL(18:2/18:2/18:2/18:2) | LPC(18:1) | 0.0915 | 5.1100E-05 | 2.3121E-03 | 0.9865 | N-Methylhydantoin | Abietic Acid | -0.1041 | 4.0800E-06 | 2.6794E-04 | 0.9983 |
| TG(18:1/18:1/18:2) | TG(20:2/18:2/18:2) | 0.0917 | 4.9400E-05 | 2.2478E-03 | 0.9865 | 6-Hydroxynicotinic acid | Abietic Acid | 0.1137 | 4.7900E-07 | 4.0200E-05 | 0.9996 |
| DG(16:0/18:1) | PI(16:0/18:1) | 0.0920 | 4.7200E-05 | 2.1618E-03 | 0.9865 | 1-Hexadecanol | Acetol | 0.1338 | 2.9600E-09 | 3.6400E-07 | 1.0000 |
| PE(18:1/18:1) | PG(16:0/18:1) | 0.0932 | 3.7100E-05 | 1.7694E-03 | 0.9895 | 3-hydroxy-L-proline | Acetol | -0.1032 | 4.9200E-06 | 3.1542E-04 | 0.9975 |
| DG(20:0/18:2) | PE(18:2/18:2) | 0.0933 | 3.6500E-05 | 1.7479E-03 | 0.9895 | 5-Amino-4-imidazolecarboxyamide | Acetol | 0.2106 | 2.2200E-16 | 5.3200E-14 | 1.0000 |
| TG(16:0/16:0/18:2) | TG(16:0/18:1/18:3) | 0.0935 | 3.5300E-05 | 1.6968E-03 | 0.9895 | Acetol | adenine | 0.1456 | 1.0200E-10 | 1.5100E-08 | 1.0000 |
| TG(16:0/18:1/18:3) | TG(18:1/18:1/18:3) | 0.0937 | 3.4100E-05 | 1.6487E-03 | 0.9895 | 3-Hydroxypropionic acid | alanine | 0.1471 | 6.4900E-11 | 9.9800E-09 | 1.0000 |
| CL(18:2/18:2/18:2/18:2) | LPC(16:0) | 0.0938 | 3.2900E-05 | 1.5993E-03 | 0.9895 | 4-Acetamidobutanoate | alanine | 0.2183 | 2.2200E-16 | 5.3200E-14 | 1.0000 |
| TG(18:1/18:1/18:3) | PI(16:0/18:3) | 0.0941 | 3.1500E-05 | 1.5440E-03 | 0.9895 | 4-Acetamidobutanoate | alpha-D-glucosamine 1-phosphate | 0.1464 | 8.1300E-11 | 1.2300E-08 | 1.0000 |
| PE(18:0/18:1) | PE(20:0/18:2) | 0.0942 | 3.0600E-05 | 1.5044E-03 | 0.9895 | 2-Oxoadipate | alpha-ketoisocaproic acid | 0.1166 | 2.4000E-07 | 2.1500E-05 | 0.9998 |
| DG(18:0/18:1) | PC(18:0/18:1) | 0.0944 | 2.9300E-05 | 1.4497E-03 | 0.9933 | 2-Oxobutyric acid | alpha-ketoisocaproic acid | 0.1212 | 7.7300E-08 | 7.5800E-06 | 0.9999 |
| TG(16:0/18:1/18:3) | TG(18:0/18:0/18:1) | 0.0946 | 2.8200E-05 | 1.4076E-03 | 0.9933 | 3-hydroxy-L-proline | alpha-ketoisocaproic acid | 0.1002 | 9.2000E-06 | 5.4645E-04 | 0.9961 |
| OAHFA(18:1/18:0) | FA(18:1) | 0.0946 | 2.8200E-05 | 1.4074E-03 | 0.9933 | 6-Hydroxynicotinic acid | alpha-ketoisocaproic acid | 0.0921 | 4.5700E-05 | 2.1040E-03 | 0.9865 |
| TG(18:1/18:1/18:2) | TG(18:1/18:1/18:3) | 0.0947 | 2.7800E-05 | 1.3920E-03 | 0.9933 | 2-Oxoadipate | Arachidic acid | -0.0928 | 4.0400E-05 | 1.9006E-03 | 0.9895 |
| TG(16:0/18:1/18:2) | TG(18:0/18:1/18:1) | 0.0950 | 2.6400E-05 | 1.3377E-03 | 0.9933 | 2-Aminophenol | Atrazine-2-hydroxy | -0.0963 | 2.0500E-05 | 1.0843E-03 | 0.9933 |
| DG(18:3/18:2) | DG(20:1/18:3) | 0.0950 | 2.6300E-05 | 1.3316E-03 | 0.9933 | 4-Acetamidobutanoate | Atrazine-2-hydroxy | 0.1222 | 6.1400E-08 | 6.1200E-06 | 0.9999 |
| TG(20:0/18:1/18:2) | TG(20:1/18:1/18:2) | 0.0950 | 2.6100E-05 | 1.3233E-03 | 0.9933 | 4-Hydroxybenzoic acid | Atrazine-2-hydroxy | 0.1257 | 2.5400E-08 | 2.6900E-06 | 0.9999 |
| CL(18:2/18:2/18:2/18:1) | DGDG(16:0/18:3) | 0.0951 | 2.5600E-05 | 1.3029E-03 | 0.9933 | 4-Hydroxyphenylethanol | Atrazine-2-hydroxy | 0.1552 | 5.4400E-12 | 9.4300E-10 | 1.0000 |
| DGDG(16:0/18:3) | SQDG(16:0/18:1) | 0.0952 | 2.5000E-05 | 1.2799E-03 | 0.9933 | Acetol | Atrazine-2-hydroxy | -0.1044 | 3.7700E-06 | 2.5042E-04 | 0.9983 |
| OAHFA(18:1/18:0) | SQDG(16:0/18:2) | 0.0957 | 2.2800E-05 | 1.1839E-03 | 0.9933 | adenine | Atrazine-2-hydroxy | 0.1140 | 4.4100E-07 | 3.7300E-05 | 0.9997 |
| CL(18:2/18:1/18:1/18:2) | LPE(16:0) | 0.0958 | 2.2300E-05 | 1.1664E-03 | 0.9933 | 3-Phenylcatechol | Atropine | 0.1239 | 4.0300E-08 | 4.1600E-06 | 0.9999 |
| DG(18:3/18:2) | DG(18:3/18:3) | 0.0960 | 2.1400E-05 | 1.1251E-03 | 0.9933 | 4-Hydroxyphenylethanol | Atropine | 0.0995 | 1.0600E-05 | 6.1704E-04 | 0.9953 |
| DG(18:2/18:2) | DG(20:1/18:2) | 0.0965 | 1.9300E-05 | 1.0330E-03 | 0.9933 | 5-Amino-4-imidazolecarboxyamide | Atropine | 0.1012 | 7.4800E-06 | 4.5683E-04 | 0.9967 |
| DGDG(16:0/18:2) | SQDG(16:0/18:2) | 0.0966 | 1.8900E-05 | 1.0146E-03 | 0.9933 | alanine | Atropine | -0.0916 | 5.0400E-05 | 2.2857E-03 | 0.9865 |
| FA(20:0) | FA(20:1) | 0.0976 | 1.5700E-05 | 8.6570E-04 | 0.9940 | alpha-ketoisocaproic acid | Atropine | 0.1016 | 6.8700E-06 | 4.2421E-04 | 0.9967 |
| DG(16:0/18:1) | DG(18:1/18:2) | 0.0977 | 1.5300E-05 | 8.4629E-04 | 0.9940 | 4-Aminobutyric acid(GABA) | Behenic acid | 0.0945 | 2.8800E-05 | 1.4321E-03 | 0.9933 |
| CL(18:3/18:2/18:2/18:2) | LPE(18:1) | 0.0987 | 1.2500E-05 | 7.1269E-04 | 0.9953 | L-2,4-Diaminobutanoate | benzoic acid | 0.1072 | 2.0800E-06 | 1.4817E-04 | 0.9990 |
| TG(16:0/16:0/18:1) | TG(18:3/18:2/18:2) | 0.0987 | 1.2500E-05 | 7.0995E-04 | 0.9953 | 3-hydroxybutyric acid | benzoic acid | 0.1163 | 2.5900E-07 | 2.3100E-05 | 0.9998 |
| TG(16:0/16:0/18:2) | LPC(18:2) | 0.0988 | 1.2300E-05 | 7.0088E-04 | 0.9953 | 9-Fluorenone | benzoic acid | -0.0970 | 1.7700E-05 | 9.5653E-04 | 0.9933 |
| DGDG(18:3/18:3) | LPE(16:0) | 0.0990 | 1.1700E-05 | 6.7232E-04 | 0.9953 | Abietic Acid | benzoic acid | 0.1001 | 9.3900E-06 | 5.5634E-04 | 0.9961 |
| TG(16:0/16:0/18:1) | LPC(18:2) | 0.0991 | 1.1600E-05 | 6.6957E-04 | 0.9953 | N-Methylhydantoin | beta-Alanine | 0.0983 | 1.3500E-05 | 7.5925E-04 | 0.9949 |
| CL(18:3/18:2/18:2/18:2) | OAHFA(18:1/18:0) | 0.1002 | 9.1600E-06 | 5.4437E-04 | 0.9961 | 5-aminovaleric acid lactam | beta-Alanine | 0.0966 | 1.9000E-05 | 1.0203E-03 | 0.9933 |
| CL(18:2/18:2/18:2/18:1) | SQDG(16:0/18:1) | 0.1003 | 8.9800E-06 | 5.3540E-04 | 0.9961 | L-2,4-Diaminobutanoate | Biphenyl | -0.0910 | 5.6200E-05 | 2.4988E-03 | 0.9865 |
| CL(18:2/18:2/18:2/18:1) | OAHFA(18:1/18:0) | 0.1004 | 8.8900E-06 | 5.3053E-04 | 0.9961 | 3-Methylbenzyl Alcohol | Biphenyl | 0.1501 | 2.6800E-11 | 4.3300E-09 | 1.0000 |
| PE(18:2/18:2) | PE(18:3/18:2) | 0.1006 | 8.5400E-06 | 5.1247E-04 | 0.9961 | 9-Fluorenone | Biphenyl | 0.0964 | 1.9900E-05 | 1.0608E-03 | 0.9933 |
| CL(18:2/18:2/18:2/18:2) | SQDG(16:0/18:2) | 0.1007 | 8.2400E-06 | 4.9681E-04 | 0.9961 | Arachidic acid | Biphenyl | 0.1000 | 9.5600E-06 | 5.6515E-04 | 0.9961 |
| PC(18:2/18:2) | PE(18:3/18:2) | 0.1010 | 7.8200E-06 | 4.7492E-04 | 0.9961 | benzoic acid | Biphenyl | 0.2332 | 2.2200E-16 | 5.3200E-14 | 1.0000 |
| PI(18:0/18:1) | PI(18:1/18:2) | 0.1013 | 7.3700E-06 | 4.5084E-04 | 0.9967 | L-2,4-Diaminobutanoate | Biuret | 0.1111 | 8.6900E-07 | 6.8700E-05 | 0.9994 |
| TG(20:1/18:1/18:2) | TG(20:1/18:2/18:2) | 0.1014 | 7.1200E-06 | 4.3783E-04 | 0.9967 | 6-Hydroxynicotinic acid | Biuret | 0.1216 | 7.1100E-08 | 7.0000E-06 | 0.9999 |
| CL(18:3/18:2/18:2/18:2) | SQDG(16:0/18:3) | 0.1015 | 7.0400E-06 | 4.3325E-04 | 0.9967 | β-Sitosterol | Capric Acid | 0.1153 | 3.2600E-07 | 2.8400E-05 | 0.9997 |
| DGDG(16:0/18:2) | LPE(18:2) | 0.1019 | 6.5000E-06 | 4.0400E-04 | 0.9967 | L-2,4-Diaminobutanoate | Octanoic acid | -0.1226 | 5.4800E-08 | 5.5200E-06 | 0.9999 |
| DG(18:1/18:1) | DG(20:0/18:2) | 0.1019 | 6.4400E-06 | 4.0104E-04 | 0.9967 | 2-Hydroxybutanoic acid | Octanoic acid | 0.0926 | 4.1900E-05 | 1.9578E-03 | 0.9895 |
| TG(18:0/18:1/18:1) | TG(18:1/18:1/18:1) | 0.1022 | 6.0600E-06 | 3.7993E-04 | 0.9967 | 6-Hydroxynicotinic acid | Octanoic acid | 0.0959 | 2.1800E-05 | 1.1440E-03 | 0.9933 |
| DGDG(18:3/18:3) | OAHFA(18:1/18:0) | 0.1029 | 5.2000E-06 | 3.3132E-04 | 0.9967 | Abietic Acid | Octanoic acid | 0.1121 | 6.8700E-07 | 5.5700E-05 | 0.9994 |
| CL(18:2/18:2/18:2/18:2) | CL(18:3/18:2/18:2/18:2) | 0.1030 | 5.1600E-06 | 3.2894E-04 | 0.9967 | benzoic acid | Octanoic acid | 0.3146 | 2.2200E-16 | 5.3200E-14 | 1.0000 |
| TG(16:0/16:0/18:2) | TG(16:0/16:0/18:3) | 0.1032 | 4.8800E-06 | 3.1290E-04 | 0.9975 | 5-aminovaleric acid lactam | cholic acid | 0.0938 | 3.2900E-05 | 1.6024E-03 | 0.9895 |
| SQDG(16:0/18:3) | FA(18:3) | 0.1034 | 4.6600E-06 | 3.0060E-04 | 0.9975 | Aconitic Acid | cholic acid | 0.0976 | 1.5600E-05 | 8.5880E-04 | 0.9940 |
| LPE(18:1) | PG(18:0/18:1) | 0.1039 | 4.2000E-06 | 2.7433E-04 | 0.9977 | 4-Hydroxyphenylethanol | Icosenoic acid | 0.1171 | 2.1000E-07 | 1.9100E-05 | 0.9998 |
| LPC(16:0) | OAHFA(18:1/18:0) | 0.1041 | 4.0600E-06 | 2.6632E-04 | 0.9983 | Atrazine-2-hydroxy | citric acid | 0.0937 | 3.3800E-05 | 1.6394E-03 | 0.9895 |
| FA(20:1) | FA(20:2) | 0.1042 | 3.9600E-06 | 2.6087E-04 | 0.9983 | cholic acid | citric acid | -0.1182 | 1.6100E-07 | 1.4900E-05 | 0.9998 |
| CL(18:2/18:2/18:2/18:2) | DGDG(18:3/18:3) | 0.1043 | 3.9100E-06 | 2.5834E-04 | 0.9983 | alpha-ketoisocaproic acid | citrulline | -0.0944 | 2.9500E-05 | 1.4604E-03 | 0.9933 |
| DG(18:0/18:1) | PI(18:0/18:1) | 0.1047 | 3.5500E-06 | 2.3787E-04 | 0.9983 | cholic acid | citrulline | 0.3514 | 2.2200E-16 | 5.3200E-14 | 1.0000 |
| DG(18:2/18:2) | DG(18:3/18:2) | 0.1052 | 3.1900E-06 | 2.1646E-04 | 0.9983 | Icosenoic acid | citrulline | 0.1196 | 1.1700E-07 | 1.1200E-05 | 0.9999 |
| DGDG(18:3/18:3) | SQDG(16:0/18:1) | 0.1062 | 2.5600E-06 | 1.7859E-04 | 0.9984 | 2-Oxobutyric acid | coniferyl alcohol | 0.0959 | 2.1800E-05 | 1.1435E-03 | 0.9933 |
| LPE(18:2) | FA(18:2) | 0.1063 | 2.5100E-06 | 1.7508E-04 | 0.9984 | 3-Methyl-2-oxobutanoic acid | coniferyl alcohol | -0.0918 | 4.8700E-05 | 2.2221E-03 | 0.9865 |
| PC(18:1/18:1) | PC(18:1/18:2) | 0.1069 | 2.2100E-06 | 1.5618E-04 | 0.9984 | 3-Phenylcatechol | coniferyl alcohol | 0.1072 | 2.0500E-06 | 1.4662E-04 | 0.9990 |
| DGDG(16:0/18:2) | LPC(16:0) | 0.1071 | 2.1000E-06 | 1.4945E-04 | 0.9988 | 1-Hexadecanol | Creatine | 0.2122 | 2.2200E-16 | 5.3200E-14 | 1.0000 |
| PE(18:1/18:1) | SQDG(16:0/18:1) | 0.1072 | 2.0700E-06 | 1.4765E-04 | 0.9990 | 3-hydroxy-L-proline | Creatine | 0.1090 | 1.3800E-06 | 1.0372E-04 | 0.9991 |
| PE(16:0/18:2) | PE(18:3/18:2) | 0.1086 | 1.5200E-06 | 1.1265E-04 | 0.9991 | 6-Phospho-D-gluconate | Creatine | -0.0955 | 2.3800E-05 | 1.2281E-03 | 0.9933 |
| LPC(18:1) | SQDG(16:0/18:1) | 0.1092 | 1.3300E-06 | 9.9900E-05 | 0.9991 | Capric Acid | Creatine | 0.1143 | 4.1000E-07 | 3.5000E-05 | 0.9997 |
| PG(18:0/18:1) | PG(18:1/18:2) | 0.1099 | 1.1400E-06 | 8.6900E-05 | 0.9991 | alpha-D-glucosamine 1-phosphate | cycloserine | 0.1112 | 8.3600E-07 | 6.6400E-05 | 0.9994 |
| CL(18:2/18:2/18:2/18:2) | DGDG(16:0/18:2) | 0.1101 | 1.0900E-06 | 8.3800E-05 | 0.9991 | Creatine | cycloserine | 0.0920 | 4.6600E-05 | 2.1394E-03 | 0.9865 |
| PI(18:1/18:1) | PI(18:2/18:2) | 0.1101 | 1.0700E-06 | 8.2600E-05 | 0.9994 | 3-hydroxy-L-proline | Glyceryl 1-phosphate | 0.0985 | 1.3100E-05 | 7.4268E-04 | 0.9953 |
| TG(16:0/18:1/18:1) | TG(18:1/18:1/18:1) | 0.1106 | 9.7300E-07 | 7.5900E-05 | 0.9994 | 3-Hydroxyphenylacetic acid | Glyceryl 1-phosphate | 0.0905 | 6.2800E-05 | 2.7392E-03 | 0.9833 |
| TG(18:2/18:2/18:2) | LPC(18:1) | 0.1107 | 9.4200E-07 | 7.3800E-05 | 0.9994 | N-Methylhydantoin | D-Glyceric acid | 0.0946 | 2.8500E-05 | 1.4202E-03 | 0.9933 |
| CL(18:2/18:2/18:2/18:2) | SQDG(16:0/18:1) | 0.1108 | 9.1500E-07 | 7.1900E-05 | 0.9994 | 5-Aminovaleric acid | D-Glyceric acid | 0.1107 | 9.5100E-07 | 7.4300E-05 | 0.9994 |
| DG(20:0/18:2) | DG(20:1/18:2) | 0.1115 | 7.7900E-07 | 6.2400E-05 | 0.9994 | alpha-D-glucosamine 1-phosphate | D-Glyceric acid | 0.1563 | 3.8100E-12 | 6.7400E-10 | 1.0000 |
| DGDG(16:0/18:2) | SQDG(16:0/18:1) | 0.1117 | 7.4500E-07 | 5.9900E-05 | 0.9994 | alpha-D-glucosamine 1-phosphate | dibenzofuran | 0.1138 | 4.6100E-07 | 3.8800E-05 | 0.9997 |
| PE(16:0/18:3) | PE(18:3/18:2) | 0.1121 | 6.7900E-07 | 5.5100E-05 | 0.9994 | 9-Fluorenone | Ethanolamine | 0.1400 | 5.2600E-10 | 7.0700E-08 | 1.0000 |
| PG(18:0/16:0) | PG(18:0/18:2) | 0.1127 | 6.0200E-07 | 4.9400E-05 | 0.9994 | Cytidine-5'-monophosphate(CMP) | Ethanolamine | 0.5011 | 2.2200E-16 | 5.3200E-14 | 1.0000 |
| DG(18:0/18:1) | PG(18:0/18:1) | 0.1132 | 5.2900E-07 | 4.3900E-05 | 0.9996 | 3-Methylbenzyl Alcohol | Farnesal | 0.1426 | 2.4600E-10 | 3.4800E-08 | 1.0000 |
| FA(18:2) | FA(20:2) | 0.1132 | 5.2700E-07 | 4.3800E-05 | 0.9996 | 5-Amino-4-imidazolecarboxyamide | Farnesal | -0.1000 | 9.6600E-06 | 5.7012E-04 | 0.9961 |
| TG(16:0/16:0/18:1) | TG(16:0/18:1/18:2) | 0.1134 | 5.1200E-07 | 4.2700E-05 | 0.9996 | Capric Acid | Farnesal | 0.1855 | 2.2200E-16 | 5.3200E-14 | 1.0000 |
| CL(18:2/18:2/18:2/18:2) | OAHFA(18:1/18:0) | 0.1134 | 5.1200E-07 | 4.2600E-05 | 0.9996 | 3-Methyl-2-oxobutanoic acid | D-Fructose 2,6-bisphosphate | -0.0955 | 2.3900E-05 | 1.2322E-03 | 0.9933 |
| LPE(18:1) | SQDG(16:0/18:1) | 0.1138 | 4.5800E-07 | 3.8600E-05 | 0.9997 | 6-Phospho-D-gluconate | D-Fructose 2,6-bisphosphate | 0.1137 | 4.7600E-07 | 3.9900E-05 | 0.9997 |
| TG(16:0/18:1/18:2) | TG(20:1/18:2/18:2) | 0.1140 | 4.4600E-07 | 3.7700E-05 | 0.9997 | 3-Methylcatechol | fucose | 0.1184 | 1.5600E-07 | 1.4500E-05 | 0.9999 |
| LPE(16:0) | SQDG(16:0/18:2) | 0.1140 | 4.4200E-07 | 3.7400E-05 | 0.9997 | 9-Fluorenone | fucose | 0.1315 | 5.5500E-09 | 6.6200E-07 | 1.0000 |
| PC(16:0/18:1) | PC(18:1/18:1) | 0.1144 | 4.0100E-07 | 3.4300E-05 | 0.9997 | Biuret | fucose | 0.1314 | 5.6500E-09 | 6.7300E-07 | 1.0000 |
| DG(16:0/18:2) | DG(16:0/18:3) | 0.1146 | 3.8400E-07 | 3.3000E-05 | 0.9997 | Citraconic acid | fucose | 0.1082 | 1.6600E-06 | 1.2180E-04 | 0.9990 |
| DG(18:2/18:2) | DG(20:1/18:3) | 0.1158 | 2.8800E-07 | 2.5400E-05 | 0.9998 | coniferyl alcohol | fucose | 0.1239 | 3.9800E-08 | 4.1100E-06 | 0.9999 |
| TG(18:0/16:0/18:1) | LPC(18:2) | 0.1160 | 2.7900E-07 | 2.4700E-05 | 0.9998 | Glyceryl 1-phosphate | fucose | 0.1479 | 5.1700E-11 | 8.0500E-09 | 1.0000 |
| CL(18:3/18:2/18:2/18:2) | SQDG(16:0/18:2) | 0.1160 | 2.7400E-07 | 2.4300E-05 | 0.9998 | D-Glyceric acid | fucose | 0.2269 | 2.2200E-16 | 5.3200E-14 | 1.0000 |
| FA(18:3) | FA(20:2) | 0.1161 | 2.7000E-07 | 2.4000E-05 | 0.9998 | 1-Hexadecanol | fumaric acid | 0.1009 | 7.9400E-06 | 4.8112E-04 | 0.9961 |
| LPE(16:0) | SQDG(16:0/18:1) | 0.1167 | 2.3200E-07 | 2.0900E-05 | 0.9998 | 2-Aminophenol | fumaric acid | -0.1085 | 1.5500E-06 | 1.1472E-04 | 0.9990 |
| PG(16:0/18:2) | FA(18:3) | 0.1172 | 2.0700E-07 | 1.8900E-05 | 0.9998 | 3-Hydroxyphenylacetic acid | fumaric acid | -0.0956 | 2.3400E-05 | 1.2128E-03 | 0.9933 |
| DGDG(16:0/18:2) | LPE(16:0) | 0.1175 | 1.9200E-07 | 1.7500E-05 | 0.9998 | 3-Phenylcatechol | fumaric acid | 0.1139 | 4.5400E-07 | 3.8300E-05 | 0.9997 |
| DG(18:2/18:2) | DG(20:1/18:2) | 0.1176 | 1.9000E-07 | 1.7400E-05 | 0.9998 | Icosenoic acid | fumaric acid | 0.1466 | 7.5500E-11 | 1.1500E-08 | 1.0000 |
| PE(16:0/18:2) | PE(16:0/18:3) | 0.1186 | 1.4700E-07 | 1.3700E-05 | 0.9999 | citric acid | fumaric acid | 0.1204 | 9.5600E-08 | 9.2500E-06 | 0.9999 |
| PC(16:0/18:2) | PE(16:0/18:2) | 0.1198 | 1.1100E-07 | 1.0600E-05 | 0.9999 | Cytidine-5'-monophosphate(CMP) | fumaric acid | 0.2882 | 2.2200E-16 | 5.3200E-14 | 1.0000 |
| TG(18:0/16:0/18:1) | TG(20:0/18:1/18:2) | 0.1199 | 1.0700E-07 | 1.0300E-05 | 0.9999 | Ethanolamine | fumaric acid | 0.1049 | 3.4300E-06 | 2.3050E-04 | 0.9983 |
| TG(18:1/18:1/18:1) | TG(20:1/18:1/18:2) | 0.1220 | 6.4300E-08 | 6.3800E-06 | 0.9999 | 3-Phenylcatechol | gentisic acid | -0.1025 | 5.6300E-06 | 3.5601E-04 | 0.9967 |
| Cer(d18:2/16:0) | CerG1(d18:2/22:1) | 0.1222 | 6.0500E-08 | 6.0400E-06 | 0.9999 | 6-Hydroxynicotinic acid | gentisic acid | 0.1365 | 1.3900E-09 | 1.7900E-07 | 1.0000 |
| DG(18:2/18:2) | DG(18:3/18:2) | 0.1229 | 5.1200E-08 | 5.1900E-06 | 0.9999 | Creatine | gentisic acid | 0.1057 | 2.8400E-06 | 1.9583E-04 | 0.9983 |
| DG(16:0/18:3) | PE(18:3/18:3) | 0.1238 | 4.0700E-08 | 4.2000E-06 | 0.9999 | D-Glyceric acid | gentisic acid | 0.1571 | 2.9400E-12 | 5.2700E-10 | 1.0000 |
| TG(16:0/16:0/18:3) | TG(16:0/18:1/18:3) | 0.1242 | 3.6600E-08 | 3.8000E-06 | 0.9999 | 1-Hexadecanol | Geraniol | -0.1464 | 8.0600E-11 | 1.2200E-08 | 1.0000 |
| LPE(18:2) | FA(20:2) | 0.1257 | 2.5100E-08 | 2.6700E-06 | 1.0000 | 5-Amino-4-imidazolecarboxyamide | Geraniol | 0.0986 | 1.2700E-05 | 7.1958E-04 | 0.9953 |
| LPC(18:1) | SQDG(16:0/18:3) | 0.1258 | 2.4500E-08 | 2.6100E-06 | 1.0000 | Abietic Acid | Geraniol | 0.2131 | 2.2200E-16 | 5.3200E-14 | 1.0000 |
| TG(16:0/18:1/18:3) | TG(16:0/18:2/18:3) | 0.1264 | 2.1100E-08 | 2.2600E-06 | 1.0000 | Capric Acid | Geraniol | -0.1414 | 3.4700E-10 | 4.8000E-08 | 1.0000 |
| TG(18:1/18:1/18:1) | TG(18:1/18:1/18:2) | 0.1270 | 1.7900E-08 | 1.9500E-06 | 1.0000 | cholic acid | Geraniol | -0.0922 | 4.5100E-05 | 2.0812E-03 | 0.9865 |
| CL(18:2/18:1/18:1/18:2) | SQDG(16:0/18:1) | 0.1270 | 1.7800E-08 | 1.9400E-06 | 1.0000 | Icosenoic acid | Geraniol | 0.0939 | 3.2700E-05 | 1.5928E-03 | 0.9895 |
| DG(18:2/18:2) | DG(20:1/18:3) | 0.1273 | 1.6500E-08 | 1.8100E-06 | 1.0000 | Cytidine-5'-monophosphate(CMP) | Geraniol | 0.1566 | 3.5200E-12 | 6.2500E-10 | 1.0000 |
| PE(16:0/18:1) | PE(16:0/18:3) | 0.1275 | 1.5700E-08 | 1.7300E-06 | 1.0000 | fumaric acid | Geraniol | 0.1420 | 2.9700E-10 | 4.1500E-08 | 1.0000 |
| DGDG(16:0/18:2) | OAHFA(18:1/18:0) | 0.1282 | 1.3000E-08 | 1.4600E-06 | 1.0000 | 3-hydroxybutyric acid | D-Glucose | 0.1110 | 8.8900E-07 | 7.0100E-05 | 0.9994 |
| OAHFA(18:1/18:0) | SQDG(16:0/18:1) | 0.1282 | 1.3000E-08 | 1.4600E-06 | 1.0000 | 3-Methylbenzyl Alcohol | D-Glucose | 0.2559 | 2.2200E-16 | 5.3200E-14 | 1.0000 |
| PG(16:0/16:0) | PG(16:0/18:1) | 0.1293 | 9.7900E-09 | 1.1200E-06 | 1.0000 | adenine | D-Glucose | 0.1271 | 1.7500E-08 | 1.9000E-06 | 1.0000 |
| TG(18:1/18:1/18:2) | TG(20:1/18:2/18:2) | 0.1294 | 9.7200E-09 | 1.1200E-06 | 1.0000 | Biphenyl | D-Glucose | 0.1166 | 2.4100E-07 | 2.1600E-05 | 0.9998 |
| PE(20:0/18:1) | PE(20:0/18:3) | 0.1302 | 7.7600E-09 | 9.0400E-07 | 1.0000 | Farnesal | D-Glucose | -0.0966 | 1.9200E-05 | 1.0258E-03 | 0.9933 |
| TG(20:1/18:2/18:2) | TG(20:2/18:2/18:2) | 0.1304 | 7.4200E-09 | 8.6700E-07 | 1.0000 | 3-Hydroxyphenylacetic acid | Glucose-1-phosphate | -0.0963 | 2.0100E-05 | 1.0689E-03 | 0.9933 |
| LPE(16:0) | OAHFA(18:1/18:0) | 0.1322 | 4.5000E-09 | 5.4300E-07 | 1.0000 | Phenyllactate | Glucose-1-phosphate | 0.1009 | 7.9800E-06 | 4.8301E-04 | 0.9961 |
| TG(16:0/18:1/18:2) | TG(18:1/18:1/18:3) | 0.1329 | 3.7600E-09 | 4.5700E-07 | 1.0000 | 6-Hydroxynicotinic acid | Glucose-1-phosphate | 0.1186 | 1.4700E-07 | 1.3800E-05 | 0.9999 |
| PE(16:0/18:1) | PE(16:0/18:2) | 0.1331 | 3.5500E-09 | 4.3300E-07 | 1.0000 | benzoic acid | Glucose-1-phosphate | -0.0954 | 2.4200E-05 | 1.2438E-03 | 0.9933 |
| CL(18:2/18:2/18:2/18:2) | LPE(16:0) | 0.1337 | 3.0600E-09 | 3.7600E-07 | 1.0000 | Creatine | Glucose-1-phosphate | -0.1041 | 4.0800E-06 | 2.6747E-04 | 0.9983 |
| CL(18:3/18:2/18:2/18:2) | LPE(18:2) | 0.1344 | 2.5100E-09 | 3.1200E-07 | 1.0000 | benzoic acid | Glucose-6-phosphate | -0.0945 | 2.9100E-05 | 1.4451E-03 | 0.9933 |
| PC(18:1/18:2) | PC(18:2/18:2) | 0.1355 | 1.8700E-09 | 2.3600E-07 | 1.0000 | Biphenyl | Glucose-6-phosphate | 0.1406 | 4.4600E-10 | 6.0500E-08 | 1.0000 |
| DG(18:1/18:1) | DG(18:3/18:3) | 0.1362 | 1.5100E-09 | 1.9300E-07 | 1.0000 | Capric Acid | Glucose-6-phosphate | 0.0973 | 1.6500E-05 | 9.0044E-04 | 0.9933 |
| DG(18:1/18:2) | DG(20:1/18:2) | 0.1392 | 6.5700E-10 | 8.7400E-08 | 1.0000 | cholic acid | Glucose-6-phosphate | 0.1089 | 1.4200E-06 | 1.0595E-04 | 0.9991 |
| DG(18:1/18:1) | LPC(18:1) | 0.1395 | 6.0700E-10 | 8.1100E-08 | 1.0000 | 3-hydroxybutyric acid | glutamine | 0.0964 | 1.9800E-05 | 1.0552E-03 | 0.9933 |
| TG(18:0/16:0/18:1) | LPC(18:1) | 0.1399 | 5.3600E-10 | 7.2000E-08 | 1.0000 | 6-Phospho-D-gluconate | glutamine | -0.0919 | 4.7400E-05 | 2.1697E-03 | 0.9865 |
| DG(18:1/18:2) | DG(20:1/18:2) | 0.1402 | 4.9700E-10 | 6.7000E-08 | 1.0000 | Biuret | glutamine | 0.1274 | 1.6300E-08 | 1.7900E-06 | 1.0000 |
| TG(16:0/16:0/18:2) | TG(20:1/18:2/18:2) | 0.1408 | 4.1200E-10 | 5.6200E-08 | 1.0000 | Icosenoic acid | glutamine | 0.1346 | 2.3900E-09 | 2.9700E-07 | 1.0000 |
| TG(16:0/18:1/18:1) | TG(18:0/18:1/18:1) | 0.1415 | 3.4400E-10 | 4.7600E-08 | 1.0000 | coniferyl alcohol | glutamine | 0.1405 | 4.5300E-10 | 6.1400E-08 | 1.0000 |
| FA(16:0) | FA(18:0) | 0.1420 | 2.9100E-10 | 4.0600E-08 | 1.0000 | Creatine | glutamine | 0.1466 | 7.5800E-11 | 1.1500E-08 | 1.0000 |
| PE(16:0/18:1) | PG(16:0/18:1) | 0.1424 | 2.6400E-10 | 3.7200E-08 | 1.0000 | D-Fructose 2,6-bisphosphate | glutamine | 0.0990 | 1.1800E-05 | 6.7922E-04 | 0.9953 |
| PE(16:0/18:1) | PE(18:1/18:2) | 0.1428 | 2.3100E-10 | 3.2800E-08 | 1.0000 | 3-hydroxybutyric acid | Glutaric Acid | 0.1273 | 1.6400E-08 | 1.8000E-06 | 1.0000 |
| PG(16:0/18:1) | PG(16:0/18:2) | 0.1432 | 2.0900E-10 | 2.9900E-08 | 1.0000 | Citraconic acid | Glutaric Acid | 0.1059 | 2.7600E-06 | 1.9040E-04 | 0.9984 |
| PE(20:0/18:1) | PE(20:0/18:2) | 0.1450 | 1.2300E-10 | 1.8000E-08 | 1.0000 | Anabasine | Glutaric Acid | 0.0964 | 2.0000E-05 | 1.0652E-03 | 0.9933 |
| CL(18:2/18:2/18:2/18:1) | DGDG(16:0/18:2) | 0.1451 | 1.1800E-10 | 1.7400E-08 | 1.0000 | 2,5-Dihydroxybenzaldehyde | glutathione | 0.0995 | 1.0500E-05 | 6.1372E-04 | 0.9953 |
| DG(18:2/18:2) | DG(18:3/18:2) | 0.1453 | 1.1100E-10 | 1.6500E-08 | 1.0000 | 2-Aminophenol | glutathione | 0.1103 | 1.0300E-06 | 7.9800E-05 | 0.9994 |
| FA(18:0) | FA(20:0) | 0.1461 | 8.7300E-11 | 1.3100E-08 | 1.0000 | 2-Hydroxybutanoic acid | glutathione | -0.1004 | 8.8500E-06 | 5.2878E-04 | 0.9961 |
| DG(18:0/18:1) | DG(18:1/18:1) | 0.1466 | 7.6500E-11 | 1.1600E-08 | 1.0000 | 3-Methyl-2-oxobutanoic acid | glutathione | -0.1081 | 1.7000E-06 | 1.2444E-04 | 0.9990 |
| PE(20:0/18:2) | PG(18:0/16:0) | 0.1474 | 6.0300E-11 | 9.3200E-09 | 1.0000 | 3-hydroxybutyric acid | glutathione | 0.1037 | 4.4100E-06 | 2.8637E-04 | 0.9975 |
| DG(18:0/18:1) | DG(20:0/18:2) | 0.1484 | 4.4100E-11 | 6.9300E-09 | 1.0000 | 4-Acetamidobutanoate | glutathione | 0.1315 | 5.4600E-09 | 6.5100E-07 | 1.0000 |
| PE(18:0/18:1) | PG(18:0/18:1) | 0.1494 | 3.3200E-11 | 5.3000E-09 | 1.0000 | 5-Amino-4-imidazolecarboxyamide | glutathione | -0.1163 | 2.5800E-07 | 2.3000E-05 | 0.9998 |
| LPC(16:0) | SQDG(16:0/18:2) | 0.1507 | 2.2300E-11 | 3.6400E-09 | 1.0000 | 9-Fluorenone | glutathione | 0.2168 | 2.2200E-16 | 5.3200E-14 | 1.0000 |
| PG(16:0/18:1) | PG(18:1/18:2) | 0.1510 | 2.0100E-11 | 3.2900E-09 | 1.0000 | Aconitic Acid | glutathione | 0.0958 | 2.2400E-05 | 1.1673E-03 | 0.9933 |
| PE(20:0/18:3) | PG(18:0/16:0) | 0.1515 | 1.7300E-11 | 2.8600E-09 | 1.0000 | Biuret | glutathione | 0.0994 | 1.0900E-05 | 6.3095E-04 | 0.9953 |
| PE(18:0/18:1) | PE(18:1/18:2) | 0.1549 | 5.9200E-12 | 1.0200E-09 | 1.0000 | Octanoic acid | glutathione | 0.1899 | 2.2200E-16 | 5.3200E-14 | 1.0000 |
| LPE(18:2) | SQDG(16:0/18:3) | 0.1551 | 5.5600E-12 | 9.6200E-10 | 1.0000 | fumaric acid | glutathione | 0.0922 | 4.4800E-05 | 2.0709E-03 | 0.9865 |
| TG(18:1/18:1/18:2) | TG(20:1/18:2/18:2) | 0.1585 | 1.8600E-12 | 3.3800E-10 | 1.0000 | Geraniol | glutathione | 0.2878 | 2.2200E-16 | 5.3200E-14 | 1.0000 |
| DG(20:0/18:3) | DG(20:1/18:3) | 0.1587 | 1.7700E-12 | 3.2200E-10 | 1.0000 | D-Glucose | glutathione | 0.1146 | 3.8100E-07 | 3.2800E-05 | 0.9997 |
| DG(16:0/18:1) | DG(18:0/18:1) | 0.1602 | 1.0900E-12 | 2.0300E-10 | 1.0000 | N-Methylhydantoin | glycerol | -0.1064 | 2.4900E-06 | 1.7377E-04 | 0.9984 |
| TG(16:0/16:0/18:3) | TG(20:1/18:2/18:3) | 0.1667 | 1.2200E-13 | 2.4500E-11 | 1.0000 | 2,5-Dihydroxybenzaldehyde | glycerol | 0.1350 | 2.1500E-09 | 2.7000E-07 | 1.0000 |
| PC(16:0/18:3) | PC(18:2/18:2) | 0.1668 | 1.2100E-13 | 2.4200E-11 | 1.0000 | 3-hydroxy-L-proline | glycerol | -0.1107 | 9.4100E-07 | 7.3700E-05 | 0.9994 |
| DG(16:0/18:1) | DG(18:0/18:1) | 0.1684 | 6.9700E-14 | 1.4300E-11 | 1.0000 | Atrazine-2-hydroxy | glycerol | 0.1013 | 7.2500E-06 | 4.4469E-04 | 0.9967 |
| TG(18:0/18:0/18:1) | TG(20:0/18:1/18:1) | 0.1691 | 5.4600E-14 | 1.1300E-11 | 1.0000 | cycloserine | glycerol | 0.0918 | 4.9000E-05 | 2.2319E-03 | 0.9865 |
| TG(16:0/18:1/18:2) | TG(18:1/18:1/18:2) | 0.1739 | 1.0200E-14 | 2.2200E-12 | 1.0000 | glutamine | glycerol | -0.1846 | 2.2200E-16 | 5.3200E-14 | 1.0000 |
| PC(18:1/18:2) | PE(18:1/18:2) | 0.1763 | 4.2200E-15 | 9.5500E-13 | 1.0000 | glutathione | glycerol | -0.1646 | 2.4900E-13 | 4.8800E-11 | 1.0000 |
| DG(18:0/18:1) | DG(18:1/18:1) | 0.1766 | 4.0000E-15 | 9.0700E-13 | 1.0000 | 2-Oxobutyric acid | glycolic acid | -0.1191 | 1.3100E-07 | 1.2300E-05 | 0.9999 |
| PE(18:1/18:1) | PE(18:1/18:2) | 0.1772 | 3.1100E-15 | 7.1200E-13 | 1.0000 | 3-hydroxybutyric acid | glycolic acid | 0.0931 | 3.8300E-05 | 1.8176E-03 | 0.9895 |
| PE(18:0/18:1) | PE(20:0/18:1) | 0.1781 | 2.2200E-15 | 5.1400E-13 | 1.0000 | 6-Phospho-D-gluconate | glycolic acid | -0.1259 | 2.4000E-08 | 2.5600E-06 | 1.0000 |
| TG(18:0/16:0/18:1) | TG(18:0/18:1/18:1) | 0.1804 | 8.8800E-16 | 2.0900E-13 | 1.0000 | Capric Acid | glycolic acid | 0.1234 | 4.5500E-08 | 4.6600E-06 | 0.9999 |
| PC(16:0/18:1) | PE(16:0/18:1) | 0.1848 | 2.2200E-16 | 5.3200E-14 | 1.0000 | Farnesal | glycolic acid | 0.1102 | 1.0500E-06 | 8.1000E-05 | 0.9994 |
| TG(18:1/18:1/18:3) | TG(20:1/18:3/18:3) | 0.1854 | 2.2200E-16 | 5.3200E-14 | 1.0000 | gentisic acid | glycolic acid | -0.0918 | 4.8900E-05 | 2.2288E-03 | 0.9865 |
| DG(20:0/18:2) | DG(20:0/18:3) | 0.1856 | 2.2200E-16 | 5.3200E-14 | 1.0000 | 19-Hydroxyandrost-4-ene-3,17-dione | guanine | 0.1040 | 4.1500E-06 | 2.7144E-04 | 0.9983 |
| TG(16:0/18:1/18:2) | TG(16:0/18:1/18:3) | 0.1865 | 2.2200E-16 | 5.3200E-14 | 1.0000 | Atropine | guanine | 0.2325 | 2.2200E-16 | 5.3200E-14 | 1.0000 |
| PC(18:1/18:1) | PE(18:1/18:1) | 0.1866 | 2.2200E-16 | 5.3200E-14 | 1.0000 | Geraniol | guanine | -0.1184 | 1.5700E-07 | 1.4500E-05 | 0.9999 |
| CL(18:2/18:1/18:1/18:2) | CL(18:2/18:2/18:2/18:1) | 0.1885 | 2.2200E-16 | 5.3200E-14 | 1.0000 | Glucose-6-phosphate | guanine | 0.1144 | 4.0700E-07 | 3.4800E-05 | 0.9997 |
| TG(18:1/18:1/18:3) | TG(20:2/18:2/18:2) | 0.1896 | 2.2200E-16 | 5.3200E-14 | 1.0000 | Glutaric Acid | guanine | 0.1118 | 7.2900E-07 | 5.8700E-05 | 0.9994 |
| TG(16:0/16:0/18:3) | TG(16:0/18:3/18:3) | 0.1924 | 2.2200E-16 | 5.3200E-14 | 1.0000 | 2-Oxobutyric acid | Hesperetin | 0.0905 | 6.2600E-05 | 2.7331E-03 | 0.9833 |
| CL(18:2/18:1/18:1/18:2) | DGDG(16:0/18:2) | 0.1930 | 2.2200E-16 | 5.3200E-14 | 1.0000 | 4-Acetamidobutanoate | Hesperetin | 0.1191 | 1.3100E-07 | 1.2400E-05 | 0.9999 |
| PG(16:0/18:1) | PG(18:0/18:2) | 0.1945 | 2.2200E-16 | 5.3200E-14 | 1.0000 | 6-Phospho-D-gluconate | Hesperetin | 0.0987 | 1.2400E-05 | 7.0893E-04 | 0.9953 |
| PI(18:1/18:1) | PI(18:1/18:2) | 0.1966 | 2.2200E-16 | 5.3200E-14 | 1.0000 | cholic acid | Hesperetin | 0.0974 | 1.6200E-05 | 8.9021E-04 | 0.9940 |
| TG(16:0/18:1/18:1) | TG(20:1/18:1/18:2) | 0.1975 | 2.2200E-16 | 5.3200E-14 | 1.0000 | 3-hydroxy-L-proline | hydroxylamine | -0.0991 | 1.1600E-05 | 6.6984E-04 | 0.9953 |
| TG(16:0/16:0/18:1) | TG(16:0/16:0/18:3) | 0.2002 | 2.2200E-16 | 5.3200E-14 | 1.0000 | 3-Methylcatechol | hydroxylamine | 0.1225 | 5.6300E-08 | 5.6600E-06 | 0.9999 |
| PG(16:0/16:0) | PG(16:0/18:2) | 0.2006 | 2.2200E-16 | 5.3200E-14 | 1.0000 | Acetol | hydroxylamine | 0.1039 | 4.2000E-06 | 2.7433E-04 | 0.9983 |
| PC(16:0/18:3) | PE(18:3/18:3) | 0.2033 | 2.2200E-16 | 5.3200E-14 | 1.0000 | 19-Hydroxyandrost-4-ene-3,17-dione | indole-3-acetamide | 0.0905 | 6.2100E-05 | 2.7120E-03 | 0.9833 |
| PE(16:0/18:3) | PE(20:0/18:3) | 0.2038 | 2.2200E-16 | 5.3200E-14 | 1.0000 | Arachidic acid | indole-3-acetamide | 0.0921 | 4.5900E-05 | 2.1108E-03 | 0.9865 |
| TG(20:1/18:2/18:2) | TG(20:1/18:2/18:3) | 0.2095 | 2.2200E-16 | 5.3200E-14 | 1.0000 | 3-Methyl-2-oxobutanoic acid | isocitric acid | -0.1081 | 1.7100E-06 | 1.2508E-04 | 0.9990 |
| PC(16:0/18:1) | PC(18:1/18:2) | 0.2115 | 2.2200E-16 | 5.3200E-14 | 1.0000 | Creatine | isocitric acid | 0.1086 | 1.5100E-06 | 1.1179E-04 | 0.9991 |
| PE(18:1/18:2) | PG(18:1/18:2) | 0.2122 | 2.2200E-16 | 5.3200E-14 | 1.0000 | gentisic acid | isocitric acid | 0.1945 | 2.2200E-16 | 5.3200E-14 | 1.0000 |
| TG(16:0/16:0/18:1) | TG(16:0/18:1/18:1) | 0.2159 | 2.2200E-16 | 5.3200E-14 | 1.0000 | glycerol | isocitric acid | 0.1247 | 3.2600E-08 | 3.4100E-06 | 0.9999 |
| PE(18:3/18:3) | PE(20:0/18:3) | 0.2163 | 2.2200E-16 | 5.3200E-14 | 1.0000 | alpha-D-glucosamine 1-phosphate | Isoleucine | -0.0945 | 2.8800E-05 | 1.4309E-03 | 0.9933 |
| TG(16:0/16:0/18:2) | TG(16:0/18:1/18:2) | 0.2210 | 2.2200E-16 | 5.3200E-14 | 1.0000 | dibenzofuran | Isoleucine | 0.0905 | 6.1900E-05 | 2.7066E-03 | 0.9833 |
| DG(20:1/18:2) | DG(20:1/18:3) | 0.2227 | 2.2200E-16 | 5.3200E-14 | 1.0000 | fucose | Isoleucine | 0.0948 | 2.7300E-05 | 1.3710E-03 | 0.9933 |
| TG(20:1/18:3/18:3) | TG(20:2/18:2/18:2) | 0.2294 | 2.2200E-16 | 5.3200E-14 | 1.0000 | isocitric acid | Isoleucine | 0.4602 | 2.2200E-16 | 5.3200E-14 | 1.0000 |
| TG(16:0/18:1/18:3) | TG(18:3/18:2/18:3) | 0.2298 | 2.2200E-16 | 5.3200E-14 | 1.0000 | β-Sitosterol | Itaconic acid | -0.1308 | 6.6900E-09 | 7.8700E-07 | 1.0000 |
| CL(18:3/18:2/18:2/18:2) | DGDG(18:3/18:3) | 0.2303 | 2.2200E-16 | 5.3200E-14 | 1.0000 | 3-hydroxybutyric acid | Itaconic acid | -0.1767 | 3.7700E-15 | 8.5900E-13 | 1.0000 |
| PC(16:0/18:3) | PE(16:0/18:3) | 0.2309 | 2.2200E-16 | 5.3200E-14 | 1.0000 | 6-Hydroxynicotinic acid | Itaconic acid | 0.1094 | 1.2600E-06 | 9.5200E-05 | 0.9991 |
| PG(18:0/18:2) | PG(18:1/18:2) | 0.2316 | 2.2200E-16 | 5.3200E-14 | 1.0000 | alpha-ketoisocaproic acid | Itaconic acid | -0.1218 | 6.7400E-08 | 6.6700E-06 | 0.9999 |
| PC(18:2/18:2) | PE(18:2/18:2) | 0.2327 | 2.2200E-16 | 5.3200E-14 | 1.0000 | fumaric acid | Itaconic acid | 0.1089 | 1.4200E-06 | 1.0639E-04 | 0.9991 |
| FA(18:2) | FA(18:3) | 0.2339 | 2.2200E-16 | 5.3200E-14 | 1.0000 | glycolic acid | Itaconic acid | 0.1162 | 2.6600E-07 | 2.3600E-05 | 0.9998 |
| LPC(16:0) | LPE(16:0) | 0.2364 | 2.2200E-16 | 5.3200E-14 | 1.0000 | hydroxylamine | Itaconic acid | -0.0944 | 2.9400E-05 | 1.4560E-03 | 0.9933 |
| LPC(18:2) | LPE(18:2) | 0.2397 | 2.2200E-16 | 5.3200E-14 | 1.0000 | indole-3-acetamide | Itaconic acid | 0.0910 | 5.6400E-05 | 2.5054E-03 | 0.9865 |
| TG(16:0/18:3/18:3) | TG(18:3/18:3/18:3) | 0.2422 | 2.2200E-16 | 5.3200E-14 | 1.0000 | β-Sitosterol | lauric acid | -0.1636 | 3.5400E-13 | 6.8700E-11 | 1.0000 |
| PE(16:0/18:2) | PE(18:2/18:2) | 0.2450 | 2.2200E-16 | 5.3200E-14 | 1.0000 | 2-Oxobutyric acid | lauric acid | 0.1450 | 1.2100E-10 | 1.7800E-08 | 1.0000 |
| LPC(18:1) | LPE(18:1) | 0.2467 | 2.2200E-16 | 5.3200E-14 | 1.0000 | 3-Hydroxyphenylacetic acid | lauric acid | 0.1043 | 3.8700E-06 | 2.5582E-04 | 0.9983 |
| TG(18:3/18:2/18:3) | TG(18:3/18:3/18:3) | 0.2518 | 2.2200E-16 | 5.3200E-14 | 1.0000 | 3-Methylcatechol | lauric acid | 0.1202 | 1.0000E-07 | 9.6800E-06 | 0.9999 |
| TG(18:2/18:2/18:2) | TG(18:3/18:2/18:2) | 0.2532 | 2.2200E-16 | 5.3200E-14 | 1.0000 | 4-Aminobutyric acid(GABA) | lauric acid | 0.0948 | 2.7100E-05 | 1.3634E-03 | 0.9933 |
| PG(16:0/18:2) | PG(18:0/18:2) | 0.2570 | 2.2200E-16 | 5.3200E-14 | 1.0000 | 4-Hydroxybenzoic acid | lauric acid | 0.2008 | 2.2200E-16 | 5.3200E-14 | 1.0000 |
| FA(18:1) | FA(20:1) | 0.2623 | 2.2200E-16 | 5.3200E-14 | 1.0000 | Acetol | lauric acid | 0.1068 | 2.2800E-06 | 1.6065E-04 | 0.9984 |
| TG(16:0/18:1/18:2) | TG(18:1/18:1/18:2) | 0.2678 | 2.2200E-16 | 5.3200E-14 | 1.0000 | adenine | lauric acid | 0.1740 | 9.7700E-15 | 2.1300E-12 | 1.0000 |
| PC(18:0/18:1) | PE(18:0/18:1) | 0.2760 | 2.2200E-16 | 5.3200E-14 | 1.0000 | alpha-ketoisocaproic acid | lauric acid | 0.1503 | 2.4900E-11 | 4.0400E-09 | 1.0000 |
| PC(16:0/18:1) | PC(18:0/18:1) | 0.2781 | 2.2200E-16 | 5.3200E-14 | 1.0000 | Atropine | lauric acid | 0.1458 | 9.5900E-11 | 1.4300E-08 | 1.0000 |
| TG(18:0/18:0/18:1) | TG(18:0/18:1/18:1) | 0.2795 | 2.2200E-16 | 5.3200E-14 | 1.0000 | benzoic acid | lauric acid | -0.0957 | 2.2900E-05 | 1.1884E-03 | 0.9933 |
| PE(18:0/18:1) | PE(18:1/18:1) | 0.2870 | 2.2200E-16 | 5.3200E-14 | 1.0000 | Octanoic acid | lauric acid | 0.1006 | 8.3800E-06 | 5.0444E-04 | 0.9961 |
| PE(20:0/18:2) | PE(20:0/18:3) | 0.2883 | 2.2200E-16 | 5.3200E-14 | 1.0000 | Glyceryl 1-phosphate | lauric acid | 0.0923 | 4.4100E-05 | 2.0441E-03 | 0.9865 |
| TG(18:0/18:1/18:1) | TG(20:0/18:1/18:2) | 0.2928 | 2.2200E-16 | 5.3200E-14 | 1.0000 | hydroxylamine | lauric acid | 0.2923 | 2.2200E-16 | 5.3200E-14 | 1.0000 |
| PG(16:0/18:2) | PG(18:1/18:2) | 0.2930 | 2.2200E-16 | 5.3200E-14 | 1.0000 | 3-Hydroxyphenylacetic acid | leucine | 0.0998 | 1.0000E-05 | 5.8718E-04 | 0.9953 |
| PE(16:0/18:3) | PE(18:3/18:3) | 0.2984 | 2.2200E-16 | 5.3200E-14 | 1.0000 | 3-Methylbenzyl Alcohol | leucine | 0.1491 | 3.6100E-11 | 5.7200E-09 | 1.0000 |
| TG(16:0/16:0/18:3) | TG(16:0/18:2/18:3) | 0.3036 | 2.2200E-16 | 5.3200E-14 | 1.0000 | 5-aminovaleric acid lactam | leucine | -0.0945 | 2.8700E-05 | 1.4298E-03 | 0.9933 |
| PE(18:2/18:2) | PE(18:3/18:2) | 0.3051 | 2.2200E-16 | 5.3200E-14 | 1.0000 | alpha-D-glucosamine 1-phosphate | leucine | -0.1021 | 6.1300E-06 | 3.8380E-04 | 0.9967 |
| PI(18:1/18:2) | PI(18:2/18:2) | 0.3101 | 2.2200E-16 | 5.3200E-14 | 1.0000 | fumaric acid | leucine | 0.1080 | 1.7300E-06 | 1.2640E-04 | 0.9990 |
| PE(18:1/18:2) | PE(18:2/18:2) | 0.3107 | 2.2200E-16 | 5.3200E-14 | 1.0000 | Geraniol | leucine | 0.1101 | 1.0800E-06 | 8.3100E-05 | 0.9992 |
| PE(16:0/18:1) | PE(18:0/18:1) | 0.3167 | 2.2200E-16 | 5.3200E-14 | 1.0000 | D-Glucose | leucine | 0.2034 | 2.2200E-16 | 5.3200E-14 | 1.0000 |
| PI(18:0/18:1) | PI(18:1/18:1) | 0.3212 | 2.2200E-16 | 5.3200E-14 | 1.0000 | glutamine | leucine | -0.1408 | 4.2000E-10 | 5.7200E-08 | 1.0000 |
| PG(16:0/18:1) | PG(18:0/18:1) | 0.3254 | 2.2200E-16 | 5.3200E-14 | 1.0000 | glycerol | leucine | 0.1280 | 1.3900E-08 | 1.5500E-06 | 1.0000 |
| TG(16:0/18:2/18:3) | TG(16:0/18:3/18:3) | 0.3323 | 2.2200E-16 | 5.3200E-14 | 1.0000 | guanine | leucine | 0.0979 | 1.4800E-05 | 8.2034E-04 | 0.9940 |
| DG(16:0/16:0) | DG(18:0/18:0) | 0.3456 | 2.2200E-16 | 5.3200E-14 | 1.0000 | L-2,4-Diaminobutanoate | L-homoserine | 0.1003 | 9.0400E-06 | 5.3827E-04 | 0.9961 |
| PC(18:0/18:1) | PC(18:1/18:1) | 0.3544 | 2.2200E-16 | 5.3200E-14 | 1.0000 | Aconitic Acid | L-homoserine | -0.1538 | 8.4600E-12 | 1.4400E-09 | 1.0000 |
| PI(16:0/18:1) | PI(18:0/18:1) | 0.3673 | 2.2200E-16 | 5.3200E-14 | 1.0000 | dibenzofuran | L-homoserine | -0.1201 | 1.0200E-07 | 9.8600E-06 | 0.9999 |
| PE(18:3/18:2) | PE(18:3/18:3) | 0.3673 | 2.2200E-16 | 5.3200E-14 | 1.0000 | hydroxylamine | L-homoserine | 0.0951 | 2.5600E-05 | 1.3047E-03 | 0.9933 |
| TG(16:0/18:1/18:3) | TG(16:0/18:2/18:2) | 0.3798 | 2.2200E-16 | 5.3200E-14 | 1.0000 | 3-Hydroxypropionic acid | Lignoceric acid | 0.0931 | 3.8000E-05 | 1.8049E-03 | 0.9895 |
| DG(16:0/16:0) | DG(18:0/16:0) | 0.3877 | 2.2200E-16 | 5.3200E-14 | 1.0000 | benzoic acid | Lignoceric acid | -0.0960 | 2.1400E-05 | 1.1251E-03 | 0.9933 |
| PG(18:0/18:1) | PG(18:0/18:2) | 0.3939 | 2.2200E-16 | 5.3200E-14 | 1.0000 | glycolic acid | Lignoceric acid | 0.1275 | 1.5800E-08 | 1.7400E-06 | 1.0000 |
| PC(16:0/18:2) | PC(16:0/18:3) | 0.3968 | 2.2200E-16 | 5.3200E-14 | 1.0000 | indole-3-acetamide | Lignoceric acid | -0.1041 | 4.0500E-06 | 2.6600E-04 | 0.9983 |
| TG(16:0/18:1/18:2) | TG(16:0/18:1/18:3) | 0.4302 | 2.2200E-16 | 5.3200E-14 | 1.0000 | alanine | linoleic acid | 0.1042 | 3.9800E-06 | 2.6230E-04 | 0.9983 |
| LPC(16:0) | LPC(18:0) | 0.4411 | 2.2200E-16 | 5.3200E-14 | 1.0000 | Behenic acid | linoleic acid | 0.1016 | 6.8700E-06 | 4.2418E-04 | 0.9967 |
| DG(18:0/16:0) | DG(18:0/18:0) | 0.4440 | 2.2200E-16 | 5.3200E-14 | 1.0000 | L-2,4-Diaminobutanoate | linolenic acid | 0.1113 | 8.3100E-07 | 6.6100E-05 | 0.9994 |
| PI(16:0/18:3) | PI(18:2/18:2) | 0.4490 | 2.2200E-16 | 5.3200E-14 | 1.0000 | 2-Aminophenol | linolenic acid | -0.0949 | 2.6600E-05 | 1.3460E-03 | 0.9933 |
| PG(16:0/16:0) | PG(18:0/16:0) | 0.4800 | 2.2200E-16 | 5.3200E-14 | 1.0000 | 3-Methylbenzyl Alcohol | linolenic acid | 0.1033 | 4.8100E-06 | 3.0913E-04 | 0.9975 |
| PC(16:0/18:2) | PC(18:2/18:2) | 0.4924 | 2.2200E-16 | 5.3200E-14 | 1.0000 | Icosenoic acid | linolenic acid | -0.1457 | 1.0100E-10 | 1.5000E-08 | 1.0000 |
| PE(18:1/18:1) | PE(18:1/18:2) | 0.5015 | 2.2200E-16 | 5.3200E-14 | 1.0000 | Sphingosine | linolenic acid | 0.1387 | 7.5400E-10 | 9.9900E-08 | 1.0000 |
| PE(18:1/18:2) | PE(18:2/18:2) | 0.5416 | 2.2200E-16 | 5.3200E-14 | 1.0000 | glycolic acid | linolenic acid | 0.0929 | 3.9800E-05 | 1.8757E-03 | 0.9895 |
| TG(18:1/18:1/18:3) | TG(18:1/18:2/18:2) | 0.7016 | 2.2200E-16 | 5.3200E-14 | 1.0000 | D-Glyceric acid | L-Malic acid | 0.1040 | 4.1500E-06 | 2.7148E-04 | 0.9983 |
| DG(16:1/18:2) | DG(20:1/18:2) | -0.0999 | 9.7200E-06 | 5.7308E-04 | 0.9961 | dibenzofuran | L-Malic acid | 0.1164 | 2.5000E-07 | 2.2400E-05 | 0.9998 |
| DG(16:1/18:2) | PE(16:0/18:3) | 0.0917 | 4.9500E-05 | 2.2525E-03 | 0.9865 | cholic acid | Maleamate | 0.1260 | 2.3500E-08 | 2.5000E-06 | 1.0000 |
| DG(16:1/18:2) | PI(16:0/18:3) | 0.0918 | 4.8700E-05 | 2.2198E-03 | 0.9865 | L-kynurenine | Maleamate | 0.5922 | 2.2200E-16 | 5.3200E-14 | 1.0000 |
| DG(16:1/18:3) | DG(17:0/18:2) | -0.1343 | 2.5800E-09 | 3.2000E-07 | 1.0000 | 19-Hydroxyandrost-4-ene-3,17-dione | maleic acid | 0.1134 | 5.1400E-07 | 4.2800E-05 | 0.9996 |
| DG(16:1/18:3) | DG(18:3/18:2) | 0.1636 | 3.5600E-13 | 6.9000E-11 | 1.0000 | D-Glyceric acid | maleic acid | 0.0937 | 3.3500E-05 | 1.6267E-03 | 0.9895 |
| DG(16:1/18:3) | DG(18:3/18:2) | 0.1636 | 3.5500E-13 | 6.8900E-11 | 1.0000 | guanine | maleic acid | 0.0951 | 2.5800E-05 | 1.3129E-03 | 0.9933 |
| DG(16:1/18:3) | DG(18:3/18:3) | 0.1603 | 1.0600E-12 | 1.9900E-10 | 1.0000 | 5-Aminovaleric acid | malonic acid | 0.1253 | 2.8200E-08 | 2.9700E-06 | 0.9999 |
| DG(16:1/18:3) | DG(20:0/18:3) | 0.1061 | 2.6300E-06 | 1.8287E-04 | 0.9984 | Creatine | malonic acid | 0.0962 | 2.0600E-05 | 1.0902E-03 | 0.9933 |
| DG(16:1/18:3) | DG(20:1/18:2) | -0.0969 | 1.7900E-05 | 9.6707E-04 | 0.9933 | Glucose-6-phosphate | malonic acid | 0.1351 | 2.0800E-09 | 2.6100E-07 | 1.0000 |
| DG(17:0/18:1) | DG(17:0/18:2) | 0.2469 | 2.2200E-16 | 5.3200E-14 | 1.0000 | L-kynurenine | malonic acid | 0.1783 | 2.2200E-15 | 5.1400E-13 | 1.0000 |
| DG(17:0/18:1) | DG(17:1/18:2) | 0.1665 | 1.3100E-13 | 2.6200E-11 | 1.0000 | 2-Aminophenol | mannose | 0.1288 | 1.1200E-08 | 1.2700E-06 | 1.0000 |
| DG(17:0/18:1) | DG(18:0/18:1) | 0.2181 | 2.2200E-16 | 5.3200E-14 | 1.0000 | 3-Methyl-2-oxobutanoic acid | mannose | -0.0925 | 4.2800E-05 | 1.9895E-03 | 0.9881 |
| DG(17:0/18:2) | DG(17:1/18:2) | 0.1024 | 5.8000E-06 | 3.6517E-04 | 0.9967 | Biphenyl | mannose | 0.1282 | 1.3200E-08 | 1.4800E-06 | 1.0000 |
| DG(17:0/18:2) | DG(20:0/18:2) | 0.0932 | 3.7300E-05 | 1.7786E-03 | 0.9895 | Glyceryl 1-phosphate | mannose | 0.1131 | 5.4500E-07 | 4.5100E-05 | 0.9996 |
| DG(17:1/18:2) | DG(18:1/18:2) | 0.1318 | 5.0500E-09 | 6.0500E-07 | 1.0000 | gentisic acid | mannose | 0.0976 | 1.5500E-05 | 8.5795E-04 | 0.9940 |
| DG(17:1/18:2) | DG(18:1/18:2) | 0.1490 | 3.6700E-11 | 5.8100E-09 | 1.0000 | Geraniol | mannose | 0.1034 | 4.7400E-06 | 3.0544E-04 | 0.9975 |
| DG(17:1/18:2) | DG(18:2/18:2) | 0.1112 | 8.4400E-07 | 6.7000E-05 | 0.9994 | linolenic acid | mannose | 0.0947 | 2.8000E-05 | 1.4018E-03 | 0.9933 |
| DG(18:0/16:0) | DG(18:0/18:0) | 0.4440 | 2.2200E-16 | 5.3200E-14 | 1.0000 | glutamine | Menthone | 0.0987 | 1.2600E-05 | 7.1744E-04 | 0.9953 |
| DG(18:0/16:0) | FA(14:0) | 0.1938 | 2.2200E-16 | 5.3200E-14 | 1.0000 | maleic acid | Menthone | 0.0954 | 2.4100E-05 | 1.2431E-03 | 0.9933 |
| DG(18:0/18:0) | FA(14:0) | 0.1044 | 3.7800E-06 | 2.5112E-04 | 0.9983 | Octanoic acid | Methyl jasmonate | 0.1048 | 3.5100E-06 | 2.3537E-04 | 0.9983 |
| DG(18:0/18:1) | DG(18:1/18:1) | 0.1766 | 4.0000E-15 | 9.0700E-13 | 1.0000 | Ethanolamine | Methyl jasmonate | 0.1141 | 4.3700E-07 | 3.7000E-05 | 0.9997 |
| DG(18:0/18:1) | DG(18:1/18:1) | 0.1466 | 7.6500E-11 | 1.1600E-08 | 1.0000 | Farnesal | Methyl jasmonate | -0.1104 | 1.0200E-06 | 7.9000E-05 | 0.9994 |
| DG(18:0/18:1) | DG(20:0/18:2) | 0.1484 | 4.4100E-11 | 6.9300E-09 | 1.0000 | Glutaric Acid | Methyl jasmonate | -0.1093 | 1.2900E-06 | 9.7700E-05 | 0.9991 |
| DG(18:0/18:1) | PC(18:0/18:1) | 0.0944 | 2.9300E-05 | 1.4497E-03 | 0.9933 | L-homoserine | Methyl jasmonate | 0.1799 | 1.3300E-15 | 3.1200E-13 | 1.0000 |
| DG(18:0/18:1) | PE(16:0/18:1) | -0.0999 | 9.7000E-06 | 5.7214E-04 | 0.9961 | malonic acid | Methyl jasmonate | 0.0954 | 2.4100E-05 | 1.2406E-03 | 0.9933 |
| DG(18:0/18:1) | PG(18:0/18:1) | 0.1132 | 5.2900E-07 | 4.3900E-05 | 0.9996 | 3-Methylcatechol | Inositol | 0.1373 | 1.1200E-09 | 1.4600E-07 | 1.0000 |
| DG(18:0/18:1) | PI(18:0/18:1) | 0.1047 | 3.5500E-06 | 2.3787E-04 | 0.9983 | citric acid | Inositol | 0.1273 | 1.6700E-08 | 1.8300E-06 | 1.0000 |
| DG(18:1/18:1) | DG(18:1/18:1) | 0.3773 | 2.2200E-16 | 5.3200E-14 | 1.0000 | L-homoserine | Inositol | -0.1023 | 5.8900E-06 | 3.7021E-04 | 0.9967 |
| DG(18:1/18:1) | DG(20:0/18:2) | 0.1019 | 6.4400E-06 | 4.0104E-04 | 0.9967 | linolenic acid | Inositol | 0.1216 | 7.0100E-08 | 6.9100E-06 | 0.9999 |
| DG(18:1/18:1) | CerG1(d24:0/18:1) | 0.1105 | 9.8300E-07 | 7.6500E-05 | 0.9994 | maleic acid | Myristic Acid | 0.1292 | 1.0100E-08 | 1.1600E-06 | 1.0000 |
| DG(18:1/18:1) | LPC(18:2) | -0.0981 | 1.4100E-05 | 7.8788E-04 | 0.9947 | 2-Aminophenol | N-Acetyl-beta-alanine | 0.1029 | 5.1800E-06 | 3.3019E-04 | 0.9967 |
| DG(18:1/18:1) | FA(20:4) | 0.1479 | 5.2100E-11 | 8.1200E-09 | 1.0000 | D-Glyceric acid | N-Acetyl-beta-alanine | 0.1278 | 1.4800E-08 | 1.6400E-06 | 1.0000 |
| DG(18:1/18:1) | DG(18:3/18:3) | 0.1362 | 1.5100E-09 | 1.9300E-07 | 1.0000 | Glucose-1-phosphate | N-Acetyl-beta-alanine | -0.1035 | 4.6000E-06 | 2.9739E-04 | 0.9975 |
| DG(18:1/18:1) | DG(20:0/18:2) | -0.1684 | 6.9700E-14 | 1.4300E-11 | 1.0000 | L-homoserine | N-Acetyl-beta-alanine | -0.0984 | 1.3300E-05 | 7.4841E-04 | 0.9949 |
| DG(18:1/18:1) | DG(24:0/18:2) | 0.1104 | 1.0100E-06 | 7.8700E-05 | 0.9994 | maleic acid | N-Acetyl-beta-alanine | 0.1008 | 8.1700E-06 | 4.9344E-04 | 0.9961 |
| DG(18:1/18:1) | Cer(d18:2/16:0 | -0.1020 | 6.2900E-06 | 3.9247E-04 | 0.9967 | Myristic Acid | N-Acetyl-beta-alanine | 0.1471 | 6.5100E-11 | 1.0000E-08 | 1.0000 |
| DG(18:1/18:1) | LPC(18:1) | 0.1395 | 6.0700E-10 | 8.1100E-08 | 1.0000 | 3-Methyl-2-oxobutanoic acid | N-Carbamyl-L-glutamate | 0.0985 | 1.3100E-05 | 7.4289E-04 | 0.9953 |
| DG(18:1/18:1) | FA(20:4) | -0.1129 | 5.7600E-07 | 4.7400E-05 | 0.9994 | Glucose-6-phosphate | N-Carbamyl-L-glutamate | 0.1032 | 4.9000E-06 | 3.1405E-04 | 0.9975 |
| DG(18:1/18:2) | DG(18:1/18:2) | 0.4319 | 2.2200E-16 | 5.3200E-14 | 1.0000 | glycerol | N-Carbamyl-L-glutamate | 0.1273 | 1.6600E-08 | 1.8200E-06 | 1.0000 |
| DG(18:1/18:2) | DG(20:1/18:2) | 0.1402 | 4.9700E-10 | 6.7000E-08 | 1.0000 | linolenic acid | N-Carbamyl-L-glutamate | 0.1650 | 2.2300E-13 | 4.3800E-11 | 1.0000 |
| DG(18:1/18:2) | DG(20:1/18:2) | 0.1392 | 6.5700E-10 | 8.7400E-08 | 1.0000 | 3-Methylbenzyl Alcohol | N-epsilon-Acetyl-L-lysine | -0.1236 | 4.2900E-08 | 4.4000E-06 | 0.9999 |
| DG(18:2/18:2) | DG(18:2/18:2) | 0.2902 | 2.2200E-16 | 5.3200E-14 | 1.0000 | 5-Amino-4-imidazolecarboxyamide | N-epsilon-Acetyl-L-lysine | 0.1007 | 8.2900E-06 | 4.9978E-04 | 0.9961 |
| DG(18:2/18:2) | DG(18:3/18:2) | 0.1052 | 3.1900E-06 | 2.1646E-04 | 0.9983 | Capric Acid | N-epsilon-Acetyl-L-lysine | 0.1896 | 2.2200E-16 | 5.3200E-14 | 1.0000 |
| DG(18:2/18:2) | DG(18:3/18:2) | 0.1229 | 5.1200E-08 | 5.1900E-06 | 0.9999 | Farnesal | N-epsilon-Acetyl-L-lysine | 0.6399 | 2.2200E-16 | 5.3200E-14 | 1.0000 |
| DG(18:2/18:2) | DG(20:1/18:2) | 0.0965 | 1.9300E-05 | 1.0330E-03 | 0.9933 | Lignoceric acid | N-epsilon-Acetyl-L-lysine | 0.1507 | 2.1900E-11 | 3.5800E-09 | 1.0000 |
| DG(18:2/18:2) | DG(20:1/18:3) | 0.1273 | 1.6500E-08 | 1.8100E-06 | 1.0000 | Menthone | N-epsilon-Acetyl-L-lysine | 0.0957 | 2.2700E-05 | 1.1807E-03 | 0.9933 |
| DG(18:2/18:2) | DG(18:3/18:2) | 0.1453 | 1.1100E-10 | 1.6500E-08 | 1.0000 | 2-Aminooctadecane-1,3-diol | N-formyl-L-methionine | 0.0968 | 1.8200E-05 | 9.8221E-04 | 0.9933 |
| DG(18:2/18:2) | DG(20:1/18:2) | 0.1176 | 1.9000E-07 | 1.7400E-05 | 0.9998 | linoleic acid | N-formyl-L-methionine | 0.0958 | 2.2500E-05 | 1.1753E-03 | 0.9933 |
| DG(18:2/18:2) | DG(20:1/18:3) | 0.1158 | 2.8800E-07 | 2.5400E-05 | 0.9998 | β-Sitosterol | nicotinamide | -0.1158 | 2.9300E-07 | 2.5800E-05 | 0.9997 |
| DG(18:2/23:0) | DG(20:0/18:2) | 0.0953 | 2.4700E-05 | 1.2662E-03 | 0.9933 | dibenzofuran | nicotinamide | 0.1040 | 4.1000E-06 | 2.6889E-04 | 0.9983 |
| DG(18:2/23:0) | DG(22:0/18:2) | 0.2320 | 2.2200E-16 | 5.3200E-14 | 1.0000 | 2-Aminooctadecane-1,3-diol | nicotinamide | 0.1040 | 4.1000E-06 | 2.6871E-04 | 0.9983 |
| DG(18:2/23:0) | DG(24:0/18:2) | 0.3176 | 2.2200E-16 | 5.3200E-14 | 1.0000 | linoleic acid | nicotinamide | 0.1231 | 4.8600E-08 | 4.9400E-06 | 0.9999 |
| DG(18:2/23:0) | PE(18:2/23:0) | 0.1019 | 6.3900E-06 | 3.9808E-04 | 0.9967 | L-kynurenine | nicotinamide | 0.1961 | 2.2200E-16 | 5.3200E-14 | 1.0000 |
| DG(18:3/18:2) | DG(18:3/18:2) | 0.2732 | 2.2200E-16 | 5.3200E-14 | 1.0000 | Methyl jasmonate | nicotinamide | 0.2048 | 2.2200E-16 | 5.3200E-14 | 1.0000 |
| DG(18:3/18:2) | D-Glyceric acid | -0.0935 | 3.4800E-05 | 1.6782E-03 | 0.9895 | 1-Hexadecanol | Nicotinoylglycine | -0.0975 | 1.6100E-05 | 8.8269E-04 | 0.9940 |
| DG(18:3/18:2) | DG(18:3/18:3) | 0.0960 | 2.1400E-05 | 1.1251E-03 | 0.9933 | 2-Aminophenol | Nicotinoylglycine | 0.0906 | 6.1500E-05 | 2.6932E-03 | 0.9833 |
| DG(18:3/18:2) | DG(20:1/18:3) | 0.0950 | 2.6300E-05 | 1.3316E-03 | 0.9933 | 3-Phenylcatechol | Nicotinoylglycine | -0.1361 | 1.5700E-09 | 2.0100E-07 | 1.0000 |
| DG(18:3/18:3) | DG(18:3/18:3) | 0.4967 | 2.2200E-16 | 5.3200E-14 | 1.0000 | 4-Hydroxybenzoic acid | Nicotinoylglycine | 0.1045 | 3.7200E-06 | 2.4746E-04 | 0.9983 |
| DG(20:0/18:2) | DG(20:0/18:3) | 0.1856 | 2.2200E-16 | 5.3200E-14 | 1.0000 | alpha-ketoisocaproic acid | Nicotinoylglycine | 0.0945 | 2.9200E-05 | 1.4464E-03 | 0.9933 |
| DG(20:0/18:2) | DG(20:1/18:2) | 0.1115 | 7.7900E-07 | 6.2400E-05 | 0.9994 | Atropine | Nicotinoylglycine | 0.1036 | 4.5500E-06 | 2.9441E-04 | 0.9975 |
| DG(20:0/18:2) | DG(22:0/18:2) | 0.2014 | 2.2200E-16 | 5.3200E-14 | 1.0000 | Behenic acid | Nicotinoylglycine | 0.0999 | 9.8400E-06 | 5.7912E-04 | 0.9961 |
| DG(20:0/18:2) | PE(18:2/18:2) | 0.0933 | 3.6500E-05 | 1.7479E-03 | 0.9895 | Biphenyl | Nicotinoylglycine | 0.1610 | 8.4300E-13 | 1.5900E-10 | 1.0000 |
| DG(20:0/18:2) | PG(18:0/18:1) | -0.0929 | 3.9600E-05 | 1.8672E-03 | 0.9895 | lauric acid | Nicotinoylglycine | 0.1072 | 2.0700E-06 | 1.4785E-04 | 0.9990 |
| DG(20:0/18:2) | FA(20:4) | 0.0912 | 5.5100E-05 | 2.4584E-03 | 0.9865 | L-homoserine | Nicotinoylglycine | -0.0908 | 5.8400E-05 | 2.5771E-03 | 0.9865 |
| DG(20:0/18:3) | DG(20:1/18:3) | 0.1587 | 1.7700E-12 | 3.2200E-10 | 1.0000 | Inositol | Nicotinoylglycine | 0.1721 | 1.9500E-14 | 4.1600E-12 | 1.0000 |
| DG(20:0/18:3) | DG(22:0/18:2) | 0.1913 | 2.2200E-16 | 5.3200E-14 | 1.0000 | β-Sitosterol | oleic acid | -0.0980 | 1.4400E-05 | 8.0347E-04 | 0.9940 |
| DG(20:0/18:3) | CerG1(d18:2/16:0 | 0.1087 | 1.4900E-06 | 1.1081E-04 | 0.9991 | 2-Aminophenol | oleic acid | -0.1347 | 2.2800E-09 | 2.8500E-07 | 1.0000 |
| DG(20:0/18:3) | CerG1(d18:2/22:0) | -0.1130 | 5.5200E-07 | 4.5600E-05 | 0.9996 | 4-Acetamidobutanoate | oleic acid | -0.0925 | 4.2500E-05 | 1.9806E-03 | 0.9881 |
| DG(20:0/18:3) | LPC(18:1) | -0.0982 | 1.3800E-05 | 7.7371E-04 | 0.9947 | Biphenyl | oleic acid | 0.1006 | 8.4600E-06 | 5.0868E-04 | 0.9961 |
| DG(20:1/18:2) | DG(20:1/18:3) | 0.2227 | 2.2200E-16 | 5.3200E-14 | 1.0000 | Biuret | oleic acid | 0.1932 | 2.2200E-16 | 5.3200E-14 | 1.0000 |
| DG(20:1/18:3) | DG(22:0/18:2) | 0.0926 | 4.2200E-05 | 1.9669E-03 | 0.9895 | D-Glucose | oleic acid | 0.1195 | 1.1800E-07 | 1.1300E-05 | 0.9999 |
| DG(22:0/18:2) | DG(24:0/18:2) | 0.1734 | 1.2200E-14 | 2.6400E-12 | 1.0000 | indole-3-acetamide | oleic acid | -0.1086 | 1.5300E-06 | 1.1323E-04 | 0.9991 |
| DG(22:0/18:2) | CerG1(d18:2/22:1) | 0.1411 | 3.8700E-10 | 5.3000E-08 | 1.0000 | N-Methylhydantoin | oxalacetic acid | 0.0947 | 2.7600E-05 | 1.3829E-03 | 0.9933 |
| DG(22:0/18:2) | CerG1(d24:0/18:1) | -0.1225 | 5.7000E-08 | 5.7300E-06 | 0.9999 | alpha-D-glucosamine 1-phosphate | oxalacetic acid | 0.0991 | 1.1500E-05 | 6.6390E-04 | 0.9953 |
| DG(22:0/18:2) | PE(22:0/18:2) | 0.1194 | 1.2100E-07 | 1.1500E-05 | 0.9999 | Biuret | oxalacetic acid | -0.0961 | 2.1100E-05 | 1.1101E-03 | 0.9933 |
| DG(24:0/18:2) | CerG1(d18:2/22:1) | -0.1627 | 4.7200E-13 | 9.0600E-11 | 1.0000 | glutamine | oxalacetic acid | -0.0985 | 1.3000E-05 | 7.3500E-04 | 0.9953 |
| DG(24:0/18:2) | CerG1(d24:0/18:1) | 0.2382 | 2.2200E-16 | 5.3200E-14 | 1.0000 | Glutaric Acid | oxalacetic acid | -0.0925 | 4.2200E-05 | 1.9679E-03 | 0.9889 |
| DG(24:0/18:2) | PE(24:0/18:2) | 0.1049 | 3.3900E-06 | 2.2826E-04 | 0.9983 | N-Carbamyl-L-glutamate | oxalacetic acid | -0.1309 | 6.4800E-09 | 7.6400E-07 | 1.0000 |
| DG(24:0/18:2) | 3-Phenylcatechol | -0.0928 | 4.0400E-05 | 1.9010E-03 | 0.9895 | N-formyl-L-methionine | oxalacetic acid | 0.1305 | 7.2500E-09 | 8.4800E-07 | 1.0000 |
| Cer(d18:2/16:0 | Cer(d18:2/16:1) | 0.2184 | 2.2200E-16 | 5.3200E-14 | 1.0000 | oleic acid | oxalacetic acid | -0.0961 | 2.1200E-05 | 1.1184E-03 | 0.9933 |
| Cer(d18:2/16:0 | CerG1(d18:2/16:0 | 0.1279 | 1.4400E-08 | 1.6000E-06 | 1.0000 | 1-Hexadecanol | oxalic acid | 0.0946 | 2.8600E-05 | 1.4222E-03 | 0.9933 |
| Cer(d18:2/16:0 | CerG1(d18:2/16:1) | 0.1964 | 2.2200E-16 | 5.3200E-14 | 1.0000 | coniferyl alcohol | oxalic acid | 0.1074 | 1.9800E-06 | 1.4180E-04 | 0.9990 |
| Cer(d18:2/16:0 | CerG1(d18:2/22:1) | 0.1222 | 6.0500E-08 | 6.0400E-06 | 0.9999 | linolenic acid | oxalic acid | -0.1003 | 8.9900E-06 | 5.3587E-04 | 0.9961 |
| Cer(d18:2/16:0 | CerG1(d24:0/18:1) | 0.1185 | 1.5000E-07 | 1.4000E-05 | 0.9999 | Methyl jasmonate | oxalic acid | 0.0945 | 2.9200E-05 | 1.4476E-03 | 0.9933 |
| Cer(d18:2/16:0 | PG(16:0/16:0) | 0.0909 | 5.8000E-05 | 2.5635E-03 | 0.9865 | N-Methylhydantoin | oxoproline | 0.1160 | 2.7500E-07 | 2.4400E-05 | 0.9998 |
| Cer(d18:2/16:0 | Cer(d18:2/16:1) | 0.7479 | 2.2200E-16 | 5.3200E-14 | 1.0000 | 2-Aminophenol | oxoproline | 0.1045 | 3.7300E-06 | 2.4790E-04 | 0.9983 |
| Cer(d18:2/16:1) | CerG1(d18:2/16:0 | 0.1545 | 6.7300E-12 | 1.1500E-09 | 1.0000 | 5-Aminovaleric acid | oxoproline | 0.0929 | 3.9300E-05 | 1.8560E-03 | 0.9895 |
| Cer(d18:2/16:1) | CerG1(d18:2/16:1) | 0.3577 | 2.2200E-16 | 5.3200E-14 | 1.0000 | D-Glucose | oxoproline | 0.1319 | 4.9900E-09 | 5.9800E-07 | 1.0000 |
| Cer(d18:2/16:1) | CerG1(d18:2/22:1) | 0.2049 | 2.2200E-16 | 5.3200E-14 | 1.0000 | glycerol | oxoproline | -0.1271 | 1.7600E-08 | 1.9200E-06 | 1.0000 |
| CerG1(d18:2/16:0 | CerG1(d18:2/16:1) | 0.1851 | 2.2200E-16 | 5.3200E-14 | 1.0000 | L-homoserine | oxoproline | 0.1104 | 1.0100E-06 | 7.8100E-05 | 0.9994 |
| CerG1(d18:2/16:0 | CerG1(d18:2/22:1) | 0.1507 | 2.2100E-11 | 3.6000E-09 | 1.0000 | Maleamate | oxoproline | 0.0912 | 5.4200E-05 | 2.4264E-03 | 0.9865 |
| CerG1(d18:2/16:0 | CerG1(d24:0/18:1) | 0.0912 | 5.4400E-05 | 2.4324E-03 | 0.9865 | β-Sitosterol | palmitic acid | -0.1160 | 2.7400E-07 | 2.4300E-05 | 0.9998 |
| CerG1(d18:2/16:0 | LPC(18:0) | -0.0992 | 1.1300E-05 | 6.5088E-04 | 0.9953 | Abietic Acid | palmitic acid | 0.1050 | 3.3100E-06 | 2.2350E-04 | 0.9983 |
| CerG1(d18:2/16:0 | FA(28:4) | -0.1097 | 1.1700E-06 | 8.9500E-05 | 0.9991 | Cytidine-5'-monophosphate(CMP) | palmitic acid | -0.1283 | 1.2700E-08 | 1.4300E-06 | 1.0000 |
| CerG1(d18:2/16:1) | CerG1(d18:2/22:1) | 0.1840 | 2.2200E-16 | 5.3200E-14 | 1.0000 | dibenzofuran | palmitic acid | 0.1378 | 9.7300E-10 | 1.2700E-07 | 1.0000 |
| CerG1(d18:2/22:0) | CerG1(d18:2/22:1) | 0.2656 | 2.2200E-16 | 5.3200E-14 | 1.0000 | glycolic acid | palmitic acid | 0.1281 | 1.3500E-08 | 1.5100E-06 | 1.0000 |
| CerG1(d18:2/22:0) | CerG1(d24:0/18:1) | 0.1137 | 4.7300E-07 | 3.9700E-05 | 0.9997 | L-homoserine | palmitic acid | 0.1076 | 1.9000E-06 | 1.3688E-04 | 0.9990 |
| CerG1(d18:2/22:0) | LPC(18:0) | 0.1009 | 7.9900E-06 | 4.8398E-04 | 0.9961 | linolenic acid | palmitic acid | 0.0975 | 1.5900E-05 | 8.7419E-04 | 0.9940 |
| CerG1(d18:2/22:0) | LPC(18:2) | 0.0954 | 2.4000E-05 | 1.2377E-03 | 0.9933 | malonic acid | palmitic acid | 0.1039 | 4.1900E-06 | 2.7377E-04 | 0.9983 |
| CerG1(d18:2/22:0) | PE(18:1/22:0) | 0.1072 | 2.0600E-06 | 1.4717E-04 | 0.9990 | N-Carbamyl-L-glutamate | palmitic acid | 0.1477 | 5.5300E-11 | 8.5800E-09 | 1.0000 |
| CerG1(d18:2/22:0) | PE(18:1/24:0) | -0.1091 | 1.3500E-06 | 1.0132E-04 | 0.9991 | oxoproline | palmitic acid | 0.0986 | 1.2700E-05 | 7.2148E-04 | 0.9953 |
| CerG1(d18:2/22:0) | PI(16:0/18:1) | -0.0934 | 3.5500E-05 | 1.7059E-03 | 0.9895 | 3-Methyl-2-oxobutanoic acid | palmitoleic acid | -0.1149 | 3.5900E-07 | 3.1100E-05 | 0.9997 |
| CerG1(d18:2/22:0) | PI(16:0/18:3) | -0.0992 | 1.1400E-05 | 6.5819E-04 | 0.9953 | 3-Methylbenzyl Alcohol | palmitoleic acid | 0.0980 | 1.4300E-05 | 7.9745E-04 | 0.9940 |
| CerG1(d18:2/22:0) | leucine | 0.0935 | 3.5000E-05 | 1.6857E-03 | 0.9895 | Octanoic acid | palmitoleic acid | 0.1313 | 5.7200E-09 | 6.8000E-07 | 1.0000 |
| CerG1(d18:2/22:1) | PE(18:1/22:0) | 0.1358 | 1.6800E-09 | 2.1400E-07 | 1.0000 | glutamine | palmitoleic acid | 0.1115 | 7.8000E-07 | 6.2400E-05 | 0.9994 |
| CerG1(d18:2/22:1) | PE(18:1/24:0) | -0.1244 | 3.4800E-08 | 3.6200E-06 | 0.9999 | nicotinamide | palmitoleic acid | -0.0941 | 3.1500E-05 | 1.5442E-03 | 0.9895 |
| CerG1(d18:2/22:1) | PE(22:0/18:2) | 0.1277 | 1.5100E-08 | 1.6700E-06 | 1.0000 | oxoproline | palmitoleic acid | 0.1038 | 4.3600E-06 | 2.8367E-04 | 0.9975 |
| CerG1(d24:0/18:1) | PC(18:1/18:2) | 0.0986 | 1.2700E-05 | 7.2134E-04 | 0.9953 | palmitic acid | palmitoleic acid | -0.1146 | 3.8600E-07 | 3.3100E-05 | 0.9997 |
| CerG1(d24:0/18:1) | PE(18:1/24:0) | 0.1002 | 9.2900E-06 | 5.5102E-04 | 0.9961 | 3-Methylbenzyl Alcohol | panthenol | 0.2266 | 2.2200E-16 | 5.3200E-14 | 1.0000 |
| CerG1(d24:0/18:1) | PI(16:0/18:3) | 0.1091 | 1.3600E-06 | 1.0238E-04 | 0.9991 | leucine | panthenol | -0.0908 | 5.8700E-05 | 2.5870E-03 | 0.9865 |
| LPC(16:0) | LPC(18:0) | 0.4411 | 2.2200E-16 | 5.3200E-14 | 1.0000 | oleic acid | panthenol | 0.1300 | 8.1800E-09 | 9.4900E-07 | 1.0000 |
| LPC(16:0) | LPC(18:2) | -0.1432 | 2.0700E-10 | 2.9600E-08 | 1.0000 | oxoproline | panthenol | 0.2957 | 2.2200E-16 | 5.3200E-14 | 1.0000 |
| LPC(16:0) | LPC(18:2) | -0.1364 | 1.4400E-09 | 1.8500E-07 | 1.0000 | palmitic acid | panthenol | -0.2780 | 2.2200E-16 | 5.3200E-14 | 1.0000 |
| LPC(16:0) | L-2,4-Diaminobutanoate | 0.0908 | 5.8600E-05 | 2.5861E-03 | 0.9865 | palmitoleic acid | panthenol | 0.2673 | 2.2200E-16 | 5.3200E-14 | 1.0000 |
| LPC(18:0) | LPC(18:2) | -0.1693 | 5.0200E-14 | 1.0400E-11 | 1.0000 | 2-Oxoadipate | phenylacetaldehyde | 0.1229 | 5.1700E-08 | 5.2400E-06 | 0.9999 |
| LPC(18:0) | LPE(18:2) | -0.0974 | 1.6300E-05 | 8.9354E-04 | 0.9940 | 19-Hydroxyandrost-4-ene-3,17-dione | phenylacetaldehyde | 0.0919 | 4.7500E-05 | 2.1748E-03 | 0.9865 |
| LPC(18:0) | PE(18:1/24:0) | -0.0949 | 2.6600E-05 | 1.3430E-03 | 0.9933 | fumaric acid | phenylacetaldehyde | -0.1146 | 3.8500E-07 | 3.3100E-05 | 0.9997 |
| LPC(18:1) | LPC(18:2) | -0.1309 | 6.4200E-09 | 7.5700E-07 | 1.0000 | glycerol | phenylacetaldehyde | 0.0908 | 5.8900E-05 | 2.5944E-03 | 0.9865 |
| LPC(18:1) | LPC(18:2) | -0.1053 | 3.1600E-06 | 2.1464E-04 | 0.9983 | N-Acetyl-beta-alanine | phenylacetaldehyde | 0.1691 | 5.4600E-14 | 1.1300E-11 | 1.0000 |
| LPC(18:2) | LPC(18:2) | -0.2203 | 2.2200E-16 | 5.3200E-14 | 1.0000 | palmitic acid | phenylacetaldehyde | -0.1011 | 7.6000E-06 | 4.6282E-04 | 0.9967 |
| LPC(18:2) | PG(16:0/18:2) | -0.1165 | 2.4600E-07 | 2.2000E-05 | 0.9998 | 2-Oxoadipate | Phosphate | -0.1235 | 4.3900E-08 | 4.5100E-06 | 0.9999 |
| LPC(18:2) | FA(20:6) | 0.1692 | 5.3500E-14 | 1.1000E-11 | 1.0000 | 4-Aminobutyric acid(GABA) | Phosphate | 0.0952 | 2.5400E-05 | 1.2963E-03 | 0.9933 |
| LPC(18:2) | FA(28:4) | -0.1012 | 7.4900E-06 | 4.5740E-04 | 0.9967 | hydroxylamine | Phosphate | 0.0908 | 5.8800E-05 | 2.5935E-03 | 0.9865 |
| LPC(18:2) | 2-Oxoadipate | 0.1003 | 9.0900E-06 | 5.4104E-04 | 0.9961 | N-Acetyl-beta-alanine | Phosphate | 0.1039 | 4.2000E-06 | 2.7420E-04 | 0.9983 |
| LPC(18:2) | D-Glyceric acid | 0.0920 | 4.6700E-05 | 2.1425E-03 | 0.9865 | palmitic acid | Phosphate | 0.0962 | 2.0800E-05 | 1.0975E-03 | 0.9933 |
| LPC(18:2) | palmitoleic acid | -0.0994 | 1.0800E-05 | 6.2884E-04 | 0.9953 | phenylacetaldehyde | Phosphate | 0.1702 | 3.6900E-14 | 7.7200E-12 | 1.0000 |
| LPC(18:2) | LPE(18:2) | 0.2397 | 2.2200E-16 | 5.3200E-14 | 1.0000 | adenine | phosphomycin | -0.0947 | 2.8100E-05 | 1.4025E-03 | 0.9933 |
| LPC(18:2) | FA(21:6) | -0.1011 | 7.6400E-06 | 4.6514E-04 | 0.9967 | Icosenoic acid | phosphomycin | 0.0928 | 4.0000E-05 | 1.8830E-03 | 0.9895 |
| LPC(18:2) | Succinate semialdehyde | 0.1041 | 4.0700E-06 | 2.6692E-04 | 0.9983 | Glucose-1-phosphate | phosphomycin | -0.1012 | 7.4500E-06 | 4.5526E-04 | 0.9967 |
| CL(18:2/18:1/18:1/18:2) | CL(18:2/18:2/18:2/18:1) | 0.1885 | 2.2200E-16 | 5.3200E-14 | 1.0000 | glutamine | phosphomycin | -0.0964 | 2.0000E-05 | 1.0648E-03 | 0.9933 |
| CL(18:2/18:1/18:1/18:2) | CL(18:2/18:2/18:2/18:2) | -0.2205 | 2.2200E-16 | 5.3200E-14 | 1.0000 | isocitric acid | phosphomycin | 0.1082 | 1.6400E-06 | 1.2063E-04 | 0.9990 |
| CL(18:2/18:1/18:1/18:2) | CL(18:3/18:2/18:2/18:2) | -0.2796 | 2.2200E-16 | 5.3200E-14 | 1.0000 | L-homoserine | phosphomycin | -0.0976 | 1.5500E-05 | 8.5758E-04 | 0.9940 |
| CL(18:2/18:1/18:1/18:2) | DGDG(16:0/18:2) | 0.1930 | 2.2200E-16 | 5.3200E-14 | 1.0000 | mannose | phosphomycin | 0.1506 | 2.2600E-11 | 3.6800E-09 | 1.0000 |
| CL(18:2/18:1/18:1/18:2) | LPE(16:0) | 0.0958 | 2.2300E-05 | 1.1664E-03 | 0.9933 | N-Carbamyl-L-glutamate | phosphomycin | -0.0975 | 1.5800E-05 | 8.6983E-04 | 0.9940 |
| CL(18:2/18:1/18:1/18:2) | OAHFA(16:0/22:3) | 0.1230 | 4.9500E-08 | 5.0300E-06 | 0.9999 | Nicotinoylglycine | phosphomycin | 0.1239 | 4.0300E-08 | 4.1600E-06 | 0.9999 |
| CL(18:2/18:1/18:1/18:2) | OAHFA(18:0/22:3) | 0.1200 | 1.0400E-07 | 1.0000E-05 | 0.9999 | oxalacetic acid | phosphomycin | -0.1239 | 3.9800E-08 | 4.1100E-06 | 0.9999 |
| CL(18:2/18:1/18:1/18:2) | SQDG(16:0/18:1) | 0.1270 | 1.7800E-08 | 1.9400E-06 | 1.0000 | palmitoleic acid | phosphomycin | 0.1022 | 6.1100E-06 | 3.8286E-04 | 0.9967 |
| CL(18:2/18:2/18:2/18:1) | CL(18:3/18:2/18:2/18:2) | -0.1555 | 4.9500E-12 | 8.6300E-10 | 1.0000 | 3-Methylbenzyl Alcohol | piceatannol | 0.1126 | 6.1700E-07 | 5.0500E-05 | 0.9994 |
| CL(18:2/18:2/18:2/18:1) | DGDG(16:0/18:2) | 0.1451 | 1.1800E-10 | 1.7400E-08 | 1.0000 | oxoproline | piceatannol | -0.1077 | 1.8400E-06 | 1.3341E-04 | 0.9990 |
| CL(18:2/18:2/18:2/18:1) | DGDG(16:0/18:3) | 0.0951 | 2.5600E-05 | 1.3029E-03 | 0.9933 | 2-Hydroxybutanoic acid | picolinic acid | 0.1057 | 2.8900E-06 | 1.9889E-04 | 0.9983 |
| CL(18:2/18:2/18:2/18:1) | OAHFA(16:0/22:3) | 0.0933 | 3.6300E-05 | 1.7381E-03 | 0.9895 | Biuret | picolinic acid | 0.0955 | 2.3600E-05 | 1.2193E-03 | 0.9933 |
| CL(18:2/18:2/18:2/18:1) | OAHFA(18:0/22:3) | 0.1040 | 4.1500E-06 | 2.7131E-04 | 0.9983 | D-Glucose | picolinic acid | 0.1112 | 8.3800E-07 | 6.6500E-05 | 0.9994 |
| CL(18:2/18:2/18:2/18:1) | OAHFA(18:1/18:0) | 0.1004 | 8.8900E-06 | 5.3053E-04 | 0.9961 | Nicotinoylglycine | picolinic acid | 0.1260 | 2.3400E-08 | 2.5000E-06 | 1.0000 |
| CL(18:2/18:2/18:2/18:1) | SQDG(16:0/18:1) | 0.1003 | 8.9800E-06 | 5.3540E-04 | 0.9961 | Acetol | proline | -0.0984 | 1.3200E-05 | 7.4371E-04 | 0.9953 |
| CL(18:2/18:2/18:2/18:2) | CL(18:3/18:2/18:2/18:2) | 0.1030 | 5.1600E-06 | 3.2894E-04 | 0.9967 | L-Malic acid | proline | 0.1003 | 9.0300E-06 | 5.3801E-04 | 0.9961 |
| CL(18:2/18:2/18:2/18:2) | DGDG(16:0/18:2) | 0.1101 | 1.0900E-06 | 8.3800E-05 | 0.9991 | phenylacetaldehyde | proline | 0.1524 | 1.3000E-11 | 2.1700E-09 | 1.0000 |
| CL(18:2/18:2/18:2/18:2) | DGDG(18:3/18:3) | 0.1043 | 3.9100E-06 | 2.5834E-04 | 0.9983 | benzoic acid | prostaglandin A2 | 0.1227 | 5.4500E-08 | 5.4900E-06 | 0.9999 |
| CL(18:2/18:2/18:2/18:2) | LPC(16:0) | 0.0938 | 3.2900E-05 | 1.5993E-03 | 0.9895 | cholic acid | prostaglandin A2 | 0.0916 | 5.1000E-05 | 2.3085E-03 | 0.9865 |
| CL(18:2/18:2/18:2/18:2) | LPC(18:1) | 0.0915 | 5.1100E-05 | 2.3121E-03 | 0.9865 | fumaric acid | prostaglandin A2 | 0.0988 | 1.2300E-05 | 6.9981E-04 | 0.9953 |
| CL(18:2/18:2/18:2/18:2) | LPE(16:0) | 0.1337 | 3.0600E-09 | 3.7600E-07 | 1.0000 | Glucose-6-phosphate | prostaglandin A2 | 0.0933 | 3.6500E-05 | 1.7459E-03 | 0.9895 |
| CL(18:2/18:2/18:2/18:2) | OAHFA(18:0/22:3) | 0.1102 | 1.0600E-06 | 8.1800E-05 | 0.9994 | indole-3-acetamide | prostaglandin A2 | 0.0943 | 3.0400E-05 | 1.4942E-03 | 0.9895 |
| CL(18:2/18:2/18:2/18:2) | OAHFA(18:1/18:0) | 0.1134 | 5.1200E-07 | 4.2600E-05 | 0.9996 | isocitric acid | prostaglandin A2 | -0.0941 | 3.1500E-05 | 1.5444E-03 | 0.9895 |
| CL(18:2/18:2/18:2/18:2) | SQDG(16:0/18:1) | 0.1108 | 9.1500E-07 | 7.1900E-05 | 0.9994 | nicotinamide | prostaglandin A2 | 0.1088 | 1.4400E-06 | 1.0718E-04 | 0.9991 |
| CL(18:2/18:2/18:2/18:2) | SQDG(16:0/18:2) | 0.1007 | 8.2400E-06 | 4.9681E-04 | 0.9961 | oxalic acid | prostaglandin A2 | 0.2557 | 2.2200E-16 | 5.3200E-14 | 1.0000 |
| CL(18:3/18:2/18:2/18:2) | DGDG(18:3/18:3) | 0.2303 | 2.2200E-16 | 5.3200E-14 | 1.0000 | 3-Hydroxyphenylacetic acid | putrescine | -0.1319 | 4.8900E-09 | 5.8600E-07 | 1.0000 |
| CL(18:3/18:2/18:2/18:2) | LPE(18:1) | 0.0987 | 1.2500E-05 | 7.1269E-04 | 0.9953 | Atropine | putrescine | -0.1137 | 4.7600E-07 | 4.0000E-05 | 0.9997 |
| CL(18:3/18:2/18:2/18:2) | LPE(18:2) | 0.1344 | 2.5100E-09 | 3.1200E-07 | 1.0000 | palmitic acid | putrescine | -0.1045 | 3.7200E-06 | 2.4773E-04 | 0.9983 |
| CL(18:3/18:2/18:2/18:2) | OAHFA(16:0/22:3) | 0.1059 | 2.7500E-06 | 1.9025E-04 | 0.9984 | L-2,4-Diaminobutanoate | Pyrophosphate | 0.0921 | 4.6200E-05 | 2.1241E-03 | 0.9865 |
| CL(18:3/18:2/18:2/18:2) | OAHFA(18:1/18:0) | 0.1002 | 9.1600E-06 | 5.4437E-04 | 0.9961 | 3-Methylbenzyl Alcohol | Pyrophosphate | 0.1748 | 7.3300E-15 | 1.6200E-12 | 1.0000 |
| CL(18:3/18:2/18:2/18:2) | SQDG(16:0/18:2) | 0.1160 | 2.7400E-07 | 2.4300E-05 | 0.9998 | Atropine | Pyrophosphate | -0.1004 | 8.8700E-06 | 5.2966E-04 | 0.9961 |
| CL(18:3/18:2/18:2/18:2) | SQDG(16:0/18:3) | 0.1015 | 7.0400E-06 | 4.3325E-04 | 0.9967 | Biphenyl | Pyrophosphate | 0.1102 | 1.0700E-06 | 8.2300E-05 | 0.9994 |
| DGDG(16:0/18:2) | DGDG(16:0/18:3) | -0.1377 | 1.0100E-09 | 1.3200E-07 | 1.0000 | D-Fructose 2,6-bisphosphate | Pyrophosphate | 0.1149 | 3.5700E-07 | 3.0900E-05 | 0.9997 |
| DGDG(16:0/18:2) | DGDG(18:3/18:3) | -0.5977 | 2.2200E-16 | 5.3200E-14 | 1.0000 | Geraniol | Pyrophosphate | -0.1864 | 2.2200E-16 | 5.3200E-14 | 1.0000 |
| DGDG(16:0/18:2) | LPC(16:0) | 0.1071 | 2.1000E-06 | 1.4945E-04 | 0.9988 | L-Malic acid | Pyrophosphate | 0.0978 | 1.5100E-05 | 8.3730E-04 | 0.9940 |
| DGDG(16:0/18:2) | LPE(16:0) | 0.1175 | 1.9200E-07 | 1.7500E-05 | 0.9998 | Menthone | Pyrophosphate | 0.1133 | 5.2600E-07 | 4.3700E-05 | 0.9996 |
| DGDG(16:0/18:2) | LPE(18:2) | 0.1019 | 6.5000E-06 | 4.0400E-04 | 0.9967 | Inositol | Pyrophosphate | 0.1219 | 6.5700E-08 | 6.5100E-06 | 0.9999 |
| DGDG(16:0/18:2) | OAHFA(18:1/18:0) | 0.1282 | 1.3000E-08 | 1.4600E-06 | 1.0000 | palmitic acid | Pyrophosphate | 0.1060 | 2.6800E-06 | 1.8585E-04 | 0.9984 |
| DGDG(16:0/18:2) | SQDG(16:0/18:1) | 0.1117 | 7.4500E-07 | 5.9900E-05 | 0.9994 | panthenol | Pyrophosphate | 0.1187 | 1.4400E-07 | 1.3500E-05 | 0.9999 |
| DGDG(16:0/18:2) | SQDG(16:0/18:2) | 0.0966 | 1.8900E-05 | 1.0146E-03 | 0.9933 | piceatannol | Pyrophosphate | -0.1018 | 6.6400E-06 | 4.1175E-04 | 0.9967 |
| DGDG(16:0/18:3) | DGDG(18:3/18:3) | -0.0924 | 4.3700E-05 | 2.0274E-03 | 0.9865 | putrescine | Pyrophosphate | 0.2761 | 2.2200E-16 | 5.3200E-14 | 1.0000 |
| DGDG(16:0/18:3) | LPE(18:1) | 0.0910 | 5.6500E-05 | 2.5106E-03 | 0.9865 | Sphingosine | Pyruvate | -0.0993 | 1.1100E-05 | 6.4541E-04 | 0.9953 |
| DGDG(16:0/18:3) | OAHFA(18:0/22:3) | 0.1024 | 5.8200E-06 | 3.6658E-04 | 0.9967 | N-Carbamyl-L-glutamate | Pyruvate | 0.0963 | 2.0300E-05 | 1.0762E-03 | 0.9933 |
| DGDG(16:0/18:3) | SQDG(16:0/18:1) | 0.0952 | 2.5000E-05 | 1.2799E-03 | 0.9933 | oxalic acid | Pyruvate | 0.1052 | 3.2200E-06 | 2.1820E-04 | 0.9983 |
| DGDG(18:3/18:3) | LPE(16:0) | 0.0990 | 1.1700E-05 | 6.7232E-04 | 0.9953 | prostaglandin A2 | Pyruvate | 0.1425 | 2.5600E-10 | 3.6100E-08 | 1.0000 |
| DGDG(18:3/18:3) | OAHFA(16:0/22:3) | 0.1222 | 6.1500E-08 | 6.1300E-06 | 0.9999 | 6-Hydroxynicotinic acid | Glucaric acid | -0.0918 | 4.8600E-05 | 2.2151E-03 | 0.9865 |
| DGDG(18:3/18:3) | OAHFA(18:0/22:3) | 0.1156 | 3.0600E-07 | 2.6800E-05 | 0.9997 | adenine | Glucaric acid | 0.0950 | 2.6200E-05 | 1.3288E-03 | 0.9933 |
| DGDG(18:3/18:3) | OAHFA(18:1/18:0) | 0.1029 | 5.2000E-06 | 3.3132E-04 | 0.9967 | mannose | Glucaric acid | -0.1035 | 4.6500E-06 | 2.9993E-04 | 0.9975 |
| DGDG(18:3/18:3) | SQDG(16:0/18:1) | 0.1062 | 2.5600E-06 | 1.7859E-04 | 0.9984 | Inositol | Glucaric acid | -0.1496 | 3.0600E-11 | 4.9100E-09 | 1.0000 |
| LPC(16:0) | LPC(18:1) | -0.2689 | 2.2200E-16 | 5.3200E-14 | 1.0000 | phosphomycin | Glucaric acid | 0.1036 | 4.4700E-06 | 2.9005E-04 | 0.9975 |
| LPC(16:0) | LPE(16:0) | 0.2364 | 2.2200E-16 | 5.3200E-14 | 1.0000 | piceatannol | Glucaric acid | 0.1046 | 3.6200E-06 | 2.4162E-04 | 0.9983 |
| LPC(16:0) | LPE(18:1) | -0.1328 | 3.9200E-09 | 4.7500E-07 | 1.0000 | 3-Methylbenzyl Alcohol | sarcosine | 0.0946 | 2.8600E-05 | 1.4247E-03 | 0.9933 |
| LPC(16:0) | OAHFA(18:0/22:3) | 0.1011 | 7.5900E-06 | 4.6237E-04 | 0.9967 | Atrazine-2-hydroxy | sarcosine | 0.1021 | 6.2500E-06 | 3.9047E-04 | 0.9967 |
| LPC(16:0) | OAHFA(18:1/18:0) | 0.1041 | 4.0600E-06 | 2.6632E-04 | 0.9983 | oxoproline | sarcosine | 0.1059 | 2.7600E-06 | 1.9049E-04 | 0.9983 |
| LPC(16:0) | SQDG(16:0/18:2) | 0.1507 | 2.2300E-11 | 3.6400E-09 | 1.0000 | indole-3-acetamide | serine | 0.0908 | 5.9000E-05 | 2.5979E-03 | 0.9865 |
| LPC(18:1) | LPE(18:1) | 0.2467 | 2.2200E-16 | 5.3200E-14 | 1.0000 | Nicotinoylglycine | serine | 0.1262 | 2.2300E-08 | 2.3800E-06 | 1.0000 |
| LPC(18:1) | LPE(18:2) | 0.0915 | 5.1600E-05 | 2.3303E-03 | 0.9865 | oleic acid | serine | 0.1130 | 5.6300E-07 | 4.6400E-05 | 0.9994 |
| LPC(18:1) | SQDG(16:0/18:1) | 0.1092 | 1.3300E-06 | 9.9900E-05 | 0.9991 | oxoproline | serine | 0.1116 | 7.7200E-07 | 6.1900E-05 | 0.9994 |
| LPC(18:1) | SQDG(16:0/18:3) | 0.1258 | 2.4500E-08 | 2.6100E-06 | 1.0000 | Pyrophosphate | serine | 0.2123 | 2.2200E-16 | 5.3200E-14 | 1.0000 |
| LPE(16:0) | LPE(18:1) | -0.2526 | 2.2200E-16 | 5.3200E-14 | 1.0000 | 4-Hydroxyphenylethanol | sorbose | -0.1381 | 9.0700E-10 | 1.1900E-07 | 1.0000 |
| LPE(16:0) | LPE(18:2) | -0.3067 | 2.2200E-16 | 5.3200E-14 | 1.0000 | alanine | sorbose | 0.0911 | 5.5900E-05 | 2.4870E-03 | 0.9865 |
| LPE(16:0) | OAHFA(16:0/22:3) | 0.1152 | 3.3400E-07 | 2.9100E-05 | 0.9997 | citric acid | sorbose | 0.0985 | 1.2900E-05 | 7.3282E-04 | 0.9953 |
| LPE(16:0) | OAHFA(18:1/18:0) | 0.1322 | 4.5000E-09 | 5.4300E-07 | 1.0000 | dibenzofuran | sorbose | 0.0969 | 1.8000E-05 | 9.7328E-04 | 0.9933 |
| LPE(16:0) | SQDG(16:0/18:1) | 0.1167 | 2.3200E-07 | 2.0900E-05 | 0.9998 | Ethanolamine | sorbose | 0.0990 | 1.1900E-05 | 6.8033E-04 | 0.9953 |
| LPE(16:0) | SQDG(16:0/18:2) | 0.1140 | 4.4200E-07 | 3.7400E-05 | 0.9997 | D-Fructose 2,6-bisphosphate | sorbose | -0.0910 | 5.6200E-05 | 2.4969E-03 | 0.9865 |
| LPE(18:1) | LPE(18:2) | -0.2204 | 2.2200E-16 | 5.3200E-14 | 1.0000 | hydroxylamine | sorbose | 0.0991 | 1.1600E-05 | 6.6967E-04 | 0.9953 |
| LPE(18:1) | OAHFA(18:0/22:3) | 0.1170 | 2.2000E-07 | 1.9900E-05 | 0.9998 | 1-Hexadecanol | squalene | 0.1269 | 1.8400E-08 | 2.0000E-06 | 1.0000 |
| LPE(18:1) | PG(18:0/18:1) | 0.1039 | 4.2000E-06 | 2.7433E-04 | 0.9977 | alpha-ketoisocaproic acid | squalene | -0.0939 | 3.2800E-05 | 1.5950E-03 | 0.9895 |
| LPE(18:1) | SQDG(16:0/18:1) | 0.1138 | 4.5800E-07 | 3.8600E-05 | 0.9997 | Behenic acid | squalene | -0.0943 | 3.0300E-05 | 1.4937E-03 | 0.9895 |
| LPE(18:1) | FA(19:1) | 0.1074 | 1.9600E-06 | 1.4067E-04 | 0.9990 | citrulline | squalene | -0.0912 | 5.4400E-05 | 2.4325E-03 | 0.9865 |
| LPE(18:2) | OAHFA(16:0/22:3) | 0.0921 | 4.6000E-05 | 2.1180E-03 | 0.9865 | L-kynurenine | squalene | 0.1187 | 1.4400E-07 | 1.3400E-05 | 0.9999 |
| LPE(18:2) | OAHFA(18:0/22:3) | 0.1073 | 2.0300E-06 | 1.4490E-04 | 0.9990 | nicotinamide | squalene | -0.0906 | 6.1300E-05 | 2.6843E-03 | 0.9833 |
| LPE(18:2) | SQDG(16:0/18:3) | 0.1551 | 5.5600E-12 | 9.6200E-10 | 1.0000 | piceatannol | squalene | 0.1911 | 2.2200E-16 | 5.3200E-14 | 1.0000 |
| LPE(18:2) | FA(18:2) | 0.1063 | 2.5100E-06 | 1.7508E-04 | 0.9984 | 5-Aminovaleric acid | stearic acid | 0.1115 | 7.8600E-07 | 6.2800E-05 | 0.9994 |
| LPE(18:2) | FA(18:4) | -0.1106 | 9.6500E-07 | 7.5300E-05 | 0.9994 | 5-aminovaleric acid lactam | stearic acid | 0.1015 | 6.9500E-06 | 4.2868E-04 | 0.9967 |
| LPE(18:2) | FA(20:2) | 0.1257 | 2.5100E-08 | 2.6700E-06 | 1.0000 | Cytidine-5'-monophosphate(CMP) | stearic acid | 0.0909 | 5.7400E-05 | 2.5438E-03 | 0.9865 |
| LPE(18:2) | FA(20:4) | -0.1096 | 1.2200E-06 | 9.2700E-05 | 0.9991 | Maleamate | Stigmasterol | 0.1028 | 5.3100E-06 | 3.3750E-04 | 0.9967 |
| OAHFA(16:0/22:3) | OAHFA(16:0/22:3) | -0.0943 | 2.9800E-05 | 1.4704E-03 | 0.9895 | malonic acid | Stigmasterol | 0.4250 | 2.2200E-16 | 5.3200E-14 | 1.0000 |
| OAHFA(16:0/22:3) | OAHFA(18:1/18:0) | -0.1985 | 2.2200E-16 | 5.3200E-14 | 1.0000 | nicotinamide | Stigmasterol | 0.2697 | 2.2200E-16 | 5.3200E-14 | 1.0000 |
| OAHFA(16:0/22:3) | FA(16:2) | 0.0915 | 5.1600E-05 | 2.3314E-03 | 0.9865 | palmitic acid | Stigmasterol | -0.0961 | 2.1100E-05 | 1.1129E-03 | 0.9933 |
| OAHFA(16:0/22:3) | 5-Aminovaleric acid | 0.1088 | 1.4300E-06 | 1.0697E-04 | 0.9991 | Pyruvate | Stigmasterol | 0.1163 | 2.5400E-07 | 2.2700E-05 | 0.9998 |
| OAHFA(16:0/22:3) | Biphenyl | 0.0963 | 2.0400E-05 | 1.0830E-03 | 0.9933 | sarcosine | Stigmasterol | 0.1763 | 4.2200E-15 | 9.5500E-13 | 1.0000 |
| OAHFA(16:0/22:3) | D-Glyceric acid | -0.1031 | 4.9800E-06 | 3.1853E-04 | 0.9975 | 5-aminovaleric acid lactam | Succinate semialdehyde | 0.1245 | 3.3900E-08 | 3.5400E-06 | 0.9999 |
| OAHFA(16:0/22:3) | Isoleucine | -0.0959 | 2.2000E-05 | 1.1527E-03 | 0.9933 | Abietic Acid | Succinate semialdehyde | -0.0981 | 1.4000E-05 | 7.8427E-04 | 0.9947 |
| OAHFA(16:0/22:3) | OAHFA(16:0/22:3) | -0.2564 | 2.2200E-16 | 5.3200E-14 | 1.0000 | Atrazine-2-hydroxy | Succinate semialdehyde | 0.1277 | 1.5000E-08 | 1.6600E-06 | 1.0000 |
| OAHFA(16:0/22:3) | OAHFA(16:0/22:3) | -0.1517 | 1.6300E-11 | 2.6900E-09 | 1.0000 | Atropine | Succinate semialdehyde | 0.1153 | 3.2500E-07 | 2.8400E-05 | 0.9997 |
| OAHFA(16:0/22:3) | OAHFA(18:0/22:3) | -0.0948 | 2.7200E-05 | 1.3701E-03 | 0.9933 | Octanoic acid | Succinate semialdehyde | 0.0967 | 1.8700E-05 | 1.0064E-03 | 0.9933 |
| OAHFA(16:0/22:3) | OAHFA(18:1/18:0) | -0.3216 | 2.2200E-16 | 5.3200E-14 | 1.0000 | linolenic acid | Succinate semialdehyde | 0.0970 | 1.7500E-05 | 9.5179E-04 | 0.9933 |
| OAHFA(16:0/22:3) | SQDG(16:0/18:3) | 0.0922 | 4.5300E-05 | 2.0878E-03 | 0.9865 | N-Acetyl-beta-alanine | Succinate semialdehyde | -0.0932 | 3.7100E-05 | 1.7696E-03 | 0.9895 |
| OAHFA(16:0/22:3) | FA(13:0) | 0.0987 | 1.2500E-05 | 7.1048E-04 | 0.9953 | serine | Succinate semialdehyde | -0.1344 | 2.5300E-09 | 3.1400E-07 | 1.0000 |
| OAHFA(16:0/22:3) | FA(16:2) | -0.1138 | 4.6600E-07 | 3.9200E-05 | 0.9997 | sorbose | Succinate semialdehyde | 0.0937 | 3.3700E-05 | 1.6340E-03 | 0.9895 |
| OAHFA(16:0/22:3) | FA(21:6) | 0.0948 | 2.7400E-05 | 1.3754E-03 | 0.9933 | stearic acid | Succinate semialdehyde | 0.1796 | 1.3300E-15 | 3.1200E-13 | 1.0000 |
| OAHFA(16:0/22:3) | OAHFA(16:0/22:3) | -0.1929 | 2.2200E-16 | 5.3200E-14 | 1.0000 | 5-aminovaleric acid lactam | succinic acid | 0.0936 | 3.4600E-05 | 1.6686E-03 | 0.9895 |
| OAHFA(16:0/22:3) | OAHFA(18:0/22:3) | -0.5236 | 2.2200E-16 | 5.3200E-14 | 1.0000 | Icosenoic acid | succinic acid | 0.1270 | 1.7900E-08 | 1.9400E-06 | 1.0000 |
| OAHFA(16:0/22:3) | OAHFA(18:1/18:0) | -0.5546 | 2.2200E-16 | 5.3200E-14 | 1.0000 | Creatine | succinic acid | 0.1055 | 3.0100E-06 | 2.0597E-04 | 0.9983 |
| OAHFA(16:0/22:3) | SQDG(16:0/18:1) | 0.0940 | 3.2100E-05 | 1.5693E-03 | 0.9895 | Sphingosine | succinic acid | 0.1211 | 7.9400E-08 | 7.7700E-06 | 0.9999 |
| OAHFA(16:0/22:3) | SQDG(16:0/18:3) | 0.0936 | 3.4200E-05 | 1.6528E-03 | 0.9895 | fumaric acid | succinic acid | -0.0945 | 2.8800E-05 | 1.4331E-03 | 0.9933 |
| OAHFA(16:0/22:3) | OAHFA(18:0/22:3) | -0.3479 | 2.2200E-16 | 5.3200E-14 | 1.0000 | Geraniol | succinic acid | 0.1067 | 2.2800E-06 | 1.6097E-04 | 0.9984 |
| OAHFA(16:0/22:3) | OAHFA(18:1/18:0) | -0.4372 | 2.2200E-16 | 5.3200E-14 | 1.0000 | linoleic acid | succinic acid | 0.1013 | 7.3900E-06 | 4.5181E-04 | 0.9967 |
| OAHFA(16:0/22:3) | FA(29:0) | -0.0909 | 5.8100E-05 | 2.5687E-03 | 0.9865 | linolenic acid | succinic acid | 0.0915 | 5.1100E-05 | 2.3106E-03 | 0.9865 |
| OAHFA(16:0/22:3) | alpha-ketoisocaproic acid | -0.0935 | 3.5500E-05 | 1.7054E-03 | 0.9895 | mannose | succinic acid | -0.1310 | 6.2400E-09 | 7.3800E-07 | 1.0000 |
| OAHFA(18:0/22:3) | OAHFA(18:1/18:0) | -0.5261 | 2.2200E-16 | 5.3200E-14 | 1.0000 | palmitic acid | succinic acid | 0.1332 | 3.4700E-09 | 4.2300E-07 | 1.0000 |
| OAHFA(18:0/22:3) | SQDG(16:0/18:2) | 0.1123 | 6.5700E-07 | 5.3400E-05 | 0.9994 | panthenol | succinic acid | 0.1559 | 4.2900E-12 | 7.5300E-10 | 1.0000 |
| OAHFA(18:0/22:3) | SQDG(16:0/18:3) | 0.1721 | 1.8900E-14 | 4.0200E-12 | 1.0000 | putrescine | succinic acid | 0.1597 | 1.2600E-12 | 2.3300E-10 | 1.0000 |
| OAHFA(18:1/18:0) | SQDG(16:0/18:1) | 0.1282 | 1.3000E-08 | 1.4600E-06 | 1.0000 | Pyrophosphate | succinic acid | 0.3214 | 2.2200E-16 | 5.3200E-14 | 1.0000 |
| OAHFA(18:1/18:0) | SQDG(16:0/18:2) | 0.0957 | 2.2800E-05 | 1.1839E-03 | 0.9933 | Glucaric acid | succinic acid | 0.1053 | 3.1300E-06 | 2.1281E-04 | 0.9983 |
| OAHFA(18:1/18:0) | FA(18:1) | 0.0946 | 2.8200E-05 | 1.4074E-03 | 0.9933 | 2-Hydroxybutanoic acid | sucrose | -0.0925 | 4.2900E-05 | 1.9944E-03 | 0.9865 |
| PC(16:0/18:1) | PC(18:0/18:1) | 0.2781 | 2.2200E-16 | 5.3200E-14 | 1.0000 | beta-Alanine | sucrose | -0.1130 | 5.5500E-07 | 4.5800E-05 | 0.9995 |
| PC(16:0/18:1) | PC(18:1/18:1) | 0.1144 | 4.0100E-07 | 3.4300E-05 | 0.9997 | phenylacetaldehyde | sucrose | 0.1631 | 4.2100E-13 | 8.1200E-11 | 1.0000 |
| PC(16:0/18:1) | PC(18:1/18:2) | 0.2115 | 2.2200E-16 | 5.3200E-14 | 1.0000 | Phosphate | sucrose | -0.1137 | 4.7100E-07 | 3.9600E-05 | 0.9997 |
| PC(16:0/18:1) | PE(16:0/18:1) | 0.1848 | 2.2200E-16 | 5.3200E-14 | 1.0000 | proline | sucrose | -0.1105 | 9.8400E-07 | 7.6600E-05 | 0.9994 |
| PC(16:0/18:1) | PE(18:0/18:1) | -0.1045 | 3.7100E-06 | 2.4709E-04 | 0.9983 | 2-Aminophenol | sulfuric acid | 0.1183 | 1.5700E-07 | 1.4600E-05 | 0.9998 |
| PC(16:0/18:1) | PE(22:0/18:3) | 0.0913 | 5.3700E-05 | 2.4065E-03 | 0.9865 | Acetol | sulfuric acid | 0.1127 | 6.0100E-07 | 4.9300E-05 | 0.9994 |
| PC(16:0/18:1) | PI(16:0/18:1) | -0.1071 | 2.1200E-06 | 1.5052E-04 | 0.9984 | glutamine | sulfuric acid | -0.0987 | 1.2500E-05 | 7.1203E-04 | 0.9953 |
| PC(16:0/18:1) | PI(16:0/18:2) | -0.2118 | 2.2200E-16 | 5.3200E-14 | 1.0000 | sorbose | sulfuric acid | 0.0988 | 1.2300E-05 | 7.0003E-04 | 0.9953 |
| PC(16:0/18:2) | PC(16:0/18:3) | 0.3968 | 2.2200E-16 | 5.3200E-14 | 1.0000 | 3-Methylcatechol | D-Tagatose | -0.0965 | 1.9400E-05 | 1.0352E-03 | 0.9933 |
| PC(16:0/18:2) | PC(18:0/18:1) | -0.1040 | 4.1100E-06 | 2.6914E-04 | 0.9983 | 5-aminovaleric acid lactam | D-Tagatose | 0.1075 | 1.9500E-06 | 1.3990E-04 | 0.9990 |
| PC(16:0/18:2) | PC(18:2/18:2) | 0.4924 | 2.2200E-16 | 5.3200E-14 | 1.0000 | D-Glyceric acid | D-Tagatose | -0.0949 | 2.6700E-05 | 1.3478E-03 | 0.9933 |
| PC(16:0/18:2) | PE(16:0/18:2) | 0.1198 | 1.1100E-07 | 1.0600E-05 | 0.9999 | picolinic acid | D-Tagatose | 0.0928 | 4.0000E-05 | 1.8841E-03 | 0.9895 |
| PC(16:0/18:2) | PE(18:1/18:2) | -0.1079 | 1.7700E-06 | 1.2905E-04 | 0.9990 | Atropine | tartaric acid | 0.0908 | 5.8400E-05 | 2.5795E-03 | 0.9865 |
| PC(16:0/18:2) | PE(18:3/18:3) | -0.1316 | 5.3600E-09 | 6.4000E-07 | 1.0000 | phosphomycin | tartaric acid | 0.1064 | 2.4500E-06 | 1.7182E-04 | 0.9984 |
| PC(16:0/18:2) | PI(16:0/18:2) | -0.1674 | 9.8400E-14 | 1.9900E-11 | 1.0000 | sorbose | tartaric acid | 0.4570 | 2.2200E-16 | 5.3200E-14 | 1.0000 |
| PC(16:0/18:3) | PC(18:2/18:2) | 0.1668 | 1.2100E-13 | 2.4200E-11 | 1.0000 | 3-Methyl-2-oxobutanoic acid | taurine | 0.0971 | 1.7200E-05 | 9.3710E-04 | 0.9933 |
| PC(16:0/18:3) | PE(16:0/18:2) | -0.0919 | 4.7900E-05 | 2.1889E-03 | 0.9865 | 4-Aminobutyric acid(GABA) | taurine | 0.1224 | 5.8200E-08 | 5.8300E-06 | 0.9999 |
| PC(16:0/18:3) | PE(16:0/18:3) | 0.2309 | 2.2200E-16 | 5.3200E-14 | 1.0000 | alanine | taurine | 0.0920 | 4.7000E-05 | 2.1556E-03 | 0.9865 |
| PC(16:0/18:3) | PE(18:2/18:2) | -0.1697 | 4.4000E-14 | 9.1500E-12 | 1.0000 | Itaconic acid | taurine | 0.1171 | 2.1400E-07 | 1.9400E-05 | 0.9998 |
| PC(16:0/18:3) | PE(18:3/18:3) | 0.2033 | 2.2200E-16 | 5.3200E-14 | 1.0000 | Menthone | taurine | 0.0950 | 2.6000E-05 | 1.3196E-03 | 0.9933 |
| PC(16:0/18:3) | FA(18:3) | -0.0929 | 3.9800E-05 | 1.8746E-03 | 0.9895 | Inositol | taurine | -0.0957 | 2.2700E-05 | 1.1823E-03 | 0.9933 |
| PC(18:0/18:1) | PC(18:1/18:1) | 0.3544 | 2.2200E-16 | 5.3200E-14 | 1.0000 | N-epsilon-Acetyl-L-lysine | taurine | -0.0951 | 2.5900E-05 | 1.3140E-03 | 0.9933 |
| PC(18:0/18:1) | PC(18:2/18:2) | -0.1406 | 4.4700E-10 | 6.0500E-08 | 1.0000 | nicotinamide | taurine | 0.1026 | 5.5700E-06 | 3.5256E-04 | 0.9967 |
| PC(18:0/18:1) | PE(18:0/18:1) | 0.2760 | 2.2200E-16 | 5.3200E-14 | 1.0000 | oxalacetic acid | taurine | -0.0941 | 3.1500E-05 | 1.5408E-03 | 0.9895 |
| PC(18:0/18:1) | PE(18:1/18:1) | -0.1056 | 2.9200E-06 | 2.0072E-04 | 0.9983 | putrescine | taurine | -0.0966 | 1.9000E-05 | 1.0171E-03 | 0.9933 |
| PC(18:0/18:1) | PE(18:1/18:2) | -0.0907 | 5.9700E-05 | 2.6235E-03 | 0.9833 | squalene | taurine | 0.1463 | 8.3400E-11 | 1.2600E-08 | 1.0000 |
| PC(18:0/18:1) | PI(16:0/18:1) | -0.1193 | 1.2500E-07 | 1.1800E-05 | 0.9999 | succinic acid | taurine | -0.0927 | 4.1000E-05 | 1.9210E-03 | 0.9895 |
| PC(18:0/18:1) | PI(16:0/18:2) | -0.1754 | 5.7700E-15 | 1.2900E-12 | 1.0000 | Atropine | Threonate | -0.1307 | 6.7400E-09 | 7.9200E-07 | 1.0000 |
| PC(18:1/18:1) | PC(18:1/18:2) | 0.1069 | 2.2100E-06 | 1.5618E-04 | 0.9984 | benzoic acid | Threonate | 0.1049 | 3.3900E-06 | 2.2865E-04 | 0.9983 |
| PC(18:1/18:1) | PE(16:0/18:1) | -0.1358 | 1.7000E-09 | 2.1600E-07 | 1.0000 | fucose | Threonate | 0.1449 | 1.2700E-10 | 1.8600E-08 | 1.0000 |
| PC(18:1/18:1) | PE(18:1/18:1) | 0.1866 | 2.2200E-16 | 5.3200E-14 | 1.0000 | glutamine | Threonate | 0.0999 | 9.7500E-06 | 5.7469E-04 | 0.9961 |
| PC(18:1/18:1) | PI(16:0/18:1) | -0.1228 | 5.3100E-08 | 5.3600E-06 | 0.9999 | glutathione | Threonate | 0.0924 | 4.3400E-05 | 2.0144E-03 | 0.9865 |
| PC(18:1/18:1) | PI(16:0/18:2) | -0.2729 | 2.2200E-16 | 5.3200E-14 | 1.0000 | linolenic acid | Threonate | 0.1570 | 3.0700E-12 | 5.4900E-10 | 1.0000 |
| PC(18:1/18:1) | PI(18:1/18:1) | -0.1131 | 5.4600E-07 | 4.5100E-05 | 0.9996 | oxalic acid | Threonate | 0.1087 | 1.4800E-06 | 1.1018E-04 | 0.9991 |
| PC(18:1/18:2) | PC(18:2/18:2) | 0.1355 | 1.8700E-09 | 2.3600E-07 | 1.0000 | oxoproline | Threonate | -0.1040 | 4.1200E-06 | 2.6965E-04 | 0.9983 |
| PC(18:1/18:2) | PE(16:0/18:3) | -0.1191 | 1.3100E-07 | 1.2400E-05 | 0.9999 | sarcosine | Threonate | 0.1194 | 1.2300E-07 | 1.1700E-05 | 0.9999 |
| PC(18:1/18:2) | PE(18:0/18:1) | -0.1402 | 4.9100E-10 | 6.6300E-08 | 1.0000 | sorbose | Threonate | 0.1060 | 2.6700E-06 | 1.8545E-04 | 0.9984 |
| PC(18:1/18:2) | PE(18:1/18:1) | -0.1284 | 1.2500E-08 | 1.4000E-06 | 1.0000 | succinic acid | Threonate | -0.1046 | 3.6200E-06 | 2.4176E-04 | 0.9983 |
| PC(18:1/18:2) | PE(18:1/18:2) | 0.1763 | 4.2200E-15 | 9.5500E-13 | 1.0000 | sulfuric acid | Threonate | 0.1052 | 3.2000E-06 | 2.1732E-04 | 0.9983 |
| PC(18:1/18:2) | PI(16:0/18:1) | -0.1349 | 2.1800E-09 | 2.7300E-07 | 1.0000 | tartaric acid | Threonate | 0.2678 | 2.2200E-16 | 5.3200E-14 | 1.0000 |
| PC(18:1/18:2) | PI(16:0/18:2) | -0.2021 | 2.2200E-16 | 5.3200E-14 | 1.0000 | 3-Methylcatechol | threonine | 0.1035 | 4.6300E-06 | 2.9910E-04 | 0.9975 |
| PC(18:2/18:2) | PE(16:0/18:1) | -0.1082 | 1.6400E-06 | 1.2046E-04 | 0.9990 | Aconitic Acid | threonine | 0.1169 | 2.2000E-07 | 1.9900E-05 | 0.9998 |
| PC(18:2/18:2) | PE(18:2/18:2) | 0.2327 | 2.2200E-16 | 5.3200E-14 | 1.0000 | L-Malic acid | threonine | 0.1342 | 2.6400E-09 | 3.2700E-07 | 1.0000 |
| PC(18:2/18:2) | PE(18:3/18:2) | 0.1010 | 7.8200E-06 | 4.7492E-04 | 0.9961 | 3-Hydroxypropionic acid | Thymidine | 0.1083 | 1.6200E-06 | 1.1892E-04 | 0.9990 |
| PC(18:2/18:2) | PI(16:0/18:2) | -0.1206 | 8.9900E-08 | 8.7300E-06 | 0.9999 | 3-Methylcatechol | Thymidine | 0.0944 | 2.9600E-05 | 1.4613E-03 | 0.9933 |
| PE(16:0/18:1) | PE(16:0/18:2) | 0.1331 | 3.5500E-09 | 4.3300E-07 | 1.0000 | Itaconic acid | Thymidine | -0.0967 | 1.8700E-05 | 1.0045E-03 | 0.9933 |
| PE(16:0/18:1) | PE(16:0/18:3) | 0.1275 | 1.5700E-08 | 1.7300E-06 | 1.0000 | Inositol | Thymidine | -0.1066 | 2.3700E-06 | 1.6630E-04 | 0.9984 |
| PE(16:0/18:1) | PE(18:0/18:1) | 0.3167 | 2.2200E-16 | 5.3200E-14 | 1.0000 | Threonate | Thymidine | 0.1036 | 4.5000E-06 | 2.9145E-04 | 0.9975 |
| PE(16:0/18:1) | PE(18:1/18:2) | 0.1428 | 2.3100E-10 | 3.2800E-08 | 1.0000 | 3-Hydroxyphenylacetic acid | uracil | 0.0948 | 2.7100E-05 | 1.3641E-03 | 0.9933 |
| PE(16:0/18:1) | PE(20:0/18:1) | 0.0910 | 5.7000E-05 | 2.5259E-03 | 0.9865 | 4-Hydroxyphenylethanol | uracil | 0.0970 | 1.7600E-05 | 9.5474E-04 | 0.9933 |
| PE(16:0/18:1) | PG(16:0/18:1) | 0.1424 | 2.6400E-10 | 3.7200E-08 | 1.0000 | 9-Fluorenone | uracil | 0.1427 | 2.4300E-10 | 3.4400E-08 | 1.0000 |
| PE(16:0/18:1) | PG(18:0/18:2) | -0.1116 | 7.7100E-07 | 6.1800E-05 | 0.9994 | Acetol | uracil | 0.1164 | 2.5100E-07 | 2.2400E-05 | 0.9998 |
| PE(16:0/18:1) | PI(16:0/18:2) | -0.1778 | 2.4400E-15 | 5.6300E-13 | 1.0000 | Creatine | uracil | 0.1415 | 3.4400E-10 | 4.7600E-08 | 1.0000 |
| PE(16:0/18:1) | PI(18:0/18:1) | -0.0926 | 4.1700E-05 | 1.9504E-03 | 0.9895 | D-Glucose | uracil | -0.0926 | 4.1700E-05 | 1.9504E-03 | 0.9895 |
| PE(16:0/18:1) | PI(18:1/18:1) | -0.0915 | 5.1200E-05 | 2.3133E-03 | 0.9865 | isocitric acid | uracil | -0.0961 | 2.1300E-05 | 1.1213E-03 | 0.9933 |
| PE(16:0/18:2) | PE(16:0/18:3) | 0.1186 | 1.4700E-07 | 1.3700E-05 | 0.9999 | lauric acid | uracil | -0.0928 | 4.0500E-05 | 1.9026E-03 | 0.9895 |
| PE(16:0/18:2) | PE(18:1/18:2) | -0.1086 | 1.5200E-06 | 1.1237E-04 | 0.9991 | mannose | uracil | 0.2333 | 2.2200E-16 | 5.3200E-14 | 1.0000 |
| PE(16:0/18:2) | PE(18:2/18:2) | 0.2450 | 2.2200E-16 | 5.3200E-14 | 1.0000 | phenylacetaldehyde | uracil | 0.1020 | 6.3300E-06 | 3.9478E-04 | 0.9967 |
| PE(16:0/18:2) | PE(18:2/23:0) | 0.1021 | 6.1400E-06 | 3.8425E-04 | 0.9967 | squalene | uracil | 0.0933 | 3.6400E-05 | 1.7414E-03 | 0.9895 |
| PE(16:0/18:2) | PE(18:3/18:2) | 0.1086 | 1.5200E-06 | 1.1265E-04 | 0.9991 | Threonate | uracil | 0.1218 | 6.8000E-08 | 6.7200E-06 | 0.9999 |
| PE(16:0/18:2) | PE(22:0/18:2) | 0.1866 | 2.2200E-16 | 5.3200E-14 | 1.0000 | Geraniol | vanillin | -0.0960 | 2.1700E-05 | 1.1377E-03 | 0.9933 |
| PE(16:0/18:2) | PI(16:0/18:2) | -0.1650 | 2.1900E-13 | 4.3200E-11 | 1.0000 | leucine | vanillin | 0.0969 | 1.8100E-05 | 9.7611E-04 | 0.9933 |
| PE(16:0/18:3) | PE(18:3/18:2) | 0.1121 | 6.7900E-07 | 5.5100E-05 | 0.9994 | N-Carbamyl-L-glutamate | vanillin | 0.1739 | 1.0200E-14 | 2.2200E-12 | 1.0000 |
| PE(16:0/18:3) | PE(18:3/18:3) | 0.2984 | 2.2200E-16 | 5.3200E-14 | 1.0000 | oxoproline | vanillin | -0.0965 | 1.9700E-05 | 1.0489E-03 | 0.9933 |
| PE(16:0/18:3) | PE(20:0/18:3) | 0.2038 | 2.2200E-16 | 5.3200E-14 | 1.0000 | Glucaric acid | vanillin | 0.1050 | 0.0000 | 0.0002 | 0.9983 |
| PE(18:0/18:1) | PE(18:1/18:1) | 0.2870451 | 2.22E-16 | 5.32E-14 | 1.0000 |  |  |  |  |  |  |

CC: Conditional pairwise Pearson correlation coefficient. PP: Posterior Probabilities (=1 - local fdr).

**Table S8.** 175 QTLs for seed oil-related traits, their candidate genes, and miRNAs identified using multiple methods or across multiple environments

| **Locus** | **Trait** | **Quantitative trait locus mapping** | | | | | |  | **Comparative genomics** | | | | | **Candidate miRNA** |
| --- | --- | --- | --- | --- | --- | --- | --- | --- | --- | --- | --- | --- | --- | --- |
|  |  | **Chr** | **Markers associated** | **Effect** | **LOD score** | **r^2^（%）** | **Method^†^** |  | **Gene ID** | **Gene name** | **Arabidopsis homologs** | **Protein Family Name** | **Reference** |  |
| q1-1 | Palmitic acids | 1 | Marker1819518, Marker1819518 | -0.13~-0.13 | 4.65~5.14 | 2.41~2.44 | 3, 6, NJ2015 |  |  |  |  |  |  | miR4406 |
| q1-2 | Linoleic acid | 1 | Marker1912349, Marker1919734 | -1.58~-0.47 | 3.14~15.8 | 2.17~16.2 | 1(ff), 2(ff), 6, 3, 1(all), NJ2015; 2(ff), 2(zf), BLUP |  |  |  |  |  |  |  |
| q1-3 | Oleic acid | 1 | Marker1937544, Marker1919734 | 0.4~0.64 | 3~4.07 | 2.4~3.05 | 2(all), NJ2015 |  |  |  |  |  |  |  |
| q1-4 | Linolenic acid | 1 | Marker1922682, Marker1827676 | -0.18~0 | 2.95~9.8 | 0~6.14 | 6, 1(all), 3, BLUP; 2(ff), WH2014; 1(all), NJ2015; 1(all), 6, EZ2015 |  |  |  |  |  |  | miR156t |
| q2-1 | Stearic acid | 2 | Marker1187241, Marker1187241 | -0.1~-0.09 | 2.99~3.3 | 4.38~5.53 | 1(ff), 2(ff), EZ2015 |  |  |  |  |  |  | miR408a |
| q2-2 | Linoleic acid | 2 | Marker1267284, Marker1208419 | -0.66~-0.25 | 3.64~13.5 | 1.52~3.39 | 6, BLUP; 2(zf), WH2014 |  | *Glyma02g08350* | *GmHAD* | *At5g60340* | Hydroxyacyl-ACP Dehydrase | Proof of function of a putative 3-hydroxyacyl-acyl carrier protein dehydratase from higher plants by mass spectrometry of product formation |  |
| q2-3 | Palmitic acids | 2 | Marker1282622, Marker1229770 | -0.14~-0.14 | 3.7~4.18 | 5.54~7.88 | 1(zf), 2(zf), WH2014 |  |  |  |  |  |  | miR9735, miR1512c, miR1512b |
| q2-4 | Palmitic acids | 2 | Marker1286890, Marker1286890 | -0.19~-0.09 | 5.42~8.75 | 3.16~6.41 | 5, 8, 7, EZ2015 |  |  |  |  |  |  | miR4348c, miR398b |
| q2-5 | Palmitic acids | 2 | Marker1223089, Marker1284445 | -0.17~-0.08 | 2.54~9.82 | 3.23~9.49 | 1(all), 5, BLUP; 2(zf), 2(ff), 2(all), 1(ff), 1(all), 3, 4, 6, EZ2015; 2(all), NJ2015 |  |  |  |  |  |  |  |
| q2-6 | Linoleic acid | 2 | Marker1225689, Marker1183683 | -0.7~-0.35 | 3.06~9.23 | 1.58~4.94 | 6, 3, 2(all), WH2014 |  |  |  |  |  |  | miR1523b, miR1523a |
| q2-7 | Palmitic acids | 2 | Marker1193624, Marker1221985 | -0.12~-0.08 | 3.67~8.95 | 3.37~5.54 | 2(all), EZ2015; 2(all), 1(all), 3, 4, WH2014 |  | *Glyma02g15600* | *GmFAB2* | *At2g43710* | Stearoyl-ACP Desaturase | A mutant of *Arabidopsis* with increased levels of stearic acid | miR10412 |
| q2-8 | Palmitic acids | 2 | Marker1161043, Marker1192262 | -0.07~-0.12 | 5.59~8.85 | 2.36~4.97 | 2(ff), WH2014; 3, BLUP |  |  |  |  |  |  |  |
| q2-9 | Oil content | 2 | Marker1289976, Marker1171430 | -0.35~-0.09 | 3.54~7.34 | 1.94~6.2 | 2(all), BLUP; 3, 6, 2(all), EZ2015 |  |  |  |  |  |  | miR166i, miR5677, miR167b, miR9751 |
| q2-10 | Linolenic acid | 2 | Marker1157322, Marker1284396 | 0.02~0.09 | 2.66~3.29 | 0.08~3.17 | 6, 2(ff), BLUP |  |  |  |  |  |  | miR319h |
| q3-1 | Linolenic acid | 3 | Marker2417930, Marker2506576 | -0.26~0.53 | 3.2~30.17 | 1.79~17.5 | 4, 8, 1(all), 2(ff), 3, 1(ff), BLUP; 4, 8, 7, 3, 1(all), 2(ff), 6, 1(ff), 2(all), NJ2015; 1(all), 2(ff), 4, 8, 6, 7, 3, EZ2015 |  |  |  |  |  |  | miR9746a, miR9746b, miR9746c, miR9746d, miR9746e, miR9746i, miR9746f, miR9746g |
| q3-2 | Palmitic acids | 3 | Marker2408504, Marker2468882 | -0.19~-0.13 | 3.44~16.78 | 3.17~11.46 | 2(ff), WH2014; 2(zf), EZ2015; 3, 4, 7, 6, NJ2015 |  | *Glyma03g04960* | *GmLTP* | *At2g38540* | Lipid Transfer Protein | Lipid transfer protein 3 as a target of *MYB96* mediates freezing and drought stress in *Arabidopsis* |  |
| q3-3 | Linolenic acid | 3 | Marker2408504, Marker2481759 | -0.13~-0.12 | 2.76~3.76 | 4.87~5.8 | 2(zf), WH2014; 2(zf), BLUP |  |  |  |  |  |  |  |
| q3-4 | Palmitic acids | 3 | Marker2451716, Marker2451716 | -0.11~-0.12 | 5.21~11.17 | 7.18~7.28 | 3, NJ2015; 2(ff), WH2014 |  |  |  |  |  |  |  |
| q3-5 | Oleic acid | 3 | Marker2413649, Marker2413649 | 0.35~0.66 | 2.74~4.03 | 0.69~2.1 | 1(all), NJ2015 |  |  |  |  |  |  |  |
| q3-6 | Palmitic acids | 3 | Marker2473759, Marker2409432 | -0.13~-0.11 | 3.71~3.86 | 6.87~8.91 | 2(zf), BLUP; 2(zf), EZ2015 |  | *Glyma03g27360* | *GmDCI1* | *At5g43280* | Dienoyl CoA Isomerase | Molecular identification and characterization of the *Arabidopsis* Delta(3,5),Delta 2,4)-dienoyl-coenzyme A isomerase, a peroxisomal enzyme participating in the beta-oxidation cycle of unsaturated fatty acids |  |
| q3-7 | Palmitic acids | 3 | Marker2483347, Marker2483347 | 0.09~0.2 | 4.11~11.63 | 2.87~11.37 | 1(all), 2(all), WH2014 |  |  |  |  |  |  |  |
| q3-8 | Palmitic acids | 3 | Marker2425803, Marker2425803 | 0.08~0.12 | 3.28~7.64 | 2.69~4.68 | 3, 4, 6, 2(ff), WH2014; 1(all), BLUP |  | *Glyma03g40250* | *GmACP3* | *At1g54630* | Acyl Carrier Protein | Differential regulation of mRNA levels of acyl carrier protein isoforms in Arabidopsis | miR164f |
| q3-9 | Palmitic acids | 3 | Marker2510599, Marker2405141 | 0.11~0.26 | 4.73~5.18 | 6.3~9.1 | 1(ff), 2(ff), NJ2015; 1(ff), BLUP |  | *Glyma03g42190* | *Gmα-PDHC* | *At1g01090* | Pyruvate Dehydrogenase alpha subunit, E1a component of Pyruvate Dehydrogenase Complex | Pyruvate-Dehydrogenase (Pdh) deficiency caused by a 21-base pair insertion mutation in the E1-Alpha subunit | miR5773, miR164g, miR1516a |
| q3-10 | Palmitic acids | 3 | Marker2405141, Marker2413638 | 0.07~0.14 | 3.12~7.15 | 1.83~6.86 | 6, NJ2015; 5, 8, 4, 1(all), 3, 6, EZ2015; 2(ff), BLUP |  | *Glyma03g42140* | *GmKCS1* | *At1g01120* | Ketoacyl-CoA Synthase | *KCS1* encodes a fatty acid elongase 3-ketoacyl-CoA synthase affecting wax biosynthesis in Arabidopsis thaliana |  |
| q3-11 | Palmitic acids | 3 | Marker2503824, Marker2390156 | 0.08~0.16 | 3.97~10.3 | 2.47~3.48 | 3, 2(all), NJ2015; 2(all), BLUP |  |  |  |  |  |  |  |
| q4-1 | Linoleic acid | 4 | Marker2252022, Marker2233523 | -0.53~-0.36 | 3.47~3.92 | 1.13~2.63 | 2(all), NJ2015 |  | *Glyma04g09040* | *GmMAGL* | *At5g11650* | Monoacylglycerol Lipase (MAGL) |  |  |
| q4-2 | Linoleic acid | 4 | Marker2230222, Marker2230222 | 0.25~0.52 | 2.75~5.9 | 1.26~1.9 | 1(all), EZ2015; 6, BLUP |  | *Glyma04g37420* | *GmFatB* | *At1g08510* | Acyl-ACP Thioesterase B | Disruption of the *FATB* gene in Arabidopsis demonstrates an essential role of saturated fatty acids in plant growth |  |
| q5-1 | Linolenic acid | 5 | Marker2153198, Marker2153198 | 0.1~0.14 | 2.71~5.8 | 1.73~5.69 | 2(all), BLUP; 2(all), EZ2015 |  | *Glyma05g00570* | *GmDAGK* | *At5g07920* | Diacylglycerol Kinase |  |  |
| q5-2 | Oil content | 5 | Marker2104405, Marker2092235 | -0.25~-0.10 | 2.81~6.38 | 0.41~4.35 | 1(all), 2(all), BLUP; 2, WH2014; EZ2015, 3; 1, EZ2015; EZ2015, 6 |  | *Glyma05g04940* | *GmACX1* | *At4g16760* | Acyl-CoA Oxidase | Sucrose rescues seedling establishment but not germination of *Arabidopsis* mutants disrupted in peroxisomal fatty acid catabolism |  |
| q5-3 | Oil content | 5 | Marker2191229, Marker2192021 | -0.65~-0.09 | 3.81~22.74 | 1.55~15.69 | 3, 4, 5, 8, 1(ff), 2(ff), BLUP; 1(ff)(all), 2(ff)(all), 3, 4, 5, 6, 8, WH2014 |  | *Glyma05g04330* | *GmLPPβ* | *At4g22550* | Phosphatidate Phosphatase | Plastidic phosphatidic acid phosphatases identified in a distinct subfamily of lipid phosphate phosphatases with prokaryotic origin |  |
| q5-4 | Linolenic acid | 5 | Marker2100153, Marker2204980 | 0.07~0.16 | 3.45~5 | 1.66~7.47 | 2(ff), EZ2015; 1(all), 6, 3, BLUP; 1(all), 2(zf), WH2014 |  | *Glyma05g08060* | *GmFatB* | *At1g08510* | Acyl-ACP Thioesterase B | Disruption of the *FATB* gene in Arabidopsis demonstrates an essential role of saturated fatty acids in plant growth | miR1509b, miR162b |
| q5-5 | Linoleic acid | 5 | Marker2120443, Marker2120443 | 0.4~0.43 | 3.89~4.26 | 1.6~1.85 | 6, 3, NJ2015 |  |  |  |  |  |  |  |
| q5-6 | Linoleic acid | 5 | Marker2205232, Marker2110241 | 0.52~0.81 | 2.58~10.92 | 2.11~8.13 | 2(zf), BLUP; 1(all), 2(zf), NJ2015 |  | *Glyma05g08880* | *GmOBO* | *At3g01570* | Oil-Body Oleosin | A novel role for oleosins in freezing tolerance of oilseeds in Arabidopsis thaliana | miR5374 |
| q5-7 | Oleic acid | 5 | Marker2095085, Marker2200075 | -0.9~-0.42 | 2.72~5.42 | 1.03~5.41 | 1(all), 2(all), WH2014 |  | *Glyma05g09400* | *GmLCBK* | *At5g23450* | Long Chain Base Kinase | Phosphorylation of sphingoid long-chain bases in *Arabidopsis*: Functional characterization and expression of the first sphingoid long-chain base kinase gene in plants |  |
| q5-8 | Linolenic acid | 5 | Marker2095085, Marker2095085 | 0.11~0.19 | 3.65~4.47 | 2.47~3.43 | 4, 5, WH2014 |  |  |  |  |  |  |  |
| q5-9 | Stearic acid | 5 | Marker2195584, Marker2195226 | -0.1~-0.09 | 3.07~3.7 | 4.48~5.69 | 2(ff), 1(ff), EZ2015 |  |  |  |  |  |  |  |
| q5-10 | Linoleic acid | 5 | Marker2104599, Marker2138708 | 0~1.04 | 2.98~11.16 | 0~10.94 | 3, 6, 1(all), 1(ff), NJ2015; 6, 1(all), 1(ff), 3, 2(all), BLUP; 1(all), 1(zf), 4, 8, 5, 3, 2(all), 6, WH2014 |  |  |  |  |  |  | miR156i |
| q5-11 | Oil content | 5 | Marker2188641, Marker2103167 | 0.12~0.52 | 3~10.4 | 1.51~7.81 | 1(all), 1(ff), 3, 4, 5, 6, 8, WH2014; 1(all), 1(ff), 3, 4, 8, BLUP; 1(all), 1(ff), 2(ff), 3, EZ2015 |  | *Glyma05g33790* | *GmPEAMT* | *At3g18000* | Phosphoethanolamine N-Methyltransferase | Translational regulation of *Arabidopsis* *XIPOTL1* is modulated by phosphocholine levels via the phylogenetically conserved upstream open reading frame 30 |  |
| q5-12 | Linolenic acid | 5 | Marker2100085, Marker2100085 | -0.16~-0.11 | 2.66~3.55 | 1.69~4.07 | 1(all), 1(ff), EZ2015 |  |  |  |  |  |  |  |
| q5-13 | Stearic acid | 5 | Marker2091868, Marker2138708 | 0~0.11 | 3.09~6.32 | 0.01~7.21 | 6, 3, 1(zf), EZ2015 |  |  |  |  |  |  |  |
| q6-1 | Palmitic acids | 6 | Marker1960320, Marker2015703 | 0.12~0.13 | 2.86~3.44 | 4.39~5.53 | 2(zf), EZ2015; 1(zf), WH2014 |  | *Glyma06g04230* | *GmPAH2* | *At5g42870* | Phosphatidate Phosphatase | PHOSPHATIDIC ACID PHOSPHOHYDROLASE1 and 2 regulate phospholipid synthesis at the endoplasmic reticulum in *Arabidopsis* |  |
| q6-2 | Palmitic acids | 6 | Marker1964032, Marker2056330 | 0.05~0.19 | 3.23~11.23 | 3.07~10.66 | 2(all), 2(zf), 1(all), 2(ff), 7, WH2014; 7, BLUP; 1(all), 5, 7, EZ2015 |  | *Glyma06g07230* | *GmPLDα6* | *At3g15730* | Phospholipase D &alpha; | PLDα1-knockdown soybean seeds display higher unsaturated glycerolipid contents and seed vigor in high temperature and humidity environments. |  |
| q6-3 | Linolenic acid | 6 | Marker2029409, Marker1949300 | 0.09~0.16 | 3.28~6.51 | 2.07~4.24 | 1(all), 1(ff)+C46:I48, 3, 4, 5+, 6, 8, WH2014; 1(all), 1(ff), 3, 4, 8, BLUP; 2, 1, 1, 3, EZ2015 |  | *Glyma06g08290* | *GmOBO* | *At2g25890* | Oil-Body Oleosin | A novel group of oleosins is present inside the pollen of *Arabidopsis* |  |
| q6-4 | Palmitic acids | 6 | Marker1970961, Marker2044697 | 0.08~0.14 | 2.53~9.41 | 3.32~9.22 | 1(ff), WH2014; 1(ff), BLUP; 2(all), NJ2015 |  | *Glyma06g07360* | *GmLPP* | *At5g20060* | Lysophospholipase |  |  |
| q6-5 | Linolenic acid | 6 | Marker2074932, Marker2014747 | 0.09~0.2 | 3.69~6.79 | 2.08~10.12 | 1(zf), 2(zf), 2(all), 4, 8, WH2014; 2(zf), BLUP; 2(zf), EZ2015 |  |  |  |  |  |  |  |
| q6-6 | Palmitic acids | 6 | Marker2044697, Marker2011902 | 0.08~0.15 | 3.36~13.08 | 2.73~9.02 | 3, EZ2015; 3, 4, 6, NJ2015; 3, 4, WH2014 |  |  |  |  |  |  |  |
| q6-7 | Oil content | 6 | Marker2031606, Marker2077769 | 0~0.27 | 3.33~4.86 | 0~7.46 | 1, WH2014; WH2014, BLUP, 6; BLUP, WH2014, 3 |  |  |  |  |  |  |  |
| q6-8 | Linolenic acid | 6 | Marker2000797, Marker2037589 | -0.26~-0.08 | 2.81~7.18 | 1.48~12.24 | 2(zf), 1(all), WH2014; 4, 8, 7, 2(all), NJ2015; 2(zf), 1(zf), 2(all), EZ2015; 7, 2(zf), BLUP |  | *Glyma06g11860* | *GmLACS9* | *At1g77590* | Long-Chain Acyl-CoA Synthetase | *Arabidopsis* contains nine long-chain acyl-coenzyme a synthetase genes that participate in fatty acid and glycerolipid metabolism | miR4405, miR171s, miR171h |
| q6-9 | Linolenic acid | 6 | Marker2064591, Marker2064591 | -0.33~-0.18 | 3.78~4.03 | 3.09~3.7 | 1(all), 5, NJ2015 |  | *Glyma06g15820* | *GmPI4Kβ* | *At5g64070* | Phosphatidylinositol-4-Kinase &beta | *Arabidopsis* type-III phosphatidylinositol 4-kinases beta 1 and beta 2 are upstream of the phospholipase c pathway triggered by cold exposure | miR6299 |
| q7-1 | Oleic acid | 7 | Marker364973, Marker352632 | -0.79~-0.46 | 3.43~10.08 | 2.24~5.67 | 2(all), 1(all), 2(ff), BLUP; 2(all), EZ2015 |  | *Glyma07g03350* | *GmPDCT/ROD1* | *At3g15820* | Phosphatidylcholine:diacylglycerol cholinephosphotransferase | An enzyme regulating triacylglycerol composition is encoded by the *ROD1* gene of *Arabidopsis* |  |
| q7-2 | Linoleic acid | 7 | Marker364973, Marker401588 | 0.4~0.68 | 4.23~6.61 | 1.45~3.72 | 2(all), EZ2015 |  |  |  |  |  |  |  |
| q7-3 | Linoleic acid | 7 | Marker401588, Marker399060 | 0.25~0.54 | 2.55~4.09 | 0.86~2.62 | 1(all), 8, 6, 3, BLUP; 1(all), 6, EZ2015; 6, NJ2015; 6, WH2014 |  |  |  |  |  |  |  |
| q7-4 | Palmitic acids | 7 | Marker402894, Marker352760 | -0.09~-0.05 | 3.1~7.69 | 0.55~3.19 | 2(all), 3, 6, 6, NJ2015; 8, 3, 4, EZ2015 |  | *Glyma07g07560* | *GmFAH* | *At2g45970* | Fatty Acyl omega-Hydroxylase | Functional analysis of the LACERATA gene of *Arabidopsis* provides evidence for different robes of fatty acid omega-hydroxylation in development | miR10423 |
| q7-5 | Stearic acid | 7 | Marker367654, Marker367654 | -0.09~-0.07 | 3.72~5.06 | 3.29~4.92 | 4, 8, EZ2015 |  | *Glyma07g07580* | *GmGPAT8* | *At4g00400* | Glycerol-3-Phosphate Acyltransferase | Identification of acyltransferases required for cutin biosynthesis and production of cutin with suberin-like monomers |  |
| q7-6 | Linolenic acid | 7 | Marker288299, Marker366921 | -0.12~-0.1 | 3.06~4.81 | 3.07~3.96 | 2(all), WH2014; 2(all), BLUP |  | *Glyma07g08950* | *GmGA20OX* | *At5g51810* | Gibberellin biosynthesis III | The transcriptomic signature of developing soybean seeds reveals the genetic basis of seed trait adaptation during domestication |  |
| q7-7 | Linoleic acid | 7 | Marker366921, Marker297491 | -1.2~-0.35 | 3.48~10.99 | 2.7~8.51 | 6, WH2014; 1(all), 4, 8, 6, 3, BLUP; 2(all), 2(zf), 1(all), 4, 8, 5, 6, 3, NJ2015 |  | *Glyma07g08810* | *GmLPEAT* | *At2g45670* | 1-Acylglycerol-3-Phosphoethanolamine Acyltransferase | Characterization of two *Arabidopsis* thaliana acyltransferases with preference for lysophosphatidylethanolamine |  |
| q7-8 | Oleic acid | 7 | Marker366921, Marker297491 | 0.51~1.12 | 2.58~16.03 | 1.5~8.48 | 2(all), 1(all), WH2014; 2(ff), NJ2024; 1(all), 1(ff), 2(ff), BLUP; 1(all), NJ2015 |  | *Glyma07g09370* | *GmMFP* | *At4g29010* | Multifunctional Protein | A defect in beta-oxidation causes abnormal inflorescence development in *Arabidopsis* |  |
| q7-9 | Linolenic acid | 7 | Marker366921, Marker394349 | -0.25~-0.06 | 3.03~7.84 | 0.91~7.2 | 6, 3, WH2014; 1(zf), 1(all), BLUP; 1(zf), 4, NJ2015 |  |  |  |  |  |  |  |
| q7-10 | Oil content | 7 | Marker366921, Marker297491 | -0.19~-0.08 | 3.02~4.83 | 1.65~2.65 | BLUP, 3; 1(all), BLUP |  |  |  |  |  |  |  |
| q7-11 | Palmitic acids | 7 | Marker357373, Marker297491 | -0.09~-0.08 | 2.67~5.75 | 2.33~3.59 | 3, 2(ff), WH2014; 2(ff), EZ2015 |  |  |  |  |  |  |  |
| q7-12 | Stearic acid | 7 | Marker314074, Marker273037 | -0.06~-0.03 | 4.26~4.65 | 2.86~5.2 | 2(all), 7, WH2014 |  |  |  |  |  |  |  |
| q7-13 | Linolenic acid | 7 | Marker348963, Marker308301 | -0.16~-0.07 | 3.23~4.85 | 1.52~2.93 | 2(all), NJ2015; 7, BLUP |  |  |  |  |  |  | miR156l, miR159e |
| q7-14 | Oil content | 7 | Marker309584, Marker298981 | -1.02~-0.04 | 3.45~10.52 | 0.1~35.97 | WH2014, 7; WH2014, 4; 1, 1, WH2014; WH2014, 8 |  |  |  |  |  |  | miR166s, miR4369, miR10424a |
| q7-15 | Stearic acid | 7 | Marker287752, Marker326184 | -0.05~0 | 3.59~11.48 | 0~5.08 | 6, BLUP; 2(zf), WH2014 |  |  |  |  |  |  |  |
| q8-1 | Oil content | 8 | Marker677743, Marker785216 | -0.24~-0.1 | 2.73~4.71 | 2.99~5.42 | 2(all), BLUP; 2, EZ2015 |  |  |  |  |  |  |  |
| q8-2 | Linoleic acid | 8 | Marker675789, Marker734870 | -0.69~-0.43 | 2.64~4.29 | 1.68~4.07 | 4, 8, 2(all), 6, 3, 2(ff), EZ2015 |  | *Glyma08g16620* | *GmPI4Kβ* | *At5g64070* | Phosphatidylinositol-4-Kinase &beta; | *Arabidopsis* Type-III Phosphatidylinositol 4-Kinases beta 1 and beta 2 are Upstream of the Phospholipase C Pathway Triggered by Cold Exposure |  |
| q8-3 | Oil content | 8 | Marker675789, Marker734870 | -0.32~-0.25 | 3.1~3.93 | 1.47~3.23 | EZ2015, 5; 2, EZ2015 |  |  |  |  |  |  |  |
| q8-4 | Stearic acid | 8 | Marker724653, Marker701184 | -0.12~-0.09 | 4.23~5.23 | 4.59~7.81 | 2(ff), 1(all), 1(ff), 5, EZ2015 |  |  |  |  |  |  |  |
| q8-5 | Linolenic acid | 8 | Marker742943, Marker775795 | -0.31~-0.12 | 2.57~14.85 | 1.33~9.88 | 1(all), 1(zf), 1(ff), 2(all), 2(ff), 4, 8, 3, BLUP; 2(zf), 1(all), 1(zf), 2(all), 6, 3, 4, 8, EZ2015; 2(ff), 1(all), 6, 3, 2(all), NJ2015; 2(all), 2(ff), 1(ff), 2(zf), 1(all), 1(zf), 4, 8, 5, 6, 3, WH2014 |  | *Glyma08g45990* | *GmENR1 (MOD1)* | *At2g05990* | Enoyl-ACP Reductase | Deficiency in fatty acid synthase leads to premature cell death and dramatic alterations in plant morphology | miR5785 |
| q8-6 | Oil content | 8 | Marker792354, Marker776631 | 0.11~0.27 | 3.08~9.11 | 1.12~5.93 | 2, 1, WH2014; WH2014, BLUP, 3; WH2014, 5; 2(all), 2(ff), BLUP; WH2014, 6 |  |  |  |  |  |  |  |
| q8-7 | Oleic acid | 8 | Marker727545, Marker750703 | 0.44~0.7 | 3.1~8.37 | 1.57~2.88 | 1(all), 2(all), BLUP; 1(all), 2(all), EZ2015 |  |  |  |  |  |  |  |
| q8-8 | Stearic acid | 8 | Marker673687, Marker674654 | -0.1~-0.09 | 2.58~3.93 | 4.52~6.78 | 1(zf), 2(zf), EZ2015 |  | *Glyma08g46360* | *GmFatA* | *At3g25110* | Acyl-ACP Thioesterase A | Reduced expression of *FatA* thioesterases in *Arabidopsis* affects the oil content and fatty acid composition of the seeds |  |
|  |  |  |  |  |  |  |  |  | *Glyma08g47240* | *GmABI3b* | *At3g24650* | Homologous to the maize transcription factor Viviparous-1 | Deciphering and modifying LAFL transcriptional regulatory network in seed for improving yield and quality of storage compounds. |  |
| q9-1 | Linolenic acid | 9 | Marker468386, Marker499635 | -0.4~-0.01 | 3.07~11.93 | 0.02~12.43 | 1(all), 4, 8, 5, 6, 3, EZ2015; 1(all), 1(zf), 2(zf), 4, 8, 5, 6, 3, NJ2015; 1(all), 1(zf), 2(all), 2(zf), 1(ff), 4, 8, 5, 6, 3, BLUP; 6, WH2014 |  |  |  |  |  |  | miR1516b |
| q9-2 | Linolenic acid | 9 | Marker499778, Marker526905 | -0.15~-0.1 | 3.47~5.06 | 3.57~5.12 | 2(all), WH2014; 2(ff), BLUP; 2(ff), EZ2015 |  |  |  |  |  |  |  |
| q9-3 | Oil content | 9 | Marker413883, Marker493960 | 0.07~0.43 | 3.35~4.98 | 1.6~4.32 | EZ2015, 8; EZ2015, 4; EZ2015, 5; EZ2015, BLUP, 3; 1, EZ2015; EZ2015, 7; EZ2015, 6 |  |  |  |  |  |  |  |
| q9-4 | Palmitic acids | 9 | Marker445069, Marker544266 | 0.09~0.13 | 3.14~9.43 | 2.74~9.39 | 1(all), EZ2015; 2(ff), BLUP |  |  |  |  |  |  |  |
| q9-5 | Palmitic acids | 9 | Marker466246, Marker434448 | 0.11~0.12 | 3.07~3.08 | 2.05~4.05 | 7, 3, NJ2015 |  |  |  |  |  |  |  |
| q9-6 | Palmitic acids | 9 | Marker448711, Marker448711 | -0.09~-0.06 | 3.04~6.86 | 1.42~3.12 | 1(all), 3, 4, EZ2015 |  | *Glyma09g36920* | *GmPLA1* | *At1g31480* | Phospholipase A1 | SGR2, a phospholipase-like protein, and ZIG/SGR4, a SNARE, are involved in the shoot gravitropism of Arabidopsis |  |
| q9-7 | Palmitic acids | 9 | Marker484248, Marker452814 | -0.1~-0.1 | 3.5~6.24 | 4.7~6.11 | 2(ff), EZ2015; 2(ff), BLUP |  | *Glyma09g38440* | *GmBCCP1* | *At5g16390* | Biotin Carboxyl Carrier Protein; subunit of Heteromeric ACCase | Reverse-genetic analysis of the two biotin-containing subunit genes of the heteromeric acetyl-coenzyme a carboxylase in *Arabidopsis* indicates a unidirectional functional redundancy | miR169u |
| q9-8 | Oleic acid | 9 | Marker516667, Marker442617 | 0.58 ~0.98 | 3.12~4.59 | 1.23~5.48 | 2(ff), WH2014; 2(ff), NJ2025; 2(ff), BLUP; 2(all), NJ2016; 1(ff), NJ2015 |  | *Glyma09g38570* | *GmSEI* | *At5g16460* | Seipin1 protein | Overexpression of *seipin1* increases oil in hydroxy fatty acid-accumulating seeds |  |
| q9-9 | Linolenic acid | 9 | Marker501874, Marker411800 | -0.11~-0.08 | 2.58~3.64 | 1.85~3.02 | 2(all), 3, WH2014; 1(ff), 2(all), BLUP |  |  |  |  |  |  |  |
| q9-10 | Linoleic acid | 9 | Marker412151, Marker470827 | -0.54~-0.42 | 2.62~5.61 | 3.64~3.71 | 6, WH2014; 2(zf), BLUP |  | *Glyma09g40130* | *GmHEX* | *At4g00730* | Homeobox-leucine zipper family protein |  |  |
| q9-11 | Linoleic acid | 9 | Marker442978, Marker524438 | -0.94~-0.29 | 3.24~6.56 | 2.83~10.05 | 2(all), 2(ff), EZ2015; 7, BLUP |  | *Glyma09g41040* | *GmALA1* | *At5g04930* | Aminophospholipid ATPase | Chilling tolerance in *Arabidopsis* involves *ALA1*, a member of a new family of putative aminophospholipid translocases |  |
| q9-12 | Oleic acid | 9 | Marker532651, Marker524438 | 0.47~0.94 | 2.52~5.3 | 1.59~7.14 | 2(all), BLUP; 2(zf), NJ2023; 2(all), 2(ff), EZ2015 |  |  |  |  |  |  |  |
| q9-13 | Linolenic acid | 9 | Marker507211, Marker426761 | -0.19~-0.09 | 3.02~6.79 | 1.22~5.47 | 2(all), 1(all), 1(ff), 8, 3, EZ2015 |  |  |  |  |  |  |  |
| q9-14 | Linoleic acid | 9 | Marker474222, Marker473030 | -0.55~-0.35 | 4.07~5.07 | 2.73~3.26 | 3, EZ2015; 2(all), BLUP |  | *Glyma09g41380* | *GmKASIII* | *At1g62640* | Ketoacyl-ACP Synthase III | De novo biosynthesis of fatty acids plays critical roles in the response of the photosynthetic machinery to low temperature in *Arabidopsis* |  |
| q9-15 | Oleic acid | 9 | Marker439035, Marker489860 | 0.52~1.16 | 2.72~7.61 | 1.57~7.87 | 1(ff), BLUP; 1(all), 1(ff), EZ2015 |  |  |  |  |  |  |  |
| q9-16 | Linoleic acid | 9 | Marker426761, Marker426761 | -0.95~-0.32 | 3.69~13.1 | 2.2~7.92 | 1(all), 6, 3, BLUP; 1(all), 1(ff), 4, 6, EZ2015 |  |  |  |  |  |  |  |
| q10-1 | Linolenic acid | 10 | Marker1079621, Marker1077464 | 0.05~0.22 | 3.22~4.38 | 0.46~7.89 | 1(all), 1(zf), 6, 2(zf), EZ2015 |  | *Glyma10g01420* | *GmGPAT2* | *At1g02660* | Fatty Acyl omega-Hydroxylase | Functional analysis of the *LACERATA* gene of *Arabidopsis* provides evidence for different robes of fatty acid omega-hydroxylation in development | miR4393a, miR10186a, miR164b |
| q10-2 | Stearic acid | 10 | Marker1056846, Marker1027312 | -0.07~-0.07 | 2.88~2.98 | 2.61~4.57 | 1(all), 2(all), EZ2015; 6, BLUP |  |  |  |  |  |  |  |
| q10-3 | Linoleic acid | 10 | Marker1043351, Marker1139002 | -0.69~-0.33 | 2.81~3.12 | 1.11~6 | 2(zf), 3, NJ2015 |  |  |  |  |  |  |  |
| q10-4 | Oleic acid | 10 | Marker1116659, Marker1083703 | 0.94~1.03 | 3.53~4.42 | 3.6~8.29 | 1(zf), 2(zf), WH2014 |  | *Glyma10g32520* | *GmTGD1* | *At1g19800* | Permease-like Protein of Inner Chloroplast Envelope | A permease-like protein involved in ER to thylakoid lipid transfer in *Arabidopsis* | miR1530, miR166f, miR10189 |
| q10-5 | Linoleic acid | 10 | Marker1116659, Marker1015006 | -0.86~-0.61 | 3.71~4.66 | 2.28~9 | 1(zf), 2(zf), 2(ff), 1(all), 5, WH2014 |  | *Glyma10g32660* | *GmCDP-DAGS* | *At4g22340* | CDP-DAG Synthase | Extraplastidial cytidinediphosphate diacylglycerol synthase activity is required for vegetative development in *Arabidopsis* *thaliana* | miR9766, miR10186g |
| q10-6 | Oil content | 10 | Marker1083703, Marker1042886 | -0.39~-0.1 | 3.13~6.31 | 0.7~3.95 | EZ2015, 3; EZ2015, BLUP, 5; EZ2015, 6; 1, 2, EZ2015; EZ2015, 7; EZ2015, 8; EZ2015, 4 |  | *Glyma10g34150* | *GmMDH2* | *At5g58330* | oxidoreductase activity | Interorganelle communication: Peroxisomal MALATE DEHYDROGENASE2 connects lipid catabolism to photosynthesis through redox coupling in *Chlamydomonas*. | miR9766 |
| q10-7 | Oleic acid | 10 | Marker1084140, Marker1006569 | 0.44~0.7 | 2.81~6.43 | 1.32~3.9 | 1(all), 2(ff), BLUP; 1(all), WH2014 |  |  |  |  |  |  | miR9767, miR9724b, miR393i, miR4388 |
| q10-8 | Linolenic acid | 10 | Marker1084140, Marker1056155 | -0.2~-0.05 | 2.97~4.92 | 0.76~4.87 | 2(ff), EZ2015; 1(all), 6, BLUP; 5, 6, 4, 8, 3, WH2014 |  |  |  |  |  |  | miR399a, miR399m, miR167f, miR167h |
| q10-9 | Palmitic acids | 10 | Marker1069694, Marker1006569 | -0.07~-0.24 | 2.61~9.96 | 3.52~9.17 | 1(all), 2(all), 2(ff), 5, 8, 3, 4, 1(zf), 2(zf), WH2014 |  |  |  |  |  |  | miR393i, miR4388 |
| q11-1 | Stearic acid | 11 | Marker615576, Marker615576 | 0.001-0.003 | 3.26~7.66 | 0~0.02 | 6, WH2014; 6, BLUP |  |  |  |  |  |  |  |
| q11-2 | Palmitic acids | 11 | Marker563276, Marker623460 | 0.03~0.07 | 3.06~4.02 | 0.28~2.4 | 3, 2(all), BLUP; 6, EZ2015 |  |  |  |  |  |  |  |
| q11-3 | Linoleic acid | 11 | Marker619127, Marker624447 | -0.7~-0.61 | 3.1~3.34 | 2.35~6 | 2(zf), 1(zf), WH2014 |  |  |  |  |  |  |  |
| q11-4 | Oleic acid | 11 | Marker618984, Marker651413 | -0.71~-0.31 | 2.5~12.7 | 1.45~3.1 | 2(all), BLUP; 2(all), NJ2017 |  |  |  |  |  |  | miR4370 |
| q11-5 | Linolenic acid | 11 | Marker555871, Marker654290 | 0.09~0.13 | 3.44~4.48 | 1.57~2.85 | 2(all), 1(all), 6, EZ2015 |  |  |  |  |  |  | miR393a |
| q11-6 | Linoleic acid | 11 | Marker593174, Marker593174 | 0.42~0.46 | 4.45~5 | 1.79~2.13 | 6, 3, NJ2015 |  | *Glyma11g37320* | *GmKAR* | *At1g24360* | Ketoacyl-ACP Reductase | Crystallization of the NADP-dependent beta-keto acyl-carrier protein reductase from *Brassica napus* |  |
| q12-1 | Palmitic acids | 12 | Marker2737872, Marker2737872 | 0.07~0.09 | 3.46164.3205~ | 1.85~3.06 | 8, 4, WH2014 |  | *Glyma12g04440* | *GmbZIP44* | *At1g75390* |  |  |  |
|  |  |  | , |  |  |  |  |  | *Glyma12g04150* | *GmFBPase* | *At4g38970* | Sucrose biosynthesis | Increasing growth and yield by altering carbon metabolism in a transgenic leaf oil crop |  |
| q12-2 | Oleic acid | 12 | Marker2719781, Marker2718329 | 0.39~0.83 | 3.04~7.98 | 1.14~4.68 | 2(ff), NJ2026; 1(all), 2(all), BLUP; 2(all), NJ2018 |  | *Glyma12g05140* | *GmLACS2* | *At1g49430* | Long-Chain Acyl-CoA Synthetase | The acyl-CoA synthetase encoded by *LACS2* is essential for normal cuticle development in *Arabidopsis* |  |
| q12-3 | Linoleic acid | 12 | Marker2719781, Marker2643231 | -0.8~-0.38 | 2.89~8.7 | 1.6~7.55 | 2(all), 7, 2(ff), 3, NJ2015; 1(all), 2(all), 2(ff), 6, 3, BLUP |  |  |  |  |  |  | miR10424b |
| q12-4 | Stearic acid | 12 | Marker2668335, Marker2665552 | -0.06~0 | 4.7~17.25 | 0.03~6.56 | 6, BLUP; 4, 3, WH2014 |  |  |  |  |  |  |  |
| q12-5 | Linoleic acid | 12 | Marker2635450, Marker2711916 | -0.91~-0.68 | 3.98~7.69 | 4.64~10.67 | 2(zf), 1(all), 1(ff), 6, NJ2015 |  |  |  |  |  |  |  |
| q12-6 | Linoleic acid | 12 | Marker2729534, Marker2729534 | -0.87~-0.79 | 6.75~6.98 | 6.35~7.68 | 4, 8, NJ2015 |  | *Glyma12g08720* | *GmAAPT1* | *At1g13560* | Diacylglycerol Cholinephosphotransferase | Characterization of aminoalcoholphosphotransferases from *Arabidopsis* *thaliana* and soybean | miR172a, miR530d |
| q12-7 | Linolenic acid | 12 | Marker2711294, Marker2668578 | -0.22~-0.04 | 2.8~5.62 | 1.37~6.93 | 2(all), 2(ff), 3, BLUP; 7, EZ2015; 2(all), 4, 8, 2(ff), NJ2015 |  |  |  |  |  |  |  |
| q13-1 | Oleic acid | 13 | Marker2812929, Marker2824537 | 0.74~1.07 | 3.94~5.16 | 1.88~4.06 | 2(ff), NJ2015; 2(all), NJ2015 |  |  |  |  |  |  | miR10430 |
| q13-2 | Stearic acid | 13 | Marker2837293, Marker2837293 | 0.08~0.15 | 10.86~16.09 | 10.64~13.06 | 4, 5, 3, WH2014 |  |  |  |  |  |  |  |
| q13-3 | Oil content | 13 | Marker2839097, Marker2838693 | 0.73~2.19 | 50.05~51.37 | 12.09~17.94 | 2, EZ2015; 2(zf), BLUP |  |  |  |  |  |  |  |
| q13-4 | Stearic acid | 13 | Marker2798086, Marker2790748 | 0.09~0.12 | 3.26~19.94 | 5.29~9.33 | 1(zf), 2(zf), EZ2015; 6, 6, BLUP |  | *Glyma13g16560* | *GmDGAT1* | *At2g19450* | Acyl-CoA : Diacylglycerol Acyltransferase | The *TAG1* locus of *Arabidopsis* encodes for a diacylglycerol acyltransferase | miR156q |
| q13-5 | Linoleic acid | 13 | Marker2850221, Marker2850221 | -0.5~-0.43 | 2.84~4.03 | 1.83~1.97 | 1(all), 6, NJ2015 |  | *Glyma13g16790* | *GmPDAT1* | *At5g13640* | Phospholipid : Diacylglycerol Acyltransferase | Isolation and characterization of an *Arabidopsis* *thaliana* knockout line for phospholipid: diacylglycerol transacylase gene |  |
| q13-6 | Oleic acid | 13 | Marker2855002, Marker2858951 | 0.48~1.01 | 5.24~11.34 | 3.28~9.05 | 2(ff), 1(all), 1(ff), 2(all), BLUP |  |  |  |  |  |  | miR160d |
| q13-7 | Linoleic acid | 13 | Marker2855002, Marker2849746 | -0.65~-0.56 | 2.88~5.83 | 4.22~9.84 | 1(ff), 2(ff), BLUP; 2(ff), WH2014 |  |  |  |  |  |  |  |
| q13-8 | Linolenic acid | 13 | Marker2801439, Marker2823060 | -0.23~-0.12 | 3.21~6.89 | 2.4~8.8 | 2(all), 1(ff), 2(ff), 4, 8, 1(all), 6, EZ2015; 2(ff), BLUP |  | *Glyma13g23150* | *GmMGDGS* | *At5g20410* | Monogalactosyldiacylglycerol Synthase | Type-B monogalactosyldiacylglycerol synthases are involved in phosphate starvation-induced lipid remodeling, and are crucial for low-phosphate adaptation | miR1507a |
| q13-9 | Oleic acid | 13 | Marker2773797, Marker2793157 | 1.18~1.43 | 7.06~12.68 | 7.56~12.31 | 1(ff), 2(ff), 1(all), 2(all), EZ2015 |  |  |  |  |  |  | miR171k |
| q13-10 | Linoleic acid | 13 | Marker2773797, Marker2793157 | -1.87~-0.69 | 3.1~10.82 | 4.37~11.86 | 2(ff), 1(all), 2(all), 4, 8, 5, 6, 7, 3, EZ2015; 1(ff), WH2014 |  |  |  |  |  |  | miR396b, miR396a |
| q13-11 | Linoleic acid | 13 | Marker2817224, Marker2778921 | -1.12~-0.48 | 5.86~10.37 | 2.51~11.03 | 1(ff), EZ2015; 1(all), BLUP |  |  |  |  |  |  | miR319n |
| q13-12 | Linolenic acid | 13 | Marker2777375, Marker2783913 | -0.14~-0.08 | 4.07~5.66 | 1.45~4.52 | 4, 8, 7, 3, 1(all), WH2014; 2(all), BLUP |  | *Glyma13g25290* | *GmMAGL* | *At1g11090* | Monoacylglycerol Lipase (MAGL) |  | miR10431 |
| q13-13 | Linoleic acid | 13 | Marker2843351, Marker2783913 | -0.56~-0.34 | 3.72~9.24 | 2.73~7.8 | 2(all), 4, 8, 7, 3, BLUP |  |  |  |  |  |  |  |
| q13-14 | Linolenic acid | 13 | Marker2808968, Marker2749962 | -0.23~-0.2 | 3.39~4.62 | 6.44~7.97 | 2(zf), 1(zf), EZ2015 |  |  |  |  |  |  |  |
| q13-15 | Linolenic acid | 13 | Marker2853789, Marker2836308 | -0.17~-0.11 | 4.15~7.65 | 2.61~4.94 | 2(all), 7, EZ2015 |  | *Glyma13g29160* | *GmTCP* | *At4g18390* | TEOSINTE BRANCHED 1, cycloidea and PCF transcription factor |  | miR4389 |
| q13-16 | Linoleic acid | 13 | Marker2838644, Marker2796333 | 0.77~0.88 | 3.88~6.19 | 4.92~7.18 | 2(zf), 1(zf), WH2014 |  |  |  |  |  |  | miR171p |
| q13-17 | Oleic acid | 13 | Marker2824982, Marker2779484 | -1.13~-0.54 | 2.68~9.1 | 2.87~8.27 | 1(zf), WH2014; 2(all), 1(all), 1(zf), 2(zf), BLUP; 2(all), NJ2020; 2(all), 1(zf), 2(zf), EZ2015 |  | *Glyma13g44170* | *GmPLDα1* | *At3g15730* | Phospholipase D &alpha; | PLDα1-knockdown soybean seeds display higher unsaturated glycerolipid contents and seed vigor in high temperature and humidity environments | miR1532 |
| q13-18 | Linoleic acid | 13 | Marker2824982, Marker2779484 | 0.28~0.88 | 2.57~4.93 | 1.21~7.23 | 3, WH2014; 2(all), 1(zf), 8, 3, 2(zf), 4, EZ2015; 4, 3, 2(zf), BLUP |  |  |  |  |  |  |  |
| q14-1 | Linolenic acid | 14 | Marker1763102, Marker1641850 | -0.14~-0.1 | 3.61~5.33 | 1.77~3.72 | 7, 3, 2(all), EZ2015 |  |  |  |  |  |  |  |
| q14-2 | Palmitic acids | 14 | Marker1643781, Marker1640132 | 0.13~0.16 | 3.19~3.54 | 6.46~7.22 | 6, EZ2015; 2(zf), 1(zf), WH2014 |  | *Glyma14g04780* | *GmECR/CER10* | *At3g55360* | Enoyl-CoA Reductase | Disruptions of the *Arabidopsis* enoyl-CoA reductase gene reveal an essential role for very-long-chain fatty acid synthesis in cell expansion during plant morphogenesis |  |
| q14-3 | Oleic acid | 14 | Marker1701172, Marker1701172 | 0.68~0.99 | 4.33~6.34 | 2.49~9.48 | 1(all), 1(ff), EZ2015; 1(ff), BLUP |  |  |  |  |  |  |  |
| q14-4 | Linoleic acid | 14 | Marker1701172, Marker1623889 | -0.79~-0.26 | 2.91~7.89 | 1.61~10.34 | 1(all), 1(ff), 6, 2(ff), 3, EZ2015; 1(ff), 4, 8, 6, 3, BLUP; 1(all), 6, WH2014 |  |  |  |  |  |  |  |
| q14-5 | Stearic acid | 14 | Marker1630405, Marker1623889 | 0~-0.05 | 3.68~15.61 | 0~6.28 | 2(ff), WH2014; 6, BLUP |  |  |  |  |  |  |  |
| q14-6 | Oleic acid | 14 | Marker1626292, Marker1677544 | 0.63~1.16 | 4.67~13.9 | 2.26~10.8 | 2(ff), BLUP; 1(all), 1(ff), NJ2015; 2(all), NJ2021 |  |  |  |  |  |  |  |
| q14-7 | Linoleic acid | 14 | Marker1626292, Marker1640915 | -0.99~-0.48 | 3.32~8.54 | 2.02~12.34 | 2(ff), BLUP; 2(all), 2(ff), 6, 3, 1(all), NJ2015 |  | *Glyma14g06290* | *GmABI4* | *At2g40220* | Triacylglycerol Biosynthesis | The *Arabidopsis* abscisic acid response locus *ABI4* encodes an APETALA2 domain protein |  |
| q14-8 | Linolenic acid | 14 | Marker1627639, Marker1623656 | -0.08~-0.08 | 3.33~5.07 | 1.71~2.71 | 2(all), BLUP; 3, WH2014 |  |  |  |  |  |  | miR393e |
| q14-9 | Palmitic acids | 14 | Marker1658471, Marker1651052 | 0.03~0.09 | 3.17~6.89 | 0.29~3.06 | 1(ff), BLUP; 6, EZ2015 |  |  |  |  |  |  |  |
| q14-10 | Linoleic acid | 14 | Marker1690474, Marker1696882 | -0.86~-0.39 | 3.53~9.25 | 3.71~9.19 | 1(zf), 2(zf), WH2014; 1(zf), 2(all), 2(zf), 6, BLUP; 2(all), NJ2015 |  |  |  |  |  |  | miR9727, miR5678, miR5370 |
| q14-11 | Linoleic acid | 14 | Marker1687940, Marker1758694 | -0.71~-0.32 | 3.06~14.35 | 2.17~4.11 | 3, BLUP; 2(all), 8, 4, EZ2015 |  | *Glyma14g09380* | *GmSPT* | *At4g36480* | Long-Chain Base 1; subunit of Serine Palmitoyltransferase | Serine palmitoyltransferase, a key enzyme for de novo synthesis of sphingolipids, is essential for male gametophyte development in *Arabidopsis* |  |
| q14-12 | Linoleic acid | 14 | Marker1634367, Marker1620782 | -1.41~-0.55 | 4.21~7.98 | 3.04~9.61 | 2(zf), 1(all), 1(zf), 4, 8, 5, 6, 3, NJ2015 |  |  |  |  |  |  |  |
| q14-13 | Oil content | 14 | Marker1673567, Marker1691565 | -0.19~-0.03 | 3.1~5.11 | 0.29~3.25 | WH2014, 6; 1(all), 2(all), BLUP; 1, WH2014; BLUP, 8 |  |  |  |  |  |  | miR4345, miR9734, miR394b |
| q15-1 | Oleic acid | 15 | Marker39573, Marker39573 | -0.39~-0.39 | 2.87~4.77 | 0.88~1.6 | 1(all), BLUP |  | *Glyma15g03030* | *GmLOX* | *At1g55020* | Lipoxygenase | Oxylipins produced by the 9-lipoxygenase pathway in *Arabidopsis* regulate lateral root development and defense responses through a specific signaling cascade |  |
| q15-2 | Linolenic acid | 15 | Marker10292, Marker45535 | 0.05~0.19 | 3.81~10.85 | 0.99~5.07 | 1(all), 6, NJ2015; 1(ff), 6, BLUP |  |  |  |  |  |  | miR171f |
| q15-3 | Oil content | 15 | Marker135457, Marker93680 | -0.22~-0.12 | 2.6~6.11 | 0.64~3.17 | 1(all), 6, WH2014 |  | *Glyma15g16270* | *GmPLDζ* | *At3g16785* | Phospholipase D &zeta; | *Phospholipase Dζ* enhances diacylglycerol flux into triacylglycerol | miR4347 |
| q15-4 | Linolenic acid | 15 | Marker115198, Marker30871 | 0~0.14 | 3.18~5.38 | 0~3.44 | 3, 2(all), 1(all), EZ2015 |  |  |  |  |  |  | miR169n, miR169d, miR169p, miR169e |
| q15-5 | Linoleic acid | 15 | Marker53417, Marker47707 | -0.75~-0.75 | 3.82~4.59 | 6.7~6.98 | 1(ff), 2(ff), WH2014 |  | *Glyma15g18770* | *GmPMT* | *At5g57020* | Protein N-Myristoyltransferase | N-myristoylation regulates the *SnRK1* pathway in *Arabidopsis* |  |
| q15-6 | Stearic acid | 15 | Marker63713, Marker18787 | 0.03~2.17 | 15.64~32.26 | 6.29~43.53 | 6, 6, 6, BLUP; 1(all), 6, NJ2015 |  |  |  |  |  |  | miR10436 |
| q15-7 | Oil content | 15 | Marker74696, Marker90244 | -0.27~-0.14 | 3.07~4.5 | 1.58~2.04 | WH2014, 3; 2, WH2014 |  |  |  |  |  |  |  |
| q15-8 | Oil content | 15 | Marker50563, Marker38327 | -0.12~-0.06 | 2.96~3.94 | 1.09~5.24 | BLUP, 6; 1(ff), 2(ff), 1(all), BLUP; BLUP, 8 |  |  |  |  |  |  |  |
| q16-1 | Linoleic acid | 16 | Marker2581493, Marker2581493 | 0.26~0.67 | 2.84~6.62 | 1.02~3.96 | 1(all), 4, 6, 3, EZ2015; 4, 8, 5, 6, 3, BLUP; 3, WH2014 |  |  |  |  |  |  |  |
| q16-2 | Oleic acid | 16 | Marker2576734, Marker2518248 | -0.92~-0.85 | 3.47~4.03 | 4.95~5.61 | 1(ff), 2(ff), EZ2015 |  | *Glyma16g02090* | *Gmalpha-PDHC* | *At1g01090* | Pyruvate Dehydrogenase alpha subunit, E1a component of Pyruvate Dehydrogenase Complex | Pyruvate-dehydrogenase (*pdh*) deficiency caused by a 21-base pair insertion mutation in the e1-alpha subunit |  |
| q16-3 | Linoleic acid | 16 | Marker2576734, Marker2518248 | 0.63~0.73 | 3.1~3.82 | 3.21~6.05 | 1(ff), 2(all), 2(ff), EZ2015 |  |  |  |  |  |  |  |
| q16-4 | Palmitic acids | 16 | Marker2592949, Marker2554554 | 0.09~0.2 | 2.68~7.1 | 3.45~7 | 1(ff), 2(ff), NJ2015; 1(ff), 2(ff), BLUP |  | *Glyma16g26210* | *GmSPT* | *At5g23670* | Long-Chain Base 1; subunit of Serine Palmitoyltransferase | Serine palmitoyltransferase, a key enzyme for de novo synthesis of sphingolipids, is essential for male gametophyte development in *Arabidopsis* | miR5041, miR5767 |
| q16-5 | Palmitic acids | 16 | Marker2595561, Marker2595561 | 0.06~0.07 | 3.81~3.97 | 1.48~2.46 | 2(all), BLUP; 7, EZ2015 |  | *Glyma16g27430* | *GmHAD* | *At5g60340* | Hydroxyacyl-ACP Dehydrase | Proof of function of a putative 3-hydroxyacyl-acyl carrier protein dehydratase from higher plants by mass spectrometry of product formation | miR1510a |
| q16-6 | Palmitic acids | 16 | Marker2539386, Marker2569418 | 0.07~0.1 | 3.07~3.15 | 2.09~3.75 | 8, 4, 2(all), EZ2015 |  |  |  |  |  |  |  |
| q17-1 | Stearic acid | 17 | Marker226711, Marker169904 | -0.15~-0.07 | 3.13~3.59 | 3.06~6.12 | 2(zf), 4, 5, EZ2015 |  | *Glyma17g00950* | *GmLEC1-b* | *At5g47670* | NF-Y Family Transcription Factors | Enhanced seed oil production in canola by conditional expression of *Brassica* *napus* *LEAFY COTYLEDON*1 and LEC1-LIKE in developing seeds |  |
| q17-2 | Oil content | 17 | Marker211094, Marker159523 | -0.27~-0.05 | 3.13~10.47 | 0.84~3.77 | 2, EZ2015; BLUP, 7; BLUP, 6; 2(all), BLUP; BLUP, EZ2015, 3 |  |  |  |  |  |  |  |
| q17-3 | Stearic acid | 17 | Marker224242, Marker224242 | 0~0.01 | 8.37~27.68 | 0.01~0.45 | 6, 7, BLUP |  |  |  |  |  |  |  |
| q17-4 | Stearic acid | 17 | Marker204502, Marker224596 | 0~0.01 | 7.19~29.92 | 0~25.75 | 6, 7, 7, BLUP |  |  |  |  |  |  |  |
| q17-5 | Stearic acid | 17 | Marker217381, Marker217381 | -0.03~0 | 4.98~52.63 | 1.65~19.49 | 7, NJ2015; 6, 7, BLUP |  |  |  |  |  |  |  |
| q17-6 | Stearic acid | 17 | Marker150342, Marker150342 | 0~0.01 | 4.39~33.07 | 0~0.15 | 6, 7, BLUP |  |  |  |  |  |  |  |
| q17-7 | Stearic acid | 17 | Marker152218, Marker152218 | 0~0.01 | 7.94~35.38 | 0.02~26.89 | 6, 7, BLUP |  |  |  |  |  |  |  |
| q18-1 | Linolenic acid | 18 | Marker864664, Marker2537405 | -0.22~0.1 | 2.78~5.42 | 1.64~5.25 | 2(all), 3, EZ2015; 1(zf), NJ2015 |  | *Glyma18g01280* | *GmKAR* | *At4g39850* | Triacylglycerol & Fatty Acid Degradation | The *Arabidopsis* pxa1 mutant is defective in an ATP-binding cassette transporter-like protein required for peroxisomal fatty acid beta-oxidation | miR4355, miR393f, miR172c, miR10441 |
| q18-2 | Linolenic acid | 18 | Marker955126, Marker955126 | -0.35~-0.04 | 3.2~6 | 0.67~5.38 | 6, BLUP; 1(all), 4, 8, 5, 6, 7, 3, EZ2015 |  | *Glyma18g50580* | *GmKASI* | *At5g46290* | Ketoacyl-ACP Synthase I | *Arabidopsis* beta-ketoacyl- acyl carrier protein synthase i is crucial for fatty acid synthesis and plays a role in chloroplast division and embryo development |  |
| q19-1 | Linoleic acid | 19 | Marker1590191, Marker1558435 | -0.79~-0.27 | 2.7~8.19 | 1.63~4.57 | 6, 7, 3, 1(all), EZ2015; 5, 6, 7, 1(all), 4, 8, 3, 2(all), BLUP; 4, 8, WH2014 |  |  |  |  |  |  | miR166k |
| q19-2 | Stearic acid | 19 | Marker1565978, Marker1460807 | 0~0.06 | 3.64~38.15 | 0~5.15 | 2(zf), WH2014; 6, BLUP; |  | *Glyma19g31610* | *GmFAX1* | *At3g57280* | Transmembrane proteins | Seed-specific overexpression of *AtFAX1* increases seed oil content in *Arabidopsis* |  |
| q19-3 | Linolenic acid | 19 | Marker1459716, Marker1535914 | -0.15~-0.08 | 3.39~4.59 | 1.13~4 | 7, 3, 1(all), 2(ff), EZ2015 |  |  |  |  |  |  | miR4995 |
| q20-1 | Oil content | 20 | Marker1310693, Marker1330183 | 0.05~1.31 | 2.67~47.38 | 0.2~11.46 | 2(ff), 1(ff), BLUP; WH2014, 6; 2, 1, 1, WH2014; WH2014, 8 |  | *Glyma20g01680* | *GmPIPK-III* | *At1g71010* | Phosphatidylinositol-Phosphate Kinase type III |  | miR4400 |
| q20-2 | Oleic acid | 20 | Marker1309940, Marker1326555 | 0.4~1.11 | 3.58~6.58 | 0.96~11.6 | 2(all), 1(zf), 2(zf), BLUP; 2(zf), EZ2015 |  |  |  |  |  |  | miR2118a |
| q20-3 | Linoleic acid | 20 | Marker1439858, Marker1326555 | -0.91~-0.53 | 3.39~4.43 | 6.45~8.67 | 2(zf), EZ2015; 2(zf), BLUP |  |  |  |  |  |  | miR482b |
| q20-4 | Oil content | 20 | Marker1420664, Marker1411877 | -0.51~-0.07 | 2.63~8.09 | 1.6~5.32 | 2(all), BLUP; WH2014, BLUP, 3; WH2014, 5; WH2014, BLUP, 7; WH2014, 6; 1, 1, 2, WH2014; WH2014, 8; WH2014, BLUP, 4 |  | *Glyma20g31630* | *GmDHLAT* | *At1g34430* | Dihydrolipoamide Acetyltransferase, E2 component of Pyruvate Dehydrogenase Complex | Disruption of *plE2*, the gene for the E2 subunit of the plastid pyruvate dehydrogenase complex, in *Arabidopsis* causes an early embryo lethal phenotype. |  |
| q20-5 | Linolenic acid | 20 | Marker1411877, Marker1429663 | 0.03~0.15 | 3.16~6.72 | 0.36~4.64 | 1(all), 1(ff), 3, BLUP; 3, EZ2015; 2(all), WH2014 |  | *Glyma20g31200* | *GmSBH1* | *At1g69640* | Sphingobase C4-Hydroxylase | Systematic analysis of protein subcellular localization and interaction using high-throughput transient transformation of *Arabidopsis* seedlings | miR1531, miR160b, miR1520b |
| q20-6 | Linolenic acid | 20 | Marker1356664, Marker1396693 | 0~0.08 | 3.6~3.89 | 0~2.91 | 6, WH2014; 2(all), BLUP |  |  |  |  |  |  | miR172j |
| q20-7 | Oil content | 20 | Marker1425110, Marker1425110 | -0.26~-0.03 | 3.59~3.75 | 0.31~6.96 | BLUP, 6; 1, WH2014 |  | *Glyma20g36090* | *GmPI5P-II* | *At1g65580* | Phosphoinositide 5-Phosphatase Type II |  |  |
| q20-8 | Linolenic acid | 20 | Marker1344810, Marker1344810 | -0.14~-0.12 | 3.22~3.39 | 1.65~2.23 | 6, 3, NJ2015 |  |  |  |  |  |  | miR4407 |

Note: The methods GCIM, ICIM, ISIS EM-BLASSO, mrMLM, FASTmrEMMA, pLARmEB, pKWmEB, and FASTmrMLM were indicated by 1 ~ 8, respectively.

**Table S9.** 36 significant QTL-by-environment interactions for oil-related traits and their candidate genes

| **QE** | **Trait** | **Pop ^a^** | **QTLs × environment (QEs)** | | | | | | | | | | | | | | |  | **Comparative genomics** | | | | |
| --- | --- | --- | --- | --- | --- | --- | --- | --- | --- | --- | --- | --- | --- | --- | --- | --- | --- | --- | --- | --- | --- | --- | --- |
|  |  |  | **Chr** | **Pos** | **Markers associated** | **LOD** | **LOD (A)** | **LOD**  **(A×E)** | **PVE (%)** | **PVE (A)** | **PVE (A×E)** | **Add** | **A×E1** | **A×E2** | **A×E3** | **Left CI** | **Right CI** |  | **Candidate**  **gene ID** | **Candidate**  **gene** | **Arabidopsis homologs** | **Protein**  **Family** | **Reference** |
| QE1 | Linoleic acid | All | 5 | 0 | Marker2095085, Marker2200075 | 7.18 | 4.44 | 2.74 | 3.07 | 2.21 | 0.86 | 0.32 | 0.11 | -0.28 | 0.17 | 0 | 0.5 |  | *Glyma05g09160* | *GmLTP* | *At3g18280* | Lipid Transfer Protein |  |
| QE2 |  | All | 5 | 107 | Marker2152470, Marker2100085 | 13.68 | 5.95 | 7.73 | 5.65 | 2.85 | 2.80 | 0.37 | 0.48 | -0.08 | -0.40 | 106.5 | 107 |  | *Glyma05g33790* | *GmPEAMT* | *At3g18000* | Phosphoethanolamine N-Methyltransferase | Translational regulation of Arabidopsis XIPOTL1 is modulated by phosphocholine levels via the phylogenetically conserved upstream open reading frame 30 |
| QE3 |  | All | 7 | 40 | Marker366921, Marker280587 | 9.80 | 5.14 | 4.66 | 4.78 | 2.55 | 2.22 | -0.35 | 0.33 | 0.11 | -0.44 | 39.5 | 40.5 |  | *Glyma07g08740* | *GmPLDβ4* | *At2g42010* | Phospholipase D &beta; | Arabidopsis phospholipase D1 modulates defense responses to bacterial and fungal pathogens |
| QE4 |  | All | 12 | 66 | Marker2719781, Marker2675339 | 10.94 | 6.85 | 4.10 | 5.35 | 3.40 | 1.95 | -0.40 | 0.29 | 0.13 | -0.42 | 65.5 | 67.5 |  | *Glyma12g07230* | *GmDIR10* | *At2g28670* | Disease resistance-responsive family protein / dirigent protein | Root Suberin Forms an Extracellular Barrier That ARCects Water Relations and Mineral Nutrition in Arabidopsis |
| QE5 |  | All | 14 | 21 | Marker1609710, Marker1671474 | 5.85 | 0.91 | 4.94 | 2.29 | 0.45 | 1.85 | -0.14 | -0.41 | 0.15 | 0.26 | 20.5 | 21.5 |  | *Glyma14g07290* | *GmGPAT5* | *At3g11430* | Glycerol-3-Phosphate Acyltransferase | The acyltransferase GPAT5 is required for the synthesis of suberin in seed coat and root of Arabidopsis |
| QE6 |  | All | 14 | 26 | Marker1690474, Marker1651052 | 5.50 | 1.96 | 3.54 | 2.53 | 0.96 | 1.58 | -0.21 | 0.15 | 0.23 | -0.38 | 25.5 | 26.5 |  | *Glyma14g08400* | *GmLPAAT4* | *At1g75020* | 1-Acylglycerol-3-Phosphate Acyltransferase | Ubiquitous and endoplasmic reticulum-located lysophosphatidyl acyltransferase, LPAT2, is essential for female but not male gametophyte development in Arabidopsis |
| QE7 |  | RC | 1 | 38 | Marker1912349, Marker1788124 | 5.55 | 2.85 | 2.70 | 3.83 | 2.06 | 1.78 | -0.34 | 0.15 | 0.29 | -0.44 | 37.5 | 38.5 |  | *Glyma01g37710* | *GmLPP-ε2* | *At5g66450* | Phosphatidate Phosphatase | Plastidic phosphatidic acid phosphatases identified in a distinct subfamily of lipid phosphate phosphatases with prokaryotic origin |
| QE8 |  | RC | 13 | 97 | Marker2773797, Marker2775509 | 8.24 | 5.70 | 2.54 | 7.10 | 4.18 | 2.93 | -0.50 | 0.18 | -0.58 | 0.39 | 95.5 | 97.5 |  | *Glyma13g22230* | *GmHACPS* | *At3g11470* | Holo-ACP Synthase | A new enzyme superfamily - The phosphopantetheinyl transferases |
| QE9 |  | OC | 12 | 57 | Marker2635450, Marker2665552 | 6.25 | 3.09 | 3.17 | 4.37 | 2.47 | 1.90 | -0.43 | -0.03 | 0.47 | -0.44 | 56.5 | 57.5 |  | *Glyma12g07230* | *GmDIR10* | *At2g28670* | Disease resistance-responsive family protein / dirigent protein | Root Suberin Forms an Extracellular Barrier That ARCects Water Relations and Mineral Nutrition in Arabidopsis |
| QE10 | Linolenic acid | All | 6 | 40 | Marker2003189, Marker2014747 | 10.05 | 5.24 | 4.81 | 2.38 | 1.84 | 0.54 | 0.09 | 0.07 | -0.02 | -0.05 | 39.5 | 40.5 |  | *Glyma06g08290* | *GmOBO* | *At2g25890* | Oil-Body Oleosin | A novel group of oleosins is present inside the pollen of Arabidopsis |
| QE11 |  | RC | 2 | 162 | Marker1165825, Marker1280449 | 7.69 | 2.04 | 5.65 | 3.34 | 1.45 | 1.89 | -0.07 | 0.08 | -0.12 | 0.04 | 161.5 | 164.5 |  | *Glyma02g03851* | *GmACBP* | *At4g27780* | Acyl CoA Binding Protein | Acyl-CoA-binding protein 2 binds lysophospholipase 2 and lysoPC to promote tolerance to cadmium-induced oxidative stress in transgenic Arabidopsis |
| QE12 |  | RC | 9 | 15 | Marker443649, Marker526905 | 6.62 | 3.77 | 2.85 | 3.48 | 2.66 | 0.82 | -0.10 | 0.07 | -0.06 | -0.01 | 14.5 | 16.5 |  | *Glyma09g04620* | *GmPLDζ3* | *At3g16785* | Phospholipase D &zeta; | The Arabidopsis phospholipase D family Characterozation of a calcium-independent and phosphatidylcholine-selective PLD zeta 1 with distinct regulatory domains |
| QE13 |  | RC | 10 | 118 | Marker1077949, Marker1065285 | 6.29 | 3.61 | 2.68 | 3.06 | 2.55 | 0.51 | -0.10 | -0.05 | 0.06 | -0.01 | 116.5 | 119.5 |  | *Glyma10g06855* | *GmSac-PIP* | *At1g22620* | Sac domain-containing Phosphoinositide Phosphatase | Mutation of SAC1, an Arabidopsis SAC domain phosphoinositide phosphatase, causes alterations in cell morphogenesis, cell wall synthesis, and actin organization |
| QE14 |  | RC | 13 | 96 | Marker2773797, Marker2775509 | 7.08 | 3.52 | 3.56 | 3.12 | 2.42 | 0.69 | -0.10 | 0.05 | -0.07 | 0.03 | 94.5 | 97.5 |  | *Glyma13g22230* | *GmHACPS* | *At3g11470* | Holo-ACP Synthase | A new enzyme superfamily - The phosphopantetheinyl transferases |
| QE15 |  | RC | 14 | 13 | Marker1751911, Marker1655668 | 8.34 | 4.14 | 4.20 | 3.87 | 2.93 | 0.94 | -0.11 | 0.06 | -0.08 | 0.03 | 12.5 | 13.5 |  | *Glyma14g04780* | *GmECR/CER10* | *At3g55360* | Enoyl-CoA Reductase | Disruptions of the Arabidopsis enoyl-CoA reductase gene reveal an essential role for very-long-chain fatty acid synthesis in cell expansion during plant morphogenesis |
| QE16 |  | OC | 6 | 64 | Marker2000797, Marker1997145 | 7.43 | 4.69 | 2.74 | 8.06 | 4.84 | 3.22 | -0.12 | 0.03 | -0.13 | 0.10 | 63.5 | 64.5 |  | *Glyma06g11650* | *GmPIPK-IB* | *At1g21920* | Phosphatidylinositol-Phosphate Kinase type IB |  |
| QE17 | Oil content | RC | 7 | 71 | Marker301297, Marker306430 | 32.02 | 19.76 | 12.26 | 27.37 | 13.07 | 14.30 | 0.46 | 0.48 | -0.48 | NA | 70.5 | 71.5 |  | *Glyma07g13780* | *GmPAH1* | *At4g37070* | Acyl-Hydrolase (Patatin-like) | Roles of Arabidopsis Patatin-Related Phospholipases A in Root Development Are Related to Auxin Responses and Phosphate Deficiency |
| QE18 |  | OC | 20 | 51 | Marker1374027, Marker1373278 | 3.75 | 1.05 | 2.70 | 0.67 | 0.38 | 0.29 | -0.13 | -0.11 | 0.11 | NA | 49.5 | 51.5 |  | *Glyma20g30570* | *GmSD* | *At1g43710* | serine decarboxylase |  |
| QE19 | Oleic acid | All | 5 | 107 | Marker2152470, Marker2100085 | 8.72 | 4.86 | 3.86 | 2.85 | 1.99 | 0.86 | -0.44 | -0.41 | 0.18 | 0.23 | 106.5 | 107 |  | *Glyma05g33790* | *GmPEAMT* | *At3g18000* | Phosphoethanolamine N-Methyltransferase | Translational regulation of Arabidopsis XIPOTL1 is modulated by phosphocholine levels via the phylogenetically conserved upstream open reading frame 30 |
| QE20 |  | All | 14 | 12 | Marker1626292, Marker1677544 | 6.56 | 2.76 | 3.80 | 3.09 | 1.15 | 1.94 | 0.33 | -0.25 | -0.36 | 0.61 | 11.5 | 12.5 |  | *Glyma14g05510* | *GmTAGL* | *At5g14180* | Triacylglycerol Lipase (TAGL) | Antibiosis against the green peach aphid requires the Arabidopsis thaliana MYZUS PERSICAE-INDUCED LIPASE1 gene |
| QE21 |  | RC | 13 | 96 | Marker2773797, Marker2775509 | 10.57 | 7.49 | 3.08 | 8.35 | 5.86 | 2.49 | 0.72 | -0.39 | 0.66 | -0.27 | 94.5 | 97.5 |  | *Glyma13g22230* | *GmHACPS* | *At3g11470* | Holo-ACP Synthase | A new enzyme superfamily - The phosphopantetheinyl transferases |
| QE22 |  | RC | 14 | 12 | Marker1630405, Marker1623889 | 6.39 | 3.58 | 2.81 | 4.93 | 2.81 | 2.13 | 0.49 | -0.05 | 0.54 | -0.49 | 11.5 | 12.5 |  | *Glyma14g04440* | *GmTGD2* | *At3g20320* | Phosphatidic Acid-Binding Protein | A phosphatidic acid-binding protein of the chloroplast inner envelope membrane involved in lipid trarcicking |
| QE23 |  | OC | 4 | 74 | Marker2357108, Marker2283904 | 3.53 | 0.57 | 2.96 | 2.11 | 0.42 | 1.69 | -0.22 | -0.62 | 0.20 | 0.42 | 73.5 | 74.5 |  | *Glyma04g04060* | *GmPAH2* | *At5g42870* | Phosphatidate Phosphatase | PHOSPHATIDIC ACID PHOSPHOHYDROLASE1 and 2 Regulate Phospholipid Synthesis at the Endoplasmic Reticulum in Arabidopsis |
| QE24 | Palmitic acid | All | 2 | 106 | Marker1193624, Marker1221985 | 5.19 | 1.32 | 3.87 | 1.26 | 0.59 | 0.67 | -0.04 | -0.06 | 0.04 | 0.02 | 105.5 | 106.5 |  | *Glyma02g14960* | *GmPI3P* | *At3g19240* | Phosphoinositide 3-Phosphatase |  |
| QE25 |  | All | 3 | 103 | Marker2483347, Marker2504505 | 11.76 | 3.10 | 8.66 | 2.90 | 1.39 | 1.51 | 0.06 | 0.09 | -0.03 | -0.05 | 102.5 | 103.5 |  | *Glyma03g32251* | *GmPLAT* | *At1g04010* | Phospholipid : Acyl acceptor Acyltransferase | Involvement of the phospholipid sterol acyltransferase1 in plant sterol homeostasis and leaf senescence |
| QE26 |  | All | 6 | 35 | Marker1964032, Marker2029409 | 13.44 | 8.29 | 5.15 | 4.10 | 3.73 | 0.37 | 0.10 | 0.04 | -0.03 | -0.01 | 34.5 | 35.5 |  | *Glyma06g07230* | *GmPLDα6* | *At3g15730* | Phospholipase D &alpha; | Phospholipase d alpha 1 and phosphatidic acid regulate nadph oxidase activity and production of reactive oxygen species in ABA-mediated stomatal closure in arabidopsis |
| QE27 |  | All | 10 | 30 | Marker1084140, Marker1069694 | 9.33 | 5.25 | 4.08 | 2.55 | 2.32 | 0.24 | -0.08 | -0.03 | 0.01 | 0.02 | 27.5 | 31.5 |  | *Glyma10g35960* | *GmDHLAT* | *At1g34430* | Dihydrolipoamide Acetyltransferase, E2 component of Pyruvate enase Complex |  |
| QE28 |  | RC | 3 | 129 | Marker2470244, Marker2415983 | 3.51 | 0.08 | 3.43 | 2.07 | 0.06 | 2.01 | 0.01 | 0.05 | 0.04 | -0.09 | 124.5 | 133.5 |  | *Glyma03g36520* | *GmPAP* | *At3g58490* | Phosphatidate Phosphatase | Systematic analysis of protein subcellular localization and interaction using high-throughput transient transformation of Arabidopsis seedlings |
| QE29 |  | RC | 4 | 10 | Marker2338546, Marker2240517 | 3.38 | 0.01 | 3.37 | 2.63 | 0.01 | 2.62 | 0.00 | -0.05 | -0.05 | 0.10 | 3.5 | 12.5 |  | *Glyma04g41220* | *GmACBP1* | *At5g53470* | Acyl CoA Binding Protein | Depletion of the Membrane-Associated Acyl-Coenzyme A-Binding Protein ACBP1 Enhances the Ability of Cold Acclimation in Arabidopsis |
| QE30 |  | OC | 2 | 146 | Marker1235765, Marker1252272 | 5.03 | 2.36 | 2.68 | 3.59 | 2.24 | 1.35 | -0.08 | -0.07 | 0.07 | 0.00 | 145.5 | 146.5 |  | *Glyma02g14211* | *GmDAG-CPT (AAPT)* | *At1g13560* | s | Characterization of aminoalcoholphosphotransferases from Arabidopsis thaliana and soybean |
| QE31 | Stearic acid | OC | 2 | 32 | Marker1287683, Marker1157322 | 4.75 | 0.80 | 3.95 | 1.10 | 0.95 | 0.15 | -0.07 | 0.01 | 0.02 | -0.04 | 30.5 | 35.5 |  | *Glyma02g40280* | *GmHSI2/VAL1* | *At2g30470* | a member of a novel family of B3 domain proteins |  |
| QE32 |  | OC | 3 | 49 | Marker2445658, Marker2404918 | 4.77 | 0.97 | 3.80 | 1.92 | 1.17 | 0.75 | -0.08 | 0.08 | -0.03 | -0.06 | 48.5 | 49.5 |  | *Glyma03g14180* | *GmGPAT6* | *At2g38110* | Glycerol-3-Phosphate Acyltransferase | Nanoridges that characterize the surface morphology of flowers require the synthesis of cutin polyester |
| QE33 |  | OC | 8 | 101 | Marker673687, Marker674654 | 4.51 | 1.11 | 3.39 | 2.04 | 1.34 | 0.70 | -0.08 | 0.08 | -0.02 | -0.06 | 100.5 | 101 |  | *Glyma08g47240* | *GmABI3* | *At3g24650* | Homologous to the maize transcription factor Viviparous-1 | ISOLATION OF THE ARABIDOPSIS-ABI3 GENE BY POSITIONAL CLONING |
| QE34 |  | OC | 13 | 35 | Marker2864523, Marker2845029 | 7.23 | 0.10 | 7.12 | 0.44 | 0.13 | 0.31 | 0.03 | 0.06 | -0.03 | -0.03 | 34.5 | 35.5 |  | *Glyma13g08000* | *GmGER-ABC* | *At1g17840* | ABC Transporter | The Arabidopsis DESPERADO/AtWBC11 transporter is required for cutin and wax secretion |
| QE35 |  | OC | 13 | 59 | Marker2798086, Marker2790748 | 4.87 | 0.23 | 4.64 | 0.78 | 0.27 | 0.51 | 0.04 | -0.03 | 0.07 | -0.04 | 57.5 | 62.5 |  | *Glyma13g16560* | *GmDGAT1* | *At2g19450* | Acyl-CoA : Diacylglycerol Acyltransferase | The TAG1 locus of Arabidopsis encodes for a diacylglycerol acyltransferase |
| QE36 |  | OC | 19 | 30 | Marker1539768, Marker1575567 | 3.97 | 0.06 | 3.91 | 0.28 | 0.08 | 0.21 | 0.02 | 0.04 | 0.01 | -0.04 | 26.5 | 30.5 |  | *Glyma19g32420* | *GmLPAAT2* | *At3g57650* | 1-Acylglycerol-3-Phosphate Acyltransferase | Ubiquitous and endoplasmic reticulum-located lysophosphatidyl acyltransferase, LPAT2, is essential for female but not male gametophyte development in Arabidopsis |

^a^ Population used in QTL mapping. All: all the recombinant inbred lines; RC: recombinant inbred lines from reciprocal cross; OC: recombinant inbred lines from orthogonal cross.

**Table S10.** miRNAs and their targeted acyl-lipid genes, predicted by *psRNAtarget*, *Target Finder*, and *psRobot*, around QTLs for seed oil-related traits in soybean

**Table S10.1.** miRNAs and their targeted acyl-lipid genes, predicted by *psRNAtarget*, around quantitative trait loci for seed oil-related traits in soybean

| **miRNA** | **Gene name 1.1** | **Expectation** | **UPE** | **miRNA aligned fragment** | **Target aligned fragment** | **Inhibition** |
| --- | --- | --- | --- | --- | --- | --- |
| miR156t | Glyma03g29901.1 | 5 | -1 | UUGACAGAAGAAAGGGAGCAC | UUGUUUUUGUUUUUUUGUCAG | Cleavage |
| miR1516b | Glyma05g33790.1 | 4 | -1 | AGCUUCUCUACAGAAAAUAUA | UAUAUAUUCUUUAGAGAAUCU | Translation |
| miR1516b | Glyma05g33790.2 | 4 | -1 | AGCUUCUCUACAGAAAAUAUA | UAUAUAUUCUUUAGAGAAUCU | Translation |
| miR166s | Glyma05g33790.1 | 3.5 | -1 | UCGGACCAGGCUUCAUUCCC | GGGACUAAAGCCUGGUCAGA | Cleavage |
| miR166s | Glyma05g33790.2 | 3.5 | -1 | UCGGACCAGGCUUCAUUCCC | GGGACUAAAGCCUGGUCAGA | Cleavage |
| miR166f | Glyma05g33790.1 | 3.5 | -1 | UCGGACCAGGCUUCAUUCCCC | UGGGACUAAAGCCUGGUCAGA | Cleavage |
| miR166f | Glyma05g33790.2 | 3.5 | -1 | UCGGACCAGGCUUCAUUCCCC | UGGGACUAAAGCCUGGUCAGA | Cleavage |
| miR166i | Glyma05g33790.1 | 3.5 | -1 | UCGGACCAGGCUUCAUUCCCC | UGGGACUAAAGCCUGGUCAGA | Cleavage |
| miR166i | Glyma05g33790.2 | 3.5 | -1 | UCGGACCAGGCUUCAUUCCCC | UGGGACUAAAGCCUGGUCAGA | Cleavage |
| miR167b | Glyma02g40650.1 | 3.5 | -1 | UGAAGCUGCCAGCAUGAUCUA | UAGAUCAGGCUGGCAGCUUGU | Cleavage |
| miR167b | Glyma02g40650.4 | 3.5 | -1 | UGAAGCUGCCAGCAUGAUCUA | UAGAUCAGGCUGGCAGCUUGU | Cleavage |
| miR167b | Glyma02g40650.3 | 3.5 | -1 | UGAAGCUGCCAGCAUGAUCUA | UAGAUCAGGCUGGCAGCUUGU | Cleavage |
| miR167b | Glyma02g40650.2 | 3.5 | -1 | UGAAGCUGCCAGCAUGAUCUA | UAGAUCAGGCUGGCAGCUUGU | Cleavage |
| miR167b | Glyma02g40650.5 | 3.5 | -1 | UGAAGCUGCCAGCAUGAUCUA | UAGAUCAGGCUGGCAGCUUGU | Cleavage |
| miR530d | Glyma14g06290.1 | 3 | -1 | UGCAUUUGCACCUGCACUUUA | UCAUGUGCAUGUGCAAGUGCA | Cleavage |
| miR166k | Glyma08g21610.2 | 3 | -1 | UCUCGGACCAGGCUUCAUUCC | GGAAUGAAGCCUGGUCCGGAU | Cleavage |
| miR166k | Glyma08g21610.1 | 3 | -1 | UCUCGGACCAGGCUUCAUUCC | GGAAUGAAGCCUGGUCCGGAU | Cleavage |
| miR5374 | Glyma02g42960.2 | 3 | -1 | UUAUAGUCUGACAUCUGGAAU | AUUCAAGAUGUCGGACUAGAA | Cleavage |
| miR5374 | Glyma02g42960.1 | 3 | -1 | UUAUAGUCUGACAUCUGGAAU | AUUCAAGAUGUCGGACUAGAA | Cleavage |
| miR319h | Glyma13g29160.2 | 2.5 | -1 | UUGGACUGAAGGGAGCUCCCU | UAGGGGGACCCUUCAGUCCAA | Cleavage |
| miR319h | Glyma13g29160.3 | 2.5 | -1 | UUGGACUGAAGGGAGCUCCCU | UAGGGGGACCCUUCAGUCCAA | Cleavage |
| miR319h | Glyma13g29160.1 | 2.5 | -1 | UUGGACUGAAGGGAGCUCCCU | UAGGGGGACCCUUCAGUCCAA | Cleavage |
| miR319h | Glyma08g10350.1 | 2.5 | -1 | UUGGACUGAAGGGAGCUCCCU | UAGGGGGACCCUUCAGUCCAA | Cleavage |
| miR1516b | Glyma20g29960.1 | 2.5 | -1 | AGCUUCUCUACAGAAAAUAUA | UUUUUUUUCAGUAGAGAAGCU | Cleavage |
| miR159e | Glyma13g25716.2 | 2.5 | -1 | UUUGGAUUGAAGGGAGCUCUA | UGGAGCUCCCUUCACUCCAAU | Cleavage |
| miR159e | Glyma13g25716.1 | 2.5 | -1 | UUUGGAUUGAAGGGAGCUCUA | UGGAGCUCCCUUCACUCCAAU | Cleavage |
| miR171k | Glyma20g36090.3 | 2.5 | -1 | CGAUGUUGGUGAGGUUCAAUC | GUGUGGACCUCACCAACGUUG | Cleavage |
| miR171k | Glyma20g36090.2 | 2.5 | -1 | CGAUGUUGGUGAGGUUCAAUC | GUGUGGACCUCACCAACGUUG | Cleavage |
| miR171k | Glyma20g36090.4 | 2.5 | -1 | CGAUGUUGGUGAGGUUCAAUC | GUGUGGACCUCACCAACGUUG | Cleavage |
| miR171k | Glyma20g36090.1 | 2.5 | -1 | CGAUGUUGGUGAGGUUCAAUC | GUGUGGACCUCACCAACGUUG | Cleavage |
| miR156t | Glyma03g29901.1 | 2 | -1 | UUGACAGAAGAAAGGGAGCAC | GUGCUCUCUCUCUUCUGUCAA | Cleavage |
| miR319h | Glyma13g25716.2 | 1.5 | -1 | UUGGACUGAAGGGAGCUCCCU | UUGGAGCUCCCUUCACUCCAA | Cleavage |
| miR319h | Glyma13g25716.1 | 1.5 | -1 | UUGGACUGAAGGGAGCUCCCU | UUGGAGCUCCCUUCACUCCAA | Cleavage |
| miR156i | Glyma03g29901.1 | 1.5 | -1 | UUGACAGAAGAUAGAGAGCAC | GUGCUCUCUCUCUUCUGUCAA | Cleavage |
| miR156l | Glyma03g29901.1 | 1.5 | -1 | UUGACAGAAGAUAGAGAGCAC | GUGCUCUCUCUCUUCUGUCAA | Cleavage |
| miR530d | Glyma02g01740.1 | 1 | -1 | UGCAUUUGCACCUGCACUUUA | GCAGGUGCAGGUGCAAGUGCA | Cleavage |
| miR156q | Glyma03g29901.1 | 1 | -1 | UGACAGAAGAGAGUGAGCACU | UGUGCUCUCUCUCUUCUGUCA | Cleavage |
| miR166s | Glyma08g21610.2 | 0.5 | -1 | UCGGACCAGGCUUCAUUCCC | UGGAAUGAAGCCUGGUCCGG | Cleavage |
| miR166s | Glyma08g21610.1 | 0.5 | -1 | UCGGACCAGGCUUCAUUCCC | UGGAAUGAAGCCUGGUCCGG | Cleavage |
| miR160b | Glyma10g06080.1 | 0.5 | -1 | UGCCUGGCUCCCUGUAUGCC | GGCAUGCAGGGAGCCAGGCA | Cleavage |
| miR160d | Glyma10g06080.1 | 0.5 | -1 | UGCCUGGCUCCCUGUAUGCC | GGCAUGCAGGGAGCCAGGCA | Cleavage |
| miR166f | Glyma08g21610.2 | 0.5 | -1 | UCGGACCAGGCUUCAUUCCCC | CUGGAAUGAAGCCUGGUCCGG | Cleavage |
| miR166f | Glyma08g21610.1 | 0.5 | -1 | UCGGACCAGGCUUCAUUCCCC | CUGGAAUGAAGCCUGGUCCGG | Cleavage |
| miR166i | Glyma08g21610.2 | 0.5 | -1 | UCGGACCAGGCUUCAUUCCCC | CUGGAAUGAAGCCUGGUCCGG | Cleavage |
| miR166i | Glyma08g21610.1 | 0.5 | -1 | UCGGACCAGGCUUCAUUCCCC | CUGGAAUGAAGCCUGGUCCGG | Cleavage |

**Table S10.2** miRNAs and their targeted acyl-lipid genes, predicted by *Target Finder*, around quantitative trait loci for seed oil-related traits in soybean

| **miRNA** | **Gene name 1.1** | **Score** | **Range** | **miRNA** | **Gene name 1.1** | **Score** | **Range** |
| --- | --- | --- | --- | --- | --- | --- | --- |
| miR319h | Glyma13g29160.1 | 2.5 | range=2286-2305 | miR160b | Glyma10g06080.3 | 0.5 | range=1768-1787 |
| miR166s | Glyma08g21610.1 | 1.5 | range=1003-1022 | miR160b | Glyma10g06080.2 | 0.5 | range=2000-2019 |
| miR166s | Glyma05g33790.2 | 4 | range=977-996 | miR160d | Glyma10g06080.3 | 0.5 | range=1768-1787 |
| miR166s | Glyma05g33790.1 | 4 | range=977-996 | miR160d | Glyma10g06080.2 | 0.5 | range=2000-2019 |
| miR319h | Glyma08g10350.1 | 2.5 | range=2120-2139 | miR166f | Glyma08g21610.1 | 2.5 | range=1002-1022 |
| miR319h | Glyma13g25716.1 | 4 | range=1328-1348 | miR166i | Glyma08g21610.1 | 2.5 | range=1002-1022 |
| miR319h | Glyma13g25716.2 | 4 | range=1818-1838 | miR166k | Glyma08g21610.1 | 4 | range=1004-1024 |
| miR530d | Glyma02g01740.1 | 3 | range=616-637 | miR167b | Glyma02g40650.1 | 4 | range=2931-2952 |
| miR1516b | Glyma20g29960.2 | 4 | range=3618-3638 | miR167b | Glyma02g40650.4 | 4 | range=3006-3027 |
| miR156i | Glyma03g29901.1 | 2 | range=1138-1158 | miR167b | Glyma02g40650.3 | 4 | range=3033-3054 |
| miR156l | Glyma03g29901.1 | 2 | range=1138-1158 | miR167b | Glyma02g40650.2 | 4 | range=2931-2952 |
| miR156q | Glyma03g29901.1 | 2 | range=1137-1157 | miR171k | Glyma20g36090.1 | 4 | range=662-683 |
| miR156t | Glyma03g29901.1 | 2.5 | range=1138-1158 | miR5374 | Glyma02g42960.1 | 4 | range=887-907 |
| miR159e | Glyma13g25716.1 | 3.5 | range=1329-1349 | miR160b | Glyma10g06080.3 | 0.5 | range=1768-1787 |
| miR159e | Glyma13g25716.2 | 3.5 | range=1819-1839 |  |  |  |  |

**Table S10.3** miRNAs and their targeted acyl-lipid genes, predicted by *psRobot*, around quantitative trait loci for seed oil-related traits in soybean

| **miRNA** | **Gene name 1.1** | **Score** | **miRNA primary transcript** | **miRNA** | **Gene name 1.1** | **Score** | **miRNA primary transcript** |
| --- | --- | --- | --- | --- | --- | --- | --- |
| miR1516b | Glyma20g29960.2 | Score: 2.0 | MIMAT0021061 | miR166f | Glyma08g21610.1 | Score: 1.2 | MIMAT0020981 |
| miR156i | Glyma03g29901.1 | Score: 1.0 | MIMAT0020969 | miR166i | Glyma08g21610.1 | Score: 1.2 | MIMAT0021646 |
| miR156l | Glyma03g29901.1 | Score: 1.0 | MIMAT0021636 | miR166k | Glyma08g21610.1 | Score: 2.0 | MIMAT0023223 |
| miR156q | Glyma03g29901.1 | Score: 1.5 | MIMAT0023203 | miR166s | Glyma08g21610.1 | Score: 0.8 | MIMAT0024901 |
| miR156t | Glyma03g29901.1 | Score: 1.5 | MIMAT0023225 | miR167b | Glyma02g40650.2 | Score: 2.5 | MIMAT0001680 |
| miR159e | Glyma13g25716.2 | Score: 1.8 | MIMAT0021641 | miR167b | Glyma02g40650.3 | Score: 2.5 | MIMAT0001680 |
| miR159e | Glyma13g25716.1 | Score: 1.8 | MIMAT0021641 | miR167b | Glyma02g40650.4 | Score: 2.5 | MIMAT0001680 |
| miR160b | Glyma10g06080.3 | Score: 0.5 | MIMAT0020970 | miR167b | Glyma02g40650.1 | Score: 2.5 | MIMAT0001680 |
| miR160b | Glyma10g06080.2 | Score: 0.5 | MIMAT0020970 | miR171k | Glyma20g36090.1 | Score: 2.2 | MIMAT0021655 |
| miR160d | Glyma10g06080.3 | Score: 0.5 | MIMAT0020972 | miR530d | Glyma14g06290.1 | Score: 2.5 | MIMAT0023199 |
| miR160d | Glyma10g06080.2 | Score: 0.5 | MIMAT0020972 | miR530d | Glyma02g01740.1 | Score: 1.5 | MIMAT0023199 |

**Table S11.** Co-expression Pearson correlation coefficients among all the candidate genes in GRN

| **Gene 1** | **Gene 2** | **Correlation coefficient** | **P-value** | **Gene 1** | **Gene 2** | **Correlation coefficient** | **P-value** |
| --- | --- | --- | --- | --- | --- | --- | --- |
| *Glyma02g01740* | *Glyma05g33790* | 0.8500 | 1.47E-07 | *Glyma10g06080* | *Glyma05g33790* | 0.8272 | 6.20E-07 |
| *Glyma02g01740* | *Glyma15g01350* | 0.8366 | 3.52E-07 | *Glyma10g06080* | *Glyma18g04540* | 0.8237 | 7.61E-07 |
| *Glyma02g01740* | *Glyma07g08950* | 0.8267 | 6.38E-07 | *Glyma10g06080* | *Glyma09g41040* | 0.8163 | 1.15E-06 |
| *Glyma02g40650* | *Glyma10g05320* | 0.9338 | 2.70E-11 | *Glyma10g06080* | *Glyma20g00810* | 0.8159 | 1.17E-06 |
| *Glyma02g40650* | *Glyma20g00810* | 0.8916 | 5.05E-09 | *Glyma10g06080* | *Glyma12g07570* | 0.8155 | 1.21E-06 |
| *Glyma02g40650* | *Glyma08g16620* | 0.8634 | 5.60E-08 | *Glyma10g06080* | *Glyma07g08810* | 0.8107 | 1.56E-06 |
| *Glyma02g40650* | *Glyma13g44170* | 0.8555 | 9.96E-08 | *Glyma10g06080* | *Glyma02g08350* | 0.8102 | 1.60E-06 |
| *Glyma02g42960* | *Glyma20g31480* | 0.9598 | 1.27E-13 | *Glyma10g06080* | *Glyma17g00950* | 0.8089 | 1.71E-06 |
| *Glyma02g42960* | *Glyma01g44600* | 0.9584 | 1.83E-13 | *Glyma10g06080* | *Glyma17g08510* | 0.8041 | 2.20E-06 |
| *Glyma02g42960* | *Glyma02g41660* | 0.9109 | 6.37E-10 | *Glyma10g06080* | *Glyma12g04990* | 0.8026 | 2.38E-06 |
| *Glyma02g42960* | *Glyma10g01770* | 0.8456 | 1.97E-07 | *Glyma11g18401* | *Glyma13g23150* | 0.8550 | 1.03E-07 |
| *Glyma02g42960* | *Glyma17g36130* | 0.8295 | 5.42E-07 | *Glyma12g04440* | *Glyma15g18770* | 0.8117 | 1.48E-06 |
| *Glyma02g42960* | *Glyma18g00381* | 0.8247 | 7.20E-07 | *Glyma12g04440* | *Glyma07g08810* | 0.8056 | 2.03E-06 |
| *Glyma02g42960* | *Glyma18g02900* | 0.8158 | 1.18E-06 | *Glyma13g25716* | *Glyma18g11683* | 0.8701 | 3.31E-08 |
| *Glyma02g42960* | *Glyma05g04940* | 0.8134 | 1.35E-06 | *Glyma13g25716* | *Glyma06g07360* | 0.8323 | 4.59E-07 |
| *Glyma02g42960* | *Glyma06g12010* | 0.8047 | 2.13E-06 | *Glyma13g25716* | *Glyma17g15320* | 0.8277 | 6.03E-07 |
| *Glyma03g29901* | *Glyma07g07580* | 0.9218 | 1.61E-10 | *Glyma13g25716* | *Glyma06g07230* | 0.8252 | 6.97E-07 |
| *Glyma03g29901* | *Glyma13g22230* | 0.8092 | 1.68E-06 | *Glyma13g25716* | *Glyma05g08060* | 0.8236 | 7.66E-07 |
| *Glyma05g28140* | *Glyma10g02040* | 0.8915 | 5.10E-09 | *Glyma13g25716* | *Glyma20g31200* | 0.8062 | 1.98E-06 |
| *Glyma05g28140* | *Glyma07g07580* | 0.8805 | 1.39E-08 | *Glyma13g25716* | *Glyma18g52590* | 0.8029 | 2.34E-06 |
| *Glyma05g28140* | *Glyma03g42140* | 0.8800 | 1.46E-08 | *Glyma13g29160* | *Glyma13g25290* | 0.9391 | 1.11E-11 |
| *Glyma05g28140* | *Glyma05g04330* | 0.8759 | 2.06E-08 | *Glyma13g29160* | *Glyma17g00950* | 0.9337 | 2.74E-11 |
| *Glyma05g28140* | *Glyma17g13280* | 0.8657 | 4.68E-08 | *Glyma13g29160* | *Glyma06g07360* | 0.9070 | 1.00E-09 |
| *Glyma05g28140* | *Glyma18g52590* | 0.8510 | 1.36E-07 | *Glyma13g29160* | *Glyma02g46550* | 0.9030 | 1.57E-09 |
| *Glyma05g28140* | *Glyma07g39890* | 0.8430 | 2.33E-07 | *Glyma13g29160* | *Glyma14g04780* | 0.8845 | 9.73E-09 |
| *Glyma05g28140* | *Glyma13g25290* | 0.8335 | 4.25E-07 | *Glyma13g29160* | *Glyma07g07580* | 0.8767 | 1.92E-08 |
| *Glyma05g28140* | *Glyma03g42190* | 0.8272 | 6.22E-07 | *Glyma13g29160* | *Glyma11g37320* | 0.8347 | 3.94E-07 |
| *Glyma05g28140* | *Glyma07g07560* | 0.8255 | 6.84E-07 | *Glyma17g03410* | *Glyma02g46550* | 0.9229 | 1.38E-10 |
| *Glyma05g28140* | *Glyma20g01680* | 0.8216 | 8.58E-07 | *Glyma17g03410* | *Glyma06g07360* | 0.8782 | 1.70E-08 |
| *Glyma05g28140* | *Glyma02g46550* | 0.8213 | 8.72E-07 | *Glyma17g03410* | *Glyma13g25290* | 0.8482 | 1.65E-07 |
| *Glyma05g28140* | *Glyma11g37320* | 0.8101 | 1.61E-06 | *Glyma17g03410* | *Glyma13g23150* | 0.8435 | 2.26E-07 |
| *Glyma05g28140* | *Glyma06g07360* | 0.8085 | 1.75E-06 | *Glyma17g03410* | *Glyma17g00950* | 0.8236 | 7.63E-07 |
| *Glyma05g28140* | *Glyma16g00790* | 0.8059 | 2.00E-06 | *Glyma17g03410* | *Glyma04g43070* | 0.8113 | 1.51E-06 |
| *Glyma05g28140* | *Glyma07g03350* | 0.8038 | 2.23E-06 | *Glyma17g03410* | *Glyma07g07560* | 0.8104 | 1.58E-06 |
| *Glyma08g10350* | *Glyma07g07580* | 0.9458 | 3.20E-12 | *Glyma17g03410* | *Glyma11g37320* | 0.8071 | 1.89E-06 |
| *Glyma08g10350* | *Glyma06g07360* | 0.9178 | 2.72E-10 | *Glyma17g03410* | *Glyma13g20100* | 0.8067 | 1.93E-06 |
| *Glyma08g10350* | *Glyma20g31630* | 0.9130 | 4.96E-10 | *Glyma17g03410* | *Glyma07g07580* | 0.8063 | 1.96E-06 |
| *Glyma08g10350* | *Glyma02g46550* | 0.8865 | 8.16E-09 | *Glyma17g03410* | *Glyma03g42190* | 0.8057 | 2.03E-06 |
| *Glyma08g10350* | *Glyma16g00790* | 0.8863 | 8.31E-09 | *Glyma20g29960* | *Glyma15g16270* | 0.9285 | 6.13E-11 |
| *Glyma08g10350* | *Glyma18g52590* | 0.8822 | 1.20E-08 | *Glyma20g29960* | *Glyma09g41040* | 0.9152 | 3.78E-10 |
| *Glyma08g10350* | *Glyma18g02210* | 0.8420 | 2.49E-07 | *Glyma20g29960* | *Glyma06g15820* | 0.9098 | 7.26E-10 |
| *Glyma08g10350* | *Glyma16g02090* | 0.8283 | 5.81E-07 | *Glyma20g29960* | *Glyma07g08810* | 0.8848 | 9.52E-09 |
| *Glyma08g10350* | *Glyma14g04780* | 0.8183 | 1.03E-06 | *Glyma20g29960* | *Glyma17g08510* | 0.8627 | 5.90E-08 |
| *Glyma08g21610* | *Glyma20g01680* | 0.9405 | 8.69E-12 | *Glyma20g29960* | *Glyma12g00320* | 0.8554 | 1.01E-07 |
| *Glyma08g21610* | *Glyma13g23150* | 0.9001 | 2.14E-09 | *Glyma20g29960* | *Glyma05g09400* | 0.8521 | 1.26E-07 |
| *Glyma08g21610* | *Glyma15g16270* | 0.8998 | 2.20E-09 | *Glyma20g29960* | *Glyma20g00810* | 0.8509 | 1.38E-07 |
| *Glyma08g21610* | *Glyma18g02110* | 0.8965 | 3.10E-09 | *Glyma20g29960* | *Glyma16g26210* | 0.8492 | 1.55E-07 |
| *Glyma08g21610* | *Glyma17g13280* | 0.8772 | 1.85E-08 | *Glyma20g29960* | *Glyma12g04990* | 0.8346 | 3.99E-07 |
| *Glyma08g21610* | *Glyma12g07570* | 0.8746 | 2.31E-08 | *Glyma20g29960* | *Glyma14g00760* | 0.8108 | 1.55E-06 |
| *Glyma08g21610* | *Glyma05g09400* | 0.8703 | 3.28E-08 | *Glyma16g02090* | *Glyma20g31630* | 0.9602 | 1.14E-13 |
| *Glyma08g21610* | *Glyma13g44170* | 0.8619 | 6.25E-08 | *Glyma10g02040* | *Glyma20g31630* | 0.9503 | 1.25E-12 |
| *Glyma08g21610* | *Glyma14g04780* | 0.8588 | 7.84E-08 | *Glyma05g34900* | *Glyma10g34150* | 0.9455 | 3.39E-12 |
| *Glyma08g21610* | *Glyma06g15820* | 0.8588 | 7.88E-08 | *Glyma06g15820* | *Glyma08g16620* | 0.9403 | 8.94E-12 |
| *Glyma08g21610* | *Glyma20g31200* | 0.8583 | 8.15E-08 | *Glyma08g19360* | *Glyma15g05630* | 0.9307 | 4.43E-11 |
| *Glyma08g21610* | *Glyma18g11683* | 0.8566 | 9.19E-08 | *Glyma10g02040* | *Glyma16g02090* | 0.9274 | 7.30E-11 |
| *Glyma08g21610* | *Glyma20g25816* | 0.8558 | 9.74E-08 | *Glyma05g09400* | *Glyma20g31200* | 0.9262 | 8.67E-11 |
| *Glyma08g21610* | *Glyma14g00760* | 0.8519 | 1.29E-07 | *Glyma17g12940* | *Glyma08g45990* | 0.9237 | 1.23E-10 |
| *Glyma08g21610* | *Glyma20g31630* | 0.8516 | 1.31E-07 | *Glyma17g12940* | *Glyma06g11860* | 0.9224 | 1.47E-10 |
| *Glyma08g21610* | *Glyma17g12940* | 0.8490 | 1.57E-07 | *Glyma08g45990* | *Glyma14g04780* | 0.9209 | 1.80E-10 |
| *Glyma08g21610* | *Glyma06g07230* | 0.8466 | 1.85E-07 | *Glyma05g09400* | *Glyma16g26210* | 0.9193 | 2.24E-10 |
| *Glyma08g21610* | *Glyma12g04990* | 0.8446 | 2.11E-07 | *Glyma13g44170* | *Glyma15g16270* | 0.9191 | 2.28E-10 |
| *Glyma08g21610* | *Glyma15g18770* | 0.8374 | 3.34E-07 | *Glyma08g16620* | *Glyma15g16270* | 0.9163 | 3.27E-10 |
| *Glyma08g21610* | *Glyma10g40910* | 0.8344 | 4.02E-07 | *Glyma02g14211* | *Glyma08g45990* | 0.9130 | 4.94E-10 |
| *Glyma08g21610* | *Glyma12g02300* | 0.8263 | 6.53E-07 | *Glyma08g45990* | *Glyma13g28590* | 0.9107 | 6.53E-10 |
| *Glyma08g21610* | *Glyma12g05140* | 0.8261 | 6.60E-07 | *Glyma03g42190* | *Glyma10g02040* | 0.9100 | 7.09E-10 |
| *Glyma08g21610* | *Glyma13g22230* | 0.8251 | 7.01E-07 | *Glyma06g07230* | *Glyma13g23150* | 0.9089 | 8.10E-10 |
| *Glyma08g21610* | *Glyma07g38910* | 0.8232 | 7.80E-07 | *Glyma12g07570* | *Glyma14g00760* | 0.9070 | 9.99E-10 |
| *Glyma08g21610* | *Glyma07g08810* | 0.8132 | 1.37E-06 | *Glyma05g09400* | *Glyma13g44170* | 0.9062 | 1.10E-09 |
| *Glyma08g21610* | *Glyma12g01500* | 0.8118 | 1.47E-06 | *Glyma03g42190* | *Glyma20g31630* | 0.9038 | 1.44E-09 |
| *Glyma08g21610* | *Glyma05g08060* | 0.8114 | 1.50E-06 | *Glyma11g37320* | *Glyma13g28590* | 0.9011 | 1.92E-09 |
| *Glyma08g21610* | *Glyma06g07360* | 0.8099 | 1.63E-06 | *Glyma03g40250* | *Glyma14g04780* | 0.8954 | 3.47E-09 |
| *Glyma08g21610* | *Glyma12g00320* | 0.8092 | 1.69E-06 | *Glyma05g26141* | *Glyma13g28590* | 0.8950 | 3.59E-09 |
| *Glyma08g21610* | *Glyma05g34900* | 0.8054 | 2.05E-06 | *Glyma17g12940* | *Glyma14g04780* | 0.8937 | 4.08E-09 |
| *Glyma08g21610* | *Glyma15g01350* | 0.8049 | 2.11E-06 | *Glyma13g23150* | *Glyma16g00790* | 0.8920 | 4.85E-09 |
| *Glyma08g21610* | *Glyma13g41520* | 0.8021 | 2.44E-06 | *Glyma02g14211* | *Glyma17g37720* | 0.8905 | 5.57E-09 |
| *Glyma09g14380* | *Glyma13g25290* | 0.9402 | 9.05E-12 | *Glyma08g45990* | *Glyma11g37320* | 0.8904 | 5.66E-09 |
| *Glyma09g14380* | *Glyma06g07360* | 0.9079 | 9.10E-10 | *Glyma01g43470* | *Glyma05g31270* | 0.8894 | 6.22E-09 |
| *Glyma09g14380* | *Glyma02g46550* | 0.9060 | 1.12E-09 | *Glyma14g00760* | *Glyma20g31630* | 0.8875 | 7.40E-09 |
| *Glyma09g14380* | *Glyma07g03350* | 0.8988 | 2.44E-09 | *Glyma06g02250* | *Glyma13g11700* | 0.8864 | 8.24E-09 |
| *Glyma09g14380* | *Glyma03g42140* | 0.8926 | 4.58E-09 | *Glyma11g03800* | *Glyma01g43470* | 0.8860 | 8.50E-09 |
| *Glyma09g14380* | *Glyma04g43070* | 0.8590 | 7.77E-08 | *Glyma15g16270* | *Glyma17g08510* | 0.8857 | 8.74E-09 |
| *Glyma09g14380* | *Glyma16g02090* | 0.8307 | 5.05E-07 | *Glyma08g16620* | *Glyma20g01680* | 0.8849 | 9.39E-09 |
| *Glyma09g29810* | *Glyma17g00950* | 0.9624 | 6.21E-14 | *Glyma05g33790* | *Glyma14g00760* | 0.8844 | 9.89E-09 |
| *Glyma09g29810* | *Glyma06g11860* | 0.9370 | 1.60E-11 | *Glyma06g15820* | *Glyma15g16270* | 0.8817 | 1.25E-08 |
| *Glyma09g29810* | *Glyma08g45990* | 0.9291 | 5.68E-11 | *Glyma07g07580* | *Glyma16g00790* | 0.8795 | 1.52E-08 |
| *Glyma09g29810* | *Glyma16g02090* | 0.9180 | 2.66E-10 | *Glyma07g38910* | *Glyma13g23150* | 0.8784 | 1.68E-08 |
| *Glyma09g29810* | *Glyma02g14211* | 0.9092 | 7.78E-10 | *Glyma13g30920* | *Glyma17g37720* | 0.8780 | 1.72E-08 |
| *Glyma09g29810* | *Glyma17g12940* | 0.8912 | 5.23E-09 | *Glyma11g03800* | *Glyma14g00760* | 0.8780 | 1.73E-08 |
| *Glyma09g29810* | *Glyma13g25290* | 0.8904 | 5.65E-09 | *Glyma13g28590* | *Glyma14g04780* | 0.8766 | 1.95E-08 |
| *Glyma09g29810* | *Glyma07g04080* | 0.8708 | 3.13E-08 | *Glyma16g26210* | *Glyma20g31200* | 0.8761 | 2.04E-08 |
| *Glyma09g29810* | *Glyma20g31200* | 0.8673 | 4.13E-08 | *Glyma12g05140* | *Glyma14g04780* | 0.8755 | 2.14E-08 |
| *Glyma09g29810* | *Glyma18g02210* | 0.8590 | 7.74E-08 | *Glyma03g40250* | *Glyma10g34150* | 0.8754 | 2.16E-08 |
| *Glyma09g29810* | *Glyma14g04780* | 0.8552 | 1.02E-07 | *Glyma12g08720* | *Glyma13g19730* | 0.8752 | 2.19E-08 |
| *Glyma09g29810* | *Glyma05g04330* | 0.8518 | 1.29E-07 | *Glyma10g32520* | *Glyma18g02110* | 0.8746 | 2.30E-08 |
| *Glyma09g29810* | *Glyma11g37320* | 0.8480 | 1.67E-07 | *Glyma12g08720* | *Glyma08g45990* | 0.8746 | 2.31E-08 |
| *Glyma09g29810* | *Glyma07g39890* | 0.8454 | 2.00E-07 | *Glyma12g08720* | *Glyma17g37720* | 0.8727 | 2.69E-08 |
| *Glyma09g29810* | *Glyma12g08720* | 0.8445 | 2.12E-07 | *Glyma12g00320* | *Glyma20g01680* | 0.8717 | 2.92E-08 |
| *Glyma09g29810* | *Glyma03g42190* | 0.8395 | 2.92E-07 | *Glyma12g08720* | *Glyma05g33790* | 0.8711 | 3.06E-08 |
| *Glyma09g29810* | *Glyma14g00760* | 0.8297 | 5.36E-07 | *Glyma07g37570* | *Glyma20g28200* | 0.8711 | 3.07E-08 |
| *Glyma09g29810* | *Glyma12g04990* | 0.8264 | 6.49E-07 | *Glyma14g09380* | *Glyma16g26210* | 0.8694 | 3.52E-08 |
| *Glyma09g29810* | *Glyma17g37720* | 0.8173 | 1.09E-06 | *Glyma14g00760* | *Glyma15g01540* | 0.8636 | 5.48E-08 |
| *Glyma09g29810* | *Glyma07g07580* | 0.8131 | 1.37E-06 | *Glyma08g46360* | *Glyma13g28590* | 0.8633 | 5.63E-08 |
| *Glyma09g29810* | *Glyma18g52590* | 0.8080 | 1.80E-06 | *Glyma07g17180* | *Glyma10g34150* | 0.8618 | 6.30E-08 |
| *Glyma09g40130* | *Glyma02g11180* | 0.8635 | 5.56E-08 | *Glyma11g03800* | *Glyma12g07570* | 0.8617 | 6.37E-08 |
| *Glyma09g40130* | *Glyma17g12940* | 0.8564 | 9.34E-08 | *Glyma13g44170* | *Glyma17g08510* | 0.8615 | 6.46E-08 |
| *Glyma09g40130* | *Glyma13g22230* | 0.8558 | 9.76E-08 | *Glyma11g03800* | *Glyma06g11860* | 0.8613 | 6.53E-08 |
| *Glyma09g40130* | *Glyma12g04990* | 0.8517 | 1.30E-07 | *Glyma14g00760* | *Glyma18g02110* | 0.8608 | 6.79E-08 |
| *Glyma09g40130* | *Glyma17g13280* | 0.8419 | 2.50E-07 | *Glyma05g34900* | *Glyma10g02040* | 0.8589 | 7.79E-08 |
| *Glyma09g40130* | *Glyma06g07360* | 0.8333 | 4.31E-07 | *Glyma07g08810* | *Glyma13g44170* | 0.8576 | 8.59E-08 |
| *Glyma09g40130* | *Glyma13g19730* | 0.8264 | 6.52E-07 | *Glyma05g31270* | *Glyma18g02110* | 0.8556 | 9.92E-08 |
| *Glyma09g40130* | *Glyma20g02980* | 0.8221 | 8.32E-07 | *Glyma07g08810* | *Glyma15g16270* | 0.8554 | 1.00E-07 |
| *Glyma09g40130* | *Glyma07g07580* | 0.8154 | 1.21E-06 | *Glyma05g09400* | *Glyma14g09380* | 0.8550 | 1.04E-07 |
| *Glyma09g40130* | *Glyma13g25290* | 0.8135 | 1.34E-06 | *Glyma06g11860* | *Glyma13g25290* | 0.8514 | 1.33E-07 |
| *Glyma09g40130* | *Glyma16g26210* | 0.8105 | 1.57E-06 | *Glyma13g19730* | *Glyma13g44170* | 0.8504 | 1.42E-07 |
| *Glyma09g40130* | *Glyma07g17110* | 0.8105 | 1.58E-06 | *Glyma14g36850* | *Glyma20g02980* | 0.8496 | 1.50E-07 |
| *Glyma09g40130* | *Glyma10g05320* | 0.8095 | 1.66E-06 | *Glyma05g08060* | *Glyma14g04780* | 0.8494 | 1.53E-07 |
| *Glyma09g40130* | *Glyma20g25816* | 0.8080 | 1.80E-06 | *Glyma10g34150* | *Glyma13g28590* | 0.8488 | 1.59E-07 |
| *Glyma09g40130* | *Glyma07g04080* | 0.8047 | 2.14E-06 | *Glyma03g40250* | *Glyma08g46360* | 0.8481 | 1.67E-07 |
| *Glyma09g40130* | *Glyma06g07230* | 0.8014 | 2.52E-06 | *Glyma05g09400* | *Glyma15g16270* | 0.8476 | 1.72E-07 |
| *Glyma09g40130* | *Glyma02g46550* | 0.8008 | 2.60E-06 | *Glyma12g08720* | *Glyma16g26210* | 0.8471 | 1.79E-07 |
| *Glyma09g40130* | *Glyma20g01680* | 0.8001 | 2.69E-06 | *Glyma12g08720* | *Glyma13g28590* | 0.8459 | 1.93E-07 |
| *Glyma10g06080* | *Glyma17g15320* | 0.9290 | 5.70E-11 | *Glyma01g43470* | *Glyma18g02110* | 0.8447 | 2.09E-07 |
| *Glyma10g06080* | *Glyma20g01680* | 0.9170 | 3.01E-10 | *Glyma05g04940* | *Glyma20g28200* | 0.8395 | 2.93E-07 |
| *Glyma10g06080* | *Glyma15g16270* | 0.9074 | 9.54E-10 | *Glyma17g15320* | *Glyma18g02110* | 0.8365 | 3.53E-07 |
| *Glyma10g06080* | *Glyma14g04780* | 0.9014 | 1.85E-09 | *Glyma12g08720* | *Glyma07g38910* | 0.8332 | 4.32E-07 |
| *Glyma10g06080* | *Glyma20g31200* | 0.8929 | 4.42E-09 | *Glyma05g31270* | *Glyma10g39540* | 0.8297 | 5.35E-07 |
| *Glyma10g06080* | *Glyma03g40250* | 0.8903 | 5.68E-09 | *Glyma04g04060* | *Glyma09g38570* | 0.8284 | 5.79E-07 |
| *Glyma10g06080* | *Glyma15g18770* | 0.8894 | 6.19E-09 | *Glyma12g05140* | *Glyma17g15320* | 0.8280 | 5.91E-07 |
| *Glyma10g06080* | *Glyma08g16620* | 0.8854 | 8.97E-09 | *Glyma06g11860* | *Glyma13g28590* | 0.8264 | 6.49E-07 |
| *Glyma10g06080* | *Glyma16g02090* | 0.8793 | 1.54E-08 | *Glyma14g09380* | *Glyma20g31200* | 0.8262 | 6.58E-07 |
| *Glyma10g06080* | *Glyma05g09400* | 0.8758 | 2.08E-08 | *Glyma03g40250* | *Glyma11g37320* | 0.8249 | 7.08E-07 |
| *Glyma10g06080* | *Glyma05g04330* | 0.8716 | 2.93E-08 | *Glyma09g32750* | *Glyma14g08400* | 0.8234 | 7.72E-07 |
| *Glyma10g06080* | *Glyma07g38910* | 0.8691 | 3.60E-08 | *Glyma10g39540* | *Glyma18g02110* | 0.8234 | 7.73E-07 |
| *Glyma10g06080* | *Glyma06g22065* | 0.8636 | 5.50E-08 | *Glyma06g15820* | *Glyma20g01680* | 0.8221 | 8.34E-07 |
| *Glyma10g06080* | *Glyma13g22230* | 0.8601 | 7.12E-08 | *Glyma14g00760* | *Glyma17g15320* | 0.8133 | 1.36E-06 |
| *Glyma10g06080* | *Glyma17g12940* | 0.8590 | 7.77E-08 | *Glyma05g04940* | *Glyma04g42740* | 0.8129 | 1.39E-06 |
| *Glyma10g06080* | *Glyma10g32520* | 0.8497 | 1.50E-07 | *Glyma11g03800* | *Glyma12g05140* | 0.8119 | 1.47E-06 |
| *Glyma10g06080* | *Glyma18g02110* | 0.8490 | 1.57E-07 | *Glyma18g11683* | *Glyma20g31630* | 0.8116 | 1.49E-06 |
| *Glyma10g06080* | *Glyma03g42190* | 0.8453 | 2.00E-07 | *Glyma05g33790* | *Glyma15g01540* | 0.8113 | 1.51E-06 |
| *Glyma10g06080* | *Glyma12g05140* | 0.8427 | 2.38E-07 | *Glyma12g05140* | *Glyma18g02110* | 0.8090 | 1.70E-06 |
| *Glyma10g06080* | *Glyma12g08720* | 0.8412 | 2.63E-07 | *Glyma12g07570* | *Glyma14g09380* | 0.8088 | 1.72E-06 |
| *Glyma10g06080* | *Glyma13g44170* | 0.8380 | 3.22E-07 | *Glyma17g12940* | *Glyma13g28590* | 0.8086 | 1.74E-06 |
| *Glyma10g06080* | *Glyma15g01350* | 0.8364 | 3.56E-07 | *Glyma03g42140* | *Glyma14g04780* | 0.8075 | 1.85E-06 |
| *Glyma10g06080* | *Glyma15g01540* | 0.8341 | 4.10E-07 | *Glyma01g36011* | *Glyma14g05510* | 0.8066 | 1.93E-06 |
| *Glyma10g06080* | *Glyma08g46360* | 0.8320 | 4.66E-07 | *Glyma07g07580* | *Glyma14g33860* | 0.8065 | 1.94E-06 |
| *Glyma10g06080* | *Glyma13g25290* | 0.8320 | 4.67E-07 | *Glyma13g44170* | *Glyma20g25816* | 0.8054 | 2.06E-06 |
| *Glyma10g06080* | *Glyma08g45990* | 0.8316 | 4.78E-07 | *Glyma11g03800* | *Glyma18g02110* | 0.8036 | 2.25E-06 |
| *Glyma10g06080* | *Glyma06g15820* | 0.8306 | 5.07E-07 | *Glyma05g26141* | *Glyma08g46360* | 0.8015 | 2.51E-06 |
| *Glyma10g06080* | *Glyma10g02040* | 0.8289 | 5.61E-07 | *Glyma05g26141* | *Glyma08g45990* | 0.8015 | 2.52E-06 |
| *Glyma10g06080* | *Glyma03g42140* | 0.8288 | 5.64E-07 | *Glyma05g33790* | *Glyma15g18770* | 0.8015 | 2.52E-06 |
| *Glyma10g06080* | *Glyma13g28590* | 0.8285 | 5.76E-07 | *Glyma05g08060* | *Glyma18g11683* | 0.8007 | 2.61E-06 |
| *Glyma10g06080* | *Glyma17g37720* | 0.8279 | 5.96E-07 | *Glyma03g27360* | *Glyma07g09370* | 0.800258109 | 2.67E-06 |
| *Glyma10g06080* | *Glyma13g23150* | 0.8275 | 6.091E-07 |  |  |  |  |

**Table S12.** Candidate genes for oil-related traits and their promoter sequences matched to motifs of miRNA-targeted TFs predicted by FIMO software

| **Motif ID**  **(Gene name 2.0)** | **Motif ID**  **(Gene name 1.1)** | **Motif alternative ID** | **Sequence gene**  **name 2.0** | **Sequence gene name 1.1** | **Start** | **Stop** | **Strand** | **Score** | **P-value** | **Q-value** | **Matched sequence** |
| --- | --- | --- | --- | --- | --- | --- | --- | --- | --- | --- | --- |
| *Glyma.08g201900* | *Glyma08g21610* | MP00200 | *Glyma.05g000200* | *Glyma05g09400* | 1555 | 1569 | - | 19.0597 | 7.92E-08 | 0.0281 | AATAGTAATGATGAT |
| *Glyma.08g201900* | *Glyma08g21610* | MP00200 | *Glyma.05g000200* | *Glyma05g09400* | 1555 | 1569 | - | 19.0597 | 7.92E-08 | 0.0281 | AATAGTAATGATGAT |
| *Glyma.08g201900* | *Glyma08g21610* | MP00200 | *Glyma.05g000200* | *Glyma05g09400* | 1555 | 1569 | - | 19.0597 | 7.92E-08 | 0.0281 | AATAGTAATGATGAT |
| *Glyma.08g201900* | *Glyma08g21610* | MP00200 | *Glyma.05g000200* | *Glyma05g09400* | 1555 | 1569 | - | 19.0597 | 7.92E-08 | 0.0281 | AATAGTAATGATGAT |
| *Glyma.02g261700* | *Glyma02g42960* | MP00302 | *Glyma.15g152100* | *Glyma15g16270* | 1448 | 1468 | - | 19.7571 | 1.13E-07 | 0.0085 | GGCGGAGACGACGGCGGAGAC |
| *Glyma.02g261700* | *Glyma02g42960* | MP00302 | *Glyma.20g172000* | *Glyma20g31200* | 1331 | 1351 | + | 19.7143 | 1.16E-07 | 0.0085 | GGTTTTGGTGGTGGTGGTGGT |
| *Glyma.02g261700* | *Glyma02g42960* | MP00302 | *Glyma.20g172000* | *Glyma20g31200* | 1331 | 1351 | + | 19.7143 | 1.16E-07 | 0.0085 | GGTTTTGGTGGTGGTGGTGGT |
| *Glyma.02g261700* | *Glyma02g42960* | MP00302 | *Glyma.20g172000* | *Glyma20g31200* | 1520 | 1540 | + | 19.7143 | 1.16E-07 | 0.0085 | GGTTTTGGTGGTGGTGGTGGT |
| *Glyma.02g261700* | *Glyma02g42960* | MP00302 | *Glyma.17g005600* | *Glyma17g00950* | 554 | 574 | + | 19.4286 | 1.45E-07 | 0.0099 | TGAATCGGTGGTGGTGGTGGA |
| *Glyma.02g261700* | *Glyma02g42960* | MP00302 | *Glyma.15g152100* | *Glyma15g16270* | 1451 | 1471 | - | 18.5143 | 2.88E-07 | 0.0133 | GCTGGCGGAGACGACGGCGGA |
| *Glyma.20g160200* | *Glyma20g29960* | MP00449 | *Glyma.09g273900* | *Glyma09g41040* | 423 | 437 | + | 15.3333 | 3.99E-07 | 0.237 | ATTTGACTTTTCAAA |
| *Glyma.02g261700* | *Glyma02g42960* | MP00302 | *Glyma.20g172000* | *Glyma20g31200* | 1328 | 1348 | + | 17.1857 | 7.31E-07 | 0.0222 | TAAGGTTTTGGTGGTGGTGGT |
| *Glyma.02g261700* | *Glyma02g42960* | MP00302 | *Glyma.20g172000* | *Glyma20g31200* | 1328 | 1348 | + | 17.1857 | 7.31E-07 | 0.0222 | TAAGGTTTTGGTGGTGGTGGT |
| *Glyma.02g261700* | *Glyma02g42960* | MP00302 | *Glyma.20g172000* | *Glyma20g31200* | 1517 | 1537 | + | 17.1857 | 7.31E-07 | 0.0222 | TAAGGTTTTGGTGGTGGTGGT |
| *Glyma.02g261700* | *Glyma02g42960* | MP00302 | *Glyma.06g152800* | *Glyma06g15820* | 1505 | 1525 | - | 16.8714 | 9.04E-07 | 0.0229 | TGGTGCGGTGGTGGTGGTTGT |
| *Glyma.10g053500* | *Glyma10g06080* | MP00461 | *Glyma.06g152800* | *Glyma06g15820* | 1682 | 1702 | + | 9.77108 | 1.18E-06 | 0.116 | TTTGCCATTTAAGGGGGAAAA |
| *Glyma.02g261700* | *Glyma02g42960* | MP00302 | *Glyma.20g176300* | *Glyma20g31630* | 1333 | 1353 | + | 16.4571 | 1.19E-06 | 0.0272 | GGAAGAGGAAGAGGAGGAGGA |
| *Glyma.02g261700* | *Glyma02g42960* | MP00302 | *Glyma.07g069700* | *Glyma07g07580* | 1761 | 1781 | + | 16.4 | 1.23E-06 | 0.0272 | GGAGGAGGATGAGGCCGCGGA |
| *Glyma.20g160200* | *Glyma20g29960* | MP00449 | *Glyma.09g273900* | *Glyma09g41040* | 944 | 958 | + | 14.8182 | 1.30E-06 | 0.28 | ATATGACTTTTCAAA |
| *Glyma.02g261700* | *Glyma02g42960* | MP00302 | *Glyma.20g172000* | *Glyma20g31200* | 1325 | 1345 | + | 16.0143 | 1.58E-06 | 0.0313 | GGATAAGGTTTTGGTGGTGGT |
| *Glyma.02g261700* | *Glyma02g42960* | MP00302 | *Glyma.20g172000* | *Glyma20g31200* | 1325 | 1345 | + | 16.0143 | 1.58E-06 | 0.0313 | GGATAAGGTTTTGGTGGTGGT |
| *Glyma.02g261700* | *Glyma02g42960* | MP00302 | *Glyma.20g172000* | *Glyma20g31200* | 1514 | 1534 | + | 16.0143 | 1.58E-06 | 0.0313 | GGATAAGGTTTTGGTGGTGGT |
| *Glyma.10g053500* | *Glyma10g06080* | MP00461 | *Glyma.05g062200* | *Glyma05g04940* | 1912 | 1932 | + | 8.66265 | 1.71E-06 | 0.116 | TTTTCGGGTTTGGTTAGAAGA |
| *Glyma.03g143100* | *Glyma03g29901* | MP00307 | *Glyma.16g146500* | *Glyma16g26210* | 2090 | 2100 | + | 13.0758 | 1.73E-06 | 1 | ATTGTACGGTC |
| *Glyma.02g261700* | *Glyma02g42960* | MP00302 | *Glyma.07g069700* | *Glyma07g07580* | 1486 | 1506 | - | 15.8571 | 1.75E-06 | 0.0338 | GAAGGTCGTGGTGACGGCGAA |
| *Glyma.02g261700* | *Glyma02g42960* | MP00302 | *Glyma.07g069700* | *Glyma07g07580* | 2012 | 2032 | - | 15.8571 | 1.75E-06 | 0.0338 | GAAGGTCGTGGTGACGGCGAA |
| *Glyma.10g053500* | *Glyma10g06080* | MP00461 | *Glyma.05g000200* | *Glyma05g09400* | 161 | 181 | - | 8.21687 | 1.99E-06 | 0.116 | TTTTTGTTTTTGGGAGGACTA |
| *Glyma.10g053500* | *Glyma10g06080* | MP00461 | *Glyma.05g000200* | *Glyma05g09400* | 161 | 181 | - | 8.21687 | 1.99E-06 | 0.116 | TTTTTGTTTTTGGGAGGACTA |
| *Glyma.10g053500* | *Glyma10g06080* | MP00461 | *Glyma.05g000200* | *Glyma05g09400* | 161 | 181 | - | 8.21687 | 1.99E-06 | 0.116 | TTTTTGTTTTTGGGAGGACTA |
| *Glyma.10g053500* | *Glyma10g06080* | MP00461 | *Glyma.05g000200* | *Glyma05g09400* | 161 | 181 | - | 8.21687 | 1.99E-06 | 0.116 | TTTTTGTTTTTGGGAGGACTA |
| *Glyma.02g261700* | *Glyma02g42960* | MP00302 | *Glyma.07g069700* | *Glyma07g07580* | 1764 | 1784 | + | 15.6 | 2.05E-06 | 0.0367 | GGAGGATGAGGCCGCGGAGGA |
| *Glyma.10g053500* | *Glyma10g06080* | MP00461 | *Glyma.10g181900* | *Glyma10g32520* | 1012 | 1032 | - | 8.10843 | 2.06E-06 | 0.116 | TTCATGGTTGTGGTTGGAAAG |
| *Glyma.02g261700* | *Glyma02g42960* | MP00302 | *Glyma.06g070100* | *Glyma06g07360* | 354 | 374 | - | 15.3143 | 2.45E-06 | 0.0397 | TGAAATGGTGGTGGTGGATGT |
| *Glyma.02g261700* | *Glyma02g42960* | MP00302 | *Glyma.06g070100* | *Glyma06g07360* | 366 | 386 | - | 15.3143 | 2.45E-06 | 0.0397 | TGAAATGGTGGTGGTGGATGT |
| *Glyma.02g261700* | *Glyma02g42960* | MP00302 | *Glyma.06g070100* | *Glyma06g07360* | 811 | 831 | - | 15.3143 | 2.45E-06 | 0.0397 | TGAAATGGTGGTGGTGGATGT |
| *Glyma.02g261700* | *Glyma02g42960* | MP00302 | *Glyma.06g070100* | *Glyma06g07360* | 947 | 967 | - | 15.3143 | 2.45E-06 | 0.0397 | TGAAATGGTGGTGGTGGATGT |
| *Glyma.02g239600* | *Glyma02g40650* | MP00033 | *Glyma.08g156400* | *Glyma08g16620* | 2011 | 2019 | + | 12.08 | 3.31E-06 | 1 | TTTGTCGGC |
| *Glyma.10g053500* | *Glyma10g06080* | MP00461 | *Glyma.20g013500* | *Glyma20g01680* | 1306 | 1326 | + | 6.60241 | 3.36E-06 | 0.116 | TTTTTTTTTTTAACAAAAAAA |
| *Glyma.02g261700* | *Glyma02g42960* | MP00302 | *Glyma.20g176300* | *Glyma20g31630* | 1247 | 1267 | - | 14.5143 | 3.97E-06 | 0.0575 | GGCGCCCGTCGCGGCGGCGGT |
| *Glyma.02g261700* | *Glyma02g42960* | MP00302 | *Glyma.11g248000* | *Glyma11g37320* | 1657 | 1677 | + | 14.5143 | 3.97E-06 | 0.0575 | GGAGGAGGCGAAATTGGCGGT |
| *Glyma.02g261700* | *Glyma02g42960* | MP00302 | *Glyma.11g248000* | *Glyma11g37320* | 1942 | 1962 | + | 14.5143 | 3.97E-06 | 0.0575 | GGAGGAGGCGAAATTGGCGGT |
| *Glyma.08g201900* | *Glyma08g21610* | MP00200 | *Glyma.05g000200* | *Glyma05g09400* | 1627 | 1641 | + | 15.3433 | 4.21E-06 | 0.142 | TAATGTGATGATGAT |
| *Glyma.08g201900* | *Glyma08g21610* | MP00200 | *Glyma.05g000200* | *Glyma05g09400* | 1627 | 1641 | + | 15.3433 | 4.21E-06 | 0.142 | TAATGTGATGATGAT |
| *Glyma.08g201900* | *Glyma08g21610* | MP00200 | *Glyma.05g000200* | *Glyma05g09400* | 1627 | 1641 | + | 15.3433 | 4.21E-06 | 0.142 | TAATGTGATGATGAT |
| *Glyma.08g201900* | *Glyma08g21610* | MP00200 | *Glyma.05g000200* | *Glyma05g09400* | 1627 | 1641 | + | 15.3433 | 4.21E-06 | 0.142 | TAATGTGATGATGAT |
| *Glyma.13g187500* | *Glyma13g25716* | MP00490 | *Glyma.06g152800* | *Glyma06g15820* | 528 | 538 | - | 13.5303 | 4.35E-06 | 0.538 | ATAACTGAATC |
| *Glyma.13g187500* | *Glyma13g25716* | MP00490 | *Glyma.06g152800* | *Glyma06g15820* | 1176 | 1186 | - | 13.5303 | 4.35E-06 | 0.538 | ATAACTGAATC |
| *Glyma.10g053500* | *Glyma10g06080* | MP00461 | *Glyma.12g047400* | *Glyma12g05140* | 104 | 124 | - | 5.75904 | 4.39E-06 | 0.116 | TTTACGAGTTTGTTAAAAAAA |
| *Glyma.10g053500* | *Glyma10g06080* | MP00461 | *Glyma.12g047400* | *Glyma12g05140* | 806 | 826 | - | 5.75904 | 4.39E-06 | 0.116 | TTTACGAGTTTGTTAAAAAAA |
| *Glyma.10g053500* | *Glyma10g06080* | MP00461 | *Glyma.12g047400* | *Glyma12g05140* | 806 | 826 | - | 5.75904 | 4.39E-06 | 0.116 | TTTACGAGTTTGTTAAAAAAA |
| *Glyma.02g261700* | *Glyma02g42960* | MP00302 | *Glyma.07g080800* | *Glyma07g08810* | 1253 | 1273 | - | 14.3 | 4.50E-06 | 0.0614 | GAAGGGGTTGCCGGTGGCGGA |
| *Glyma.02g261700* | *Glyma02g42960* | MP00302 | *Glyma.07g080800* | *Glyma07g08810* | 1328 | 1348 | - | 14.3 | 4.50E-06 | 0.0614 | GAAGGGGTTGCCGGTGGCGGA |
| *Glyma.02g261700* | *Glyma02g42960* | MP00302 | *Glyma.07g080800* | *Glyma07g08810* | 1226 | 1246 | - | 14.1143 | 5.01E-06 | 0.0661 | GGCGGAGGGGTGGACGGTGAC |
| *Glyma.02g261700* | *Glyma02g42960* | MP00302 | *Glyma.07g080800* | *Glyma07g08810* | 1301 | 1321 | - | 14.1143 | 5.01E-06 | 0.0661 | GGCGGAGGGGTGGACGGTGAC |
| *Glyma.20g160200* | *Glyma20g29960* | MP00449 | *Glyma.20g172000* | *Glyma20g31200* | 1495 | 1509 | - | 13.9394 | 5.54E-06 | 0.493 | TTTTGACTTTTCATT |
| *Glyma.20g160200* | *Glyma20g29960* | MP00449 | *Glyma.20g172000* | *Glyma20g31200* | 1495 | 1509 | - | 13.9394 | 5.54E-06 | 0.493 | TTTTGACTTTTCATT |
| *Glyma.20g160200* | *Glyma20g29960* | MP00449 | *Glyma.20g172000* | *Glyma20g31200* | 1684 | 1698 | - | 13.9394 | 5.54E-06 | 0.493 | TTTTGACTTTTCATT |
| *Glyma.10g053500* | *Glyma10g06080* | MP00461 | *Glyma.20g013500* | *Glyma20g01680* | 1311 | 1331 | - | 4.83133 | 5.86E-06 | 0.13 | TTGATTTTTTTTGTTAAAAAA |
| *Glyma.08g201900* | *Glyma08g21610* | MP00200 | *Glyma.05g246500* | *Glyma05g33790* | 113 | 127 | + | 14.8955 | 5.86E-06 | 0.142 | TATAATAATCATTAT |
| *Glyma.08g201900* | *Glyma08g21610* | MP00200 | *Glyma.05g246500* | *Glyma05g33790* | 239 | 253 | + | 14.8955 | 5.86E-06 | 0.142 | TATAATAATCATTAT |
| *Glyma.08g201900* | *Glyma08g21610* | MP00200 | *Glyma.06g152800* | *Glyma06g15820* | 538 | 552 | - | 14.8358 | 6.11E-06 | 0.142 | CAATGTGATGATGAC |
| *Glyma.13g187500* | *Glyma13g25716* | MP00490 | *Glyma.05g062200* | *Glyma05g04940* | 2033 | 2043 | + | 13.3485 | 6.14E-06 | 0.567 | GTAACCGACTC |
| *Glyma.02g261700* | *Glyma02g42960* | MP00302 | *Glyma.20g172000* | *Glyma20g31200* | 1334 | 1354 | + | 13.7143 | 6.30E-06 | 0.0695 | TTTGGTGGTGGTGGTGGTGTT |
| *Glyma.02g261700* | *Glyma02g42960* | MP00302 | *Glyma.20g172000* | *Glyma20g31200* | 1334 | 1354 | + | 13.7143 | 6.30E-06 | 0.0695 | TTTGGTGGTGGTGGTGGTGTT |
| *Glyma.02g261700* | *Glyma02g42960* | MP00302 | *Glyma.20g172000* | *Glyma20g31200* | 1523 | 1543 | + | 13.7143 | 6.30E-06 | 0.0695 | TTTGGTGGTGGTGGTGGTGTT |
| *Glyma.08g201900* | *Glyma08g21610* | MP00200 | *Glyma.06g070100* | *Glyma06g07360* | 1980 | 1994 | - | 14.7463 | 6.48E-06 | 0.142 | GAATGTGATGATGAG |
| *Glyma.08g201900* | *Glyma08g21610* | MP00200 | *Glyma.06g070100* | *Glyma06g07360* | 1992 | 2006 | - | 14.7463 | 6.48E-06 | 0.142 | GAATGTGATGATGAG |
| *Glyma.02g261700* | *Glyma02g42960* | MP00302 | *Glyma.15g152100* | *Glyma15g16270* | 1557 | 1577 | - | 13.5429 | 6.94E-06 | 0.0695 | GGAGACGGAGACTATGGAGGC |
| *Glyma.02g261700* | *Glyma02g42960* | MP00302 | *Glyma.11g248000* | *Glyma11g37320* | 1660 | 1680 | + | 13.5286 | 6.99E-06 | 0.0695 | GGAGGCGAAATTGGCGGTTCG |
| *Glyma.02g261700* | *Glyma02g42960* | MP00302 | *Glyma.11g248000* | *Glyma11g37320* | 1945 | 1965 | + | 13.5286 | 6.99E-06 | 0.0695 | GGAGGCGAAATTGGCGGTTCG |
| *Glyma.02g261700* | *Glyma02g42960* | MP00302 | *Glyma.07g080800* | *Glyma07g08810* | 1238 | 1258 | - | 13.3857 | 7.58E-06 | 0.0729 | GGCGGATGGCGCGGCGGAGGG |
| *Glyma.02g261700* | *Glyma02g42960* | MP00302 | *Glyma.07g080800* | *Glyma07g08810* | 1313 | 1333 | - | 13.3857 | 7.58E-06 | 0.0729 | GGCGGATGGCGCGGCGGAGGG |
| *Glyma.02g261700* | *Glyma02g42960* | MP00302 | *Glyma.07g080800* | *Glyma07g08810* | 1250 | 1270 | - | 13.3714 | 7.64E-06 | 0.0729 | GGGGTTGCCGGTGGCGGATGG |
| *Glyma.02g261700* | *Glyma02g42960* | MP00302 | *Glyma.07g080800* | *Glyma07g08810* | 1325 | 1345 | - | 13.3714 | 7.64E-06 | 0.0729 | GGGGTTGCCGGTGGCGGATGG |
| *Glyma.02g261700* | *Glyma02g42960* | MP00302 | *Glyma.07g080800* | *Glyma07g08810* | 1241 | 1261 | - | 13.3429 | 7.76E-06 | 0.0733 | GGTGGCGGATGGCGCGGCGGA |
| *Glyma.02g261700* | *Glyma02g42960* | MP00302 | *Glyma.07g080800* | *Glyma07g08810* | 1316 | 1336 | - | 13.3429 | 7.76E-06 | 0.0733 | GGTGGCGGATGGCGCGGCGGA |
| *Glyma.08g201900* | *Glyma08g21610* | MP00200 | *Glyma.07g080800* | *Glyma07g08810* | 954 | 968 | - | 14.4627 | 7.90E-06 | 0.142 | AAAAGTAATAATTAA |
| *Glyma.08g201900* | *Glyma08g21610* | MP00200 | *Glyma.07g080800* | *Glyma07g08810* | 1029 | 1043 | - | 14.4627 | 7.90E-06 | 0.142 | AAAAGTAATAATTAA |
| *Glyma.08g201900* | *Glyma08g21610* | MP00200 | *Glyma.07g080800* | *Glyma07g08810* | 1928 | 1942 | - | 14.4627 | 7.90E-06 | 0.142 | AAAAGTAATAATTAA |
| *Glyma.08g201900* | *Glyma08g21610* | MP00200 | *Glyma.05g246500* | *Glyma05g33790* | 117 | 131 | - | 14.4478 | 7.98E-06 | 0.142 | CTTAATAATGATTAT |
| *Glyma.08g201900* | *Glyma08g21610* | MP00200 | *Glyma.05g246500* | *Glyma05g33790* | 243 | 257 | - | 14.4478 | 7.98E-06 | 0.142 | CTTAATAATGATTAT |
| *Glyma.08g097900* | *Glyma08g10350* | MP00006 | *Glyma.14g043500* | *Glyma14g04780* | 55 | 62 | - | 15.5772 | 8.13E-06 | 0.971 | GGGCCCAC |
| *Glyma.08g097900* | *Glyma08g10350* | MP00006 | *Glyma.11g248000* | *Glyma11g37320* | 1955 | 1962 | - | 15.5772 | 8.13E-06 | 0.971 | GGGCCCAC |
| *Glyma.02g261700* | *Glyma02g42960* | MP00302 | *Glyma.15g152100* | *Glyma15g16270* | 1306 | 1326 | - | 13.1143 | 8.81E-06 | 0.0812 | TGCAACGACGTCGTTGGTGGT |
| *Glyma.08g201900* | *Glyma08g21610* | MP00200 | *Glyma.13g162600* | *Glyma13g23150* | 707 | 721 | + | 14.2388 | 9.15E-06 | 0.149 | ATGAATAATGATTAA |
| *Glyma.20g160200* | *Glyma20g29960* | MP00449 | *Glyma.07g080800* | *Glyma07g08810* | 87 | 101 | - | 13.5455 | 9.24E-06 | 0.493 | AACTGACTTTTGGGA |
| *Glyma.02g239600* | *Glyma02g40650* | MP00033 | *Glyma.13g364900* | *Glyma13g44170* | 894 | 902 | - | 11.97 | 9.34E-06 | 1 | ATTGTCGGC |
| *Glyma.02g239600* | *Glyma02g40650* | MP00033 | *Glyma.13g364900* | *Glyma13g44170* | 933 | 941 | - | 11.97 | 9.34E-06 | 1 | ATTGTCGGC |
| *Glyma.13g219900* | *Glyma13g29160* | MP00639 | *Glyma.06g070100* | *Glyma06g07360* | 84 | 91 | - | 15.102 | 9.90E-06 | 0.822 | GGGACCAC |
| *Glyma.13g219900* | *Glyma13g29160* | MP00639 | *Glyma.06g070100* | *Glyma06g07360* | 96 | 103 | - | 15.102 | 9.90E-06 | 0.822 | GGGACCAC |
| *Glyma.13g219900* | *Glyma13g29160* | MP00639 | *Glyma.06g070100* | *Glyma06g07360* | 366 | 373 | + | 15.102 | 9.90E-06 | 0.822 | GGGACCAC |
| *Glyma.13g219900* | *Glyma13g29160* | MP00639 | *Glyma.06g070100* | *Glyma06g07360* | 502 | 509 | + | 15.102 | 9.90E-06 | 0.822 | GGGACCAC |
| *Glyma.13g219900* | *Glyma13g29160* | MP00639 | *Glyma.06g070100* | *Glyma06g07360* | 541 | 548 | - | 15.102 | 9.90E-06 | 0.822 | GGGACCAC |
| *Glyma.13g219900* | *Glyma13g29160* | MP00639 | *Glyma.06g070100* | *Glyma06g07360* | 677 | 684 | - | 15.102 | 9.90E-06 | 0.822 | GGGACCAC |
| *Glyma.02g261700* | *Glyma02g42960* | MP00302 | *Glyma.07g069700* | *Glyma07g07580* | 1770 | 1790 | + | 12.7714 | 1.06E-05 | 0.0929 | TGAGGCCGCGGAGGAGGCTGC |
| *Glyma.10g053500* | *Glyma10g06080* | MP00461 | *Glyma.13g162600* | *Glyma13g23150* | 1230 | 1250 | - | 2.66265 | 1.12E-05 | 0.139 | TTTTTTTTTTTTAGAAAAAAA |
| *Glyma.02g261700* | *Glyma02g42960* | MP00302 | *Glyma.07g080800* | *Glyma07g08810* | 1450 | 1470 | + | 12.6571 | 1.13E-05 | 0.0957 | CGTGGCGACGAAGGTGGCGCT |
| *Glyma.02g261700* | *Glyma02g42960* | MP00302 | *Glyma.07g080800* | *Glyma07g08810* | 1525 | 1545 | + | 12.6571 | 1.13E-05 | 0.0957 | CGTGGCGACGAAGGTGGCGCT |
| *Glyma.02g261700* | *Glyma02g42960* | MP00302 | *Glyma.10g181900* | *Glyma10g32520* | 1107 | 1127 | - | 12.6571 | 1.13E-05 | 0.0957 | GTTGGATGCTTAGGTGGAGGA |
| *Glyma.13g187500* | *Glyma13g25716* | MP00490 | *Glyma.08g345900* | *Glyma08g45990* | 1010 | 1020 | - | 13.0152 | 1.14E-05 | 0.735 | GTAACTGAATT |
| *Glyma.13g187500* | *Glyma13g25716* | MP00490 | *Glyma.08g345900* | *Glyma08g45990* | 1220 | 1230 | - | 13.0152 | 1.14E-05 | 0.735 | GTAACTGAATT |
| *Glyma.02g261700* | *Glyma02g42960* | MP00302 | *Glyma.13g162600* | *Glyma13g23150* | 1638 | 1658 | - | 12.6429 | 1.14E-05 | 0.096 | GATGATGACAGCGATGGAGGC |
| *Glyma.10g053500* | *Glyma10g06080* | MP00461 | *Glyma.05g246500* | *Glyma05g33790* | 1248 | 1268 | - | 2.60241 | 1.14E-05 | 0.139 | ATTACATTTTATATTAAAAAA |
| *Glyma.10g053500* | *Glyma10g06080* | MP00461 | *Glyma.05g246500* | *Glyma05g33790* | 1374 | 1394 | - | 2.60241 | 1.14E-05 | 0.139 | ATTACATTTTATATTAAAAAA |
| *Glyma.20g160200* | *Glyma20g29960* | MP00449 | *Glyma.12g047400* | *Glyma12g05140* | 1607 | 1621 | - | 13.3485 | 1.16E-05 | 0.493 | TGATGGCTTTTCATG |
| *Glyma.03g143100* | *Glyma03g29901* | MP00307 | *Glyma.08g349200* | *Glyma08g46360* | 22 | 32 | - | 12.3182 | 1.27E-05 | 1 | GTTGTACTGAT |
| *Glyma.10g053500* | *Glyma10g06080* | MP00461 | *Glyma.13g183800* | *Glyma13g25290* | 1682 | 1702 | + | 2.13253 | 1.31E-05 | 0.139 | TATTCTTTTTTGGGATAAAAT |
| *Glyma.10g053500* | *Glyma10g06080* | MP00461 | *Glyma.20g172000* | *Glyma20g31200* | 977 | 997 | - | 2.12048 | 1.31E-05 | 0.139 | TATATTTTTTAAGTTGAAAAA |
| *Glyma.10g053500* | *Glyma10g06080* | MP00461 | *Glyma.20g172000* | *Glyma20g31200* | 1292 | 1312 | - | 2.12048 | 1.31E-05 | 0.139 | TATATTTTTTAAGTTGAAAAA |
| *Glyma.02g261700* | *Glyma02g42960* | MP00302 | *Glyma.08g156400* | *Glyma08g16620* | 2067 | 2087 | - | 12.3571 | 1.33E-05 | 0.101 | GTCGATGATTGTTGCGGTGAC |
| *Glyma.10g053500* | *Glyma10g06080* | MP00461 | *Glyma.15g171900* | *Glyma15g18770* | 1532 | 1552 | - | 1.93976 | 1.38E-05 | 0.139 | TTTATATTTGAAACAAAAAAA |
| *Glyma.10g053500* | *Glyma10g06080* | MP00461 | *Glyma.15g171900* | *Glyma15g18770* | 1548 | 1568 | - | 1.93976 | 1.38E-05 | 0.139 | TTTATATTTGAAACAAAAAAA |
| *Glyma.10g053500* | *Glyma10g06080* | MP00461 | *Glyma.15g171900* | *Glyma15g18770* | 1548 | 1568 | - | 1.93976 | 1.38E-05 | 0.139 | TTTATATTTGAAACAAAAAAA |
| *Glyma.02g261700* | *Glyma02g42960* | MP00302 | *Glyma.03g242600* | *Glyma03g40250* | 1990 | 2010 | - | 12.2429 | 1.41E-05 | 0.105 | AGGAGAGAGGGTGGCGGAGAA |
| *Glyma.10g053500* | *Glyma10g06080* | MP00461 | *Glyma.20g172000* | *Glyma20g31200* | 104 | 124 | + | 1.83133 | 1.43E-05 | 0.139 | TATAGGATTTAGGTGGAAAAG |
| *Glyma.10g053500* | *Glyma10g06080* | MP00461 | *Glyma.20g172000* | *Glyma20g31200* | 1656 | 1676 | + | 1.83133 | 1.43E-05 | 0.139 | TATAGGATTTAGGTGGAAAAG |
| *Glyma.10g053500* | *Glyma10g06080* | MP00461 | *Glyma.20g172000* | *Glyma20g31200* | 1656 | 1676 | + | 1.83133 | 1.43E-05 | 0.139 | TATAGGATTTAGGTGGAAAAG |
| *Glyma.10g053500* | *Glyma10g06080* | MP00461 | *Glyma.20g172000* | *Glyma20g31200* | 1845 | 1865 | + | 1.83133 | 1.43E-05 | 0.139 | TATAGGATTTAGGTGGAAAAG |
| *Glyma.08g201900* | *Glyma08g21610* | MP00200 | *Glyma.05g000200* | *Glyma05g09400* | 580 | 594 | - | 13.5075 | 1.43E-05 | 0.156 | AAGGACAATGATTAA |
| *Glyma.08g201900* | *Glyma08g21610* | MP00200 | *Glyma.05g000200* | *Glyma05g09400* | 580 | 594 | - | 13.5075 | 1.43E-05 | 0.156 | AAGGACAATGATTAA |
| *Glyma.08g201900* | *Glyma08g21610* | MP00200 | *Glyma.05g000200* | *Glyma05g09400* | 580 | 594 | - | 13.5075 | 1.43E-05 | 0.156 | AAGGACAATGATTAA |
| *Glyma.08g201900* | *Glyma08g21610* | MP00200 | *Glyma.05g000200* | *Glyma05g09400* | 580 | 594 | - | 13.5075 | 1.43E-05 | 0.156 | AAGGACAATGATTAA |
| *Glyma.20g160200* | *Glyma20g29960* | MP00449 | *Glyma.13g183800* | *Glyma13g25290* | 59 | 73 | - | 13.1667 | 1.43E-05 | 0.493 | ATGTGTCTTTTCGTG |
| *Glyma.02g261700* | *Glyma02g42960* | MP00302 | *Glyma.20g176300* | *Glyma20g31630* | 1244 | 1264 | - | 12.1857 | 1.45E-05 | 0.106 | GCCCGTCGCGGCGGCGGTTGC |
| *Glyma.08g201900* | *Glyma08g21610* | MP00200 | *Glyma.05g000200* | *Glyma05g09400* | 1607 | 1621 | - | 13.4776 | 1.46E-05 | 0.156 | GTAAATGATGATGAT |
| *Glyma.08g201900* | *Glyma08g21610* | MP00200 | *Glyma.05g000200* | *Glyma05g09400* | 1607 | 1621 | - | 13.4776 | 1.46E-05 | 0.156 | GTAAATGATGATGAT |
| *Glyma.08g201900* | *Glyma08g21610* | MP00200 | *Glyma.05g000200* | *Glyma05g09400* | 1607 | 1621 | - | 13.4776 | 1.46E-05 | 0.156 | GTAAATGATGATGAT |
| *Glyma.08g201900* | *Glyma08g21610* | MP00200 | *Glyma.05g000200* | *Glyma05g09400* | 1607 | 1621 | - | 13.4776 | 1.46E-05 | 0.156 | GTAAATGATGATGAT |
| *Glyma.10g053500* | *Glyma10g06080* | MP00461 | *Glyma.13g364900* | *Glyma13g44170* | 976 | 996 | + | 1.75904 | 1.46E-05 | 0.139 | TTAATTTTTATTAGGAAAAAA |
| *Glyma.10g053500* | *Glyma10g06080* | MP00461 | *Glyma.13g364900* | *Glyma13g44170* | 1015 | 1035 | + | 1.75904 | 1.46E-05 | 0.139 | TTAATTTTTATTAGGAAAAAA |
| *Glyma.02g013900* | *Glyma02g01740* | MP00627 | *Glyma.15g152100* | *Glyma15g16270* | 679 | 688 | + | 13.8265 | 1.47E-05 | 1 | CTACCTACCA |
| *Glyma.08g201900* | *Glyma08g21610* | MP00200 | *Glyma.06g152800* | *Glyma06g15820* | 728 | 742 | + | 13.4627 | 1.47E-05 | 0.156 | ATATGTAATAATTAT |
| *Glyma.08g201900* | *Glyma08g21610* | MP00200 | *Glyma.06g152800* | *Glyma06g15820* | 1376 | 1390 | + | 13.4627 | 1.47E-05 | 0.156 | ATATGTAATAATTAT |
| *Glyma.02g261700* | *Glyma02g42960* | MP00302 | *Glyma.17g005600* | *Glyma17g00950* | 548 | 568 | + | 12.1 | 1.52E-05 | 0.106 | TGAAAATGAATCGGTGGTGGT |
| *Glyma.02g261700* | *Glyma02g42960* | MP00302 | *Glyma.10g181900* | *Glyma10g32520* | 1104 | 1124 | - | 12.0714 | 1.54E-05 | 0.106 | GGATGCTTAGGTGGAGGAGAA |
| *Glyma.13g187500* | *Glyma13g25716* | MP00490 | *Glyma.13g364900* | *Glyma13g44170* | 3 | 13 | - | 12.8788 | 1.54E-05 | 0.735 | TTAACCGAATT |
| *Glyma.10g053500* | *Glyma10g06080* | MP00461 | *Glyma.07g080800* | *Glyma07g08810* | 1840 | 1860 | + | 1.49398 | 1.57E-05 | 0.139 | TTTTTTTTTTTTGTAAAAAGA |
| *Glyma.10g053500* | *Glyma10g06080* | MP00461 | *Glyma.07g080800* | *Glyma07g08810* | 1915 | 1935 | + | 1.49398 | 1.57E-05 | 0.139 | TTTTTTTTTTTTGTAAAAAGA |
| *Glyma.02g261700* | *Glyma02g42960* | MP00302 | *Glyma.17g005600* | *Glyma17g00950* | 1892 | 1912 | + | 11.9571 | 1.63E-05 | 0.107 | GGAGCTTGCGGTAGCCGTGAA |
| *Glyma.02g261700* | *Glyma02g42960* | MP00302 | *Glyma.13g162600* | *Glyma13g23150* | 1826 | 1846 | + | 11.8286 | 1.75E-05 | 0.109 | GGTGGTTTGACCGGCGGAGAA |
| *Glyma.10g053500* | *Glyma10g06080* | MP00461 | *Glyma.14g043500* | *Glyma14g04780* | 826 | 846 | - | 1.07229 | 1.77E-05 | 0.147 | TTTATCTATATGGCTGAAAAT |
| *Glyma.10g053500* | *Glyma10g06080* | MP00461 | *Glyma.14g043500* | *Glyma14g04780* | 1108 | 1128 | - | 1.07229 | 1.77E-05 | 0.147 | TTTATCTATATGGCTGAAAAT |
| *Glyma.10g053500* | *Glyma10g06080* | MP00461 | *Glyma.14g043500* | *Glyma14g04780* | 1476 | 1496 | - | 1.07229 | 1.77E-05 | 0.147 | TTTATCTATATGGCTGAAAAT |
| *Glyma.13g187500* | *Glyma13g25716* | MP00490 | *Glyma.16g018300* | *Glyma16g02090* | 1391 | 1401 | + | 12.7424 | 1.78E-05 | 0.735 | ATAACCGTCTT |
| *Glyma.02g261700* | *Glyma02g42960* | MP00302 | *Glyma.11g248000* | *Glyma11g37320* | 1374 | 1394 | - | 11.7429 | 1.83E-05 | 0.11 | ATCGCCGGTTGTAGTGGTGAC |
| *Glyma.02g261700* | *Glyma02g42960* | MP00302 | *Glyma.11g248000* | *Glyma11g37320* | 1659 | 1679 | - | 11.7429 | 1.83E-05 | 0.11 | ATCGCCGGTTGTAGTGGTGAC |
| *Glyma.08g201900* | *Glyma08g21610* | MP00200 | *Glyma.07g080800* | *Glyma07g08810* | 422 | 436 | - | 13.0448 | 1.87E-05 | 0.156 | TAATATAATAATTAT |
| *Glyma.02g261700* | *Glyma02g42960* | MP00302 | *Glyma.15g152100* | *Glyma15g16270* | 1466 | 1486 | + | 11.6857 | 1.88E-05 | 0.112 | GCCAGCCGCGGCGGCCGAGGA |
| *Glyma.02g261700* | *Glyma02g42960* | MP00302 | *Glyma.06g152800* | *Glyma06g15820* | 1508 | 1528 | - | 11.6714 | 1.89E-05 | 0.112 | CAATGGTGCGGTGGTGGTGGT |
| *Glyma.08g201900* | *Glyma08g21610* | MP00200 | *Glyma.05g000200* | *Glyma05g09400* | 616 | 630 | + | 13.0149 | 1.90E-05 | 0.156 | GCTAACAATGATGAC |
| *Glyma.08g201900* | *Glyma08g21610* | MP00200 | *Glyma.05g000200* | *Glyma05g09400* | 616 | 630 | + | 13.0149 | 1.90E-05 | 0.156 | GCTAACAATGATGAC |
| *Glyma.08g201900* | *Glyma08g21610* | MP00200 | *Glyma.05g000200* | *Glyma05g09400* | 616 | 630 | + | 13.0149 | 1.90E-05 | 0.156 | GCTAACAATGATGAC |
| *Glyma.08g201900* | *Glyma08g21610* | MP00200 | *Glyma.05g000200* | *Glyma05g09400* | 616 | 630 | + | 13.0149 | 1.90E-05 | 0.156 | GCTAACAATGATGAC |
| *Glyma.10g053500* | *Glyma10g06080* | MP00461 | *Glyma.03g261000* | *Glyma03g42190* | 1916 | 1936 | + | 0.795181 | 1.91E-05 | 0.148 | TATTTTTTTTATACAGAAAAA |
| *Glyma.02g261700* | *Glyma02g42960* | MP00302 | *Glyma.10g014400* | *Glyma10g01770* | 1894 | 1914 | + | 11.6429 | 1.92E-05 | 0.112 | CGAGGCGACGGCGTCGTTGGG |
| *Glyma.02g261700* | *Glyma02g42960* | MP00302 | *Glyma.10g014400* | *Glyma10g01770* | 1902 | 1922 | + | 11.6429 | 1.92E-05 | 0.112 | CGAGGCGACGGCGTCGTTGGG |
| *Glyma.02g261700* | *Glyma02g42960* | MP00302 | *Glyma.17g005600* | *Glyma17g00950* | 1962 | 1982 | + | 11.6429 | 1.92E-05 | 0.112 | AGAGATGAAGTAGGCGTTGGA |
| *Glyma.08g201900* | *Glyma08g21610* | MP00200 | *Glyma.12g047400* | *Glyma12g05140* | 774 | 788 | + | 13 | 1.92E-05 | 0.156 | AATTATAATAATTAA |
| *Glyma.08g201900* | *Glyma08g21610* | MP00200 | *Glyma.12g047400* | *Glyma12g05140* | 1476 | 1490 | + | 13 | 1.92E-05 | 0.156 | AATTATAATAATTAA |
| *Glyma.08g201900* | *Glyma08g21610* | MP00200 | *Glyma.12g047400* | *Glyma12g05140* | 1476 | 1490 | + | 13 | 1.92E-05 | 0.156 | AATTATAATAATTAA |
| *Glyma.20g160200* | *Glyma20g29960* | MP00449 | *Glyma.13g183800* | *Glyma13g25290* | 975 | 989 | - | 12.8788 | 1.93E-05 | 0.493 | TGATGACCTTTGGAT |
| *Glyma.08g201900* | *Glyma08g21610* | MP00200 | *Glyma.07g081700* | *Glyma07g08950* | 823 | 837 | + | 12.9851 | 1.94E-05 | 0.156 | ATTGATAATCATTAA |
| *Glyma.02g261700* | *Glyma02g42960* | MP00302 | *Glyma.07g069700* | *Glyma07g07580* | 1468 | 1488 | - | 11.6 | 1.96E-05 | 0.112 | GAATCCGACGCTGATGGTGGA |
| *Glyma.02g261700* | *Glyma02g42960* | MP00302 | *Glyma.07g069700* | *Glyma07g07580* | 1994 | 2014 | - | 11.6 | 1.96E-05 | 0.112 | GAATCCGACGCTGATGGTGGA |
| *Glyma.02g261700* | *Glyma02g42960* | MP00302 | *Glyma.20g176300* | *Glyma20g31630* | 1411 | 1431 | + | 11.5714 | 1.99E-05 | 0.113 | GGGAGATCCGACGGCGGCGAC |
| *Glyma.08g201900* | *Glyma08g21610* | MP00200 | *Glyma.13g162600* | *Glyma13g23150* | 689 | 703 | + | 12.9104 | 2.02E-05 | 0.158 | TATTGTAATAATTAA |
| *Glyma.10g053500* | *Glyma10g06080* | MP00461 | *Glyma.13g183800* | *Glyma13g25290* | 1978 | 1998 | - | 0.493976 | 2.08E-05 | 0.151 | TTTGGTGGTGTGGTGGAAAAA |
| *Glyma.10g053500* | *Glyma10g06080* | MP00461 | *Glyma.15g152100* | *Glyma15g16270* | 69 | 89 | - | 0.373494 | 2.15E-05 | 0.153 | TTTTTATTTATGAGAAAAAAT |
| *Glyma.08g201900* | *Glyma08g21610* | MP00200 | *Glyma.07g069700* | *Glyma07g07580* | 1732 | 1746 | + | 12.791 | 2.16E-05 | 0.161 | GTAGGCGATGATGAC |
| *Glyma.10g053500* | *Glyma10g06080* | MP00461 | *Glyma.18g017600* | *Glyma18g02110* | 2018 | 2038 | - | 0.325301 | 2.18E-05 | 0.153 | TTTGTTTTTTTGGTAAGGCAG |
| *Glyma.13g219900* | *Glyma13g29160* | MP00639 | *Glyma.15g171900* | *Glyma15g18770* | 647 | 654 | + | 13.8265 | 2.20E-05 | 1 | GGGACCAT |
| *Glyma.13g219900* | *Glyma13g29160* | MP00639 | *Glyma.15g171900* | *Glyma15g18770* | 663 | 670 | + | 13.8265 | 2.20E-05 | 1 | GGGACCAT |
| *Glyma.13g219900* | *Glyma13g29160* | MP00639 | *Glyma.15g171900* | *Glyma15g18770* | 663 | 670 | + | 13.8265 | 2.20E-05 | 1 | GGGACCAT |
| *Glyma.02g261700* | *Glyma02g42960* | MP00302 | *Glyma.07g069700* | *Glyma07g07580* | 1767 | 1787 | + | 11.3714 | 2.21E-05 | 0.122 | GGATGAGGCCGCGGAGGAGGC |
| *Glyma.08g201900* | *Glyma08g21610* | MP00200 | *Glyma.13g162600* | *Glyma13g23150* | 681 | 695 | - | 12.7463 | 2.21E-05 | 0.162 | TACAATAATGATTGT |
| *Glyma.02g261700* | *Glyma02g42960* | MP00302 | *Glyma.20g176300* | *Glyma20g31630* | 563 | 583 | - | 11.3571 | 2.22E-05 | 0.122 | GGCGGAGAGTTTGGCGGTTCC |
| *Glyma.02g261700* | *Glyma02g42960* | MP00302 | *Glyma.18g017600* | *Glyma18g02110* | 563 | 583 | - | 11.3 | 2.29E-05 | 0.125 | AGAAGTTGCAGAGGAGGTTGT |
| *Glyma.10g053500* | *Glyma10g06080* | MP00461 | *Glyma.03g260300* | *Glyma03g42140* | 1904 | 1924 | + | 0.13253 | 2.30E-05 | 0.153 | ATTTTGATATTTATAGGAAAA |
| *Glyma.02g261700* | *Glyma02g42960* | MP00302 | *Glyma.20g176300* | *Glyma20g31630* | 1330 | 1350 | + | 11.2857 | 2.30E-05 | 0.125 | TGCGGAAGAGGAAGAGGAGGA |
| *Glyma.10g053500* | *Glyma10g06080* | MP00461 | *Glyma.03g260300* | *Glyma03g42140* | 1907 | 1927 | + | 0.108434 | 2.32E-05 | 0.153 | TTGATATTTATAGGAAAAAAA |
| *Glyma.20g160200* | *Glyma20g29960* | MP00449 | *Glyma.11g248000* | *Glyma11g37320* | 977 | 991 | - | 12.6667 | 2.38E-05 | 0.493 | AAATGACTTTTAAGG |
| *Glyma.20g160200* | *Glyma20g29960* | MP00449 | *Glyma.11g248000* | *Glyma11g37320* | 1262 | 1276 | - | 12.6667 | 2.38E-05 | 0.493 | AAATGACTTTTAAGG |
| *Glyma.10g053500* | *Glyma10g06080* | MP00461 | *Glyma.06g152800* | *Glyma06g15820* | 617 | 637 | - | 0 | 2.39E-05 | 0.153 | TTTATACTTTTAATTAAAAAT |
| *Glyma.10g053500* | *Glyma10g06080* | MP00461 | *Glyma.06g152800* | *Glyma06g15820* | 1265 | 1285 | - | 0 | 2.39E-05 | 0.153 | TTTATACTTTTAATTAAAAAT |
| *Glyma.08g201900* | *Glyma08g21610* | MP00200 | *Glyma.15g171900* | *Glyma15g18770* | 1056 | 1070 | - | 12.5821 | 2.42E-05 | 0.163 | AAAAGCGATCATTAT |
| *Glyma.08g201900* | *Glyma08g21610* | MP00200 | *Glyma.15g171900* | *Glyma15g18770* | 1072 | 1086 | - | 12.5821 | 2.42E-05 | 0.163 | AAAAGCGATCATTAT |
| *Glyma.08g201900* | *Glyma08g21610* | MP00200 | *Glyma.15g171900* | *Glyma15g18770* | 1072 | 1086 | - | 12.5821 | 2.42E-05 | 0.163 | AAAAGCGATCATTAT |
| *Glyma.08g201900* | *Glyma08g21610* | MP00200 | *Glyma.15g152100* | *Glyma15g16270* | 168 | 182 | + | 12.5522 | 2.46E-05 | 0.163 | AATAATAATGATTTT |
| *Glyma.10g053500* | *Glyma10g06080* | MP00461 | *Glyma.20g013500* | *Glyma20g01680* | 966 | 986 | - | -0.13253 | 2.47E-05 | 0.153 | TTTGTGTTATTTGTCTGAGAA |
| *Glyma.08g097900* | *Glyma08g10350* | MP00006 | *Glyma.06g070100* | *Glyma06g07360* | 84 | 91 | - | 14.3089 | 2.62E-05 | 1 | GGGACCAC |
| *Glyma.08g097900* | *Glyma08g10350* | MP00006 | *Glyma.06g070100* | *Glyma06g07360* | 96 | 103 | - | 14.3089 | 2.62E-05 | 1 | GGGACCAC |
| *Glyma.08g097900* | *Glyma08g10350* | MP00006 | *Glyma.06g070100* | *Glyma06g07360* | 366 | 373 | + | 14.3089 | 2.62E-05 | 1 | GGGACCAC |
| *Glyma.08g097900* | *Glyma08g10350* | MP00006 | *Glyma.06g070100* | *Glyma06g07360* | 502 | 509 | + | 14.3089 | 2.62E-05 | 1 | GGGACCAC |
| *Glyma.08g097900* | *Glyma08g10350* | MP00006 | *Glyma.06g070100* | *Glyma06g07360* | 541 | 548 | - | 14.3089 | 2.62E-05 | 1 | GGGACCAC |
| *Glyma.08g097900* | *Glyma08g10350* | MP00006 | *Glyma.06g070100* | *Glyma06g07360* | 677 | 684 | - | 14.3089 | 2.62E-05 | 1 | GGGACCAC |
| *Glyma.08g201900* | *Glyma08g21610* | MP00200 | *Glyma.05g012300* | *Glyma05g08060* | 1632 | 1646 | - | 12.4328 | 2.62E-05 | 0.163 | AATAATAATAATGAG |
| *Glyma.08g201900* | *Glyma08g21610* | MP00200 | *Glyma.05g012300* | *Glyma05g08060* | 1633 | 1647 | - | 12.4328 | 2.62E-05 | 0.163 | AATAATAATAATGAG |
| *Glyma.08g201900* | *Glyma08g21610* | MP00200 | *Glyma.05g012300* | *Glyma05g08060* | 1784 | 1798 | - | 12.4328 | 2.62E-05 | 0.163 | AATAATAATAATGAG |
| *Glyma.10g053500* | *Glyma10g06080* | MP00461 | *Glyma.09g273900* | *Glyma09g41040* | 304 | 324 | + | -0.445783 | 2.70E-05 | 0.154 | ATTTTGTTTTTGACTGAAACT |
| *Glyma.08g201900* | *Glyma08g21610* | MP00200 | *Glyma.05g056700* | *Glyma05g04330* | 2 | 16 | - | 12.3582 | 2.72E-05 | 0.163 | AGAATTAATGATTAA |
| *Glyma.02g261700* | *Glyma02g42960* | MP00302 | *Glyma.15g152100* | *Glyma15g16270* | 1460 | 1480 | - | 10.9429 | 2.73E-05 | 0.143 | GCCGCCGCGGCTGGCGGAGAC |
| *Glyma.10g053500* | *Glyma10g06080* | MP00461 | *Glyma.16g018300* | *Glyma16g02090* | 52 | 72 | + | -0.542169 | 2.77E-05 | 0.155 | ATTGCTGTTTTGTGCGAAAAA |
| *Glyma.20g160200* | *Glyma20g29960* | MP00449 | *Glyma.13g214600* | *Glyma13g28590* | 257 | 271 | - | 12.5 | 2.78E-05 | 0.52 | ACGTGACCCTTCATA |
| *Glyma.02g013900* | *Glyma02g01740* | MP00627 | *Glyma.17g005600* | *Glyma17g00950* | 1937 | 1946 | - | 12.1735 | 2.79E-05 | 1 | ACACCTACCT |
| *Glyma.02g261700* | *Glyma02g42960* | MP00302 | *Glyma.18g017600* | *Glyma18g02110* | 554 | 574 | - | 10.8857 | 2.81E-05 | 0.145 | AGAGGAGGTTGTGAAGGATGT |
| *Glyma.08g201900* | *Glyma08g21610* | MP00200 | *Glyma.05g012300* | *Glyma05g08060* | 607 | 621 | - | 12.2985 | 2.81E-05 | 0.163 | AAGCATGATGATGAT |
| *Glyma.08g201900* | *Glyma08g21610* | MP00200 | *Glyma.05g012300* | *Glyma05g08060* | 608 | 622 | - | 12.2985 | 2.81E-05 | 0.163 | AAGCATGATGATGAT |
| *Glyma.08g201900* | *Glyma08g21610* | MP00200 | *Glyma.05g012300* | *Glyma05g08060* | 759 | 773 | - | 12.2985 | 2.81E-05 | 0.163 | AAGCATGATGATGAT |
| *Glyma.08g201900* | *Glyma08g21610* | MP00200 | *Glyma.05g012300* | *Glyma05g08060* | 1780 | 1794 | - | 12.2985 | 2.81E-05 | 0.163 | AAGCATGATGATGAT |
| *Glyma.02g261700* | *Glyma02g42960* | MP00302 | *Glyma.16g018300* | *Glyma16g02090* | 1151 | 1171 | + | 10.8 | 2.93E-05 | 0.147 | ATTGGATCTGGGGGTGGTGGA |
| *Glyma.08g201900* | *Glyma08g21610* | MP00200 | *Glyma.15g152100* | *Glyma15g16270* | 165 | 179 | + | 12.209 | 2.95E-05 | 0.169 | ATAAATAATAATGAT |
| *Glyma.13g187500* | *Glyma13g25716* | MP00490 | *Glyma.15g152100* | *Glyma15g16270* | 92 | 102 | + | 12.3939 | 2.97E-05 | 0.844 | ATAACCGATAT |
| *Glyma.13g187500* | *Glyma13g25716* | MP00490 | *Glyma.15g152100* | *Glyma15g16270* | 928 | 938 | + | 12.3939 | 2.97E-05 | 0.844 | ATAACCGATAT |
| *Glyma.02g261700* | *Glyma02g42960* | MP00302 | *Glyma.07g080800* | *Glyma07g08810* | 1453 | 1473 | + | 10.7571 | 2.99E-05 | 0.147 | GGCGACGAAGGTGGCGCTGGC |
| *Glyma.02g261700* | *Glyma02g42960* | MP00302 | *Glyma.07g080800* | *Glyma07g08810* | 1528 | 1548 | + | 10.7571 | 2.99E-05 | 0.147 | GGCGACGAAGGTGGCGCTGGC |
| *Glyma.10g053500* | *Glyma10g06080* | MP00461 | *Glyma.07g080800* | *Glyma07g08810* | 1845 | 1865 | - | -0.855422 | 3.01E-05 | 0.161 | ATAATTCTTTTTACAAAAAAA |
| *Glyma.10g053500* | *Glyma10g06080* | MP00461 | *Glyma.07g080800* | *Glyma07g08810* | 1920 | 1940 | - | -0.855422 | 3.01E-05 | 0.161 | ATAATTCTTTTTACAAAAAAA |
| *Glyma.10g053500* | *Glyma10g06080* | MP00461 | *Glyma.08g345900* | *Glyma08g45990* | 687 | 707 | - | -0.903614 | 3.05E-05 | 0.162 | TTTTCTTTTTTTAAAAAAAAA |
| *Glyma.10g053500* | *Glyma10g06080* | MP00461 | *Glyma.08g345900* | *Glyma08g45990* | 897 | 917 | - | -0.903614 | 3.05E-05 | 0.162 | TTTTCTTTTTTTAAAAAAAAA |
| *Glyma.08g201900* | *Glyma08g21610* | MP00200 | *Glyma.15g171900* | *Glyma15g18770* | 958 | 972 | - | 12.1343 | 3.06E-05 | 0.173 | ACTCGTGATGATTAT |
| *Glyma.08g201900* | *Glyma08g21610* | MP00200 | *Glyma.15g171900* | *Glyma15g18770* | 974 | 988 | - | 12.1343 | 3.06E-05 | 0.173 | ACTCGTGATGATTAT |
| *Glyma.08g201900* | *Glyma08g21610* | MP00200 | *Glyma.15g171900* | *Glyma15g18770* | 974 | 988 | - | 12.1343 | 3.06E-05 | 0.173 | ACTCGTGATGATTAT |
| *Glyma.20g160200* | *Glyma20g29960* | MP00449 | *Glyma.15g152100* | *Glyma15g16270* | 1962 | 1976 | + | 12.3939 | 3.06E-05 | 0.54 | TTTTGACTTTTCCTT |
| *Glyma.10g053500* | *Glyma10g06080* | MP00461 | *Glyma.12g081900* | *Glyma12g08720* | 572 | 592 | + | -0.939759 | 3.08E-05 | 0.162 | TTTATGTTAGTGATTTGGAAA |
| *Glyma.10g053500* | *Glyma10g06080* | MP00461 | *Glyma.12g081900* | *Glyma12g08720* | 572 | 592 | + | -0.939759 | 3.08E-05 | 0.162 | TTTATGTTAGTGATTTGGAAA |
| *Glyma.02g261700* | *Glyma02g42960* | MP00302 | *Glyma.07g080800* | *Glyma07g08810* | 1447 | 1467 | + | 10.6857 | 3.10E-05 | 0.149 | GTACGTGGCGACGAAGGTGGC |
| *Glyma.02g261700* | *Glyma02g42960* | MP00302 | *Glyma.07g080800* | *Glyma07g08810* | 1522 | 1542 | + | 10.6857 | 3.10E-05 | 0.149 | GTACGTGGCGACGAAGGTGGC |
| *Glyma.02g261700* | *Glyma02g42960* | MP00302 | *Glyma.07g069700* | *Glyma07g07580* | 1786 | 1806 | + | 10.6714 | 3.12E-05 | 0.15 | GCTGCCGGCTTCGACGGCGAC |
| *Glyma.10g053500* | *Glyma10g06080* | MP00461 | *Glyma.17g005600* | *Glyma17g00950* | 1258 | 1278 | - | -1.03614 | 3.16E-05 | 0.163 | TTTATATTTTAGAGTGGAACA |
| *Glyma.03g143100* | *Glyma03g29901* | MP00307 | *Glyma.07g080800* | *Glyma07g08810* | 516 | 526 | + | 11.8182 | 3.17E-05 | 1 | AATGTACTTAT |
| *Glyma.03g143100* | *Glyma03g29901* | MP00307 | *Glyma.07g080800* | *Glyma07g08810* | 591 | 601 | + | 11.8182 | 3.17E-05 | 1 | AATGTACTTAT |
| *Glyma.03g143100* | *Glyma03g29901* | MP00307 | *Glyma.07g080800* | *Glyma07g08810* | 1490 | 1500 | + | 11.8182 | 3.17E-05 | 1 | AATGTACTTAT |
| *Glyma.08g201900* | *Glyma08g21610* | MP00200 | *Glyma.15g171900* | *Glyma15g18770* | 396 | 410 | + | 12 | 3.27E-05 | 0.179 | TGAGATAATCATTAA |
| *Glyma.08g201900* | *Glyma08g21610* | MP00200 | *Glyma.15g171900* | *Glyma15g18770* | 412 | 426 | + | 12 | 3.27E-05 | 0.179 | TGAGATAATCATTAA |
| *Glyma.08g201900* | *Glyma08g21610* | MP00200 | *Glyma.15g171900* | *Glyma15g18770* | 412 | 426 | + | 12 | 3.27E-05 | 0.179 | TGAGATAATCATTAA |
| *Glyma.20g160200* | *Glyma20g29960* | MP00449 | *Glyma.05g000200* | *Glyma05g09400* | 1283 | 1297 | + | 12.3182 | 3.28E-05 | 0.54 | TTTTGTCTTTTGATT |
| *Glyma.20g160200* | *Glyma20g29960* | MP00449 | *Glyma.05g000200* | *Glyma05g09400* | 1283 | 1297 | + | 12.3182 | 3.28E-05 | 0.54 | TTTTGTCTTTTGATT |
| *Glyma.20g160200* | *Glyma20g29960* | MP00449 | *Glyma.05g000200* | *Glyma05g09400* | 1283 | 1297 | + | 12.3182 | 3.28E-05 | 0.54 | TTTTGTCTTTTGATT |
| *Glyma.20g160200* | *Glyma20g29960* | MP00449 | *Glyma.05g000200* | *Glyma05g09400* | 1283 | 1297 | + | 12.3182 | 3.28E-05 | 0.54 | TTTTGTCTTTTGATT |
| *Glyma.10g053500* | *Glyma10g06080* | MP00461 | *Glyma.14g043500* | *Glyma14g04780* | 1585 | 1605 | + | -1.22892 | 3.33E-05 | 0.163 | TATAGATTTTAGATGGGAGAA |
| *Glyma.10g053500* | *Glyma10g06080* | MP00461 | *Glyma.14g043500* | *Glyma14g04780* | 1867 | 1887 | + | -1.22892 | 3.33E-05 | 0.163 | TATAGATTTTAGATGGGAGAA |
| *Glyma.10g053500* | *Glyma10g06080* | MP00461 | *Glyma.06g068700* | *Glyma06g07230* | 53 | 73 | + | -1.28916 | 3.39E-05 | 0.163 | TTTACATGTTAGGATTAAAAA |
| *Glyma.13g219900* | *Glyma13g29160* | MP00639 | *Glyma.07g069700* | *Glyma07g07580* | 242 | 249 | + | 12.9286 | 3.40E-05 | 1 | TGGACCAC |
| *Glyma.13g219900* | *Glyma13g29160* | MP00639 | *Glyma.13g183800* | *Glyma13g25290* | 1032 | 1039 | - | 12.9286 | 3.40E-05 | 1 | TGGACCAC |
| *Glyma.10g053500* | *Glyma10g06080* | MP00461 | *Glyma.13g183800* | *Glyma13g25290* | 351 | 371 | - | -1.33735 | 3.43E-05 | 0.163 | ATTTTCTTTTTAACAGAACAA |
| *Glyma.10g053500* | *Glyma10g06080* | MP00461 | *Glyma.13g214600* | *Glyma13g28590* | 1175 | 1195 | - | -1.33735 | 3.43E-05 | 0.163 | TTTTTGCTTTATGGCTGAAAA |
| *Glyma.08g201900* | *Glyma08g21610* | MP00200 | *Glyma.20g172000* | *Glyma20g31200* | 374 | 388 | - | 11.8806 | 3.47E-05 | 0.179 | CATAGTAATAATTAA |
| *Glyma.08g201900* | *Glyma08g21610* | MP00200 | *Glyma.20g172000* | *Glyma20g31200* | 689 | 703 | - | 11.8806 | 3.47E-05 | 0.179 | CATAGTAATAATTAA |
| *Glyma.10g053500* | *Glyma10g06080* | MP00461 | *Glyma.08g156400* | *Glyma08g16620* | 1301 | 1321 | + | -1.39759 | 3.49E-05 | 0.163 | CTTTTTTTTTTTACAAAAAAT |
| *Glyma.02g261700* | *Glyma02g42960* | MP00302 | *Glyma.06g070100* | *Glyma06g07360* | 357 | 377 | - | 10.4286 | 3.51E-05 | 0.162 | CAATGAAATGGTGGTGGTGGA |
| *Glyma.02g261700* | *Glyma02g42960* | MP00302 | *Glyma.06g070100* | *Glyma06g07360* | 369 | 389 | - | 10.4286 | 3.51E-05 | 0.162 | CAATGAAATGGTGGTGGTGGA |
| *Glyma.02g261700* | *Glyma02g42960* | MP00302 | *Glyma.06g070100* | *Glyma06g07360* | 814 | 834 | - | 10.4286 | 3.51E-05 | 0.162 | CAATGAAATGGTGGTGGTGGA |
| *Glyma.02g261700* | *Glyma02g42960* | MP00302 | *Glyma.06g070100* | *Glyma06g07360* | 950 | 970 | - | 10.4286 | 3.51E-05 | 0.162 | CAATGAAATGGTGGTGGTGGA |
| *Glyma.03g143100* | *Glyma03g29901* | MP00307 | *Glyma.15g152100* | *Glyma15g16270* | 1834 | 1844 | + | 11.7727 | 3.53E-05 | 1 | TTTGTACGTAC |
| *Glyma.02g261700* | *Glyma02g42960* | MP00302 | *Glyma.06g152800* | *Glyma06g15820* | 1499 | 1519 | - | 10.4143 | 3.53E-05 | 0.162 | GGTGGTGGTGGTTGTCGTCGT |
| *Glyma.02g013900* | *Glyma02g01740* | MP00627 | *Glyma.11g248000* | *Glyma11g37320* | 1326 | 1335 | + | 11.9286 | 3.54E-05 | 1 | TTACCTAACG |
| *Glyma.02g013900* | *Glyma02g01740* | MP00627 | *Glyma.11g248000* | *Glyma11g37320* | 1611 | 1620 | + | 11.9286 | 3.54E-05 | 1 | TTACCTAACG |
| *Glyma.02g261700* | *Glyma02g42960* | MP00302 | *Glyma.13g162600* | *Glyma13g23150* | 525 | 545 | - | 10.4 | 3.56E-05 | 0.163 | GGAGGCGGGGCTGATGGAGTA |
| *Glyma.08g201900* | *Glyma08g21610* | MP00200 | *Glyma.15g171900* | *Glyma15g18770* | 400 | 414 | - | 11.8209 | 3.57E-05 | 0.179 | ACAATTAATGATTAT |
| *Glyma.08g201900* | *Glyma08g21610* | MP00200 | *Glyma.15g171900* | *Glyma15g18770* | 416 | 430 | - | 11.8209 | 3.57E-05 | 0.179 | ACAATTAATGATTAT |
| *Glyma.08g201900* | *Glyma08g21610* | MP00200 | *Glyma.15g171900* | *Glyma15g18770* | 416 | 430 | - | 11.8209 | 3.57E-05 | 0.179 | ACAATTAATGATTAT |
| *Glyma.08g097900* | *Glyma08g10350* | MP00006 | *Glyma.20g176300* | *Glyma20g31630* | 620 | 627 | + | 13.3252 | 3.60E-05 | 1 | GGCACCAC |
| *Glyma.10g053500* | *Glyma10g06080* | MP00461 | *Glyma.13g162600* | *Glyma13g23150* | 1232 | 1252 | - | -1.55422 | 3.64E-05 | 0.163 | TTTTTTTTTTTTTTAGAAAAA |
| *Glyma.08g201900* | *Glyma08g21610* | MP00200 | *Glyma.05g012300* | *Glyma05g08060* | 370 | 384 | + | 11.7761 | 3.65E-05 | 0.179 | AATGCAAATGATTAC |
| *Glyma.10g053500* | *Glyma10g06080* | MP00461 | *Glyma.12g081900* | *Glyma12g08720* | 1373 | 1393 | - | -1.71084 | 3.79E-05 | 0.163 | TTTATTATTATAGTAAAAGAA |
| *Glyma.10g053500* | *Glyma10g06080* | MP00461 | *Glyma.12g081900* | *Glyma12g08720* | 1373 | 1393 | - | -1.71084 | 3.79E-05 | 0.163 | TTTATTATTATAGTAAAAGAA |
| *Glyma.20g160200* | *Glyma20g29960* | MP00449 | *Glyma.07g081700* | *Glyma07g08950* | 751 | 765 | + | 12.1515 | 3.80E-05 | 0.546 | AGTTGACCCTTTGAA |
| *Glyma.02g261700* | *Glyma02g42960* | MP00302 | *Glyma.02g076000* | *Glyma02g08350* | 1978 | 1998 | - | 10.2429 | 3.84E-05 | 0.169 | TGAAGAGTGAACGGTGGTGAA |
| *Glyma.10g053500* | *Glyma10g06080* | MP00461 | *Glyma.13g162600* | *Glyma13g23150* | 1229 | 1249 | - | -1.75904 | 3.84E-05 | 0.163 | TTTTTTTTTTTAGAAAAAAAA |
| *Glyma.10g053500* | *Glyma10g06080* | MP00461 | *Glyma.08g156400* | *Glyma08g16620* | 1704 | 1724 | - | -1.75904 | 3.84E-05 | 0.163 | TATATATTTTTAACCGTAAAA |
| *Glyma.10g053500* | *Glyma10g06080* | MP00461 | *Glyma.12g047400* | *Glyma12g05140* | 1208 | 1228 | + | -1.81928 | 3.90E-05 | 0.163 | TTTCTAGTTTTTACCGAAGAA |
| *Glyma.10g053500* | *Glyma10g06080* | MP00461 | *Glyma.12g047400* | *Glyma12g05140* | 1910 | 1930 | + | -1.81928 | 3.90E-05 | 0.163 | TTTCTAGTTTTTACCGAAGAA |
| *Glyma.10g053500* | *Glyma10g06080* | MP00461 | *Glyma.12g047400* | *Glyma12g05140* | 1910 | 1930 | + | -1.81928 | 3.90E-05 | 0.163 | TTTCTAGTTTTTACCGAAGAA |
| *Glyma.13g187500* | *Glyma13g25716* | MP00490 | *Glyma.03g242600* | *Glyma03g40250* | 1361 | 1371 | + | 12.1061 | 3.94E-05 | 0.844 | ATAACCAAATT |
| *Glyma.10g053500* | *Glyma10g06080* | MP00461 | *Glyma.20g013500* | *Glyma20g01680* | 1152 | 1172 | - | -1.85542 | 3.94E-05 | 0.163 | TTTTTGATTTTGTGAAAGAAA |
| *Glyma.02g261700* | *Glyma02g42960* | MP00302 | *Glyma.10g181900* | *Glyma10g32520* | 1160 | 1180 | - | 10.1714 | 3.97E-05 | 0.174 | TCAAAAGAAGGAGGTGGTGGG |
| *Glyma.08g201900* | *Glyma08g21610* | MP00200 | *Glyma.07g080800* | *Glyma07g08810* | 419 | 433 | - | 11.597 | 3.98E-05 | 0.179 | TATAATAATTATTAA |
| *Glyma.08g201900* | *Glyma08g21610* | MP00200 | *Glyma.06g152800* | *Glyma06g15820* | 732 | 746 | - | 11.5672 | 4.04E-05 | 0.179 | TTATATAATTATTAC |
| *Glyma.08g201900* | *Glyma08g21610* | MP00200 | *Glyma.06g152800* | *Glyma06g15820* | 1380 | 1394 | - | 11.5672 | 4.04E-05 | 0.179 | TTATATAATTATTAC |
| *Glyma.13g187500* | *Glyma13g25716* | MP00490 | *Glyma.17g005600* | *Glyma17g00950* | 543 | 553 | + | 12.0909 | 4.04E-05 | 0.844 | CTAACTGAAAA |
| *Glyma.02g261700* | *Glyma02g42960* | MP00302 | *Glyma.08g156400* | *Glyma08g16620* | 2058 | 2078 | - | 10.1143 | 4.08E-05 | 0.178 | TGTTGCGGTGACAACGGTGCT |
| *Glyma.10g053500* | *Glyma10g06080* | MP00461 | *Glyma.08g156400* | *Glyma08g16620* | 179 | 199 | + | -2.0241 | 4.12E-05 | 0.163 | ATTATGTTTTAAGTGTGTAAA |
| *Glyma.08g201900* | *Glyma08g21610* | MP00200 | *Glyma.02g076000* | *Glyma02g08350* | 668 | 682 | - | 11.5075 | 4.15E-05 | 0.18 | TATTACAATAATTAC |
| *Glyma.08g201900* | *Glyma08g21610* | MP00200 | *Glyma.02g076000* | *Glyma02g08350* | 848 | 862 | - | 11.5075 | 4.15E-05 | 0.18 | TATTACAATAATTAC |
| *Glyma.10g053500* | *Glyma10g06080* | MP00461 | *Glyma.10g181900* | *Glyma10g32520* | 288 | 308 | + | -2.13253 | 4.24E-05 | 0.163 | TTCAAGTTTAATGGGAAAAAA |
| *Glyma.02g261700* | *Glyma02g42960* | MP00302 | *Glyma.05g012300* | *Glyma05g08060* | 49 | 69 | - | 10 | 4.30E-05 | 0.18 | TCGGATGCAGGTTGTGGTGGA |
| *Glyma.02g261700* | *Glyma02g42960* | MP00302 | *Glyma.05g012300* | *Glyma05g08060* | 50 | 70 | - | 10 | 4.30E-05 | 0.18 | TCGGATGCAGGTTGTGGTGGA |
| *Glyma.02g261700* | *Glyma02g42960* | MP00302 | *Glyma.05g012300* | *Glyma05g08060* | 201 | 221 | - | 10 | 4.30E-05 | 0.18 | TCGGATGCAGGTTGTGGTGGA |
| *Glyma.02g261700* | *Glyma02g42960* | MP00302 | *Glyma.05g012300* | *Glyma05g08060* | 1222 | 1242 | - | 10 | 4.30E-05 | 0.18 | TCGGATGCAGGTTGTGGTGGA |
| *Glyma.02g261700* | *Glyma02g42960* | MP00302 | *Glyma.17g005600* | *Glyma17g00950* | 551 | 571 | + | 10 | 4.30E-05 | 0.18 | AAATGAATCGGTGGTGGTGGT |
| *Glyma.10g053500* | *Glyma10g06080* | MP00461 | *Glyma.05g062200* | *Glyma05g04940* | 1300 | 1320 | - | -2.20482 | 4.32E-05 | 0.163 | AAATTGGTTTTGGTCGGGAAT |
| *Glyma.10g053500* | *Glyma10g06080* | MP00461 | *Glyma.20g172000* | *Glyma20g31200* | 1011 | 1031 | + | -2.25301 | 4.38E-05 | 0.163 | AATGCAATTTTAGCAAGAGAT |
| *Glyma.10g053500* | *Glyma10g06080* | MP00461 | *Glyma.20g172000* | *Glyma20g31200* | 1011 | 1031 | + | -2.25301 | 4.38E-05 | 0.163 | AATGCAATTTTAGCAAGAGAT |
| *Glyma.10g053500* | *Glyma10g06080* | MP00461 | *Glyma.20g172000* | *Glyma20g31200* | 1200 | 1220 | + | -2.25301 | 4.38E-05 | 0.163 | AATGCAATTTTAGCAAGAGAT |
| *Glyma.08g201900* | *Glyma08g21610* | MP00200 | *Glyma.07g069700* | *Glyma07g07580* | 1221 | 1235 | + | 11.3731 | 4.42E-05 | 0.182 | GATAGTAATCATAAT |
| *Glyma.08g201900* | *Glyma08g21610* | MP00200 | *Glyma.07g069700* | *Glyma07g07580* | 1747 | 1761 | + | 11.3731 | 4.42E-05 | 0.182 | GATAGTAATCATAAT |
| *Glyma.02g013900* | *Glyma02g01740* | MP00627 | *Glyma.05g056700* | *Glyma05g04330* | 898 | 907 | + | 11.5204 | 4.45E-05 | 1 | TTACCTAACT |
| *Glyma.02g013900* | *Glyma02g01740* | MP00627 | *Glyma.05g056700* | *Glyma05g04330* | 906 | 915 | + | 11.5204 | 4.45E-05 | 1 | TTACCTAACT |
| *Glyma.02g013900* | *Glyma02g01740* | MP00627 | *Glyma.05g056700* | *Glyma05g04330* | 906 | 915 | + | 11.5204 | 4.45E-05 | 1 | TTACCTAACT |
| *Glyma.02g013900* | *Glyma02g01740* | MP00627 | *Glyma.05g056700* | *Glyma05g04330* | 931 | 940 | + | 11.5204 | 4.45E-05 | 1 | TTACCTAACT |
| *Glyma.02g239600* | *Glyma02g40650* | MP00033 | *Glyma.20g176300* | *Glyma20g31630* | 1498 | 1506 | + | 11.19 | 4.47E-05 | 1 | CTTGTCGGA |
| *Glyma.20g160200* | *Glyma20g29960* | MP00449 | *Glyma.03g260300* | *Glyma03g42140* | 1226 | 1240 | - | 11.9545 | 4.47E-05 | 0.58 | CATTGTCTTTTCATA |
| *Glyma.08g201900* | *Glyma08g21610* | MP00200 | *Glyma.13g214600* | *Glyma13g28590* | 277 | 291 | - | 11.3284 | 4.51E-05 | 0.182 | GAAAGTAATAATGGA |
| *Glyma.10g053500* | *Glyma10g06080* | MP00461 | *Glyma.12g047400* | *Glyma12g05140* | 455 | 475 | + | -2.39759 | 4.54E-05 | 0.163 | GATACTTTTTTTATTGGAGAA |
| *Glyma.10g053500* | *Glyma10g06080* | MP00461 | *Glyma.12g047400* | *Glyma12g05140* | 455 | 475 | + | -2.39759 | 4.54E-05 | 0.163 | GATACTTTTTTTATTGGAGAA |
| *Glyma.02g013900* | *Glyma02g01740* | MP00627 | *Glyma.05g246500* | *Glyma05g33790* | 1835 | 1844 | - | 11.4694 | 4.56E-05 | 1 | AAACCTACCT |
| *Glyma.02g013900* | *Glyma02g01740* | MP00627 | *Glyma.05g246500* | *Glyma05g33790* | 1961 | 1970 | - | 11.4694 | 4.56E-05 | 1 | AAACCTACCT |
| *Glyma.08g201900* | *Glyma08g21610* | MP00200 | *Glyma.05g056700* | *Glyma05g04330* | 348 | 362 | - | 11.2985 | 4.58E-05 | 0.182 | TATAATAATGATAAA |
| *Glyma.08g201900* | *Glyma08g21610* | MP00200 | *Glyma.05g056700* | *Glyma05g04330* | 356 | 370 | - | 11.2985 | 4.58E-05 | 0.182 | TATAATAATGATAAA |
| *Glyma.08g201900* | *Glyma08g21610* | MP00200 | *Glyma.05g056700* | *Glyma05g04330* | 356 | 370 | - | 11.2985 | 4.58E-05 | 0.182 | TATAATAATGATAAA |
| *Glyma.08g201900* | *Glyma08g21610* | MP00200 | *Glyma.05g056700* | *Glyma05g04330* | 381 | 395 | - | 11.2985 | 4.58E-05 | 0.182 | TATAATAATGATAAA |
| *Glyma.08g201900* | *Glyma08g21610* | MP00200 | *Glyma.02g076000* | *Glyma02g08350* | 1617 | 1631 | + | 11.2985 | 4.58E-05 | 0.182 | AACACTAATCATGAT |
| *Glyma.08g201900* | *Glyma08g21610* | MP00200 | *Glyma.02g076000* | *Glyma02g08350* | 1797 | 1811 | + | 11.2985 | 4.58E-05 | 0.182 | AACACTAATCATGAT |
| *Glyma.13g187500* | *Glyma13g25716* | MP00490 | *Glyma.05g062200* | *Glyma05g04940* | 777 | 787 | + | 12 | 4.60E-05 | 0.861 | TTAACTGAATT |
| *Glyma.02g261700* | *Glyma02g42960* | MP00302 | *Glyma.08g345900* | *Glyma08g45990* | 93 | 113 | - | 9.85714 | 4.60E-05 | 0.186 | TATGCTGAAAGTGGAGGTTGA |
| *Glyma.02g261700* | *Glyma02g42960* | MP00302 | *Glyma.03g242600* | *Glyma03g40250* | 2063 | 2083 | - | 9.85714 | 4.60E-05 | 0.186 | GCCAGTGAATGCATTGGTGGC |
| *Glyma.02g261700* | *Glyma02g42960* | MP00302 | *Glyma.20g176300* | *Glyma20g31630* | 1909 | 1929 | - | 9.81429 | 4.70E-05 | 0.189 | TGCGGTGATGTGGTCGGAGCA |
| *Glyma.10g053500* | *Glyma10g06080* | MP00461 | *Glyma.05g246500* | *Glyma05g33790* | 1366 | 1386 | + | -2.55422 | 4.73E-05 | 0.165 | ATTTTAATTTTGTTAGAAAAA |
| *Glyma.10g053500* | *Glyma10g06080* | MP00461 | *Glyma.05g246500* | *Glyma05g33790* | 1492 | 1512 | + | -2.55422 | 4.73E-05 | 0.165 | ATTTTAATTTTGTTAGAAAAA |
| *Glyma.10g053500* | *Glyma10g06080* | MP00461 | *Glyma.07g069700* | *Glyma07g07580* | 2077 | 2097 | + | -2.56627 | 4.75E-05 | 0.165 | TTTATGGTTATGGCGTTTAAT |
| *Glyma.02g261700* | *Glyma02g42960* | MP00302 | *Glyma.20g176300* | *Glyma20g31630* | 1283 | 1303 | + | 9.68571 | 4.99E-05 | 0.197 | TGAGATTCCACCGGCGGTGCT |
| *Glyma.02g013900* | *Glyma02g01740* | MP00627 | *Glyma.20g013500* | *Glyma20g01680* | 466 | 475 | - | 11.3469 | 5.03E-05 | 1 | CCACCTACCT |
| *Glyma.02g261700* | *Glyma02g42960* | MP00302 | *Glyma.06g152800* | *Glyma06g15820* | 101 | 121 | + | 9.65714 | 5.05E-05 | 0.198 | GTGGTCTCTGGTGGTGGAGGG |
| *Glyma.02g261700* | *Glyma02g42960* | MP00302 | *Glyma.06g152800* | *Glyma06g15820* | 749 | 769 | + | 9.65714 | 5.05E-05 | 0.198 | GTGGTCTCTGGTGGTGGAGGG |
| *Glyma.02g239600* | *Glyma02g40650* | MP00033 | *Glyma.20g176300* | *Glyma20g31630* | 1489 | 1497 | + | 11.17 | 5.14E-05 | 1 | CATGTCGGC |
| *Glyma.10g053500* | *Glyma10g06080* | MP00461 | *Glyma.10g014400* | *Glyma10g01770* | 374 | 394 | - | -2.90361 | 5.19E-05 | 0.168 | TTGATAATTTAGCCAGGGAAA |
| *Glyma.10g053500* | *Glyma10g06080* | MP00461 | *Glyma.10g014400* | *Glyma10g01770* | 382 | 402 | - | -2.90361 | 5.19E-05 | 0.168 | TTGATAATTTAGCCAGGGAAA |
| *Glyma.13g219900* | *Glyma13g29160* | MP00639 | *Glyma.06g070100* | *Glyma06g07360* | 188 | 195 | + | 11.8163 | 5.20E-05 | 1 | GGGACCAG |
| *Glyma.13g219900* | *Glyma13g29160* | MP00639 | *Glyma.06g070100* | *Glyma06g07360* | 324 | 331 | + | 11.8163 | 5.20E-05 | 1 | GGGACCAG |
| *Glyma.10g053500* | *Glyma10g06080* | MP00461 | *Glyma.07g080800* | *Glyma07g08810* | 1838 | 1858 | + | -2.93976 | 5.23E-05 | 0.168 | TTTTTTTTTTTTTTGTAAAAA |
| *Glyma.10g053500* | *Glyma10g06080* | MP00461 | *Glyma.07g080800* | *Glyma07g08810* | 1913 | 1933 | + | -2.93976 | 5.23E-05 | 0.168 | TTTTTTTTTTTTTTGTAAAAA |
| *Glyma.08g201900* | *Glyma08g21610* | MP00200 | *Glyma.15g171900* | *Glyma15g18770* | 795 | 809 | - | 11 | 5.25E-05 | 0.182 | ATTGTCAATGATGAC |
| *Glyma.08g201900* | *Glyma08g21610* | MP00200 | *Glyma.15g171900* | *Glyma15g18770* | 811 | 825 | - | 11 | 5.25E-05 | 0.182 | ATTGTCAATGATGAC |
| *Glyma.08g201900* | *Glyma08g21610* | MP00200 | *Glyma.15g171900* | *Glyma15g18770* | 811 | 825 | - | 11 | 5.25E-05 | 0.182 | ATTGTCAATGATGAC |
| *Glyma.08g201900* | *Glyma08g21610* | MP00200 | *Glyma.12g047400* | *Glyma12g05140* | 58 | 72 | - | 10.9701 | 5.32E-05 | 0.182 | ATTTATAATAATTAG |
| *Glyma.08g201900* | *Glyma08g21610* | MP00200 | *Glyma.05g246500* | *Glyma05g33790* | 114 | 128 | - | 10.9701 | 5.32E-05 | 0.182 | AATAATGATTATTAT |
| *Glyma.08g201900* | *Glyma08g21610* | MP00200 | *Glyma.05g246500* | *Glyma05g33790* | 240 | 254 | - | 10.9701 | 5.32E-05 | 0.182 | AATAATGATTATTAT |
| *Glyma.08g201900* | *Glyma08g21610* | MP00200 | *Glyma.12g047400* | *Glyma12g05140* | 760 | 774 | - | 10.9701 | 5.32E-05 | 0.182 | ATTTATAATAATTAG |
| *Glyma.08g201900* | *Glyma08g21610* | MP00200 | *Glyma.12g047400* | *Glyma12g05140* | 760 | 774 | - | 10.9701 | 5.32E-05 | 0.182 | ATTTATAATAATTAG |
| *Glyma.10g053500* | *Glyma10g06080* | MP00461 | *Glyma.07g069700* | *Glyma07g07580* | 660 | 680 | + | -3.03614 | 5.37E-05 | 0.168 | TTAACAATTTTCATTAAAAAA |
| *Glyma.10g053500* | *Glyma10g06080* | MP00461 | *Glyma.07g069700* | *Glyma07g07580* | 1186 | 1206 | + | -3.03614 | 5.37E-05 | 0.168 | TTAACAATTTTCATTAAAAAA |
| *Glyma.08g201900* | *Glyma08g21610* | MP00200 | *Glyma.07g080800* | *Glyma07g08810* | 950 | 964 | + | 10.9403 | 5.39E-05 | 0.182 | AAAATTAATTATTAC |
| *Glyma.08g201900* | *Glyma08g21610* | MP00200 | *Glyma.07g080800* | *Glyma07g08810* | 1025 | 1039 | + | 10.9403 | 5.39E-05 | 0.182 | AAAATTAATTATTAC |
| *Glyma.08g201900* | *Glyma08g21610* | MP00200 | *Glyma.10g181900* | *Glyma10g32520* | 1668 | 1682 | + | 10.9403 | 5.39E-05 | 0.182 | AACAATGATCATTAT |
| *Glyma.08g201900* | *Glyma08g21610* | MP00200 | *Glyma.07g080800* | *Glyma07g08810* | 1924 | 1938 | + | 10.9403 | 5.39E-05 | 0.182 | AAAATTAATTATTAC |
| *Glyma.10g053500* | *Glyma10g06080* | MP00461 | *Glyma.02g076000* | *Glyma02g08350* | 96 | 116 | - | -3.10843 | 5.47E-05 | 0.168 | TTTTCTATTATTGTTGGAGAT |
| *Glyma.02g239600* | *Glyma02g40650* | MP00033 | *Glyma.13g364900* | *Glyma13g44170* | 597 | 605 | - | 11.14 | 5.47E-05 | 1 | CTTGTCGGT |
| *Glyma.02g239600* | *Glyma02g40650* | MP00033 | *Glyma.13g364900* | *Glyma13g44170* | 636 | 644 | - | 11.14 | 5.47E-05 | 1 | CTTGTCGGT |
| *Glyma.03g143100* | *Glyma03g29901* | MP00307 | *Glyma.15g152100* | *Glyma15g16270* | 1630 | 1640 | - | 11.4697 | 5.48E-05 | 1 | TTTGTACTGAA |
| *Glyma.10g053500* | *Glyma10g06080* | MP00461 | *Glyma.05g056700* | *Glyma05g04330* | 1038 | 1058 | - | -3.22892 | 5.64E-05 | 0.168 | ATTGCGGTAATAATGGGAGAT |
| *Glyma.10g053500* | *Glyma10g06080* | MP00461 | *Glyma.05g056700* | *Glyma05g04330* | 1046 | 1066 | - | -3.22892 | 5.64E-05 | 0.168 | ATTGCGGTAATAATGGGAGAT |
| *Glyma.10g053500* | *Glyma10g06080* | MP00461 | *Glyma.05g056700* | *Glyma05g04330* | 1046 | 1066 | - | -3.22892 | 5.64E-05 | 0.168 | ATTGCGGTAATAATGGGAGAT |
| *Glyma.10g053500* | *Glyma10g06080* | MP00461 | *Glyma.05g056700* | *Glyma05g04330* | 1071 | 1091 | - | -3.22892 | 5.64E-05 | 0.168 | ATTGCGGTAATAATGGGAGAT |
| *Glyma.13g187500* | *Glyma13g25716* | MP00490 | *Glyma.06g070100* | *Glyma06g07360* | 1468 | 1478 | - | 11.7727 | 5.68E-05 | 0.92 | ATAACCAAATA |
| *Glyma.13g187500* | *Glyma13g25716* | MP00490 | *Glyma.06g070100* | *Glyma06g07360* | 1480 | 1490 | - | 11.7727 | 5.68E-05 | 0.92 | ATAACCAAATA |
| *Glyma.13g187500* | *Glyma13g25716* | MP00490 | *Glyma.06g070100* | *Glyma06g07360* | 1925 | 1935 | - | 11.7727 | 5.68E-05 | 0.92 | ATAACCAAATA |
| *Glyma.13g187500* | *Glyma13g25716* | MP00490 | *Glyma.06g070100* | *Glyma06g07360* | 2061 | 2071 | - | 11.7727 | 5.68E-05 | 0.92 | ATAACCAAATA |
| *Glyma.13g187500* | *Glyma13g25716* | MP00490 | *Glyma.03g242600* | *Glyma03g40250* | 4 | 14 | + | 11.7727 | 5.68E-05 | 0.92 | ATAACCAAATA |
| *Glyma.08g201900* | *Glyma08g21610* | MP00200 | *Glyma.20g172000* | *Glyma20g31200* | 356 | 370 | + | 10.8209 | 5.69E-05 | 0.184 | GTGGGAAATGATTAC |
| *Glyma.08g201900* | *Glyma08g21610* | MP00200 | *Glyma.20g172000* | *Glyma20g31200* | 671 | 685 | + | 10.8209 | 5.69E-05 | 0.184 | GTGGGAAATGATTAC |
| *Glyma.10g053500* | *Glyma10g06080* | MP00461 | *Glyma.08g156400* | *Glyma08g16620* | 1211 | 1231 | - | -3.26506 | 5.69E-05 | 0.168 | TTTACATTGATGATAGAGAAT |
| *Glyma.10g053500* | *Glyma10g06080* | MP00461 | *Glyma.15g152100* | *Glyma15g16270* | 354 | 374 | + | -3.27711 | 5.71E-05 | 0.168 | TATAAATTTTTGAGGAAGAAT |
| *Glyma.08g201900* | *Glyma08g21610* | MP00200 | *Glyma.07g080800* | *Glyma07g08810* | 418 | 432 | + | 10.806 | 5.73E-05 | 0.184 | TTTAATAATTATTAT |
| *Glyma.08g201900* | *Glyma08g21610* | MP00200 | *Glyma.20g013500* | *Glyma20g01680* | 433 | 447 | + | 10.806 | 5.73E-05 | 0.184 | TCCCGTAATGATGAT |
| *Glyma.03g143100* | *Glyma03g29901* | MP00307 | *Glyma.07g069700* | *Glyma07g07580* | 59 | 69 | + | 11.4242 | 5.85E-05 | 1 | CATGTACGGTA |
| *Glyma.02g261700* | *Glyma02g42960* | MP00302 | *Glyma.13g162600* | *Glyma13g23150* | 1566 | 1586 | - | 9.32857 | 5.88E-05 | 0.213 | ATGAGTGATACTGGTGGCGGT |
| *Glyma.20g160200* | *Glyma20g29960* | MP00449 | *Glyma.20g172000* | *Glyma20g31200* | 308 | 322 | - | 11.6061 | 5.89E-05 | 0.603 | AATTGTCTTTTGCTA |
| *Glyma.20g160200* | *Glyma20g29960* | MP00449 | *Glyma.20g172000* | *Glyma20g31200* | 623 | 637 | - | 11.6061 | 5.89E-05 | 0.603 | AATTGTCTTTTGCTA |
| *Glyma.10g053500* | *Glyma10g06080* | MP00461 | *Glyma.17g005600* | *Glyma17g00950* | 314 | 334 | + | -3.42169 | 5.92E-05 | 0.168 | TTTTTGCGTGTGGAGGGAGAA |
| *Glyma.02g261700* | *Glyma02g42960* | MP00302 | *Glyma.07g069700* | *Glyma07g07580* | 1456 | 1476 | - | 9.28571 | 5.99E-05 | 0.217 | GATGGTGGAGCCGTTCGTGAA |
| *Glyma.02g261700* | *Glyma02g42960* | MP00302 | *Glyma.07g069700* | *Glyma07g07580* | 1982 | 2002 | - | 9.28571 | 5.99E-05 | 0.217 | GATGGTGGAGCCGTTCGTGAA |
| *Glyma.08g201900* | *Glyma08g21610* | MP00200 | *Glyma.06g152800* | *Glyma06g15820* | 802 | 816 | + | 10.7015 | 6.00E-05 | 0.185 | ATTAATAATGATAAA |
| *Glyma.08g201900* | *Glyma08g21610* | MP00200 | *Glyma.06g152800* | *Glyma06g15820* | 1450 | 1464 | + | 10.7015 | 6.00E-05 | 0.185 | ATTAATAATGATAAA |
| *Glyma.13g219900* | *Glyma13g29160* | MP00639 | *Glyma.14g043500* | *Glyma14g04780* | 55 | 62 | - | 11.7041 | 6.02E-05 | 1 | GGGCCCAC |
| *Glyma.13g219900* | *Glyma13g29160* | MP00639 | *Glyma.11g248000* | *Glyma11g37320* | 1955 | 1962 | - | 11.7041 | 6.02E-05 | 1 | GGGCCCAC |
| *Glyma.08g201900* | *Glyma08g21610* | MP00200 | *Glyma.13g162600* | *Glyma13g23150* | 500 | 514 | + | 10.6866 | 6.04E-05 | 0.185 | GACACTGATGATGAT |
| *Glyma.08g201900* | *Glyma08g21610* | MP00200 | *Glyma.10g181900* | *Glyma10g32520* | 1669 | 1683 | - | 10.6716 | 6.08E-05 | 0.185 | GATAATGATCATTGT |
| *Glyma.10g053500* | *Glyma10g06080* | MP00461 | *Glyma.12g081900* | *Glyma12g08720* | 336 | 356 | - | -3.53012 | 6.09E-05 | 0.168 | TATGAGTTTATGGCCAAAAGA |
| *Glyma.10g053500* | *Glyma10g06080* | MP00461 | *Glyma.12g081900* | *Glyma12g08720* | 336 | 356 | - | -3.53012 | 6.09E-05 | 0.168 | TATGAGTTTATGGCCAAAAGA |
| *Glyma.20g160200* | *Glyma20g29960* | MP00449 | *Glyma.16g146500* | *Glyma16g26210* | 1872 | 1886 | - | 11.5606 | 6.10E-05 | 0.603 | GTTTGGCTTTTCTAA |
| *Glyma.02g261700* | *Glyma02g42960* | MP00302 | *Glyma.14g043500* | *Glyma14g04780* | 1752 | 1772 | + | 9.22857 | 6.15E-05 | 0.221 | GTTGATGAAATTGATGGTGAA |
| *Glyma.02g261700* | *Glyma02g42960* | MP00302 | *Glyma.14g043500* | *Glyma14g04780* | 2034 | 2054 | + | 9.22857 | 6.15E-05 | 0.221 | GTTGATGAAATTGATGGTGAA |
| *Glyma.10g053500* | *Glyma10g06080* | MP00461 | *Glyma.13g183800* | *Glyma13g25290* | 1573 | 1593 | + | -3.57831 | 6.17E-05 | 0.168 | TAATTGTTTATGGCTGAAGAC |
| *Glyma.10g053500* | *Glyma10g06080* | MP00461 | *Glyma.07g080800* | *Glyma07g08810* | 220 | 240 | - | -3.59036 | 6.18E-05 | 0.168 | TTGATAATTATTATAGAAAAT |
| *Glyma.10g053500* | *Glyma10g06080* | MP00461 | *Glyma.20g013500* | *Glyma20g01680* | 1458 | 1478 | - | -3.59036 | 6.18E-05 | 0.168 | TTTCTATTTTCGGTGTAAATA |
| *Glyma.10g053500* | *Glyma10g06080* | MP00461 | *Glyma.05g056700* | *Glyma05g04330* | 1533 | 1553 | - | -3.61446 | 6.22E-05 | 0.168 | TTGACGATATTTGTGGAGATG |
| *Glyma.10g053500* | *Glyma10g06080* | MP00461 | *Glyma.05g056700* | *Glyma05g04330* | 1541 | 1561 | - | -3.61446 | 6.22E-05 | 0.168 | TTGACGATATTTGTGGAGATG |
| *Glyma.10g053500* | *Glyma10g06080* | MP00461 | *Glyma.05g056700* | *Glyma05g04330* | 1541 | 1561 | - | -3.61446 | 6.22E-05 | 0.168 | TTGACGATATTTGTGGAGATG |
| *Glyma.10g053500* | *Glyma10g06080* | MP00461 | *Glyma.05g056700* | *Glyma05g04330* | 1566 | 1586 | - | -3.61446 | 6.22E-05 | 0.168 | TTGACGATATTTGTGGAGATG |
| *Glyma.08g201900* | *Glyma08g21610* | MP00200 | *Glyma.09g273900* | *Glyma09g41040* | 1583 | 1597 | + | 10.6119 | 6.24E-05 | 0.185 | ATATACAATCATGAG |
| *Glyma.10g053500* | *Glyma10g06080* | MP00461 | *Glyma.16g018300* | *Glyma16g02090* | 885 | 905 | - | -3.66265 | 6.30E-05 | 0.168 | CAAATTGTTTTGGTTCAAAAA |
| *Glyma.10g053500* | *Glyma10g06080* | MP00461 | *Glyma.10g181900* | *Glyma10g32520* | 286 | 306 | + | -3.6988 | 6.36E-05 | 0.168 | GATTCAAGTTTAATGGGAAAA |
| *Glyma.10g053500* | *Glyma10g06080* | MP00461 | *Glyma.10g014400* | *Glyma10g01770* | 1930 | 1950 | + | -3.72289 | 6.40E-05 | 0.168 | TTTCCGTGTTTGGTTTGGGAA |
| *Glyma.10g053500* | *Glyma10g06080* | MP00461 | *Glyma.10g014400* | *Glyma10g01770* | 1938 | 1958 | + | -3.72289 | 6.40E-05 | 0.168 | TTTCCGTGTTTGGTTTGGGAA |
| *Glyma.03g143100* | *Glyma03g29901* | MP00307 | *Glyma.10g014400* | *Glyma10g01770* | 1186 | 1196 | - | 11.3485 | 6.46E-05 | 1 | ATGGTACGGTT |
| *Glyma.03g143100* | *Glyma03g29901* | MP00307 | *Glyma.10g014400* | *Glyma10g01770* | 1194 | 1204 | - | 11.3485 | 6.46E-05 | 1 | ATGGTACGGTT |
| *Glyma.20g160200* | *Glyma20g29960* | MP00449 | *Glyma.05g000200* | *Glyma05g09400* | 693 | 707 | - | 11.4848 | 6.46E-05 | 0.603 | ATGTGACTTTTTAAT |
| *Glyma.20g160200* | *Glyma20g29960* | MP00449 | *Glyma.05g000200* | *Glyma05g09400* | 693 | 707 | - | 11.4848 | 6.46E-05 | 0.603 | ATGTGACTTTTTAAT |
| *Glyma.20g160200* | *Glyma20g29960* | MP00449 | *Glyma.05g000200* | *Glyma05g09400* | 693 | 707 | - | 11.4848 | 6.46E-05 | 0.603 | ATGTGACTTTTTAAT |
| *Glyma.20g160200* | *Glyma20g29960* | MP00449 | *Glyma.05g000200* | *Glyma05g09400* | 693 | 707 | - | 11.4848 | 6.46E-05 | 0.603 | ATGTGACTTTTTAAT |
| *Glyma.10g053500* | *Glyma10g06080* | MP00461 | *Glyma.06g152800* | *Glyma06g15820* | 1747 | 1767 | + | -3.77108 | 6.47E-05 | 0.169 | TTAACTTTTATCACGAAAAAG |
| *Glyma.02g261700* | *Glyma02g42960* | MP00302 | *Glyma.12g047400* | *Glyma12g05140* | 1452 | 1472 | - | 9.11429 | 6.48E-05 | 0.227 | GGCGTTAGAAGAGTTGGTTGA |
| *Glyma.08g201900* | *Glyma08g21610* | MP00200 | *Glyma.17g005600* | *Glyma17g00950* | 1295 | 1309 | - | 10.5224 | 6.49E-05 | 0.185 | AATGAAGATGATGAC |
| *Glyma.10g053500* | *Glyma10g06080* | MP00461 | *Glyma.13g214600* | *Glyma13g28590* | 1174 | 1194 | - | -3.79518 | 6.51E-05 | 0.169 | TTTTGCTTTATGGCTGAAAAC |
| *Glyma.08g201900* | *Glyma08g21610* | MP00200 | *Glyma.02g076000* | *Glyma02g08350* | 664 | 678 | + | 10.4925 | 6.57E-05 | 0.185 | ATTTGTAATTATTGT |
| *Glyma.08g201900* | *Glyma08g21610* | MP00200 | *Glyma.02g076000* | *Glyma02g08350* | 844 | 858 | + | 10.4925 | 6.57E-05 | 0.185 | ATTTGTAATTATTGT |
| *Glyma.08g201900* | *Glyma08g21610* | MP00200 | *Glyma.14g043500* | *Glyma14g04780* | 206 | 220 | - | 10.4776 | 6.61E-05 | 0.185 | TCCTGCAATGATTAT |
| *Glyma.08g201900* | *Glyma08g21610* | MP00200 | *Glyma.14g043500* | *Glyma14g04780* | 574 | 588 | - | 10.4776 | 6.61E-05 | 0.185 | TCCTGCAATGATTAT |
| *Glyma.10g053500* | *Glyma10g06080* | MP00461 | *Glyma.08g345900* | *Glyma08g45990* | 1496 | 1516 | - | -3.86747 | 6.64E-05 | 0.169 | TTTTTGTTAGTTGTTTAAAAA |
| *Glyma.10g053500* | *Glyma10g06080* | MP00461 | *Glyma.08g345900* | *Glyma08g45990* | 1706 | 1726 | - | -3.86747 | 6.64E-05 | 0.169 | TTTTTGTTAGTTGTTTAAAAA |
| *Glyma.20g160200* | *Glyma20g29960* | MP00449 | *Glyma.02g076000* | *Glyma02g08350* | 1651 | 1665 | + | 11.4394 | 6.69E-05 | 0.603 | TTGTGTCCTTTGATT |
| *Glyma.20g160200* | *Glyma20g29960* | MP00449 | *Glyma.02g076000* | *Glyma02g08350* | 1831 | 1845 | + | 11.4394 | 6.69E-05 | 0.603 | TTGTGTCCTTTGATT |
| *Glyma.10g053500* | *Glyma10g06080* | MP00461 | *Glyma.05g056700* | *Glyma05g04330* | 854 | 874 | + | -3.92771 | 6.74E-05 | 0.169 | ATATTGTTTTTGACACAAATT |
| *Glyma.10g053500* | *Glyma10g06080* | MP00461 | *Glyma.05g056700* | *Glyma05g04330* | 862 | 882 | + | -3.92771 | 6.74E-05 | 0.169 | ATATTGTTTTTGACACAAATT |
| *Glyma.10g053500* | *Glyma10g06080* | MP00461 | *Glyma.05g056700* | *Glyma05g04330* | 862 | 882 | + | -3.92771 | 6.74E-05 | 0.169 | ATATTGTTTTTGACACAAATT |
| *Glyma.10g053500* | *Glyma10g06080* | MP00461 | *Glyma.05g056700* | *Glyma05g04330* | 887 | 907 | + | -3.92771 | 6.74E-05 | 0.169 | ATATTGTTTTTGACACAAATT |
| *Glyma.20g160200* | *Glyma20g29960* | MP00449 | *Glyma.06g152800* | *Glyma06g15820* | 497 | 511 | - | 11.4242 | 6.76E-05 | 0.603 | AATTGACCCTTTGCT |
| *Glyma.08g201900* | *Glyma08g21610* | MP00200 | *Glyma.07g080800* | *Glyma07g08810* | 227 | 241 | - | 10.403 | 6.83E-05 | 0.185 | TTTGATAATTATTAT |
| *Glyma.10g053500* | *Glyma10g06080* | MP00461 | *Glyma.16g018300* | *Glyma16g02090* | 941 | 961 | - | -4.01205 | 6.88E-05 | 0.169 | CTCTTCGTTTTTGCTTAAAAA |
| *Glyma.08g201900* | *Glyma08g21610* | MP00200 | *Glyma.20g176300* | *Glyma20g31630* | 1727 | 1741 | - | 10.3731 | 6.92E-05 | 0.185 | CAATCCAATGATGGC |
| *Glyma.08g201900* | *Glyma08g21610* | MP00200 | *Glyma.14g043500* | *Glyma14g04780* | 1205 | 1219 | + | 10.3731 | 6.92E-05 | 0.185 | ATTTCTAATAATTAT |
| *Glyma.08g201900* | *Glyma08g21610* | MP00200 | *Glyma.14g043500* | *Glyma14g04780* | 1487 | 1501 | + | 10.3731 | 6.92E-05 | 0.185 | ATTTCTAATAATTAT |
| *Glyma.08g201900* | *Glyma08g21610* | MP00200 | *Glyma.14g043500* | *Glyma14g04780* | 1855 | 1869 | + | 10.3731 | 6.92E-05 | 0.185 | ATTTCTAATAATTAT |
| *Glyma.10g053500* | *Glyma10g06080* | MP00461 | *Glyma.13g214600* | *Glyma13g28590* | 1726 | 1746 | - | -4.03614 | 6.93E-05 | 0.169 | TAAACCTGTTTTAAGGGAAAA |
| *Glyma.03g143100* | *Glyma03g29901* | MP00307 | *Glyma.16g146500* | *Glyma16g26210* | 1603 | 1613 | - | 11.2879 | 6.95E-05 | 1 | TTTGTACGTAA |
| *Glyma.02g261700* | *Glyma02g42960* | MP00302 | *Glyma.10g181900* | *Glyma10g32520* | 1226 | 1246 | - | 8.95714 | 6.95E-05 | 0.24 | GGAAGATGTGTGGGTGGATGG |
| *Glyma.08g201900* | *Glyma08g21610* | MP00200 | *Glyma.07g081700* | *Glyma07g08950* | 827 | 841 | - | 10.3582 | 6.97E-05 | 0.185 | ACACTTAATGATTAT |
| *Glyma.13g187500* | *Glyma13g25716* | MP00490 | *Glyma.06g068700* | *Glyma06g07230* | 1067 | 1077 | - | 11.5455 | 7.03E-05 | 0.92 | ATAACCAACTT |
| *Glyma.08g201900* | *Glyma08g21610* | MP00200 | *Glyma.13g162600* | *Glyma13g23150* | 684 | 698 | - | 10.3284 | 7.05E-05 | 0.185 | TATTACAATAATGAT |
| *Glyma.08g201900* | *Glyma08g21610* | MP00200 | *Glyma.20g013500* | *Glyma20g01680* | 436 | 450 | + | 10.3284 | 7.05E-05 | 0.185 | CGTAATGATGATGAA |
| *Glyma.02g013900* | *Glyma02g01740* | MP00627 | *Glyma.07g081700* | *Glyma07g08950* | 1388 | 1397 | + | 10.8673 | 7.10E-05 | 1 | ATACCTACTA |
| *Glyma.10g053500* | *Glyma10g06080* | MP00461 | *Glyma.18g017600* | *Glyma18g02110* | 1908 | 1928 | - | -4.15663 | 7.14E-05 | 0.169 | TATATGTTTTTTCTGGGGATT |
| *Glyma.10g053500* | *Glyma10g06080* | MP00461 | *Glyma.17g005600* | *Glyma17g00950* | 991 | 1011 | - | -4.18072 | 7.18E-05 | 0.169 | TTTTTTTTTTTTTTAAAAAAA |
| *Glyma.08g201900* | *Glyma08g21610* | MP00200 | *Glyma.07g080800* | *Glyma07g08810* | 415 | 429 | + | 10.2687 | 7.23E-05 | 0.185 | AATTTTAATAATTAT |
| *Glyma.03g143100* | *Glyma03g29901* | MP00307 | *Glyma.15g171900* | *Glyma15g18770* | 363 | 373 | + | 11.2576 | 7.24E-05 | 1 | GTTGTACTTTT |
| *Glyma.03g143100* | *Glyma03g29901* | MP00307 | *Glyma.15g171900* | *Glyma15g18770* | 379 | 389 | + | 11.2576 | 7.24E-05 | 1 | GTTGTACTTTT |
| *Glyma.03g143100* | *Glyma03g29901* | MP00307 | *Glyma.15g171900* | *Glyma15g18770* | 379 | 389 | + | 11.2576 | 7.24E-05 | 1 | GTTGTACTTTT |
| *Glyma.10g053500* | *Glyma10g06080* | MP00461 | *Glyma.06g152800* | *Glyma06g15820* | 1683 | 1703 | + | -4.24096 | 7.29E-05 | 0.169 | TTGCCATTTAAGGGGGAAAAT |
| *Glyma.08g201900* | *Glyma08g21610* | MP00200 | *Glyma.06g068700* | *Glyma06g07230* | 1551 | 1565 | + | 10.2239 | 7.36E-05 | 0.185 | TATTGTGATTATGAT |
| *Glyma.10g053500* | *Glyma10g06080* | MP00461 | *Glyma.14g043500* | *Glyma14g04780* | 1801 | 1821 | - | -4.28916 | 7.38E-05 | 0.169 | TACACTTTTTTAAAAGAAAAA |
| *Glyma.13g187500* | *Glyma13g25716* | MP00490 | *Glyma.05g246500* | *Glyma05g33790* | 660 | 670 | + | 11.5152 | 7.43E-05 | 0.92 | CTAACCGCAAT |
| *Glyma.13g187500* | *Glyma13g25716* | MP00490 | *Glyma.05g246500* | *Glyma05g33790* | 786 | 796 | + | 11.5152 | 7.43E-05 | 0.92 | CTAACCGCAAT |
| *Glyma.10g053500* | *Glyma10g06080* | MP00461 | *Glyma.08g156400* | *Glyma08g16620* | 2020 | 2040 | - | -4.3253 | 7.45E-05 | 0.169 | GTTACTTTTTCAGAGTAAAAA |
| *Glyma.08g201900* | *Glyma08g21610* | MP00200 | *Glyma.05g056700* | *Glyma05g04330* | 351 | 365 | - | 10.194 | 7.46E-05 | 0.185 | ACTTATAATAATGAT |
| *Glyma.08g201900* | *Glyma08g21610* | MP00200 | *Glyma.05g056700* | *Glyma05g04330* | 359 | 373 | - | 10.194 | 7.46E-05 | 0.185 | ACTTATAATAATGAT |
| *Glyma.08g201900* | *Glyma08g21610* | MP00200 | *Glyma.05g056700* | *Glyma05g04330* | 359 | 373 | - | 10.194 | 7.46E-05 | 0.185 | ACTTATAATAATGAT |
| *Glyma.08g201900* | *Glyma08g21610* | MP00200 | *Glyma.05g056700* | *Glyma05g04330* | 384 | 398 | - | 10.194 | 7.46E-05 | 0.185 | ACTTATAATAATGAT |
| *Glyma.08g201900* | *Glyma08g21610* | MP00200 | *Glyma.07g069700* | *Glyma07g07580* | 1026 | 1040 | - | 10.194 | 7.46E-05 | 0.185 | TACACCAATGATTAA |
| *Glyma.08g201900* | *Glyma08g21610* | MP00200 | *Glyma.07g069700* | *Glyma07g07580* | 1552 | 1566 | - | 10.194 | 7.46E-05 | 0.185 | TACACCAATGATTAA |
| *Glyma.03g143100* | *Glyma03g29901* | MP00307 | *Glyma.14g043500* | *Glyma14g04780* | 557 | 567 | - | 11.2424 | 7.47E-05 | 1 | GTTGTACTGTA |
| *Glyma.03g143100* | *Glyma03g29901* | MP00307 | *Glyma.14g043500* | *Glyma14g04780* | 839 | 849 | - | 11.2424 | 7.47E-05 | 1 | GTTGTACTGTA |
| *Glyma.03g143100* | *Glyma03g29901* | MP00307 | *Glyma.14g043500* | *Glyma14g04780* | 1207 | 1217 | - | 11.2424 | 7.47E-05 | 1 | GTTGTACTGTA |
| *Glyma.13g219900* | *Glyma13g29160* | MP00639 | *Glyma.03g242600* | *Glyma03g40250* | 1508 | 1515 | + | 11.6531 | 7.48E-05 | 1 | TGGACCAT |
| *Glyma.13g219900* | *Glyma13g29160* | MP00639 | *Glyma.17g005600* | *Glyma17g00950* | 856 | 863 | - | 11.6531 | 7.48E-05 | 1 | TGGACCAT |
| *Glyma.13g187500* | *Glyma13g25716* | MP00490 | *Glyma.06g152800* | *Glyma06g15820* | 1837 | 1847 | - | 11.5 | 7.56E-05 | 0.92 | TTAACCGATTC |
| *Glyma.13g187500* | *Glyma13g25716* | MP00490 | *Glyma.12g081900* | *Glyma12g08720* | 1939 | 1949 | + | 11.5 | 7.56E-05 | 0.92 | ATAACCAAAAT |
| *Glyma.13g187500* | *Glyma13g25716* | MP00490 | *Glyma.12g081900* | *Glyma12g08720* | 1939 | 1949 | + | 11.5 | 7.56E-05 | 0.92 | ATAACCAAAAT |
| *Glyma.10g053500* | *Glyma10g06080* | MP00461 | *Glyma.15g152100* | *Glyma15g16270* | 1677 | 1697 | - | -4.38554 | 7.56E-05 | 0.169 | CTTTTTTTTTTTGTTGAGAAT |
| *Glyma.02g261700* | *Glyma02g42960* | MP00302 | *Glyma.07g069700* | *Glyma07g07580* | 1465 | 1485 | - | 8.74286 | 7.65E-05 | 0.25 | TCCGACGCTGATGGTGGAGCC |
| *Glyma.02g261700* | *Glyma02g42960* | MP00302 | *Glyma.07g069700* | *Glyma07g07580* | 1991 | 2011 | - | 8.74286 | 7.65E-05 | 0.25 | TCCGACGCTGATGGTGGAGCC |
| *Glyma.10g053500* | *Glyma10g06080* | MP00461 | *Glyma.15g152100* | *Glyma15g16270* | 1676 | 1696 | - | -4.44578 | 7.68E-05 | 0.17 | TTTTTTTTTTTGTTGAGAATT |
| *Glyma.10g053500* | *Glyma10g06080* | MP00461 | *Glyma.14g043500* | *Glyma14g04780* | 1997 | 2017 | + | -4.44578 | 7.68E-05 | 0.17 | ATTTCGTTTTTGATATGAGTT |
| *Glyma.13g187500* | *Glyma13g25716* | MP00490 | *Glyma.05g056700* | *Glyma05g04330* | 1361 | 1371 | + | 11.4848 | 7.69E-05 | 0.92 | ATAACTGTAAA |
| *Glyma.13g187500* | *Glyma13g25716* | MP00490 | *Glyma.05g056700* | *Glyma05g04330* | 1369 | 1379 | + | 11.4848 | 7.69E-05 | 0.92 | ATAACTGTAAA |
| *Glyma.13g187500* | *Glyma13g25716* | MP00490 | *Glyma.05g056700* | *Glyma05g04330* | 1369 | 1379 | + | 11.4848 | 7.69E-05 | 0.92 | ATAACTGTAAA |
| *Glyma.13g187500* | *Glyma13g25716* | MP00490 | *Glyma.05g056700* | *Glyma05g04330* | 1394 | 1404 | + | 11.4848 | 7.69E-05 | 0.92 | ATAACTGTAAA |
| *Glyma.02g013900* | *Glyma02g01740* | MP00627 | *Glyma.05g056700* | *Glyma05g04330* | 1217 | 1226 | + | 10.6429 | 7.71E-05 | 1 | CAACCTACCT |
| *Glyma.02g013900* | *Glyma02g01740* | MP00627 | *Glyma.05g056700* | *Glyma05g04330* | 1225 | 1234 | + | 10.6429 | 7.71E-05 | 1 | CAACCTACCT |
| *Glyma.02g013900* | *Glyma02g01740* | MP00627 | *Glyma.05g056700* | *Glyma05g04330* | 1225 | 1234 | + | 10.6429 | 7.71E-05 | 1 | CAACCTACCT |
| *Glyma.02g013900* | *Glyma02g01740* | MP00627 | *Glyma.05g056700* | *Glyma05g04330* | 1250 | 1259 | + | 10.6429 | 7.71E-05 | 1 | CAACCTACCT |
| *Glyma.20g160200* | *Glyma20g29960* | MP00449 | *Glyma.12g081900* | *Glyma12g08720* | 102 | 116 | + | 11.2424 | 7.72E-05 | 0.603 | AGTTGTCCTTTAGAG |
| *Glyma.20g160200* | *Glyma20g29960* | MP00449 | *Glyma.12g081900* | *Glyma12g08720* | 102 | 116 | + | 11.2424 | 7.72E-05 | 0.603 | AGTTGTCCTTTAGAG |
| *Glyma.20g160200* | *Glyma20g29960* | MP00449 | *Glyma.12g081900* | *Glyma12g08720* | 409 | 423 | - | 11.2424 | 7.72E-05 | 0.603 | CAATGGCTTTTGCGT |
| *Glyma.20g160200* | *Glyma20g29960* | MP00449 | *Glyma.12g081900* | *Glyma12g08720* | 409 | 423 | - | 11.2424 | 7.72E-05 | 0.603 | CAATGGCTTTTGCGT |
| *Glyma.10g053500* | *Glyma10g06080* | MP00461 | *Glyma.06g068700* | *Glyma06g07230* | 562 | 582 | - | -4.49398 | 7.77E-05 | 0.171 | GTTATAGTATTTATGAAAATA |
| *Glyma.13g187500* | *Glyma13g25716* | MP00490 | *Glyma.12g047400* | *Glyma12g05140* | 700 | 710 | + | 11.4697 | 7.79E-05 | 0.92 | GTAACGGAAAC |
| *Glyma.13g187500* | *Glyma13g25716* | MP00490 | *Glyma.12g047400* | *Glyma12g05140* | 1402 | 1412 | + | 11.4697 | 7.79E-05 | 0.92 | GTAACGGAAAC |
| *Glyma.13g187500* | *Glyma13g25716* | MP00490 | *Glyma.12g047400* | *Glyma12g05140* | 1402 | 1412 | + | 11.4697 | 7.79E-05 | 0.92 | GTAACGGAAAC |
| *Glyma.08g201900* | *Glyma08g21610* | MP00200 | *Glyma.06g152800* | *Glyma06g15820* | 1014 | 1028 | - | 10.0896 | 7.79E-05 | 0.191 | AAATCTAATAATTGT |
| *Glyma.08g201900* | *Glyma08g21610* | MP00200 | *Glyma.06g152800* | *Glyma06g15820* | 1662 | 1676 | - | 10.0896 | 7.79E-05 | 0.191 | AAATCTAATAATTGT |
| *Glyma.20g160200* | *Glyma20g29960* | MP00449 | *Glyma.05g056700* | *Glyma05g04330* | 543 | 557 | - | 11.2121 | 7.89E-05 | 0.603 | TTGTGCCCCTTGGTC |
| *Glyma.20g160200* | *Glyma20g29960* | MP00449 | *Glyma.05g056700* | *Glyma05g04330* | 551 | 565 | - | 11.2121 | 7.89E-05 | 0.603 | TTGTGCCCCTTGGTC |
| *Glyma.20g160200* | *Glyma20g29960* | MP00449 | *Glyma.05g056700* | *Glyma05g04330* | 551 | 565 | - | 11.2121 | 7.89E-05 | 0.603 | TTGTGCCCCTTGGTC |
| *Glyma.20g160200* | *Glyma20g29960* | MP00449 | *Glyma.05g056700* | *Glyma05g04330* | 576 | 590 | - | 11.2121 | 7.89E-05 | 0.603 | TTGTGCCCCTTGGTC |
| *Glyma.10g053500* | *Glyma10g06080* | MP00461 | *Glyma.09g273900* | *Glyma09g41040* | 2 | 22 | + | -4.55422 | 7.89E-05 | 0.171 | ATTTCGATTATGATAAAAAGT |
| *Glyma.08g201900* | *Glyma08g21610* | MP00200 | *Glyma.13g162600* | *Glyma13g23150* | 677 | 691 | + | 10.0448 | 7.93E-05 | 0.193 | GGGAACAATCATTAT |
| *Glyma.10g053500* | *Glyma10g06080* | MP00461 | *Glyma.08g345900* | *Glyma08g45990* | 1222 | 1242 | - | -4.57831 | 7.94E-05 | 0.171 | GTAATTCTTTTAATGGAAAAT |
| *Glyma.10g053500* | *Glyma10g06080* | MP00461 | *Glyma.08g345900* | *Glyma08g45990* | 1432 | 1452 | - | -4.57831 | 7.94E-05 | 0.171 | GTAATTCTTTTAATGGAAAAT |
| *Glyma.10g053500* | *Glyma10g06080* | MP00461 | *Glyma.12g047400* | *Glyma12g05140* | 72 | 92 | - | -4.59036 | 7.96E-05 | 0.171 | ATTATAATTTTTTTAAAAAAA |
| *Glyma.10g053500* | *Glyma10g06080* | MP00461 | *Glyma.12g047400* | *Glyma12g05140* | 774 | 794 | - | -4.59036 | 7.96E-05 | 0.171 | ATTATAATTTTTTTAAAAAAA |
| *Glyma.10g053500* | *Glyma10g06080* | MP00461 | *Glyma.12g047400* | *Glyma12g05140* | 774 | 794 | - | -4.59036 | 7.96E-05 | 0.171 | ATTATAATTTTTTTAAAAAAA |
| *Glyma.10g053500* | *Glyma10g06080* | MP00461 | *Glyma.05g246500* | *Glyma05g33790* | 992 | 1012 | - | -4.59036 | 7.96E-05 | 0.171 | GTTACAAGATTAGCTAGAAAT |
| *Glyma.10g053500* | *Glyma10g06080* | MP00461 | *Glyma.05g246500* | *Glyma05g33790* | 1118 | 1138 | - | -4.59036 | 7.96E-05 | 0.171 | GTTACAAGATTAGCTAGAAAT |
| *Glyma.10g053500* | *Glyma10g06080* | MP00461 | *Glyma.16g018300* | *Glyma16g02090* | 940 | 960 | - | -4.62651 | 8.03E-05 | 0.172 | TCTTCGTTTTTGCTTAAAAAA |
| *Glyma.13g187500* | *Glyma13g25716* | MP00490 | *Glyma.05g056700* | *Glyma05g04330* | 2027 | 2037 | + | 11.4242 | 8.11E-05 | 0.92 | CTAACCGTTTT |
| *Glyma.13g187500* | *Glyma13g25716* | MP00490 | *Glyma.05g056700* | *Glyma05g04330* | 2035 | 2045 | + | 11.4242 | 8.11E-05 | 0.92 | CTAACCGTTTT |
| *Glyma.13g187500* | *Glyma13g25716* | MP00490 | *Glyma.05g056700* | *Glyma05g04330* | 2035 | 2045 | + | 11.4242 | 8.11E-05 | 0.92 | CTAACCGTTTT |
| *Glyma.13g187500* | *Glyma13g25716* | MP00490 | *Glyma.05g056700* | *Glyma05g04330* | 2060 | 2070 | + | 11.4242 | 8.11E-05 | 0.92 | CTAACCGTTTT |
| *Glyma.02g261700* | *Glyma02g42960* | MP00302 | *Glyma.05g062200* | *Glyma05g04940* | 2033 | 2053 | - | 8.58571 | 8.20E-05 | 0.262 | TGAGTTGGATGAGTCGGTTAC |
| *Glyma.08g097900* | *Glyma08g10350* | MP00006 | *Glyma.07g069700* | *Glyma07g07580* | 291 | 298 | - | 8.14634 | 8.20E-05 | 1 | GGACCCAC |
| *Glyma.08g097900* | *Glyma08g10350* | MP00006 | *Glyma.15g171900* | *Glyma15g18770* | 645 | 652 | - | 8.14634 | 8.20E-05 | 1 | GGTCCCAC |
| *Glyma.08g097900* | *Glyma08g10350* | MP00006 | *Glyma.15g171900* | *Glyma15g18770* | 661 | 668 | - | 8.14634 | 8.20E-05 | 1 | GGTCCCAC |
| *Glyma.08g097900* | *Glyma08g10350* | MP00006 | *Glyma.15g171900* | *Glyma15g18770* | 661 | 668 | - | 8.14634 | 8.20E-05 | 1 | GGTCCCAC |
| *Glyma.08g097900* | *Glyma08g10350* | MP00006 | *Glyma.16g018300* | *Glyma16g02090* | 844 | 851 | + | 8.14634 | 8.20E-05 | 1 | GGGTCCAC |
| *Glyma.08g201900* | *Glyma08g21610* | MP00200 | *Glyma.07g080800* | *Glyma07g08810* | 532 | 546 | + | 9.95522 | 8.23E-05 | 0.197 | TATAAAAATGATTGT |
| *Glyma.08g201900* | *Glyma08g21610* | MP00200 | *Glyma.07g080800* | *Glyma07g08810* | 607 | 621 | + | 9.95522 | 8.23E-05 | 0.197 | TATAAAAATGATTGT |
| *Glyma.08g201900* | *Glyma08g21610* | MP00200 | *Glyma.07g080800* | *Glyma07g08810* | 1506 | 1520 | + | 9.95522 | 8.23E-05 | 0.197 | TATAAAAATGATTGT |
| *Glyma.13g187500* | *Glyma13g25716* | MP00490 | *Glyma.07g081700* | *Glyma07g08950* | 1960 | 1970 | + | 11.4091 | 8.24E-05 | 0.92 | ATAACGGACAC |
| *Glyma.10g053500* | *Glyma10g06080* | MP00461 | *Glyma.14g043500* | *Glyma14g04780* | 1917 | 1937 | - | -4.73494 | 8.25E-05 | 0.172 | TTTTTCATTTTTAGGAGAAGT |
| *Glyma.08g201900* | *Glyma08g21610* | MP00200 | *Glyma.13g364900* | *Glyma13g44170* | 1219 | 1233 | - | 9.9403 | 8.28E-05 | 0.197 | ATGTTTAATGATGAA |
| *Glyma.08g201900* | *Glyma08g21610* | MP00200 | *Glyma.13g364900* | *Glyma13g44170* | 1258 | 1272 | - | 9.9403 | 8.28E-05 | 0.197 | ATGTTTAATGATGAA |
| *Glyma.08g201900* | *Glyma08g21610* | MP00200 | *Glyma.15g152100* | *Glyma15g16270* | 172 | 186 | - | 9.92537 | 8.33E-05 | 0.197 | TAAAAAAATCATTAT |
| *Glyma.13g187500* | *Glyma13g25716* | MP00490 | *Glyma.20g172000* | *Glyma20g31200* | 1034 | 1044 | + | 11.3939 | 8.37E-05 | 0.92 | TTAACTGAAAT |
| *Glyma.13g187500* | *Glyma13g25716* | MP00490 | *Glyma.20g172000* | *Glyma20g31200* | 1349 | 1359 | + | 11.3939 | 8.37E-05 | 0.92 | TTAACTGAAAT |
| *Glyma.03g143100* | *Glyma03g29901* | MP00307 | *Glyma.10g014400* | *Glyma10g01770* | 830 | 840 | - | 11.1364 | 8.46E-05 | 1 | CTTGTACTTAA |
| *Glyma.03g143100* | *Glyma03g29901* | MP00307 | *Glyma.10g014400* | *Glyma10g01770* | 838 | 848 | - | 11.1364 | 8.46E-05 | 1 | CTTGTACTTAA |
| *Glyma.10g053500* | *Glyma10g06080* | MP00461 | *Glyma.15g171900* | *Glyma15g18770* | 1665 | 1685 | + | -4.86747 | 8.53E-05 | 0.175 | TTAACATTTTTTGAAGAAGTA |
| *Glyma.10g053500* | *Glyma10g06080* | MP00461 | *Glyma.15g171900* | *Glyma15g18770* | 1681 | 1701 | + | -4.86747 | 8.53E-05 | 0.175 | TTAACATTTTTTGAAGAAGTA |
| *Glyma.10g053500* | *Glyma10g06080* | MP00461 | *Glyma.15g171900* | *Glyma15g18770* | 1681 | 1701 | + | -4.86747 | 8.53E-05 | 0.175 | TTAACATTTTTTGAAGAAGTA |
| *Glyma.02g261700* | *Glyma02g42960* | MP00302 | *Glyma.10g181900* | *Glyma10g32520* | 1110 | 1130 | - | 8.48571 | 8.56E-05 | 0.269 | GAAGTTGGATGCTTAGGTGGA |
| *Glyma.13g187500* | *Glyma13g25716* | MP00490 | *Glyma.05g012300* | *Glyma05g08060* | 1568 | 1578 | + | 11.3333 | 8.63E-05 | 0.92 | GTAACTGTAAC |
| *Glyma.13g187500* | *Glyma13g25716* | MP00490 | *Glyma.05g012300* | *Glyma05g08060* | 1569 | 1579 | + | 11.3333 | 8.63E-05 | 0.92 | GTAACTGTAAC |
| *Glyma.13g187500* | *Glyma13g25716* | MP00490 | *Glyma.05g012300* | *Glyma05g08060* | 1720 | 1730 | + | 11.3333 | 8.63E-05 | 0.92 | GTAACTGTAAC |
| *Glyma.10g053500* | *Glyma10g06080* | MP00461 | *Glyma.05g000200* | *Glyma05g09400* | 89 | 109 | + | -4.92771 | 8.66E-05 | 0.175 | TATATTTTTTAGATTAAAATA |
| *Glyma.10g053500* | *Glyma10g06080* | MP00461 | *Glyma.05g000200* | *Glyma05g09400* | 89 | 109 | + | -4.92771 | 8.66E-05 | 0.175 | TATATTTTTTAGATTAAAATA |
| *Glyma.10g053500* | *Glyma10g06080* | MP00461 | *Glyma.05g000200* | *Glyma05g09400* | 89 | 109 | + | -4.92771 | 8.66E-05 | 0.175 | TATATTTTTTAGATTAAAATA |
| *Glyma.10g053500* | *Glyma10g06080* | MP00461 | *Glyma.05g000200* | *Glyma05g09400* | 89 | 109 | + | -4.92771 | 8.66E-05 | 0.175 | TATATTTTTTAGATTAAAATA |
| *Glyma.10g053500* | *Glyma10g06080* | MP00461 | *Glyma.03g242600* | *Glyma03g40250* | 1614 | 1634 | - | -4.93976 | 8.68E-05 | 0.175 | TAAACTGTTTCTATTAAAAAA |
| *Glyma.08g201900* | *Glyma08g21610* | MP00200 | *Glyma.13g162600* | *Glyma13g23150* | 711 | 725 | - | 9.80597 | 8.73E-05 | 0.2 | CATTTTAATCATTAT |
| *Glyma.08g201900* | *Glyma08g21610* | MP00200 | *Glyma.13g364900* | *Glyma13g44170* | 969 | 983 | - | 9.80597 | 8.73E-05 | 0.2 | AAAATTAATAATTAA |
| *Glyma.08g201900* | *Glyma08g21610* | MP00200 | *Glyma.13g364900* | *Glyma13g44170* | 1008 | 1022 | - | 9.80597 | 8.73E-05 | 0.2 | AAAATTAATAATTAA |
| *Glyma.08g201900* | *Glyma08g21610* | MP00200 | *Glyma.05g000200* | *Glyma05g09400* | 142 | 156 | - | 9.79104 | 8.78E-05 | 0.2 | AACATTGATGATTAT |
| *Glyma.08g201900* | *Glyma08g21610* | MP00200 | *Glyma.05g000200* | *Glyma05g09400* | 142 | 156 | - | 9.79104 | 8.78E-05 | 0.2 | AACATTGATGATTAT |
| *Glyma.08g201900* | *Glyma08g21610* | MP00200 | *Glyma.05g000200* | *Glyma05g09400* | 142 | 156 | - | 9.79104 | 8.78E-05 | 0.2 | AACATTGATGATTAT |
| *Glyma.08g201900* | *Glyma08g21610* | MP00200 | *Glyma.05g000200* | *Glyma05g09400* | 142 | 156 | - | 9.79104 | 8.78E-05 | 0.2 | AACATTGATGATTAT |
| *Glyma.10g053500* | *Glyma10g06080* | MP00461 | *Glyma.10g014400* | *Glyma10g01770* | 417 | 437 | + | -5.0241 | 8.87E-05 | 0.176 | TATATGCATTTAGTAGAAAAT |
| *Glyma.10g053500* | *Glyma10g06080* | MP00461 | *Glyma.10g014400* | *Glyma10g01770* | 425 | 445 | + | -5.0241 | 8.87E-05 | 0.176 | TATATGCATTTAGTAGAAAAT |
| *Glyma.10g053500* | *Glyma10g06080* | MP00461 | *Glyma.08g349200* | *Glyma08g46360* | 1223 | 1243 | - | -5.03614 | 8.89E-05 | 0.177 | TTTATATTTATGATAAATAAT |
| *Glyma.13g187500* | *Glyma13g25716* | MP00490 | *Glyma.06g152800* | *Glyma06g15820* | 48 | 58 | - | 11.2879 | 8.92E-05 | 0.92 | CTAACCAAATA |
| *Glyma.13g187500* | *Glyma13g25716* | MP00490 | *Glyma.06g152800* | *Glyma06g15820* | 696 | 706 | - | 11.2879 | 8.92E-05 | 0.92 | CTAACCAAATA |
| *Glyma.10g053500* | *Glyma10g06080* | MP00461 | *Glyma.06g068700* | *Glyma06g07230* | 1312 | 1332 | + | -5.07229 | 8.97E-05 | 0.178 | GTTGCATTTTTTAGGGAGGGA |
| *Glyma.02g261700* | *Glyma02g42960* | MP00302 | *Glyma.20g176300* | *Glyma20g31630* | 1851 | 1871 | - | 8.37143 | 9.00E-05 | 0.279 | ATTGGGGGCTATGGTGGTTGG |
| *Glyma.03g143100* | *Glyma03g29901* | MP00307 | *Glyma.05g012300* | *Glyma05g08060* | 248 | 258 | - | 11.0758 | 9.10E-05 | 1 | TGTGTACTGAT |
| *Glyma.08g201900* | *Glyma08g21610* | MP00200 | *Glyma.05g246500* | *Glyma05g33790* | 718 | 732 | + | 9.70149 | 9.10E-05 | 0.2 | TAAAATGATAATTAA |
| *Glyma.08g201900* | *Glyma08g21610* | MP00200 | *Glyma.05g246500* | *Glyma05g33790* | 844 | 858 | + | 9.70149 | 9.10E-05 | 0.2 | TAAAATGATAATTAA |
| *Glyma.10g053500* | *Glyma10g06080* | MP00461 | *Glyma.20g013500* | *Glyma20g01680* | 1153 | 1173 | - | -5.13253 | 9.11E-05 | 0.179 | TTTTTTGATTTTGTGAAAGAA |
| *Glyma.08g201900* | *Glyma08g21610* | MP00200 | *Glyma.06g152800* | *Glyma06g15820* | 711 | 725 | - | 9.68657 | 9.16E-05 | 0.2 | TTTTATAATTATTAA |
| *Glyma.08g201900* | *Glyma08g21610* | MP00200 | *Glyma.06g152800* | *Glyma06g15820* | 1359 | 1373 | - | 9.68657 | 9.16E-05 | 0.2 | TTTTATAATTATTAA |
| *Glyma.10g053500* | *Glyma10g06080* | MP00461 | *Glyma.09g273900* | *Glyma09g41040* | 1695 | 1715 | + | -5.18072 | 9.22E-05 | 0.179 | GATATTTTTTTGACTTAAATA |
| *Glyma.08g201900* | *Glyma08g21610* | MP00200 | *Glyma.18g017600* | *Glyma18g02110* | 618 | 632 | + | 9.61194 | 9.42E-05 | 0.203 | AATCGTGATTATTAA |
| *Glyma.02g261700* | *Glyma02g42960* | MP00302 | *Glyma.05g012300* | *Glyma05g08060* | 739 | 759 | - | 8.2 | 9.70E-05 | 0.287 | GGCAGCAAACTTGGTGGTGGG |
| *Glyma.02g261700* | *Glyma02g42960* | MP00302 | *Glyma.05g012300* | *Glyma05g08060* | 740 | 760 | - | 8.2 | 9.70E-05 | 0.287 | GGCAGCAAACTTGGTGGTGGG |
| *Glyma.02g261700* | *Glyma02g42960* | MP00302 | *Glyma.05g012300* | *Glyma05g08060* | 891 | 911 | - | 8.2 | 9.70E-05 | 0.287 | GGCAGCAAACTTGGTGGTGGG |
| *Glyma.02g261700* | *Glyma02g42960* | MP00302 | *Glyma.05g012300* | *Glyma05g08060* | 1912 | 1932 | - | 8.2 | 9.70E-05 | 0.287 | GGCAGCAAACTTGGTGGTGGG |
| *Glyma.08g201900* | *Glyma08g21610* | MP00200 | *Glyma.03g242600* | *Glyma03g40250* | 1282 | 1296 | + | 9.52239 | 9.76E-05 | 0.204 | AGACCTAATAATGAC |
| *Glyma.10g053500* | *Glyma10g06080* | MP00461 | *Glyma.12g047400* | *Glyma12g05140* | 381 | 401 | + | -5.43373 | 9.81E-05 | 0.182 | TTTTTTTTTTTGAGACAAAGT |
| *Glyma.10g053500* | *Glyma10g06080* | MP00461 | *Glyma.12g047400* | *Glyma12g05140* | 381 | 401 | + | -5.43373 | 9.81E-05 | 0.182 | TTTTTTTTTTTGAGACAAAGT |
| *Glyma.08g201900* | *Glyma08g21610* | MP00200 | *Glyma.06g068700* | *Glyma06g07230* | 1387 | 1401 | - | 9.50746 | 9.82E-05 | 0.204 | TTAGCTAATCATGAG |
| *Glyma.10g053500* | *Glyma10g06080* | MP00461 | *Glyma.13g183800* | *Glyma13g25290* | 1681 | 1701 | + | -5.45783 | 9.87E-05 | 0.182 | TTATTCTTTTTTGGGATAAAA |
| *Glyma.02g261700* | *Glyma02g42960* | MP00302 | *Glyma.20g013500* | *Glyma20g01680* | 1396 | 1416 | + | 8.14286 | 9.94E-05 | 0.291 | ATCAAAGGAGGAGGAGGCGAC |
| *Glyma.10g053500* | *Glyma10g06080* | MP00461 | *Glyma.13g364900* | *Glyma13g44170* | 973 | 993 | + | -5.49398 | 9.96E-05 | 0.182 | TTATTAATTTTTATTAGGAAA |
| *Glyma.10g053500* | *Glyma10g06080* | MP00461 | *Glyma.13g364900* | *Glyma13g44170* | 1012 | 1032 | + | -5.49398 | 9.96E-05 | 0.182 | TTATTAATTTTTATTAGGAAA |
| *Glyma.02g261700* | *Glyma02g42960* | MP00302 | *Glyma.06g152800* | *Glyma06g15820* | 135 | 155 | + | 8.12857 | 0.0001 | 0.291 | GTCGTTGGAGGCAGTGGACGG |
| *Glyma.02g261700* | *Glyma02g42960* | MP00302 | *Glyma.06g152800* | *Glyma06g15820* | 783 | 803 | + | 8.12857 | 0.0001 | 0.291 | GTCGTTGGAGGCAGTGGACGG |

**Table S15.** 302 mQTL clusters for metabolites and lipids, their candidate genes, and miRNAs

| **Num** | **Class** | **Metabolites / lipids** | **Quantitative trait locus mapping** | | | | | |  | **Comparative genomics** | | | | | | **Candidate miRNAs** |
| --- | --- | --- | --- | --- | --- | --- | --- | --- | --- | --- | --- | --- | --- | --- | --- | --- |
|  |  |  | **Method^†^** | **Chr** | **Markers**  **associated** | **Effect** | **LOD score** | **r^2^（%）** |  | **Gene ID** | **Gene name** | **Arabidopsis homologs** | **Same pathway** | **KEGG pathway** | **Protein Family Name** |  |
| mQTL-C1 | **Carbohydrates** | D-Glucose, mannose | 1, 2 | 2 | Marker1287683, Marker1287683 | -0.82~0.80 | 2.53~2.87 | 5.61~7.71 |  | *Glyma02g39010* | *GmGH* | *AT3G47000* | Yes | gmx00500 | glycosyl hydrolase family protein | miR5669, miR390d, miR319h |
| mQTL-C2 |  | D-Glucose | 1, 8 | 4 | Marker2291230, Marker2351480 | 0.00~0.77 | 2.65~3.02 | 0.00~5.96 |  | *Glyma04g42740* | *GmALDH1* | *AT1G44170* | Yes | gmx00010 | aldehyde dehydrogenase |  |
| mQTL-C3 |  | fucose | 7, 1, 3 | 6 | Marker1981180, Marker2049801 | -0.67~-0.42 | 2.83~3.86 | 1.42~2.98 |  |  |  |  |  |  |  |  |
| mQTL-C4 |  | mannose | 1, 2 | 7 | Marker399016, Marker384918 | -0.72~0.69 | 2.60~2.82 | 7.33~7.40 |  | *Glyma07g17180* | *GmFPBP* | *AT3G54050* | Yes | gmx00051 | fructose-bisphosphatase | miR4359b, miR5770b |
| mQTL-C5 |  | D-Glucose | 1, 2 | 8 | Marker709955, Marker742943 | -0.56~0.83 | 3.52~4.09 | 4.41~8.36 |  |  |  |  |  |  |  |  |
| mQTL-C6 |  | D-Glucose | 1, 2, 7, 3 | 8 | Marker706158, Marker750703 | -0.97~0.52 | 3.14~3.63 | 2.48~8.16 |  | *Glyma08g45210* | *GmPHS* | *AT3G46970* | Yes | gmx00500 | α-glucan phosphorylase | miR5785 |
| mQTL-C7 |  | Inositol | 1, 4, 5, 7, 3 | 10 | Marker1085583, Marker1103627 | -1.27~-0.58 | 2.56~3.65 | 1.65~6.67 |  | *Glyma10g03770* | *GmGAUT* | *AT1G06780* | No | gmx00520 | galacturonosyl transferase | miR166g |
| mQTL-C8 |  | D-Fructose 2,6-bisphosphate | 1, 8, 7, 3 | 11 | Marker551663, Marker633142 | -1.58~-0.60 | 3.11~3.63 | 1.93~8.04 |  | *Glyma11g11160* | *GmDHLAT* | *AT4G21200* | No | gmx00010 | Dihydrolipoamide acetyltransferase | miR5380a, miR5380b |
| mQTL-C9 |  | D-Fructose 2,6-bisphosphate, D-Glucose | 1, 2, 8 | 11 | Marker584166, Marker624950 | -1.19~1.20 | 2.59~3.26 | 0.00~7.68 |  | *Glyma11g08020* | *GmGMD* | *AT5G66280* | No | gmx00051 | GDP-D-mannose 4,6-dehydratase | miR10191 |
| mQTL-C10 |  | D-Fructose 2,6-bisphosphate, mannose | 2 | 13 | Marker2855002, Marker2766645 | -0.62~0.45 | 2.56~2.73 | 3.06~5.56 |  | *Glyma13g20170* | *GmPOD* | *AT2G37130* | No | gmx00940 | Peroxidase superfamily protein | miR160d |
| mQTL-C11 |  | mannose, D-Fructose 2,6-bisphosphate | 2, 1 | 13 | Marker2849746, Marker2767659 | 0.45~0.76 | 2.56~2.74 | 3.06~3.14 |  | *Glyma13g21540* | *GmFBA* | *AT2G36460* | Yes | gmx00051 | fructose-bisphosphate aldolase |  |
| mQTL-P1 | **Peptides** | serine | 2, 1, 4, 8, 6, 3 | 1 | Marker1898999, Marker1898999 | -0.74~0.70 | 4.05~6.02 | 2.62~4.38 |  | *Glyma01g42900* | *GmGCL* | *AT4G23100* | Yes | gmx00270 | glutamate-cysteine ligase |  |
| mQTL-P2 |  | L-homoserine, beta-Alanine | 6, 2 | 2 | Marker1166836, Marker1282622 | -0.32~0.00 | 2.86~3.47 | 0.00~6.94 |  | *Glyma02g10060* | *GmPheRS* | *AT3G58140* | No | gmx00970 | phenylalanyl-tRNA synthetase class IIc family protein | miR9735, miR1512c, miR1512b |
| mQTL-P3 |  | threonine | 2 | 2 | Marker1188545, Marker1243816 | -1.15~-0.70 | 2.67~2.81 | 3.08~9.01 |  | *Glyma02g43650* | *GmSTYK* | *AT4G08850* | Yes | gmx00270 | serine/threonine protein kinase | miR9757, miR5380c |
|  |  |  |  |  |  |  |  |  |  | *Glyma02g42960* | *GmERF* | *AT2G40340* | No |  | AP2 domain |  |
| mQTL-P4 |  | oxoproline | 4, 8, 5, 3, 2 | 3 | Marker2476550, Marker2508846 | -0.86~0.62 | 2.74~3.36 | 1.52~3.35 |  | *Glyma03g39540* | *GmTAZ* | *AT3G05510* | No | gmx00564 | Cardiolipin Transacylase | miR164f |
| mQTL-P5 |  | leucine | 2, 1 | 4 | Marker2364583, Marker2364583 | -1.10~0.94 | 3.50~4.99 | 9.06~10.29 |  |  |  |  |  |  |  | miR10416 |
| mQTL-P6 |  | proline | 2, 1 | 4 | Marker2264224, Marker2264224 | -0.78~0.88 | 3.27~4.31 | 6.41~7.24 |  | *Glyma04g43360* | *GmSHMT* | *AT1G36370* | No | gmx00260 | serine hydroxymethyltransferase |  |
| mQTL-P7 |  | beta-Alanine, Isoleucine | 2, 4, 8, 5, 7, 3, 1, 6 | 6 | Marker2023741, Marker2016055 | -0.40~0.75 | 3.05~4.01 | 0.90~5.98 |  | *Glyma06g06475* | *GmPP* |  | No | gmx00564 | phospholipases | miR156k |
| mQTL-P8 |  | beta-Alanine | 1, 4, 8, 7, 3 | 6 | Marker1993733, Marker2000797 | -0.55~-0.32 | 2.87~4.51 | 3.06~5.77 |  | *Glyma06g12010* | *GmALDH2* | *AT1G44170* | Yes | gmx00410 | aldehyde dehydrogenase | miR4405 |
| mQTL-P9 |  | proline, oxoproline | 1, 3, 2, 4, 8, 5, 7 | 6 | Marker1997145, Marker1996387 | -1.04~0.65 | 2.56~4.55 | 1.20~6.74 |  | *Glyma06g13210* | *GmSMS* | *AT5G53120* | No | gmx00410 | spermine synthase |  |
| mQTL-P10 |  | leucine | 2, 3, 1 | 6 | Marker2005198, Marker2063896 | -0.88~0.92 | 3.56~3.81 | 2.01~7.18 |  | *Glyma06g22065* | *GmPSP1* | *AT1G18640* | Yes | gmx00260 | L-3-phosphoserine phosphatase | miR4382 |
| mQTL-P11 |  | putrescine | 4, 7 | 7 | Marker295268, Marker295268 | -0.79~-0.56 | 3.23~3.26 | 3.68~3.73 |  |  |  |  |  |  |  |  |
| mQTL-P12 |  | serine | 1, 4, 8, 5, 6, 7, 3, 2 | 9 | Marker464868, Marker468386 | -1.26~1.03 | 2.56~6.74 | 3.33~8.97 |  | *Glyma09g02160* | *GmGH* | *AT5G49720* | No | gmx00500 | glycosyl hydrolase family protein |  |
| mQTL-P13 |  | proline | 2, 8, 7, 3 | 9 | Marker435784, Marker465031 | -0.56~0.62 | 2.82~3.38 | 2.05~4.34 |  |  |  |  |  |  |  | miR168a |
| mQTL-P14 |  | Isoleucine, serine | 6, 2, 1 | 10 | Marker1096354, Marker1071152 | -0.83~0.63 | 2.68~4.41 | 1.85~8.28 |  | *Glyma10g00580* | *GmUDS* | *AT2G17570* | No | gmx00900 | Undecaprenyl pyrophosphate synthetase family protein |  |
| mQTL-P15 |  | putrescine | 2, 1, 7, 3 | 11 | Marker554961, Marker584166 | -0.99~0.97 | 2.74~3.86 | 2.76~6.01 |  | *Glyma11g07240* | *GmCYP* | *AT3G50660* | No | gmx00905 | Cytochrome P450 superfamily protein | miR1520j |
| mQTL-P16 |  | 4-Aminobutyric acid(GABA), Ethanolamine | 1, 3, 2 | 11 | Marker654290, Marker621663 | -0.47~0.78 | 2.57~3.22 | 0.99~6.09 |  | *Glyma11g35440* | *GmPAI* | *AT1G07780* | No | gmx00400 | phosphoribosylanthranilate isomerase | miR393a |
| mQTL-P17 |  | Isoleucine | 3, 2 | 12 | Marker2697651, Marker2635450 | -0.49~0.84 | 3.91~7.77 | 2.88~5.10 |  | *Glyma12g07570* | *GmIVD* | *AT3G45300* | Yes | gmx00280 | isovaleryl-CoA dehydrogenase | miR10424b |
| mQTL-P18 |  | serine | 1, 4, 8, 5, 6, 3 | 12 | Marker2729534, Marker2729534 | 0.68~1.00 | 3.25~6.21 | 1.84~6.17 |  | *Glyma12g08470* | *GmLGI* | *AT1G08110* | No | gmx01230 | lactoylglutathione lyase family protein | miR172a, miR530d |
| mQTL-P19 |  | serine | 1, 4, 8, 6, 3, 2 | 13 | Marker2851307, Marker2820990 | -0.82~0.84 | 4.22~5.71 | 2.79~5.64 |  | *Glyma13g22230* | *GmHACPS* | *AT3G11470* | No | gmx00770 | Holo-ACP Synthase | miR1507a |
| mQTL-P20 |  | beta-Alanine | 1, 2 | 14 | Marker1684827, Marker1675074 | -0.37~0.36 | 3.61~3.74 | 8.91~9.51 |  | *Glyma14g00760* | *GmACAT* | *AT5G47720* | Yes | gmx00640 | acetyl-CoA C-acetyltransferase |  |
| mQTL-P21 |  | Ethanolamine | 1, 7, 3, 2 | 15 | Marker12570, Marker12570 | -0.77~0.68 | 2.87~4.55 | 2.24~4.15 |  | *Glyma15g05630* | *GmSD* | *AT1G43710* | No | gmx00340 | Serine Decarboxylase |  |
| mQTL-P22 |  | Ethanolamine, Isoleucine | 3, 1 | 16 | Marker2528893, Marker2589580 | -0.77~-0.50 | 3.97~4.10 | 1.94~8.71 |  | *Glyma16g05480* | *GmFUS3* | *AT3G26790* | No |  | AP2/B3-like transcriptional factor family protein |  |
| mQTL-P23 |  | 4-Aminobutyric acid(GABA) | 8, 2, 4, 5, 7, 3 | 18 | Marker917786, Marker827261 | -0.76~0.89 | 3.26~4.36 | 0.00~10.19 |  |  |  |  |  |  |  |  |
| mQTL-P24 |  | oxoproline | 4, 8, 5, 7, 3 | 19 | Marker1495220, Marker1495220 | 0.43~0.84 | 3.02~3.68 | 1.40~3.34 |  | *Glyma19g01390* | *GmALDH3* | *AT1G23800* | No | gmx00410 | aldehyde dehydrogenase |  |
| mQTL-P25 |  | 4-Aminobutyric acid(GABA) | 2, 1 | 20 | Marker1448070, Marker1443183 | -1.00~0.99 | 3.64~3.98 | 8.54~9.89 |  | *Glyma20g00810* | *GmKMT* | *AT4G13460* | No | gmx00310 | histone-lysine N-methyltransferase |  |
| mQTL-P26 |  | leucine | 6, 3 | 20 | Marker1324457, Marker1324457 | -0.40~-0.39 | 3.14~3.14 | 1.21~1.29 |  | *Glyma20g02980* | *GmPK* | *AT5G56350* | Yes | gmx01230 | Pyruvate Kinase |  |
| mQTL-P27 |  | leucine | 6, 3 | 20 | Marker1372451, Marker1372451 | 0.49~0.50 | 3.70~3.70 | 1.95~2.03 |  | *Glyma20g24200* | *GmHADH* | *AT1G68010* | No | gmx00260 | D-isomer specific 2-hydroxyacid dehydrogenase |  |
| mQTL-O1 | **Organic acid** | 2-Oxoadipate | 5, 3, 2 | 2 | Marker1286890, Marker1286890 | -0.36~0.27 | 3.05~3.09 | 0.00~3.65 |  | *Glyma02g12660* | *GmCHIA* | *AT5G24090* | No | gmx00520 | chitinase A | miR4348c, miR398b |
| mQTL-O2 |  | Pyruvate | 6, 3 | 2 | Marker1193792, Marker1193792 | -0.91~-0.70 | 4.32~6.56 | 4.85~7.30 |  | *Glyma02g14211* | *GmAAPT* | *AT1G13560* | Yes | gmx00564 | Diacylglycerol Cholinephosphotransferase |  |
| mQTL-O3 |  | Pyruvate | 6, 3 | 2 | Marker1289976, Marker1284515 | 0.92~1.10 | 6.43~8.64 | 8.38~10.59 |  | *Glyma02g16010* | *GmGSR* | *AT3G54660* | No | gmx00480 | Pyridine nucleotide-disulphide oxidoreductase | miR166i, miR5677, miR167b, miR9751 |
| mQTL-O4 |  | Citraconic acid | 2, 1 | 4 | Marker2294377, Marker2292419 | -0.52~0.50 | 2.61~2.82 | 5.56~5.60 |  | *Glyma04g04060* | *GmPAH2* | *AT5G42870* | No | gmx00564 | Phosphatidate Phosphatase |  |
| mQTL-O5 |  | succinic acid | 2, 1, 4, 8, 7 | 5 | Marker2187077, Marker2178818 | -0.62~0.54 | 2.58~5.18 | 2.49~4.45 |  | *Glyma05g26660* | *GmGAD* | *AT2G02010* | Yes | gmx00250 | glutamate decarboxylase |  |
| mQTL-O6 |  | Pyruvate | 1, 4, 8, 5, 7, 3 | 5 | Marker2149219, Marker2119485 | 0.00~0.66 | 3.16~3.40 | 0.00~8.71 |  | *Glyma05g27960* | *GmCCOMT* | *AT1G67980* | No | gmx00941 | caffeoyl-CoA 3-O-methyltransferase |  |
| mQTL-O7 |  | citric acid | 2, 7 | 5 | Marker2122354, Marker2091293 | -1.08~0.39 | 3.70~3.97 | 2.85~6.65 |  | *Glyma05g34900* | *GmASS* | *AT4G24830* | No | gmx00250 | arginosuccinate synthase | miR156i |
| mQTL-O8 |  | succinic acid | 2, 1, 5 | 6 | Marker2053648, Marker2049860 | -0.74~0.74 | 3.17~3.84 | 1.56~6.74 |  |  |  |  |  |  |  |  |
| mQTL-O9 |  | citric acid, α-ketoisocaproic acid | 2, 5 | 7 | Marker397272, Marker386829 | -0.63~1.06 | 3.22~3.66 | 2.01~6.40 |  | *Glyma07g15170* | *GmPFK* | *AT4G26270* | No | gmx00010 | phosphofructokinase |  |
| mQTL-O10 |  | α-ketoisocaproic acid | 2, 1 | 7 | Marker352079, Marker299967 | -0.92~0.92 | 3.09~3.09 | 8.10~8.24 |  |  |  |  |  |  |  |  |
| mQTL-O11 |  | succinic acid | 1, 2 | 7 | Marker403343, Marker350745 | -0.75~0.75 | 2.92~3.41 | 3.60~6.80 |  | *Glyma07g33570* | *GmNR* | *AT2G15620* | No | gmx00910 | nitrite reductase |  |
| mQTL-O12 |  | 3-Hydroxypropionic acid | 2, 1 | 7 | Marker325634, Marker389074 | -0.38~0.38 | 2.63~2.94 | 3.37~5.54 |  | *Glyma07g38910* | *GmCLS* | *AT4G04870* | No | gmx00564 | Cardiolipin Synthase | miR171c |
| mQTL-O13 |  | 2-Oxobutyric acid | 2, 1, 8, 5, 6, 3 | 9 | Marker493960, Marker464961 | -0.69~0.53 | 2.65~4.14 | 1.51~3.51 |  | *Glyma09g04901* | *GmKCS* | *AT5G49070* | Yes | gmx00062 | Ketoacyl-CoA Synthase |  |
| mQTL-O14 |  | Pyruvate | 7, 3, 1 | 9 | Marker429142, Marker482975 | -0.54~-0.37 | 2.88~3.94 | 1.74~3.17 |  | *Glyma10g02040* | *GmFUM* | *AT2G47510* | Yes | gmx00020 | fumarase | miR159a, miR156m |
| mQTL-O15 |  | Pyruvate, D-Glyceric acid | 2, 1, 4, 8, 5, 6, 7, 3 | 10 | Marker1110830, Marker1118083 | -0.80~0.58 | 2.88~4.53 | 1.90~3.97 |  | *Glyma10g01840* | *GmCTPS* | *AT4G02120* | No | gmx00240 | CTP synthase family protein | miR4393a, miR10186a, miR164b |
| mQTL-O16 |  | Threonate | 1, 2 | 10 | Marker1116659, Marker1006888 | -0.63~0.66 | 2.96~3.46 | 5.72~6.14 |  | *Glyma10g32660* | *GmCDS* | *AT4G22340* | No | gmx00564 | CDP-DAG Synthase |  |
| mQTL-O17 |  | L-Malic acid | 1, 8, 7, 3 | 12 | Marker2669788, Marker2733475 | -1.19~-0.44 | 2.91~4.27 | 1.98~11.09 |  | *Glyma12g01380* | *GmGPDH* | *AT2G41540* | No | gmx00564 | NAD-dependent Glycerol-3-Phosphate Dehydrogenase | miR5762 |
| mQTL-O18 |  | isocitric acid | 1, 6 | 13 | Marker2824750, Marker2824750 | 0.00~0.84 | 3.81~4.05 | 0.00~5.64 |  | *Glyma13g01420* | *GmTPS* | *AT2G18700* | No | gmx00500 | trehalose phosphatase/synthase |  |
| mQTL-O19 |  | isocitric acid, oxalic acid | 1, 6, 2 | 13 | Marker2842700, Marker2759137 | -0.81~0.00 | 2.57~3.68 | 0.00~5.27 |  | *Glyma13g16440* | *GmMDH1* | *AT1G04410* | Yes | gmx00020 | lactate/malate dehydrogenase |  |
| mQTL-O20 |  | succinic acid, citric acid | 2, 1 | 13 | Marker2798086, Marker2850221 | -0.90~0.94 | 2.86~3.09 | 4.93~9.05 |  | *Glyma13g16440* | *GmMDH1* | *AT1G04410* | Yes | gmx00020 | lactate/malate dehydrogenase | miR156q |
| mQTL-O21 |  | 2-Oxobutyric acid | 5, 6, 3, 2 | 13 | Marker2803588, Marker2816233 | -0.47~0.66 | 3.04~3.34 | 1.61~2.72 |  | *Glyma13g26270* | *GmUR* | *AT3G12260* | No | gmx00190 | NADH: ubiquinone reductase |  |
| mQTL-O22 |  | Glucose-6-phosphate | 1 | 13 | Marker2832301, Marker2815445 | 1.21~1.21 | 2.88~2.88 | 7.66~7.66 |  | *Glyma13g30920* | *GmGP* | *AT3G46970* | No | gmx00500 | alpha-glucan phosphorylase |  |
| mQTL-O23 |  | citric acid | 2, 1 | 13 | Marker2866358, Marker2778095 | -1.22~1.29 | 3.87~5.45 | 9.48~9.88 |  | *Glyma13g41520* | *GmXTH* | *AT4G34890* | No | gmx04146 | xanthine dehydrogenase | miR172l |
| mQTL-O24 |  | D-Glyceric acid | 6 | 14 | Marker1651052, Marker1651052 | 0.24~0.24 | 3.70~3.70 | 0.95~0.95 |  | *Glyma14g08400* | *GmLPAAT4* | *AT1G75020* | No | gmx00561 | 1-Acylglycerol-3-Phosphate Acyltransferase | miR5678, miR5370 |
| mQTL-O25 |  | D-Glyceric acid | 1, 6, 3, 2 | 14 | Marker1738741, Marker1763128 | -0.43~0.45 | 2.82~4.05 | 2.70~3.80 |  | *Glyma14g36850* | *GmFBA* | *AT2G36460* | No | gmx01230 | fructose-bisphosphate aldolase |  |
| mQTL-O26 |  | L-Malic acid | 3 | 15 | Marker114116, Marker114116 | 0.36~0.36 | 3.17~3.17 | 1.35~1.35 |  | *Glyma15g01540* | *GmGLYK* | *AT1G80380* | No | gmx00260 | glycerate kinase |  |
| mQTL-O27 |  | D-Glyceric acid | 2, 1, 4, 8, 5, 6, 3 | 15 | Marker7992, Marker7992 | -0.55~0.80 | 3.33~4.34 | 2.15~4.06 |  | *Glyma15g05120* | *GmKCS7* | *AT1G71160* | No | gmx00062 | Ketoacyl-CoA Synthase | miR166j |
| mQTL-O28 |  | oxalic acid | 5, 7, 3, 1, 2 | 15 | Marker128559, Marker128559 | -0.76~1.06 | 3.28~4.40 | 0.00~6.50 |  |  |  |  |  |  |  |  |
| mQTL-O29 |  | α-ketoisocaproic acid | 4, 8, 5, 7, 3 | 16 | Marker2545413, Marker2545413 | -0.91~-0.46 | 3.12~3.55 | 1.83~3.65 |  |  |  |  |  |  |  |  |
| mQTL-O30 |  | oxalacetic acid | 1 | 17 | Marker169306, Marker169306 | 1.34~1.34 | 2.58~2.58 | 6.82~6.82 |  | *Glyma17g37720* | *GmHXK* | *AT1G47840* | No | gmx00500 | Hexokinase |  |
| mQTL-O31 |  | L-Malic acid | 2, 3, 1, 4, 8, 5, 7 | 20 | Marker1425110, Marker1398247 | -0.97~1.16 | 3.09~4.56 | 2.16~11.51 |  | *Glyma20g36740* | *GmGME* | *AT5G28840* | No | gmx00520 | GDP-D-mannose 3',5'-epimeras | miR167j |
| mQTL-F1 | **Fatty acids** | linoleic acid | 7, 3 | 1 | Marker1893758, Marker1893758 | -0.22~-0.22 | 4.61~5.08 | 3.01~5.08 |  |  |  |  |  |  |  |  |
| mQTL-F2 |  | linoleic acid, stearic acid | 8, 7 | 1 | Marker1866687, Marker1806679 | 0.00~0.00 | 3.58~4.71 | 0.00~2.23 |  | *Glyma01g22850* | *GmWBC14* | *AT3G25620* | No | gmx00073 | ABC Transporter | miR10407a |
| mQTL-F3 |  | FA(18:3), FA(16:0), FA(18:0), FA(18:1), FA(20:0), FA(20:1), FA(22:1) | 2, 4, 8, 5, 1, 7 | 1 | Marker1907045, Marker1804614 | -0.42~0.30 | 2.57~3.22 | 1.92~3.64 |  | *Glyma01g44600* | *GmOPR* | *AT1G76690* | No | gmx00592 | Oxo-Phytodienoic Acid Reductase |  |
| mQTL-F4 |  | prostaglandin A | 1, 2 | 2 | Marker1229647, Marker1172550 | -0.86~0.86 | 3.62~3.89 | 6.88~7.32 |  |  |  |  |  |  |  |  |
| mQTL-F5 |  | OAHFA(18:1/18:0), FA(18:2), FA(18:3), FA(20:1), linoleic acid, Octanoic acid | 2, 7, 6, 3, 5, 4, 8 | 2 | Marker1210105, Marker1280678 | -0.46~0.68 | 3.01~3.75 | 1.10~9.85 |  | *Glyma02g11570* | *GmCer9* | *AT4G34100* | Yes | gmx04141 | E3 ubiquitin Ligase |  |
| mQTL-F6 |  | Arachidic acid, FA(18:2) | 2 | 2 | Marker1281312, Marker1281312 | -0.15~0.46 | 2.73~3.35 | 7.24~8.83 |  |  |  |  |  |  |  | miR169b, miR5670b, miR4348c |
| mQTL-F7 |  | OAHFA(18:1/18:0), Arachidic acid | 1, 2 | 2 | Marker1174825, Marker1206053 | -0.61~0.23 | 2.81~3.18 | 7.47~8.30 |  | *Glyma02g15600* | *GmFAB2* | *AT2G43710* | Yes | gmx01040 | Stearoyl-ACP Desaturase | miR166i |
| mQTL-F8 |  | FA(18:2) | 6, 3 | 2 | Marker1255692, Marker1255692 | 0.17~0.17 | 3.03~3.40 | 1.04~1.04 |  | *Glyma02g41660* | *GmGPAT5* | *AT3G11430* | Yes | gmx00561 | Glycerol-3-Phosphate Acyltransferase |  |
| mQTL-F9 |  | palmitic acid | 5, 2 | 3 | Marker2454509, Marker2498774 | -0.28~0.23 | 2.54~3.20 | 1.91~6.89 |  | *Glyma03g01210* | *GmACT2* | *AT1G01710* | No | gmx01040 | Acyl-CoA Thioesterase |  |
| mQTL-F10 |  | 1-Hexadecanol | 1, 2 | 3 | Marker2487419, Marker2407895 | -0.48~0.51 | 2.51~2.78 | 2.48~4.44 |  | *Glyma03g02171* | *GmLPCAT* | *AT2G45670* | Yes | gmx00565 | 1-Acylglycerol-3-Phosphocholine Acyltransferase,1-Acylglycerol-3-Phosphoethanolamine Acyltransferase | |
| mQTL-F11 |  | oleic acid | 5, 7, 3 | 4 | Marker2351480, Marker2351480 | 0.00~0.42 | 3.52~3.71 | 0.00~4.18 |  | *Glyma04g43070* | *GmPIPK-IB* | *AT1G21920* | No | gmx00564 | Phosphatidylinositol-Phosphate Kinase type IB |  |
| mQTL-F12 |  | Octanoic acid | 3, 2 | 5 | Marker2137873, Marker2163727 | -0.71~0.14 | 3.50~33.61 | 1.58~5.59 |  | *Glyma05g31270* | *GmCTS* | *AT4G39850* | Yes | gmx00073 | ABC Acyl Transporter |  |
| mQTL-F13 |  | FA(18:2), FA(18:3) | 1 | 6 | Marker1997145, Marker1997145 | -0.33~-0.32 | 2.92~3.30 | 3.29~3.70 |  |  |  |  |  |  |  |  |
| mQTL-F14 |  | linoleic acid, linolenic acid | 7, 2, 1, 3 | 6 | Marker1998840, Marker1981720 | -0.57~0.58 | 2.77~4.03 | 2.33~7.72 |  | *Glyma06g20800* | *GmMYB41* | *AT3G47600* | Yes |  | MYB Transcription Factors |  |
| mQTL-F15 |  | lauric acid, Octanoic acid | 7, 3, 2 | 6 | Marker1992482, Marker2060238 | -0.45~0.88 | 2.83~3.54 | 1.77~6.11 |  |  |  |  |  |  |  |  |
| mQTL-F16 |  | lauric acid, Octanoic acid | 1 | 6 | Marker2063896, Marker2048062 | -0.82~-0.24 | 2.56~2.88 | 5.04~5.37 |  |  |  |  |  |  |  |  |
| mQTL-F17 |  | FA(18:0), FA(20:0), FA(22:1) | 2 | 6 | Marker1996457, Marker2044143 | 0.27~0.35 | 2.59~3.25 | 3.47~4.34 |  | *Glyma06g44440* | *GmZF351* | *AT1G03790* | No |  | Zinc finger C-x8-C-x5-C-x3-H type family protein | miR10200 |
| mQTL-F18 |  | Capric Acid | 2 | 7 | Marker318453, Marker311437 | -1.06~-0.60 | 3.01~3.26 | 3.92~9.68 |  | *Glyma07g04080* | *GmPDAT1* | *AT5G13640* | Yes | gmx00561 | Phospholipid : Diacylglycerol Acyltransferase |  |
| mQTL-F19 |  | Capric Acid | 4, 8, 6, 1 | 7 | Marker365141, Marker371896 | 0.44~1.07 | 2.81~3.35 | 1.84~8.05 |  | *Glyma07g05390* | *GmPPase* | *AT1G01050* | No | gmx00190 | Pyrophosphatase |  |
| mQTL-F20 |  | linolenic acid | 7, 3 | 7 | Marker370376, Marker370376 | -0.39~-0.39 | 3.50~3.61 | 1.78~2.74 |  | *Glyma07g13780* | *GmPAH* | *AT4G37070* | No |  | Acyl-Hydrolase (Patatin-like) |  |
| mQTL-F21 |  | FA(16:0), FA(18:0), FA(18:1), FA(20:0), FA(20:1) | 2, 1 | 7 | Marker365984, Marker395114 | -0.46~0.44 | 2.67~3.19 | 6.94~8.30 |  | *Glyma07g17110* | *GmIPCS* | *AT3G54020* | No |  | Inositolphosphorylceramide Synthase |  |
| mQTL-F22 |  | linolenic acid, prostaglandin A | 2, 1 | 8 | Marker744781, Marker667322 | -0.81~0.72 | 2.81~13.58 | 4.11~5.09 |  |  |  |  |  |  |  | miR319b, miR5036 |
| mQTL-F23 |  | FA(18:2), FA(16:0) | 7, 2 | 9 | Marker522118, Marker464246 | -0.21~0.25 | 2.54~3.13 | 3.11~3.20 |  | *Glyma09g00960* | *GmMCD* | *AT4G04320* | No | gmx00410 | Malonyl-CoA Decarboxylase |  |
| mQTL-F24 |  | palmitic acid | 1, 6 | 9 | Marker499635, Marker499635 | -0.62~-0.41 | 4.61~5.18 | 5.78~11.71 |  |  |  |  |  |  |  | miR1516b |
| mQTL-F25 |  | palmitic acid | 1, 6 | 9 | Marker526905, Marker526905 | 0.44~0.65 | 5.60~5.69 | 6.59~12.69 |  | *Glyma09g04901* | *GmKCS* | *AT5G49070* | Yes | gmx00062 | Ketoacyl-CoA Synthase |  |
| mQTL-F26 |  | FA(16:0), FA(18:0), FA(18:2) | 2, 1 | 9 | Marker418134, Marker434940 | -0.36~0.40 | 2.51~2.83 | 5.39~5.93 |  |  |  |  |  |  |  |  |
| mQTL-F27 |  | FA(22:1) | 1, 2 | 10 | Marker1056155, Marker1056155 | -0.60~0.57 | 2.61~3.36 | 0.19~5.37 |  | *Glyma10g38660* | *GmKCS6* | *AT1G68530* | No | gmx00062 | Ketoacyl-CoA Synthase | miR167f, miR167h |
| mQTL-F28 |  | oleic acid | 2, 1 | 11 | Marker664607, Marker584166 | -0.79~0.80 | 2.57~2.68 | 3.09~5.42 |  | *Glyma11g07590* | *GmLPP-ε2* | *AT5G66450* | No |  | Phosphatidate Phosphatase | miR1520j |
| mQTL-F29 |  | FA(20:1), FA(20:2), FA(22:1) | 2 | 12 | Marker2710775, Marker2718329 | 0.32~0.53 | 2.87~2.98 | 0.16~4.36 |  |  |  |  |  |  |  | miR10424b |
| mQTL-F30 |  | Capric Acid | 2, 6, 7, 3 | 14 | Marker1640915, Marker1640915 | -0.56~0.48 | 2.67~3.31 | 0.00~4.17 |  | *Glyma14g05510* | *GmTAGL* | *AT5G14180* | No | gmx00561 | Triacylglycerol Lipase (TAGL) |  |
| mQTL-F31 |  | FA(18:0), FA(20:0) | 2 | 14 | Marker1627639, Marker1623656 | 0.24~0.26 | 2.65~2.84 | 3.95~4.40 |  | *Glyma14g06290* | *GmABI* | *AT2G40220* | Yes |  | Abscisic Acid Insensitive (ABI) transcription factors | miR393e |
| mQTL-F32 |  | FA(16:0), FA(18:0), FA(20:0) | 4, 1, 5, 3, 7 | 14 | Marker1647615, Marker1647615 | -0.43~-0.20 | 3.03~3.84 | 1.97~4.00 |  | *Glyma14g08010* | *GmEK* | *AT2G26830* | No | gmx00564 | Ethanolamine Kinase | miR172d |
| mQTL-F33 |  | FA(22:1), FA(18:0), palmitic acid | 2, 7 | 15 | Marker121025, Marker61697 | 0.00~0.52 | 2.60~8.62 | 3.58~26.47 |  | *Glyma15g05470* | *GmSWEET10a* | *AT5G13170* | No | gmx00500 | Sugar transporter | miR166j, miR394d, miR3522 |
| mQTL-F34 |  | FA(18:1), FA(20:0), FA(20:1), OAHFA(18:1/18:0), FA(16:0) | 5, 4, 8, 7, 3, 1 | 15 | Marker96492, Marker26894 | -0.58~-0.20 | 2.73~4.69 | 2.34~7.10 |  |  |  |  |  |  |  | miR169n, miR169d, miR169p, miR169e |
| mQTL-F35 |  | lauric acid | 2, 4, 8, 5 | 16 | Marker2543407, Marker2543407 | -1.10~0.64 | 2.70~3.40 | 2.17~3.92 |  | *Glyma16g00790* | *GmPDAT1* | *AT5G13640* | Yes | gmx00561 | Phospholipid : Diacylglycerol Acyltransferase |  |
| mQTL-F36 |  | stearic acid | 2, 1, 6, 7 | 16 | Marker2545413, Marker2545413 | -0.87~0.81 | 2.78~3.93 | 2.16~3.55 |  |  |  |  |  |  |  | miR166a, miR5766 |
| mQTL-F37 |  | prostaglandin A | 2, 1 | 16 | Marker2522737, Marker2524671 | -0.70~0.69 | 2.60~2.61 | 4.49~4.80 |  | *Glyma16g06230* | *GmTAGL* | *AT5G14180* | Yes |  | Triacylglycerol Lipase (TAGL) | miR1526 |
| mQTL-F38 |  | palmitoleic acid, oleic acid | 1, 2, 7, 3 | 16 | Marker2614429, Marker2592949 | -0.75~0.87 | 2.62~3.68 | 1.58~6.69 |  |  |  |  |  |  |  | miR9729, miR5041, miR5767 |
| mQTL-F39 |  | oleic acid | 5, 7 | 16 | Marker2542918, Marker2542918 | 0.00~0.00 | 3.02~3.06 | 0.00~3.38 |  | *Glyma16g27430* | *GmHAD* | *AT5G60340* | No | gmx00061 | Hydroxyacyl-ACP Dehydrase | miR1510a |
| mQTL-F40 |  | Octanoic acid | 2, 1 | 17 | Marker206433, Marker200629 | -0.24~0.24 | 2.56~2.76 | 5.26~5.41 |  | *Glyma17g12940* | *GmFatB1* | *AT1G08510* | Yes | gmx00061 | Acyl-ACP Thioesterase B | miR171l, miR319l |
| mQTL-F41 |  | FA(18:3) | 4, 5, 1 | 18 | Marker884878, Marker884878 | -0.48~-0.31 | 3.01~3.27 | 1.98~3.62 |  | *Glyma18g53100* | *GmPP* | *AT3G02600* | No | gmx00561 | Phosphatidate Phosphatase | miR156e, miR482c, miR862a, miR5037b, miR5044 |
| mQTL-F42 |  | FA(22:1) | 1, 2 | 20 | Marker1335369, Marker1322197 | -0.63~5.01 | 3.15~91.87 | 5.88~14.59 |  | *Glyma20g02570* | *GmLCAT* | *AT4G19860* | Yes | gmx00592 | Lecithin:cholesterol acyltransferase |  |
| mQTL-F43 |  | FA(16:0), FA(18:1), FA(20:1), FA(20:2), FA(18:0), FA(20:0) | 1 | 20 | Marker1327768, Marker1414253 | -0.40~-0.31 | 2.66~3.60 | 5.26~7.06 |  |  |  |  |  |  |  | miR10446 |
| mQTL-S1 | **Sphingolipids** | Cer(d18:2/16:0), CerG1(d18:2/16:0) | 2 | 15 | Marker5035, Marker29446 | 0.22~0.38 | 2.57~2.91 | 5.22~5.86 |  | *Glyma15g02710* | *GmPLDε1* | *AT1G55180* | No | gmx00564 | Phospholipase D ε |  |
| mQTL-S2 |  | CerG1(d18:2/22:1) | 1, 2 | 15 | Marker107799, Marker107799 | -0.25~0.25 | 3.74~3.77 | 7.30~7.46 |  | *Glyma15g05150* | *GmPIPK-IB* | *AT1G10900* | Yes | gmx00564 | Phosphatidylinositol-Phosphate Kinase type IB | miR166j, miR394d |
| mQTL-S3 |  | 2-Aminooctadecane-1,3-diol | 4, 3, 2 | 15 | Marker15320, Marker6642 | -0.49~0.75 | 2.89~3.48 | 2.17~5.30 |  | *Glyma15g21444* | *GmnsPLC* | *AT1G07230* | No | gmx00565 | Phospholipase C (Non specific) |  |
| mQTL-G1 | **Glycerolipids** | TG(16:0/16:0/18:1), TG(18:0/18:0/18:1), TG(18:0/18:1/18:1) | 3, 5, 1, 7, 2 | 17 | Marker201006, Marker243952 | -0.24~0.21 | 2.93~5.55 | 1.35~5.63 |  | *Glyma17g08220* | *GmFAD6* | *AT4G30950* | No |  | Oleate Desaturase |  |
| mQTL-G2 |  | TG(18:0/16:0/18:1), TG(18:2/18:2/18:2), TG(20:0/18:1/18:2) | 6, 8, 4, 5, 1, 3, 7 | 17 | Marker231031, Marker231031 | -0.19~-0.05 | 2.87~4.88 | 1.23~3.97 |  | *Glyma17g08510* | *GmDAGK* | *AT5G07920* | No | gmx00561 | Diacylglycerol Kinase | miR4373 |
| mQTL-G3 |  | TG(16:0/16:0/18:1), TG(18:3/18:2/18:2), TG(18:0/18:0/18:1), TG(18:0/18:1/18:1) | 2 | 17 | Marker206433, Marker200629 | 0.19~0.27 | 2.65~15.20 | 3.37~8.31 |  | *Glyma17g12940* | *GmFatB1* | *AT1G08510* | Yes | gmx00061 | Acyl-ACP Thioesterase B | miR319l |
| mQTL-G4 |  | TG(18:0/16:0/18:1), TG(16:0/16:0/18:1), TG(18:3/18:2/18:2) | 2, 1, 5, 3, 8, 6 | 17 | Marker223571, Marker223406 | -0.37~0.28 | 2.58~23.35 | 0.00~8.49 |  | *Glyma17g13120* | *GmOBO* | *AT3G18570* | Yes |  | Oil-Body Oleosin | miR162c |
| mQTL-G5 |  | TG(16:0/16:0/18:2), TG(16:0/16:0/18:1) | 7, 2 | 17 | Marker229064, Marker177973 | -0.09~0.09 | 2.50~4.96 | 2.88~3.41 |  | *Glyma17g15320* | *GmACX1* | *AT4G16760* | Yes | gmx00592 | Acyl-CoA Oxidase |  |
| mQTL-G6 |  | TG(16:0/18:1/18:1) | 2, 7 | 17 | Marker203384, Marker236854 | -0.16~0.11 | 2.62~4.25 | 3.37~3.64 |  |  |  |  |  |  |  |  |
| mQTL-G7 |  | DG(20:0/18:2), TG(18:3/18:2/18:3) | 6, 2, 1 | 17 | Marker254299, Marker156755 | -0.22~0.22 | 2.68~5.01 | 0.01~6.79 |  |  |  |  |  |  |  | miR156y |
| mQTL-G8 |  | TG(16:0/16:0/18:1), TG(16:0/18:1/18:1), TG(16:0/18:2/18:3) | 1, 2, 7, 8 | 17 | Marker249266, Marker211267 | -0.22~0.22 | 2.63~3.69 | 0.00~7.89 |  | *Glyma17g36130* | *GmPAH2* | *AT5G42870* | Yes |  | Phosphatidate Phosphatase |  |
| mQTL-G9 |  | TG(16:0/16:0/18:2), DG(18:2/18:2), TG(16:0/18:2/18:3), TG(16:0/16:0/18:1), TG(16:0/18:1/18:1), TG(18:3/18:2/18:2), TG(18:1/18:2/18:2), TG(18:0/16:0/18:1), TG(20:0/18:1/18:2), TG(16:0/16:0/18:3) | 2, 4, 5, 1, 7, 6 | 17 | Marker182106, Marker181452 | -0.23~0.22 | 2.51~5.41 | 0.10~11.01 |  | *Glyma17g36670* | *GmLPAAT4* | *AT1G75020* | Yes | gmx00561 | 1-Acylglycerol-3-Phosphate Acyltransferase |  |
| mQTL-G10 |  | TG(16:0/16:0/18:3), TG(16:0/16:0/18:1), TG(18:0/16:0/18:1), TG(16:0/18:1/18:2), TG(20:0/18:1/18:2) | 2, 1, 4, 5, 3, 8 | 17 | Marker201004, Marker224450 | -0.20~0.27 | 2.99~5.29 | 0.50~9.59 |  |  |  |  |  |  |  |  |
| mQTL-G11 |  | SQDG(16:0/18:1), DGDG(16:0/18:3), DGDG(18:3/18:3), TG(18:1/18:2/18:3) | 1, 7, 2 | 18 | Marker861014, Marker864664 | -0.66~0.81 | 2.89~3.87 | 2.93~9.61 |  | *Glyma18g00381* | *GmLTP* | *AT1G55260* | No | gmx00073 | Lipid Transfer Protein |  |
| mQTL-G12 |  | SQDG(16:0/18:2), TG(18:0/18:0/18:1), TG(20:0/18:1/18:1), TG(18:0/18:1/18:1) | 2, 6, 1 | 18 | Marker864664, Marker955424 | -0.28~0.44 | 2.52~4.73 | 0.91~8.92 |  | *Glyma18g02900* | *GmUGP3* | *AT3G56040* | No | gmx00561 | UDP-Glucose Pyrophosphorylase | miR4355 |
| mQTL-G13 |  | TG(18:0/18:1/18:1), SQDG(16:0/18:2) | 2, 1 | 18 | Marker955424, Marker958881 | -0.19~0.39 | 3.44~4.07 | 3.41~8.41 |  | *Glyma18g03090* | *GmPIPLC* | *AT3G08510* | Yes | gmx04070 | Phosphoinositide-specific Phospholipase C | miR4355 |
| mQTL-G14 |  | TG(18:0/18:1/18:1) | 2 | 18 | Marker902147, Marker919180 | -0.32~0.45 | 10.13~18.90 | 3.18~6.34 |  | *Glyma18g04540* | *GmLAH* | *AT5G37710* | No |  | Lipid Acylhydrolase-like | miR172c, miR10441, miR4396 |
| mQTL-G15 |  | TG(18:0/18:0/18:1) | 7, 6, 4, 3 | 18 | Marker893979, Marker935328 | -0.15~0.00 | 3.11~4.41 | 1.57~3.79 |  | *Glyma18g11620* | *GmFAD4* | *AT4G27030* | No |  | FAD4-like Desaturase |  |
| mQTL-G16 |  | TG(18:0/18:0/18:1), TG(20:0/18:1/18:1) | 2, 1, 8 | 18 | Marker965265, Marker826585 | -0.14~0.36 | 2.70~27.68 | 0.00~5.12 |  | *Glyma18g11683* | *GmLT* | *AT4G31050* | No | gmx00785 | Lipoyltransferase | miR5042 |
| mQTL-G17 |  | DG(16:0/16:0), DG(18:0/16:0), DG(18:0/18:0), DG(18:0/18:1) | 7, 4, 3, 8, 5, 6 | 18 | Marker989483, Marker846243 | 0.00~0.34 | 3.23~4.17 | 0.00~4.07 |  |  |  |  |  |  |  |  |
| mQTL-G18 |  | DG(16:0/16:0), DG(18:0/16:0), DG(18:0/18:0) | 2 | 18 | Marker875290, Marker955973 | -0.24~-0.21 | 3.16~4.00 | 3.96~5.02 |  |  |  |  |  |  |  |  |
| mQTL-G19 |  | TG(16:0/18:1/18:3) | 2, 3, 8, 4, 1, 7 | 18 | Marker841529, Marker835065 | -0.21~0.18 | 2.83~3.97 | 2.21~5.19 |  |  |  |  |  |  |  | miR4364b |
| mQTL-G20 |  | DG(16:0/16:0), DG(18:0/16:0), DG(18:0/18:0), DG(16:0/18:1) | 2, 4, 8 | 18 | Marker935165, Marker958051 | -0.42~0.19 | 3.15~5.34 | 1.96~11.52 |  | *Glyma18g43240* | *GmACX4* | *AT3G51840* | Yes | gmx00592 | Acyl-CoA Oxidase | miR10440 |
| mQTL-G21 |  | DG(18:0/18:1), DG(16:0/18:1), DG(18:1/18:1), DG(18:0/18:0), DG(16:0/16:0) | 2, 1 | 18 | Marker920733, Marker926627 | -0.45~0.41 | 2.72~4.45 | 5.97~11.39 |  | *Glyma18g45210* | *GmPECT1* | *AT2G38670* | No | gmx00564 | CTP:phosphorylethanolamine cytidyltransferase |  |
| mQTL-G22 |  | TG(18:1/18:2/18:3), TG(18:1/18:2/18:2) | 6, 3 | 18 | Marker935098, Marker840259 | 0.00~0.05 | 3.15~3.97 | 0.00~1.54 |  | *Glyma18g51100* | *GmPI4Kγ* | *AT2G03890* | No |  | Phosphatidylinositol-4-Kinase γ |  |
| mQTL-G23 |  | TG(18:3/18:3/18:3), DG(20:0/18:2) | 2, 6 | 18 | Marker990849, Marker846466 | 0.00~0.28 | 2.91~3.76 | 0.00~5.22 |  | *Glyma18g52590* | *GmECI1* | *AT1G65520* | Yes | gmx00071 | Enoyl CoA isomerase | miR156e, miR482c, miR862a, miR5037b, miR5044 |
| mQTL-G24 |  | TG(16:0/16:0/18:2) | 2 | 19 | Marker1540580, Marker1527795 | 0.09~0.14 | 2.96~3.96 | 3.02~8.18 |  |  |  |  |  |  |  |  |
| mQTL-G25 |  | TG(16:0/16:0/18:2) | 8, 3, 4 | 19 | Marker1522898, Marker1522898 | -0.10~-0.08 | 3.55~3.69 | 2.45~4.08 |  | *Glyma19g24531* | *GmMYB30* | *AT3G28910* | No |  | MYB Transcription Factors |  |
| mQTL-G26 |  | DG(20:0/18:2) | 1, 2, 3, 7 | 20 | Marker1335369, Marker1436813 | -0.30~0.20 | 3.22~4.16 | 2.47~6.88 |  | *Glyma20g02570* | *GmLCAT* | *AT4G19860* | Yes | gmx00592 | Lecithin:cholesterol acyltransferase |  |
| mQTL-G27 |  | TG(18:0/16:0/18:1), TG(18:3/18:2/18:2), TG(20:1/18:3/18:3) | 1, 3 | 20 | Marker1345857, Marker1343572 | -0.17~0.45 | 3.22~3.77 | 0.00~7.85 |  |  |  |  |  |  |  | miR10447 |
| mQTL-G28 |  | TG(20:2/18:2/18:2), SQDG(16:0/18:2) | 1, 2 | 1 | Marker1827676, Marker1830570 | 0.25~0.37 | 2.58~3.76 | 3.12~6.74 |  | *Glyma01g44600* | *GmOPR* | *AT1G76690* | No | gmx00592 | Oxo-Phytodienoic Acid Reductase | miR156t |
| mQTL-G29 |  | TG(18:3/18:2/18:2), SQDG(16:0/18:3), DG(20:0/18:2), TG(18:1/18:2/18:3) | 2, 8, 5, 4, 1, 6 | 20 | Marker1335669, Marker1357734 | -0.36~0.32 | 2.65~5.37 | 0.00~5.46 |  | *Glyma20g24530* | *GmFAD2* | *AT3G12120* | Yes | gmx01040 | Oleate Desaturase |  |
| mQTL-G30 |  | TG(18:0/18:0/18:1), DG(16:0/18:2), DG(20:1/18:3), TG(18:3/18:2/18:2) | 1, 7, 6 | 20 | Marker1309940, Marker1333431 | -0.19~0.00 | 2.59~5.44 | 0.00~4.90 |  | *Glyma20g25833* | *GmFatB3* | *AT1G08510* | No | gmx00061 |  | miR2118a, miR482b |
| mQTL-G31 |  | DG(20:1/18:3), DG(16:0/16:0), DG(18:0/16:0), DG(18:0/18:0) | 6, 2 | 20 | Marker1398370, Marker1417578 | -0.35~0.00 | 2.85~4.55 | 0.00~8.01 |  | *Glyma20g28200* | *GmLACS6* | *AT5G27600* | Yes | gmx00061 | Long-Chain Acyl-CoA Synthetase | miR167i, miR167e |
| mQTL-G32 |  | SQDG(16:0/18:1) | 5, 1 | 1 | Marker1804614, Marker1804614 | -0.41~-0.27 | 3.05~3.21 | 1.91~3.31 |  |  |  |  |  |  |  |  |
| mQTL-G33 |  | DG(16:0/16:0), DG(18:0/18:0) | 7, 4, 3, 5 | 20 | Marker1354208, Marker1354208 | 0.16~0.33 | 3.01~3.70 | 1.99~4.04 |  | *Glyma20g31030* | *GmCK* | *AT1G74320* | Yes | gmx00564 | Choline Kinase |  |
| mQTL-G34 |  | TG(16:0/18:1/18:2) | 1, 3 | 1 | Marker1865193, Marker1865193 | -0.23~-0.08 | 3.29~3.39 | 0.90~6.89 |  |  |  |  |  |  |  |  |
| mQTL-G35 |  | TG(20:1/18:3/18:3), TG(20:2/18:2/18:2) | 7, 3, 4, 5, 1 | 2 | Marker1229647, Marker1229647 | 0.10~0.32 | 2.83~3.37 | 1.73~4.98 |  |  |  |  |  |  |  |  |
| mQTL-G36 |  | TG(20:1/18:3/18:3), TG(18:0/18:1/18:1), DG(18:1/18:1) | 2, 1, 6 | 2 | Marker1167573, Marker1282727 | -0.25~-0.10 | 2.65~3.83 | 1.09~3.86 |  |  |  |  |  |  |  | miR482a, miR156j, miR9735, miR1512c, miR1512b |
| mQTL-G37 |  | SQDG(16:0/18:2), DG(16:0/16:0), DG(18:0/18:0), DG(18:1/18:2), DG(18:1/18:1), DG(16:0/18:1), DG(18:0/16:0), DG(18:0/18:1), DG(20:1/18:2), TG(18:1/18:2/18:3), TG(20:1/18:3/18:3), TG(20:2/18:2/18:2) | 3, 4, 1, 2 | 2 | Marker1210105, Marker1280678 | -0.47~0.39 | 2.58~6.23 | 1.96~13.49 |  | *Glyma02g11570* | *GmCer9* | *AT4G34100* | Yes | gmx04141 | E3 ubiquitin Ligase |  |
| mQTL-G38 |  | TG(18:1/18:1/18:2) | 2 | 1 | Marker1769068, Marker1891895 | 0.23~0.23 | 3.87~4.22 | 6.91~7.23 |  | *Glyma01g00710* | *GmPLA2* | *AT4G29460* | No | gmx00564 | Phospholipase A2 |  |
| mQTL-G39 |  | TG(16:0/18:2/18:3), TG(16:0/18:3/18:3) | 2, 1 | 2 | Marker1161043, Marker1241414 | -0.16~0.19 | 2.73~4.14 | 6.52~8.69 |  | *Glyma02g15600* | *GmFAB2* | *AT2G43710* | Yes | gmx01040 | Stearoyl-ACP Desaturase |  |
| mQTL-G40 |  | DGDG(16:0/18:2) | 2, 7 | 3 | Marker2485779, Marker2406610 | -0.21~1.44 | 3.24~31.92 | 3.49~10.22 |  | *Glyma03g22860* | *GmnsPLC* | *AT3G03520* | No | gmx00564 | Phospholipase C |  |
| mQTL-G41 |  | TG(16:0/18:1/18:3) | 4, 5 | 3 | Marker2508846, Marker2508846 | 0.17~0.22 | 3.07~3.35 | 1.44~3.60 |  | *Glyma03g39540* | *GmTAZ* | *AT3G05510* | No | gmx00564 | Cardiolipin Transacylase | miR164f |
| mQTL-G42 |  | DG(20:1/18:2), DG(20:1/18:3) | 6, 7, 3, 5 | 1 | Marker1818868, Marker1818868 | 0.00~0.13 | 3.01~3.83 | 0.00~4.04 |  |  |  |  |  |  |  |  |
| mQTL-G43 |  | TG(18:1/18:2/18:3) | 2 | 5 | Marker2192021, Marker2191229 | 0.07~0.09 | 2.93~4.30 | 3.15~4.92 |  | *Glyma05g04940* | *GmACX1* | *AT4G16760* | Yes | gmx00592 | Acyl-CoA Oxidase |  |
| mQTL-G44 |  | DG(16:0/16:0), DG(18:0/18:0) | 2 | 5 | Marker2117205, Marker2117678 | -0.19~-0.19 | 2.71~2.93 | 2.85~3.17 |  | *Glyma05g05290* | *GmBI1* | *AT5G47120* | No | gmx00600 | Bax inhibitor-1 |  |
| mQTL-G45 |  | DG(16:0/16:0), DG(18:0/16:0) | 6 | 5 | Marker2172599, Marker2172599 | 0.15~0.15 | 3.00~3.98 | 1.30~1.53 |  |  |  |  |  |  |  |  |
| mQTL-G46 |  | TG(16:0/18:3/18:3) | 1, 2 | 5 | Marker2086805, Marker2154969 | -0.16~0.16 | 2.89~2.92 | 5.70~5.78 |  |  |  |  |  |  |  |  |
| mQTL-G47 |  | TG(16:0/18:2/18:3), TG(16:0/18:1/18:3), TG(20:1/18:2/18:3), TG(18:1/18:1/18:2) | 2, 1, 6, 7 | 5 | Marker2187077, Marker2171016 | -0.19~0.16 | 2.56~3.93 | 0.01~8.06 |  | *Glyma05g26141* | *GmGPAT9* | *AT5G60620* | Yes | gmx00561 | Glycerol-3-Phosphate Acyl Transferase 9 |  |
| mQTL-G48 |  | TG(20:1/18:2/18:2), TG(20:2/18:2/18:2), TG(18:1/18:2/18:3), TG(18:1/18:1/18:2), TG(18:1/18:1/18:3) | 2, 1 | 5 | Marker2152218, Marker2124082 | -0.39~0.47 | 2.69~4.71 | 2.98~11.20 |  | *Glyma05g31270* | *GmCTS* | *AT4G39850* | Yes | gmx00073 | ABC Acyl Transporter |  |
| mQTL-G49 |  | TG(20:1/18:3/18:3), TG(20:2/18:2/18:2) | 2 | 5 | Marker2104700, Marker2163727 | -0.42~-0.40 | 3.00~3.76 | 6.27~11.08 |  | *Glyma05g31630* | *GmAGT* | *AT4G39660* | No | gmx00410 | Alanine-glyoxylate aminotransferase |  |
| mQTL-G50 |  | TG(18:0/18:0/18:1), TG(18:0/18:1/18:1), TG(20:0/18:1/18:1) | 2 | 1 | Marker1934440, Marker1784992 | 0.18~0.27 | 2.86~3.13 | 6.61~8.41 |  |  |  |  |  |  |  |  |
| mQTL-G51 |  | TG(18:1/18:1/18:2), TG(20:1/18:1/18:2), TG(16:0/18:1/18:2), SQDG(16:0/18:2) | 7, 2, 5, 3, 6 | 6 | Marker1991901, Marker1969292 | -0.33~0.22 | 2.62~4.27 | 1.46~8.53 |  | *Glyma06g01240* | *GmbZIP123* | *AT4G34590* | Yes |  | Basic region leucine zipper | miR394g |
| mQTL-G52 |  | TG(18:0/16:0/18:1), TG(18:2/18:2/18:2), TG(18:0/18:0/18:1) | 2, 6 | 6 | Marker1969292, Marker1968397 | 0.00~0.17 | 2.73~3.40 | 0.00~6.06 |  | *Glyma06g04230* | *GmPAH2* | *AT3G09560* | Yes | gmx00561 | Phosphatidate Phosphatase |  |
| mQTL-G53 |  | TG(18:1/18:2/18:2) | 7 | 6 | Marker1978197, Marker2048040 | 0.00~0.00 | 3.12~4.33 | 5.63~11.43 |  |  |  |  |  |  |  | miR1520h |
| mQTL-G54 |  | TG(20:1/18:2/18:2), TG(18:1/18:1/18:2) | 1, 6 | 6 | Marker2044143, Marker2048823 | 0.00~0.25 | 2.51~3.73 | 0.00~6.50 |  | *Glyma06g45941* | *GmLOX3* | *AT2G46410* | No | gmx00561 | Lipoxygenase | miR10200 |
| mQTL-G55 |  | TG(18:1/18:1/18:2) | 2, 1 | 6 | Marker1980245, Marker1965534 | -0.27~0.25 | 2.73~3.60 | 7.10~8.43 |  | *Glyma06g47300* | *GmALA1* | *AT5G04930* | No |  | Aminophospholipid ATPase |  |
| mQTL-G56 |  | DG(18:0/18:0), DG(16:0/16:0), DG(18:0/16:0), DG(18:0/18:1), DG(18:1/18:1) | 7, 2, 5, 4, 6, 3, 8 | 1 | Marker1806679, Marker1775184 | -0.23~0.54 | 3.25~5.88 | 0.00~7.25 |  | *Glyma01g22850* | *GmWBC14* | *AT3G25620* | No | gmx00073 | ABC Transporter | miR10407a |
| mQTL-G57 |  | DGDG(18:3/18:3), TG(16:0/18:1/18:2), TG(18:1/18:1/18:2), DGDG(16:0/18:3), SQDG(16:0/18:1), SQDG(16:0/18:2) | 2, 1 | 1 | Marker1827971, Marker1827971 | -0.23~0.71 | 2.50~3.36 | 2.98~7.39 |  |  |  |  |  |  |  | miR4367 |
| mQTL-G58 |  | TG(18:1/18:1/18:3) | 3, 2 | 7 | Marker357402, Marker377605 | -0.08~0.14 | 2.69~3.09 | 0.94~3.17 |  | *Glyma07g31660* | *GmLOX3* | *AT1G17420* |  | gmx00592 | Lipoxygenase 3 |  |
| mQTL-G59 |  | TG(16:0/18:1/18:2) | 1, 7 | 7 | Marker373127, Marker370963 | -0.15~-0.09 | 2.89~3.28 | 2.98~3.39 |  | *Glyma07g37570* | *GmMFP* | *AT3G06860* | Yes | gmx00592 | Multifunctional Protein |  |
| mQTL-G60 |  | DG(16:0/16:0), DG(18:0/16:0), DG(18:0/18:0), TG(16:0/18:1/18:2) | 2, 1, 4, 3, 5, 8 | 7 | Marker281134, Marker325634 | -0.36~0.44 | 2.72~5.66 | 2.02~8.19 |  | *Glyma07g39481* | *GmLPLA* | *AT4G22300* | No | gmx00564 | Lysophospholipase | miR1514a, miR1513a, miR171c |
| mQTL-G61 |  | DG(18:0/16:0), DG(18:0/18:0), TG(18:1/18:1/18:1) | 2, 4, 5, 7 | 7 | Marker332117, Marker326274 | -0.23~0.36 | 3.10~3.41 | 2.33~4.85 |  | *Glyma07g40360* | *GmPI5P-II* | *AT2G43900* | No |  | Phosphoinositide 5-Phosphatase Type II |  |
| mQTL-G62 |  | DG(18:0/18:0), DG(18:0/16:0), TG(18:1/18:1/18:2) | 1, 6 | 8 | Marker786036, Marker747366 | 0.11~0.33 | 2.63~3.82 | 1.45~7.28 |  | *Glyma08g07871* | *GmHAD* | *AT5G10160* | No | gmx00780 | Hydroxyacyl-ACP Dehydrase |  |
| mQTL-G63 |  | DGDG(16:0/18:2) | 1, 2 | 8 | Marker791165, Marker718573 | -0.39~0.36 | 2.57~3.53 | 0.76~6.38 |  |  |  |  |  |  |  |  |
| mQTL-G64 |  | TG(16:0/18:2/18:2) | 2, 1 | 8 | Marker685772, Marker685772 | -0.16~0.16 | 2.68~2.73 | 7.28~7.62 |  |  |  |  |  |  |  |  |
| mQTL-G65 |  | TG(18:1/18:1/18:2), TG(20:1/18:2/18:2), TG(18:3/18:3/18:3), TG(20:2/18:2/18:2) | 7, 2, 1 | 8 | Marker787649, Marker802731 | -0.40~0.16 | 2.57~4.40 | 1.81~7.84 |  | *Glyma08g41120* | *GmSLD2* | *AT2G46210* | No | gmx00600 | Sphingobase-D8 Desaturase |  |
| mQTL-G66 |  | DG(18:1/18:1), SQDG(16:0/18:1), SQDG(16:0/18:3) | 5, 7, 4, 3 | 9 | Marker474932, Marker445705 | -0.25~0.01 | 3.18~3.61 | 0.00~4.73 |  | *Glyma09g00960* | *GmMCD* | *AT4G04320* | No | gmx00410 | Malonyl-CoA Decarboxylase |  |
| mQTL-G67 |  | TG(18:1/18:2/18:3), DG(20:0/18:2) | 7, 3, 6 | 9 | Marker446445, Marker448313 | 0.00~0.02 | 3.04~3.86 | 0.00~1.01 |  |  |  |  |  |  |  |  |
| mQTL-G68 |  | DG(20:0/18:2) | 1, 2 | 9 | Marker412139, Marker440943 | -0.30~0.28 | 3.09~4.01 | 3.35~6.14 |  | *Glyma09g32750* | *GmTAZ* | *AT1G78690* | No | gmx00564 | Cardiolipin Transacylase |  |
| mQTL-G69 |  | TG(18:1/18:2/18:3), DG(18:1/18:2) | 6 | 9 | Marker426761, Marker522000 | 0.00~0.00 | 3.11~3.17 | 0.00~0.00 |  | *Glyma09g40640* | *GmPECT1* | *AT2G38670* | No | gmx00564 | CTP:phosphorylethanolamine cytidyltransferase |  |
| mQTL-G70 |  | DG(16:0/16:0), DG(18:0/16:0), DG(18:0/18:0) | 5, 4, 7, 8, 6, 3, 1 | 10 | Marker1096354, Marker1096354 | 0.00~0.42 | 2.91~4.66 | 0.00~5.05 |  |  |  |  |  |  |  |  |
| mQTL-G71 |  | TG(16:0/16:0/18:2), TG(18:1/18:2/18:3), DG(16:0/18:2), TG(18:0/18:0/18:1) | 2, 1 | 10 | Marker1118083, Marker1030067 | -0.22~0.22 | 2.52~3.75 | 1.12~6.15 |  | *Glyma10g01770* | *GmLAH* | *AT3G62590* | No | gmx00561 | Lipid Acylhydrolase-like | miR164b |
| mQTL-G72 |  | TG(18:0/18:0/18:1), TG(20:0/18:1/18:1), TG(18:0/18:1/18:1), DG(16:0/16:0), TG(16:0/16:0/18:2) | 1, 2, 6 | 10 | Marker1030067, Marker1080126 | -0.22~0.17 | 2.92~3.62 | 0.95~8.10 |  | *Glyma10g04060* | *GmLAH* | *AT2G42450* | No | gmx00561 | Lipid Acylhydrolase-like | miR167d |
| mQTL-G73 |  | TG(20:2/18:2/18:2), DGDG(18:3/18:3) | 2 | 10 | Marker1015006, Marker1132356 | -0.21~0.67 | 2.52~2.79 | 5.02~6.32 |  | *Glyma10g35170* | *GmSDP6* | *AT3G10370* | No | gmx00564 | FAD-dependent Glycerol-3-Phosphate Dehydrogenase | miR9766, miR10186g |
| mQTL-G74 |  | TG(16:0/18:1/18:1), TG(16:0/18:1/18:2), TG(18:1/18:1/18:2) | 4, 5, 3, 1, 2 | 10 | Marker1069694, Marker1006569 | -0.23~0.26 | 2.65~3.96 | 1.47~7.52 |  | *Glyma10g38660* | *GmKCS6* | *AT1G68530* | No | gmx00062 | Ketoacyl-CoA Synthase | miR393i, miR4388, miR399a, miR399m |
| mQTL-G75 |  | SQDG(16:0/18:1), TG(18:1/18:2/18:2) | 1, 2, 3 | 10 | Marker1056155, Marker1059474 | -0.32~0.03 | 2.53~3.55 | 0.65~6.74 |  |  |  |  |  |  |  | miR167f, miR167h |
| mQTL-G76 |  | TG(18:1/18:1/18:3), DG(18:3/18:3) | 1, 3 | 10 | Marker1101150, Marker1024375 | -0.08~0.16 | 2.58~3.07 | 0.99~5.07 |  | *Glyma10g40910* | *GmTAGL* | *AT2G15230* | Yes | gmx00561 | Triacylglycerol Lipase (TAGL) | miR319f, miR10190, miR482d |
| mQTL-G77 |  | TG(16:0/16:0/18:3), TG(16:0/18:2/18:3), TG(20:0/18:1/18:1) | 1, 2 | 11 | Marker580107, Marker580107 | -0.16~0.17 | 2.68~3.90 | 6.79~7.98 |  | *Glyma11g03800* | *GmACX1* | *AT4G16760* | Yes | gmx00592 | Acyl-CoA Oxidase |  |
| mQTL-G78 |  | TG(20:0/18:1/18:2), TG(20:2/18:2/18:2), DG(18:1/18:2), TG(18:1/18:2/18:3) | 6, 1 | 11 | Marker655069, Marker664607 | -0.03~0.39 | 3.22~4.60 | 0.00~7.62 |  | *Glyma11g07590* | *GmLPP-ε2* | *AT5G66450* | No | gmx00564 | Phosphatidate Phosphatase | miR1520p, miR1520j |
| mQTL-G79 |  | TG(18:2/18:2/18:2), TG(18:3/18:2/18:2), TG(20:0/18:1/18:2), TG(16:0/16:0/18:1) | 2, 1 | 11 | Marker652526, Marker579785 | -0.17~0.17 | 2.56~5.57 | 5.11~10.74 |  | *Glyma11g10640* | *GmFAH* | *AT5G23190* | No | gmx00073 | Fatty Acyl omega-Hydroxylase | miR5380a, miR5380b |
| mQTL-G80 |  | TG(16:0/18:3/18:3), TG(18:0/16:0/18:1) | 6, 2 | 11 | Marker582376, Marker659557 | 0.00~0.16 | 3.27~3.87 | 0.00~5.42 |  | *Glyma11g13300* | *GmVIPP1* | *AT1G65260* | No |  | Vesicle-Inducing Protein in Plastids | miR828a |
| mQTL-G81 |  | TG(18:0/18:1/18:1), TG(20:0/18:1/18:2) | 4, 6, 1 | 11 | Marker609435, Marker635459 | -0.20~-0.01 | 2.96~3.29 | 0.02~3.89 |  | *Glyma11g33660* | *GmLAH* | *AT5G37710* | No | gmx00561 | Lipid Acylhydrolase-like |  |
| mQTL-G82 |  | TG(18:0/18:1/18:1) | 1, 2 | 11 | Marker609435, Marker635459 | -0.17~0.17 | 2.70~2.89 | 3.23~3.78 |  |  |  |  |  |  |  | miR172e |
| mQTL-G83 |  | DGDG(16:0/18:2), TG(18:2/18:2/18:2), TG(18:3/18:2/18:2) | 1, 3, 4, 5 | 12 | Marker2710716, Marker2652335 | -0.40~-0.06 | 3.21~4.16 | 1.75~9.01 |  | *Glyma12g00320* | *GmPIP* | *AT1G22620* | No | gmx00562 | Sac domain-containing Phosphoinositide Phosphatase |  |
| mQTL-G84 |  | TG(18:2/18:2/18:2), DGDG(16:0/18:2), DG(20:0/18:2), TG(18:3/18:2/18:2) | 2, 1, 7, 4, 3 | 12 | Marker2655259, Marker2712962 | -0.33~0.12 | 2.56~4.52 | 1.91~5.57 |  | *Glyma12g02300* | *GmABCG15* | *AT3G21090* | No | gmx00073 | ABC Transporter | miR5762 |
| mQTL-G85 |  | TG(18:3/18:2/18:2), TG(18:0/16:0/18:1), TG(18:2/18:2/18:2), TG(20:0/18:1/18:2) | 7, 1, 3, 6 | 12 | Marker2694823, Marker2648174 | -0.16~0.00 | 3.07~5.35 | 0.13~7.69 |  | *Glyma12g03450* | *GmGIPC* | *AT4G38690* | No | gmx00564 | Glycosylphosphatidylinositol-specific Phospholipase C |  |
| mQTL-G86 |  | TG(18:2/18:2/18:2) | 2, 1 | 12 | Marker2662588, Marker2679795 | -0.21~0.21 | 3.47~5.65 | 5.06~10.26 |  |  |  |  |  |  |  |  |
| mQTL-G87 |  | TG(16:0/18:3/18:3), TG(18:0/16:0/18:1), TG(18:0/18:0/18:1), TG(20:0/18:1/18:2), TG(20:0/18:1/18:1), DGDG(16:0/18:2) | 1, 2 | 12 | Marker2719781, Marker2719781 | -0.23~0.37 | 3.09~7.54 | 3.76~11.31 |  | *Glyma12g05330* | *GmVIPP1* | *AT1G65260* | No |  | Vesicle-Inducing Protein in Plastids |  |
| mQTL-G88 |  | TG(16:0/18:3/18:3), TG(20:1/18:3/18:3), TG(16:0/16:0/18:1), TG(16:0/16:0/18:3), TG(18:0/16:0/18:1), TG(18:0/18:0/18:1), TG(16:0/18:1/18:1), TG(18:0/18:1/18:1), TG(20:0/18:1/18:2), TG(16:0/18:2/18:3), TG(18:3/18:2/18:3), TG(18:1/18:2/18:2), TG(18:1/18:1/18:1) | 2, 1, 7, 6, 3 | 12 | Marker2675339, Marker2635450 | -0.26~0.44 | 2.57~6.95 | 0.00~11.78 |  |  |  |  |  |  |  | miR10424b |
| mQTL-G89 |  | DG(20:1/18:2), DG(20:1/18:3) | 3, 5 | 1 | Marker1905701, Marker1905701 | 0.00~0.12 | 3.17~3.57 | 0.00~2.62 |  |  |  |  |  |  |  |  |
| mQTL-G90 |  | TG(16:0/18:2/18:3), TG(18:3/18:2/18:3), TG(20:0/18:1/18:2), TG(18:1/18:1/18:1) | 6, 7, 5, 8, 3, 1, 2 | 12 | Marker2729868, Marker2734590 | -0.25~0.26 | 3.09~3.86 | 0.00~9.02 |  |  |  |  |  |  |  |  |
| mQTL-G91 |  | TG(16:0/18:2/18:3), TG(18:1/18:1/18:1) | 1, 3 | 12 | Marker2696379, Marker2729534 | 0.12~0.17 | 3.16~3.49 | 1.58~6.32 |  | *Glyma12g09270* | *GmCER4* | *AT4G33790* | No | gmx00073 | alcohol-forming fatty acyl-CoA reductase | miR172a, miR530d |
| mQTL-G92 |  | TG(16:0/18:2/18:3), TG(18:0/16:0/18:1), TG(18:3/18:2/18:3), TG(16:0/18:1/18:3) | 2, 1, 7, 3, 4, 8 | 12 | Marker2624957, Marker2692248 | -0.26~0.26 | 3.14~3.95 | 1.46~9.64 |  |  |  |  |  |  |  |  |
| mQTL-G93 |  | DG(16:0/16:0), DG(18:0/16:0), DG(18:0/18:0) | 2 | 1 | Marker1929229, Marker1918824 | -0.22~-0.20 | 3.12~4.23 | 3.86~4.61 |  |  |  |  |  |  |  |  |
| mQTL-G94 |  | DG(20:1/18:2), TG(18:0/16:0/18:1), DG(18:3/18:3) | 6, 2, 7 | 12 | Marker2668097, Marker2705284 | -0.12~0.00 | 3.07~3.75 | 0.00~3.16 |  | *Glyma12g11150* | *GmDREBL* | *AT2G40340* | No |  | AP2 domain | miR4384 |
| mQTL-G95 |  | DG(20:0/18:2), TG(18:1/18:2/18:2) | 6, 7 | 12 | Marker2737432, Marker2643431 | 0.00~0.00 | 3.12~5.67 | 0.00~1.50 |  |  |  |  |  |  |  | miR10428 |
| mQTL-G96 |  | TG(20:1/18:2/18:2) | 3, 4, 5, 7 | 12 | Marker2623805, Marker2623805 | 0.16~0.31 | 3.36~4.08 | 2.38~5.15 |  |  |  |  |  |  |  |  |
| mQTL-G97 |  | TG(20:1/18:2/18:2) | 2 | 12 | Marker2648049, Marker2648049 | -0.18~-0.16 | 2.50~3.09 | 2.89~3.31 |  |  |  |  |  |  |  | miR1520g, miR172k |
| mQTL-G98 |  | TG(18:0/18:0/18:1), TG(18:0/18:1/18:1), TG(18:0/16:0/18:1) | 3, 4, 2 | 13 | Marker2824750, Marker2837109 | -0.15~0.16 | 2.60~3.94 | 1.04~4.30 |  | *Glyma13g01080* | *GmCL1* | *AT3G21240* | No | gmx00940 | 4-Coumarate-CoA Ligase |  |
| mQTL-G99 |  | TG(16:0/18:2/18:3), TG(16:0/18:3/18:3) | 2 | 13 | Marker2853160, Marker2861579 | -0.17~-0.14 | 2.54~2.90 | 5.20~7.51 |  | *Glyma13g11700* | *GmLACS8* | *AT2G04350* | No | gmx00061 | Long-Chain Acyl-CoA Synthetase |  |
| mQTL-G100 |  | TG(16:0/18:2/18:3), TG(20:1/18:3/18:3), TG(18:3/18:3/18:3), TG(20:2/18:2/18:2) | 6, 2, 1 | 13 | Marker2790748, Marker2850221 | -0.39~0.32 | 2.83~5.40 | 0.02~10.08 |  | *Glyma13g16560* | *GmDGAT1a* | *AT2G19450* | Yes | gmx00561 | Acyl-CoA : Diacylglycerol Acyltransferase | miR156q |
| mQTL-G101 |  | TG(20:0/18:1/18:1), TG(18:0/18:0/18:1), TG(20:0/18:1/18:2), TG(18:0/18:1/18:1) | 2, 1, 6, 3, 4, 5, 7 | 13 | Marker2762711, Marker2855328 | -0.15~0.32 | 2.50~5.70 | 1.93~10.20 |  | *Glyma13g17860* | *GmDGAT3* | *AT1G48300* | Yes | gmx00561 | Acyl-CoA : Diacylglycerol Acyltransferase | miR9725 |
| mQTL-G102 |  | TG(18:0/16:0/18:1), TG(18:0/18:0/18:1), TG(18:0/18:1/18:1) | 2, 3 | 13 | Marker2849746, Marker2815423 | -0.33~0.16 | 2.96~6.56 | 2.52~10.40 |  | *Glyma13g19730* | *GmPAH1* | *AT5G42870* | Yes | gmx00561 | Phosphatidate Phosphatase | miR160d |
| mQTL-G103 |  | TG(18:0/18:0/18:1), TG(18:0/18:1/18:1) | 4, 8, 5, 3, 7 | 1 | Marker1918392, Marker1851699 | -0.31~-0.12 | 3.12~4.68 | 3.25~5.25 |  |  |  |  |  |  |  | miR4374a, miR393b |
| mQTL-G104 |  | TG(20:1/18:3/18:3) | 2, 4, 5, 8, 1 | 13 | Marker2834105, Marker2836475 | -0.26~0.42 | 3.09~3.47 | 2.15~3.68 |  | *Glyma13g28710* | *GmTAGL* | *AT5G18640* | Yes | gmx00561 | Triacylglycerol Lipase (TAGL) |  |
| mQTL-G105 |  | TG(16:0/16:0/18:3), TG(16:0/18:3/18:3), TG(18:0/16:0/18:1) | 8, 7, 1, 2 | 14 | Marker1626292, Marker1701754 | -0.65~0.12 | 2.72~57.49 | 0.24~32.94 |  | *Glyma14g06290* |  | *AT2G40220* | Yes | gmx00561 | Abscisic Acid Insensitive (ABI) transcription factors |  |
| mQTL-G106 |  | DGDG(16:0/18:2), SQDG(16:0/18:1) | 1, 5 | 14 | Marker1722968, Marker1647615 | -0.42~-0.24 | 3.24~3.53 | 2.19~3.64 |  | *Glyma14g08010* | *GmEK* | *AT2G26830* | No | gmx00564 | Ethanolamine Kinase | miR169l, miR172d |
| mQTL-G107 |  | DG(20:1/18:3), DG(20:0/18:3) | 7 | 1 | Marker1919401, Marker1920209 | 0.00~0.00 | 3.31~5.30 | 1.41~1.68 |  |  |  |  |  |  |  |  |
| mQTL-G108 |  | DG(16:0/16:0), DG(18:0/16:0), DG(18:0/18:0) | 2 | 15 | Marker29446, Marker129454 | 0.20~0.24 | 3.26~4.77 | 3.80~5.16 |  | *Glyma15g01350* | *GmLPLA* | *AT1G52700* | No | gmx00564 | Lysophospholipase |  |
| mQTL-G109 |  | DG(16:0/16:0), DG(18:0/16:0), DG(18:0/18:0), DG(18:3/18:3) | 8, 3, 4, 7, 6, 1, 2 | 15 | Marker29446, Marker5035 | -0.27~0.26 | 2.56~5.36 | 0.24~5.73 |  | *Glyma15g02710* | *GmPLDε1* | *AT1G55180* | No | gmx04144 | Phospholipase D ε |  |
| mQTL-G110 |  | TG(20:1/18:2/18:2) | 8, 3 | 1 | Marker1847458, Marker1847458 | -0.09~-0.09 | 3.52~3.82 | 0.82~0.82 |  | *Glyma01g32190* | *GmLP* | *AT2G38530* | No | gmx00073 | Lipid Transfer Protein |  |
| mQTL-G111 |  | DG(16:0/18:1), DG(18:0/18:1) | 6, 7 | 1 | Marker1896737, Marker1896737 | 0.12~0.13 | 3.07~3.86 | 1.63~3.01 |  |  |  |  |  |  |  |  |
| mQTL-G112 |  | DG(16:0/18:1), DG(16:0/18:2), DG(18:1/18:2), DG(20:1/18:2), TG(18:1/18:2/18:3) | 3, 6, 7, 1, 2, 5, 8 | 15 | Marker107799, Marker23766 | -0.19~0.39 | 2.81~17.32 | 0.00~6.87 |  | *Glyma15g05470* | *GmSWEET10a* | *AT5G13170* |  | gmx00500 | Sugar transporter | miR166j, miR394d, miR3522 |
| mQTL-G113 |  | DG(20:0/18:2), DG(20:1/18:3), DG(20:1/18:2) | 2 | 1 | Marker1864840, Marker1792642 | -0.23~-0.20 | 2.75~2.91 | 7.60~8.43 |  | *Glyma01g36011* | *GmDGAT* | *AT3G51520* | Yes | gmx00561 | Acyl-CoA : Diacylglycerol Acyltransferase,Monoacylglycerol Acyltransferase | miR168b, miR4393b |
| mQTL-G114 |  | TG(16:0/18:1/18:3) | 1, 2 | 15 | Marker119263, Marker25552 | -0.23~0.23 | 3.37~3.64 | 3.49~6.72 |  | *Glyma15g16950* | *GmCCT1* | *AT2G32260* | Yes | gmx00564 | Choline-Phosphate Cytidylyltransferase | miR10434 |
| mQTL-G115 |  | DG(16:0/16:0), DG(20:0/18:2), DG(20:0/18:3), TG(18:1/18:2/18:3), DG(18:0/16:0), DG(18:0/18:0), DG(18:1/18:1) | 2, 1 | 1 | Marker1801142, Marker1822692 | -0.44~0.46 | 2.62~4.71 | 5.92~10.87 |  | *Glyma01g42420* | *GmPLDγ* | *AT2G42010* | Yes | gmx00564 | Phospholipase D γ | miR1529, miR5031 |
| mQTL-G116 |  | SQDG(16:0/18:3), SQDG(16:0/18:2) | 1 | 15 | Marker50563, Marker57754 | -0.41~-0.32 | 2.90~2.92 | 7.67~7.70 |  |  |  |  |  |  |  |  |
| mQTL-G117 |  | DGDG(16:0/18:3) | 2, 1 | 16 | Marker2612063, Marker2522737 | -0.77~0.53 | 2.57~2.60 | 6.89~7.08 |  | *Glyma16g06230* | *GmTAGL* | *AT5G14180* | Yes | gmx00561 | Triacylglycerol lipase | miR156c, miR171g, miR4409, miR5372, miR1526 |
| mQTL-G118 |  | TG(20:0/18:1/18:1), TG(20:0/18:1/18:2) | 3 | 16 | Marker2525834, Marker2568647 | 0.06~0.07 | 3.22~3.90 | 0.85~1.43 |  | *Glyma16g07800* | *GmOBO* | *AT3G01570* | Yes | gmx00561 | Oil-Body Oleosin |  |
| mQTL-G119 |  | TG(20:1/18:1/18:2) | 5, 3, 6 | 16 | Marker2564998, Marker2564998 | 0.09~0.18 | 3.35~4.23 | 2.71~2.88 |  | *Glyma18g42280* | *Gmα-CT* | *AT2G38040* | No | gmx00061 | Carboxyltransferase alpha Subunit of Heteromeric ACCase |  |
| mQTL-G120 |  | DG(16:0/16:0), DG(18:0/16:0), DG(18:0/18:0), DGDG(16:0/18:3), SQDG(16:0/18:2), SQDG(16:0/18:3) | 2, 4, 1 | 16 | Marker2568135, Marker2542716 | -0.44~-0.19 | 2.60~3.67 | 2.67~7.10 |  |  |  |  |  |  |  | miR5668 |
| mQTL-G121 |  | SQDG(16:0/18:2), SQDG(16:0/18:3) | 5, 8 | 16 | Marker2603205, Marker2603205 | -0.44~0.00 | 3.14~3.68 | 0.00~2.40 |  |  |  |  |  |  |  | miR9729 |
| mQTL-G122 |  | TG(18:0/18:1/18:1), TG(18:1/18:2/18:2) | 1, 7 | 17 | Marker240323, Marker227763 | -0.23~-0.02 | 3.16~3.73 | 2.76~5.32 |  | *Glyma17g04650* | *GmDGAT3* | *AT1G48300* | Yes | gmx00561 | Acyl-CoA : Diacylglycerol Acyltransferase |  |
| mQTL-G123 |  | TG(18:0/18:0/18:1), TG(18:0/18:1/18:1) | 2 | 17 | Marker220014, Marker227763 | 0.17~0.25 | 2.79~3.86 | 4.36~5.84 |  |  |  |  |  |  |  |  |
| mQTL-GP1 | **Glycerophospholipids** | PE(18:3/18:2), PE(18:3/18:3) | 2, 1 | 17 | Marker200629, Marker166189 | -1.62~1.67 | 2.57~2.61 | 6.91~7.43 |  |  |  |  |  |  |  |  |
| mQTL-GP2 |  | PE(18:3/18:3), PE(18:2/18:2) | 1, 2 | 17 | Marker200629, Marker200629 | -0.38~1.58 | 2.59~2.68 | 3.18~6.93 |  | *Glyma17g12940* | *GmFatB1* | *AT1G08510* | Yes | gmx00061 | Acyl-ACP Thioesterase B | miR396i, miR396d, miR171l, miR319l |
| mQTL-GP3 |  | CL(18:2/18:1/18:1/18:2) | 1, 2 | 17 | Marker223571, Marker223571 | -0.32~0.31 | 2.80~2.98 | 5.15~5.80 |  | *Glyma17g13280* | *GmTL* | *AT1G68710* | No |  | Translocase | miR162c |
| mQTL-GP4 |  | PE(20:0/18:3), PE(20:0/18:2) | 1, 7, 6 | 17 | Marker219784, Marker219784 | 0.00~0.35 | 2.61~3.85 | 0.00~0.96 |  | *Glyma17g35480* | *GmACBP3* | *AT4G24230* | Yes | gmx00564 | Acyl CoA Binding Protein |  |
| mQTL-GP5 |  | LPE(16:0), CL(18:2/18:1/18:1/18:2), CL(18:2/18:2/18:2/18:1), CL(18:2/18:2/18:2/18:2), CL(18:3/18:2/18:2/18:2) | 4, 1, 2 | 18 | Marker982150, Marker864664 | -0.39~0.72 | 2.78~3.72 | 4.06~10.93 |  | *Glyma18g00381* | *GmLTP* | *AT1G55260* | No | gmx00073 | Lipid Transfer Protein |  |
| mQTL-GP6 |  | LPE(16:0) | 1, 2 | 18 | Marker867529, Marker939037 | -0.46~0.35 | 2.84~4.26 | 7.44~11.46 |  | *Glyma18g02210* | *GmMIPS* | *AT2G22240* | Yes | gmx00562 | myo-inositol-3-phosphate synthase |  |
| mQTL-GP7 |  | CL(18:2/18:1/18:1/18:2), CL(18:2/18:2/18:2/18:1), CL(18:2/18:2/18:2/18:2), PE(20:0/18:1) | 2 | 18 | Marker817771, Marker827362 | 0.28~0.34 | 2.81~3.49 | 5.85~7.12 |  | *Glyma18g03090* | *GmPIPLC* | *AT3G08510* | Yes | gmx04070 | Phosphoinositide-specific Phospholipase C | miR393f |
| mQTL-GP8 |  | PE(20:0/18:2), PG(16:0/18:2), PI(16:0/18:2) | 7, 6 | 18 | Marker913530, Marker967018 | 0.00~0.00 | 3.56~4.70 | 0.00~1.29 |  |  |  |  |  |  |  |  |
| mQTL-GP9 |  | PI(16:0/18:3), PI(18:1/18:1) | 1, 2 | 1 | Marker1922682, Marker1849486 | -0.22~0.28 | 2.50~2.74 | 6.49~8.02 |  | *Glyma01g43470* | *GmLACS4* | *AT4G23850* | Yes | gmx00061 | Long-Chain Acyl-CoA Synthetase |  |
| mQTL-GP10 |  | PG(18:0/18:2), PG(18:1/18:2), PI(18:1/18:2), PI(18:0/18:1) | 2, 8, 4, 7, 1, 5, 6 | 20 | Marker1320978, Marker1424697 | -0.26~0.48 | 2.63~4.86 | 0.68~29.37 |  | *Glyma20g02570* | *GmLCAT* | *AT4G19860* | Yes | gmx00592 | Lecithin:cholesterol acyltransferase |  |
| mQTL-GP11 |  | CL(18:2/18:1/18:1/18:2), CL(18:2/18:2/18:2/18:1), LPE(18:1), PG(16:0/18:1), CL(18:2/18:2/18:2/18:2) | 2, 1, 5, 8, 4, 3, 7 | 1 | Marker1907045, Marker1804614 | -0.52~0.29 | 2.68~4.15 | 1.81~5.46 |  |  |  |  |  |  |  |  |
| mQTL-GP12 |  | PE(20:0/18:2), PI(16:0/18:2), PG(18:0/16:0), PG(16:0/16:0), LPE(18:2) | 2 | 20 | Marker1430475, Marker1417801 | -0.45~0.38 | 2.69~4.38 | 3.50~9.62 |  |  |  |  |  |  |  | miR2606a |
| mQTL-GP13 |  | LPE(18:2), PE(20:0/18:2), PG(16:0/16:0), PG(18:0/16:0), PG(16:0/18:1), PG(16:0/18:2), PI(16:0/18:2), PE(18:1/18:2), PE(18:3/18:3), PE(20:0/18:3), PE(18:3/18:2) | 4, 5, 3, 8, 7, 2, 1, 6 | 20 | Marker1413435, Marker1426291 | -1.04~0.49 | 2.65~10.32 | 0.00~8.29 |  | *Glyma20g24530* | *GmFAD2* | *AT3G12120* | Yes | gmx01040 | Oleate Desaturase |  |
| mQTL-GP14 |  | PE(20:0/18:3), PE(16:0/18:2), PG(18:0/16:0) | 6, 2, 3 | 20 | Marker1448987, Marker1347546 | -0.38~0.16 | 3.21~5.31 | 2.41~6.28 |  | *Glyma20g25816* | *GmPSS* | *AT1G15110* | Yes | gmx00564 | Base-Exchange-type Phosphatidylserine Synthase | miR2118a, miR482b |
| mQTL-GP15 |  | PE(16:0/18:2), PE(18:3/18:2), PE(18:3/18:3), PE(20:0/18:2) | 6, 3, 7, 1 | 20 | Marker1412383, Marker1314287 | 0.00~1.20 | 2.51~5.33 | 0.00~6.07 |  | *Glyma20g28200* | *GmLACS6* | *AT5G27600* | Yes | gmx00561 | Long-Chain Acyl-CoA Synthetase | miR9730 |
| mQTL-GP16 |  | PE(18:3/18:3), PE(20:0/18:3) | 2, 1, 3, 6, 4, 7, 8 | 20 | Marker1401980, Marker1398370 | -1.66~1.59 | 2.60~4.92 | 2.63~6.94 |  | *Glyma20g29090* | *GmKCS6* | *AT1G68530* | No | gmx00062 | Ketoacyl-CoA Synthase |  |
| mQTL-GP17 |  | PE(18:3/18:2), PE(18:3/18:3) | 5, 7 | 20 | Marker1309177, Marker1309177 | 0.75~1.56 | 3.14~3.75 | 1.64~3.44 |  |  |  |  |  |  |  | miR393k |
| mQTL-GP18 |  | PC(16:0/18:3), PE(16:0/18:3), PE(18:3/18:2), PE(18:3/18:3), PE(20:0/18:3) | 2, 3, 6, 5, 7, 1 | 20 | Marker1374027, Marker1406581 | -2.96~1.16 | 2.58~71.15 | 1.44~7.58 |  | *Glyma20g31030* | *GmCK* | *AT1G74320* | Yes | gmx00564 | Choline Kinase |  |
| mQTL-GP19 |  | PC(16:0/18:3), PE(18:3/18:2), PE(20:0/18:3) | 2 | 20 | Marker1411877, Marker1429663 | 0.83~2.03 | 10.38~17.47 | 2.92~4.16 |  | *Glyma20g31480* | *GmABC* | *AT1G71960* | No | gmx00073 | ABC Transporter | miR1531 |
| mQTL-GP20 |  | CL(18:2/18:1/18:1/18:2), CL(18:2/18:2/18:2/18:1) | 2 | 2 | Marker1162857, Marker1232231 | 0.21~0.22 | 2.59~2.63 | 3.12~3.20 |  | *Glyma02g11180* | *GmGT* | *AT2G04560* | Yes | gmx00564 | glycosyl transferases |  |
| mQTL-GP21 |  | PG(16:0/18:2), CL(18:2/18:1/18:1/18:2), CL(18:2/18:2/18:2/18:1), PG(18:0/16:0) | 2, 4, 8, 3 | 2 | Marker1210105, Marker1210105 | -0.26~0.23 | 2.61~3.60 | 1.41~8.23 |  | *Glyma02g11570* | *GmCer9* | *AT4G34100* | Yes | gmx04141 | E3 ubiquitin Ligase |  |
| mQTL-GP22 |  | PG(18:0/18:2), PG(16:0/18:1) | 2, 1 | 1 | Marker1773932, Marker1938898 | -0.20~0.19 | 2.69~3.10 | 3.13~3.77 |  |  |  |  |  |  |  |  |
| mQTL-GP23 |  | PE(18:3/18:3) | 1, 2 | 1 | Marker1816431, Marker1816431 | -1.47~1.49 | 3.09~3.13 | 5.54~5.89 |  | *Glyma01g05550* | *GmSBH1* | *AT1G14290* | No | gmx00600 | Sphingobase C4-Hydroxylase |  |
| mQTL-GP24 |  | PC(16:0/18:2), PC(18:2/18:2) | 2 | 2 | Marker1287683, Marker1187976 | 0.56~0.58 | 2.94~4.50 | 6.96~8.85 |  |  |  |  |  |  |  |  |
| mQTL-GP25 |  | PE(20:0/18:3), PE(16:0/18:2) | 6, 7 | 2 | Marker1170483, Marker1244390 | -0.16~0.00 | 3.81~7.57 | 0.59~44.63 |  | *Glyma02g46550* | *GmWS* | *AT5G55340* |  | gmx00073 | Wax Synthase |  |
| mQTL-GP26 |  | PC(18:2/18:2) | 2, 6 | 5 | Marker2161030, Marker2164122 | -1.04~6.73 | 4.39~204.51 | 0.89~12.33 |  | *Glyma05g08060* | *GmFatB2* | *AT1G08510* | Yes | gmx00061 | Acyl-ACP Thioesterase B | miR1509b, miR162b |
| mQTL-GP27 |  | PC(16:0/18:2), PC(18:2/18:2), LPE(18:1), PE(18:1/18:1) | 1, 6, 7, 2, 4, 5, 8, 3 | 5 | Marker2205232, Marker2200075 | -0.45~1.38 | 2.54~6.32 | 2.18~21.62 |  | *Glyma05g08880* | *GmOBO* | *AT3G01570* | No |  | Oil-Body Oleosin | miR5374 |
| mQTL-GP28 |  | PE(20:0/18:2) | 1, 3 | 5 | Marker2206570, Marker2206570 | -0.20~-0.12 | 3.80~4.61 | 2.44~8.91 |  |  |  |  |  |  |  |  |
| mQTL-GP29 |  | CL(18:2/18:2/18:2/18:2), PE(18:3/18:3), CL(18:3/18:2/18:2/18:2) | 2, 1 | 6 | Marker1990237, Marker1960320 | -1.53~1.57 | 2.57~3.63 | 2.48~7.51 |  | *Glyma06g02250* | *GmECH2* | *AT1G76150* | Yes | gmx00561 | Peroxisomal Enoyl-CoA Hydratase 2 |  |
| mQTL-GP30 |  | PE(20:0/18:2), PI(18:0/18:1) | 3, 1, 2 | 6 | Marker2078169, Marker2037589 | -0.25~0.27 | 2.54~3.23 | 1.52~7.21 |  | *Glyma06g13630* | *GmACBP1* | *AT4G27780* | Yes | gmx00564 | Acyl CoA Binding Protein | miR171s, miR171h |
| mQTL-GP31 |  | PE(18:2/18:2), PE(16:0/18:2) | 1, 2 | 6 | Marker2037589, Marker2002042 | -0.45~0.43 | 2.62~2.83 | 3.60~6.11 |  | *Glyma06g14530* | *GmLPLA* | *AT3G10840* | Yes | gmx00564 | Lysophospholipase | miR4341, miR6299 |
| mQTL-GP32 |  | PI(16:0/18:2), LPC(18:2), PI(18:1/18:1) | 1, 3, 6 | 7 | Marker303978, Marker335716 | -0.40~0.00 | 2.79~3.91 | 0.00~4.47 |  |  |  |  |  |  |  | miR156l, miR159e |
| mQTL-GP33 |  | PC(16:0/18:1) | 7, 2 | 7 | Marker318686, Marker324565 | 0.00~1.38 | 6.86~40.36 | 0.68~0.75 |  |  |  |  |  |  |  | miR4369 |
| mQTL-GP34 |  | PI(16:0/18:2), LPC(18:2) | 1, 2 | 8 | Marker747366, Marker721168 | -0.57~0.42 | 2.50~2.83 | 6.59~7.50 |  |  |  |  |  |  |  |  |
| mQTL-GP35 |  | PI(16:0/18:2) | 6, 2 | 8 | Marker721168, Marker780349 | 0.00~0.59 | 2.75~3.84 | 0.00~7.20 |  | *Glyma08g11840* | *GmLTP* | *AT1G55260* | No | gmx00073 | Lipid Transfer Protein |  |
| mQTL-GP36 |  | CL(18:2/18:1/18:1/18:2), CL(18:2/18:2/18:2/18:1) | 1, 2 | 8 | Marker791165, Marker718573 | -0.29~0.31 | 2.64~2.66 | 4.76~5.67 |  |  |  |  |  |  |  |  |
| mQTL-GP37 |  | PC(16:0/18:1), PC(18:1/18:2), PE(18:0/18:1), CL(18:2/18:1/18:1/18:2) | 1, 2, 6 | 8 | Marker801358, Marker711697 | -0.42~0.42 | 2.60~4.76 | 0.00~6.78 |  | *Glyma08g19360* | *GmSD* | *AT1G43710* | No | gmx00340 | serine decarboxylase | miR398c |
| mQTL-GP38 |  | LPE(18:2) | 2, 1 | 8 | Marker697758, Marker724472 | -0.52~2.58 | 3.38~54.02 | 6.24~9.38 |  |  |  |  |  |  |  |  |
| mQTL-GP39 |  | CL(18:3/18:2/18:2/18:2), CL(18:2/18:1/18:1/18:2), CL(18:2/18:2/18:2/18:2), LPE(18:1) | 3, 5, 4, 1 | 9 | Marker522118, Marker445705 | -0.43~-0.18 | 2.73~3.84 | 1.32~4.05 |  | *Glyma09g00960* | *GmMCD* | *AT4G04320* | No | gmx00410 | Malonyl-CoA Decarboxylase |  |
| mQTL-GP40 |  | LPC(18:2) | 6, 7, 3, 2 | 10 | Marker1077464, Marker1077464 | -0.27~0.19 | 2.80~4.57 | 1.34~3.46 |  | *Glyma10g01770* | *GmLAH* | *AT3G62590* | No |  | Lipid Acylhydrolase-like | miR10186a, miR164b |
| mQTL-GP41 |  | LPC(18:0), PI(16:0/18:2), PC(16:0/18:1), PE(16:0/18:1), LPC(18:2) | 7, 1, 6, 2, 3 | 10 | Marker996488, Marker1120872 | -0.45~0.45 | 3.00~3.65 | 0.00~5.46 |  | *Glyma10g04060* | *GmLAH* | *AT2G42450* | No |  | Lipid Acylhydrolase-like | miR167d, miR166g |
| mQTL-GP42 |  | PC(16:0/18:1), PI(16:0/18:2) | 6 | 10 | Marker1105060, Marker1140953 | 0.00~0.00 | 3.07~3.75 | 0.00~0.00 |  | *Glyma10g05320* | *GmPAH1* | *AT3G09560* | Yes | gmx00561 | Phosphatidate Phosphatase | miR10426 |
| mQTL-GP43 |  | LPE(18:2) | 1, 7 | 10 | Marker1122410, Marker1021153 | -0.41~-0.19 | 2.82~7.56 | 5.26~13.56 |  | *Glyma10g39540* | *GmLACS6* | *AT3G05970* | No | gmx00061 | Long-Chain Acyl-CoA Synthetase |  |
| mQTL-GP44 |  | CL(18:2/18:1/18:1/18:2), PI(16:0/18:3) | 1, 3 | 12 | Marker2733475, Marker2689018 | -0.26~0.00 | 3.09~3.97 | 0.00~4.11 |  |  |  |  |  |  |  | miR5762 |
| mQTL-GP45 |  | LPC(18:1) | 7, 3, 2 | 12 | Marker2681437, Marker2681437 | -0.12~0.29 | 3.96~5.32 | 1.35~6.73 |  | *Glyma12g02300* | *GmABCG15* | *AT3G21090* | No | gmx00073 | ABC Transporter |  |
| mQTL-GP46 |  | CL(18:2/18:1/18:1/18:2), PE(18:0/18:1), PI(18:2/18:2) | 1, 2, 6 | 12 | Marker2702319, Marker2625558 | -0.31~0.29 | 2.94~6.37 | 0.00~6.32 |  | *Glyma12g04980* | *GmLPAAT5* | *AT3G18850* | Yes | gmx00564 | lysophosphatidyl acyltransferase 5 | miR828b |
| mQTL-GP47 |  | LPC(18:1), PG(16:0/16:0) | 3, 1 | 12 | Marker2675339, Marker2710775 | -0.25~0.13 | 2.86~3.18 | 2.04~5.33 |  |  |  |  |  |  |  | miR10424b |
| mQTL-GP48 |  | LPC(16:0), LPC(18:0) | 6 | 12 | Marker2668097, Marker2668097 | 0.00~0.05 | 3.55~4.57 | 0.00~0.46 |  | *Glyma12g11150* | *GmDREBL* | *AT2G40340* | No |  | AP2 domain | miR4384 |
| mQTL-GP49 |  | PI(16:0/18:3), PI(18:2/18:2) | 6 | 13 | Marker2799118, Marker2799118 | 0.00~0.00 | 3.12~5.84 | 0.00~0.00 |  |  |  |  |  |  |  |  |
| mQTL-GP50 |  | PI(16:0/18:3), PI(18:2/18:2) | 6 | 13 | Marker2819325, Marker2819325 | 0.00~0.01 | 3.67~4.85 | 0.00~0.00 |  |  |  |  |  |  |  |  |
| mQTL-GP51 |  | PC(16:0/18:1), PC(18:1/18:1) | 2 | 13 | Marker2834427, Marker2789430 | -0.39~-0.31 | 2.51~2.64 | 5.31~5.49 |  |  |  |  |  |  |  |  |
| mQTL-GP52 |  | CL(18:2/18:1/18:1/18:2), CL(18:2/18:2/18:2/18:1), CL(18:2/18:2/18:2/18:2), CL(18:3/18:2/18:2/18:2) | 2 | 1 | Marker1865193, Marker1827971 | 0.28~0.39 | 2.54~3.23 | 5.39~6.58 |  |  |  |  |  |  |  |  |
| mQTL-GP53 |  | PG(16:0/18:1), PG(18:0/18:2) | 7 | 13 | Marker2794360, Marker2794360 | 0.00~0.02 | 3.10~3.16 | 1.50~1.56 |  |  |  |  |  |  |  |  |
| mQTL-GP54 |  | PE(16:0/18:1), PE(16:0/18:2), PE(16:0/18:3), PE(18:3/18:2) | 1, 2, 4, 8 | 13 | Marker2818991, Marker2827481 | -1.08~1.02 | 2.53~3.34 | 0.22~10.26 |  | *Glyma13g40420* | *GmDof11* | *AT5G60200* | No |  | Dof domain | miR10192, miR391 |
| mQTL-GP55 |  | PE(18:3/18:2) | 7, 5 | 13 | Marker2779484, Marker2779484 | -1.46~-0.74 | 3.05~3.07 | 1.53~2.65 |  | *Glyma13g43990* | *GmLPLA* | *AT1G52700* | Yes | gmx00564 | Lysophospholipase |  |
| mQTL-GP56 |  | CL(18:2/18:1/18:1/18:2), CL(18:2/18:2/18:2/18:1) | 2, 8, 1 | 14 | Marker1609710, Marker1728394 | -0.29~0.32 | 2.88~3.32 | 2.01~6.52 |  |  |  |  |  |  |  | miR393e, miR169l, miR172d |
| mQTL-GP57 |  | LPC(18:2) | 2 | 14 | Marker1610278, Marker1630592 | 0.26~0.36 | 2.69~3.21 | 3.25~5.58 |  | *Glyma14g33860* | *GmGPAT2* | *AT1G02390* | No | gmx00561 | Glycerol-3-Phosphate Acyltransferase |  |
| mQTL-GP58 |  | PI(16:0/18:3) | 7 | 15 | Marker116270, Marker117451 | 0.00~0.00 | 3.75~4.96 | 24.54~33.65 |  |  |  |  |  |  |  |  |
| mQTL-GP59 |  | PG(16:0/16:0), LPE(18:2) | 2 | 15 | Marker57754, Marker57754 | 0.24~0.45 | 2.71~3.19 | 7.16~8.29 |  |  |  |  |  |  |  |  |
| mQTL-GP60 |  | PI(18:1/18:1) | 6 | 16 | Marker2575348, Marker2575348 | 0.00~0.00 | 3.01~3.22 | 0.00~0.00 |  | *Glyma16g00790* | *GmPDAT1* | *AT5G13640* | Yes | gmx00561 | Phospholipid : Diacylglycerol Acyltransferase |  |
| mQTL-GP61 |  | PE(18:1/18:2), PG(16:0/18:2), CL(18:2/18:2/18:2/18:1), CL(18:2/18:2/18:2/18:2), LPE(18:2) | 7, 4, 5, 3, 1 | 16 | Marker2535866, Marker2542716 | -0.34~0.00 | 3.30~3.71 | 0.00~4.22 |  |  |  |  |  |  |  | miR5668 |
| mQTL-GP62 |  | LPE(18:2), PE(16:0/18:2), CL(18:3/18:2/18:2/18:2) | 1, 2, 5 | 16 | Marker2614429, Marker2603205 | -0.66~0.40 | 2.50~3.37 | 2.09~5.58 |  |  |  |  |  |  |  | miR9729 |
| mQTL-GP63 |  | LPC(18:2), LPC(18:1) | 6, 2 | 16 | Marker2614797, Marker2595561 | -0.23~0.00 | 3.32~3.58 | 0.00~4.13 |  | *Glyma16g27430* | *GmHAD* | *AT5G60340* | No | gmx00061 | Hydroxyacyl-ACP Dehydrase | miR1510a |
| mQTL-GP64 |  | PC(16:0/18:3), PE(18:3/18:3), PE(20:0/18:3) | 6, 1, 2 | 16 | Marker2606142, Marker2602198 | -1.49~1.48 | 2.88~3.19 | 0.00~5.91 |  | *Glyma16g28422* | *GmGLB1* | *AT4G01900* | No | gmx00061 | PII protein | miR1508a |

Note: The methods GCIM, ICIM, ISIS EM-BLASSO, mrMLM, FASTmrEMMA, pLARmEB, pKWmEB, and FASTmrMLM were indicated by 1 ~ 8, respectively.

**Table S16** Co-located QTLs and their candidate genes for oil-related traits and metabolites/lipids

| **Chr** | **Left position** | **Right position** | **Co-located QTL** | **Oil-related traits, metabolites, and lipids** | **Validated by cliques** | **Candidate gene** | **Gene name** |
| --- | --- | --- | --- | --- | --- | --- | --- |
| 14 | 3553972 | 4106610 | q14-7, q14-6, mQTL-G105, mQTL-F30 | Linoleic acid (T), Oleic acid (T), TG(16:0/16:0/18:3), TG(16:0/18:3/18:3), TG(18:0/16:0/18:1), Capric Acid | Oleic acid (T) and TG(18:0/16:0/18:1); Linoleic acid (T) and TG(18:0/16:0/18:1) | *Glyma14g06290* | *GmABI4* |
| 16 | 29720669 | 30277545 | q16-4, mQTL-GP62, mQTL-G121, mQTL-F38 | Palmitic acids (T), LPE(18:2), PE(16:0/18:2), CL(18:3/18:2/18:2/18:2), SQDG(16:0/18:2), SQDG(16:0/18:3), palmitoleic acid, oleic acid |  | *Glyma16g26210* | *GmSPT* |
| 2 | 13572850 | 14213831 | q2-8, q2-7, mQTL-G39, mQTL-F7 | Palmitic acids (T), Palmitic acids (T), TG(16:0/18:2/18:3), TG(16:0/18:3/18:3), OAHFA(18:1/18:0), Arachidic acid |  | *Glyma02g15600* | *GmFAB2* |
| 13 | 22691977 | 23737342 | q13-8, q13-7, q13-6, mQTL-G102, mQTL-C10 | Linolenic acid (T), Linoleic acid (T), Oleic acid (T), TG(18:0/16:0/18:1), TG(18:0/18:0/18:1), TG(18:0/18:1/18:1), D-Fructose 2,6-bisphosphate, mannose | Oleic acid (T) and TG(18:0/16:0/18:1); Linoleic acid (T) and TG(18:0/16:0/18:1) | *Glyma13g19730* | *GmPAH1* |
| 18 | 19157 | 3060050 | q18-1, mQTL-GP6, mQTL-GP5, mQTL-G11 | Linolenic acid (T), LPE(16:0), LPE(16:0), CL(18:2/18:1/18:1/18:2), CL(18:2/18:2/18:2/18:1), CL(18:2/18:2/18:2/18:2), CL(18:3/18:2/18:2/18:2), SQDG(16:0/18:1), DGDG(16:0/18:3), DGDG(18:3/18:3), TG(18:1/18:2/18:3) | Linolenic acid (T) and LPE(16:0); CL(18:2/18:1/18:1/18:2) and CL(18:3/18:2/18:2/18:2); DGDG(16:0/18:3) and DGDG(18:3/18:3) | *Glyma18g01280* | *GmKAR* |
| 20 | 34846011 | 35406778 | q20-3, q20-2, mQTL-GP14, mQTL-G30 | Linoleic acid (T), Oleic acid (T), PE(20:0/18:3), PE(16:0/18:2), PG(18:0/16:0), TG(18:0/18:0/18:1), DG(16:0/18:2), DG(20:1/18:3), TG(18:3/18:2/18:2) |  | *Glyma20g25833* | *GmFatB3* |
| 10 | 1362116 | 2030052 | q10-1, mQTL-GP40, mQTL-O15, mQTL-G71 | Linolenic acid (T), LPC(18:2), Pyruvate, D-Glyceric acid, TG(16:0/16:0/18:2), TG(18:1/18:2/18:3), DG(16:0/18:2), TG(18:0/18:0/18:1) |  | *Glyma10g01420* | *GmGPAT2* |
| 10 | 44155092 | 46094981 | q10-9, q10-8, q10-7, mQTL-G74 | Palmitic acids (T), Linolenic acid (T), Oleic acid (T), TG(16:0/18:1/18:1), TG(16:0/18:1/18:2), TG(18:1/18:1/18:2) | TG(18:1/18:1/18:2) and TG(16:0/18:1/18:2); Oleic acid (T) and TG(16:0/18:1/18:1); Linolenic acid (T) and TG(16:0/18:1/18:2); Linolenic acid (T) and TG(18:1/18:1/18:2) | *Glyma10g38660* | *GmKCS6* |
| 12 | 3316217 | 5004714 | q12-3, q12-2, mQTL-GP46, mQTL-G87 | Linoleic acid (T), Oleic acid (T), CL(18:2/18:1/18:1/18:2), PE(18:0/18:1), PI(18:2/18:2), TG(16:0/18:3/18:3), TG(18:0/16:0/18:1), TG(18:0/18:0/18:1), TG(20:0/18:1/18:2), TG(20:0/18:1/18:1), DGDG(16:0/18:2) | Linoleic acid (T) and CL(18:2/18:1/18:1/18:2); Oleic acid (T) and CL(18:2/18:1/18:1/18:2); Oleic acid (T) and TG(18:0/16:0/18:1); Linoleic acid (T) and DGDG(16:0/18:2); Oleic acid (T) and DGDG(16:0/18:2); Linoleic acid (T) and TG(18:0/16:0/18:1); DGDG(16:0/18:2) and CL(18:2/18:1/18:1/18:2) | *Glyma12g04980* | *GmLPAAT5* |
| 5 | 8916061 | 9115095 | q5-8, q5-7, q5-6, mQTL-GP27 | Linolenic acid (T), Oleic acid (T), Linoleic acid (T), PC(16:0/18:2), PC(18:2/18:2), LPE(18:1), PE(18:1/18:1) |  | *Glyma05g08880* | *GmOBO* |
| 12 | 4661711 | 5681579 | q12-5, q12-4, mQTL-P12, mQTL-GP47, mQTL-G90, mQTL-G88, mQTL-F29 | Linoleic acid (T), Stearic acid (T), serine, LPC(18:1), PG(16:0/16:0), TG(16:0/18:2/18:3), TG(18:3/18:2/18:3), TG(20:0/18:1/18:2), TG(18:1/18:1/18:1), TG(16:0/18:3/18:3), TG(20:1/18:3/18:3), TG(16:0/16:0/18:1), TG(16:0/16:0/18:3), TG(18:0/16:0/18:1), TG(18:0/18:0/18:1), TG(16:0/18:1/18:1), TG(18:0/18:1/18:1), TG(20:0/18:1/18:2), TG(16:0/18:2/18:3), TG(18:3/18:2/18:3), TG(18:1/18:2/18:2), TG(18:1/18:1/18:1), FA(20:1), FA(20:2), FA(22:1) | Linoleic acid (T) and LPC(18:1); Linoleic acid (T) and PG(16:0/16:0); Linoleic acid (T) and TG(18:0/16:0/18:1); Linoleic acid (T) and TG(16:0/18:1/18:1); TG(18:1/18:1/18:1) and TG(16:0/18:1/18:1); TG(18:1/18:1/18:1) and TG(18:3/18:2/18:3); TG(18:0/16:0/18:1) and LPC(18:1); TG(16:0/18:2/18:3) and LPC(18:1); TG(18:1/18:1/18:1) and TG(18:0/16:0/18:1) | *Glyma09g02160* | *GmGH* |
| 5 | 38141310 | 39112357 | q5-13, q5-12, q5-11, q5-10, mQTL-O7 | Stearic acid (T), Linolenic acid (T), Oil content (T), Linoleic acid (T), citric acid |  | *Glyma05g33790* | *GmPEAMT* |
| 8 | 43619069 | 44759525 | q8-6, q8-5, mQTL-C5 | Oil content (T), Linolenic acid (T), D-Glucose | Oil content (T) and D-Glucose | *Glyma08g45990* | *GmENR1* |
| 14 | 4722312 | 5504596 | q14-8, mQTL-GP56, mQTL-F31 | Linolenic acid (T), CL(18:2/18:1/18:1/18:2), CL(18:2/18:2/18:2/18:1), FA(18:0), FA(20:0) |  | *Glyma14g06290* | *GmABI* |
| 16 | 31368945 | 31486713 | q16-5, mQTL-GP63, mQTL-F39 | Palmitic acids (T), LPC(18:2), LPC(18:1), oleic acid |  | *Glyma16g27430* | *GmHAD* |
| 20 | 1681828 | 2014997 | q20-1, mQTL-G26, mQTL-F42 | Oil content (T), DG(20:0/18:2), FA(22:1) |  | *Glyma20g02570* | *GmLCAT* |
| 13 | 20296075 | 20678080 | q13-4, mQTL-O20, mQTL-G100 | Stearic acid (T), succinic acid, citric acid, TG(16:0/18:2/18:3), TG(20:1/18:3/18:3), TG(18:3/18:3/18:3), TG(20:2/18:2/18:2) |  | *Glyma13g16440* | *GmMDH1* |
| 15 | 1926338 | 2082760 | q15-1, mQTL-S1, mQTL-G109 | Oleic acid (T), Cer(d18:2/16:0), CerG1(d18:2/16:0), DG(16:0/16:0), DG(18:0/16:0), DG(18:0/18:0), DG(18:3/18:3) | DG(16:0/16:0) and DG(18:0/18:0) | *Glyma15g02710* | *GmPLDε1* |
| 15 | 49707028 | 50152850 | q15-8, mQTL-GP59, mQTL-G116 | Oil content (T), PG(16:0/16:0), LPE(18:2), SQDG(16:0/18:3), SQDG(16:0/18:2) |  |  |  |
| 2 | 7909600 | 9020096 | q2-3, mQTL-P2, mQTL-G36 | Palmitic acids (T), L-homoserine, beta-Alanine, TG(20:1/18:3/18:3), TG(18:0/18:1/18:1), DG(18:1/18:1) |  | *Glyma02g10060* | *GmPheRS* |
| 3 | 45418367 | 45676554 | q3-8, mQTL-P3, mQTL-G41 | Palmitic acids (T), threonine, TG(16:0/18:1/18:3) |  | *Glyma03g40250* | *GmACP3* |
| 9 | 45780057 | 45959268 | q9-16, q9-15, mQTL-G69 | Linoleic acid (T), Oleic acid (T), TG(18:1/18:2/18:3), DG(18:1/18:2) |  | *Glyma09g40640* | *GmPECT1* |
| 12 | 6240016 | 6755264 | q12-6, mQTL-P12, mQTL-G91 | Linoleic acid (T), serine, TG(16:0/18:2/18:3), TG(18:1/18:1/18:1) |  | *Glyma12g08720* | *GmAAPT1* |
| 10 | 40960112 | 42852684 | q10-5, q10-4, mQTL-O16 | Linoleic acid (T), Oleic acid (T), Threonate |  | *Glyma10g32660* | *GmCDS* |
| 5 | 7566546 | 7798348 | q5-5, q5-4, mQTL-GP26 | Linoleic acid (T), Linolenic acid (T), PC(18:2/18:2) |  | *Glyma05g08060* | *GmFatB2* |
| 13 | 42342607 | 43382478 | q13-18, q13-17, mQTL-GP55 | Linoleic acid (T), Oleic acid (T), PE(18:3/18:2) |  | *Glyma13g44170* | *GmPLDα1* |
| 13 | 25826888 | 26314601 | q13-10, q13-9, mQTL-P13 | Linoleic acid (T), Oleic acid (T), proline |  |  |  |
| 8 | 44272855 | 44691378 | q8-7, mQTL-C6 | Oleic acid (T), D-Glucose | Oleic acid (T) and D-Glucose | *Glyma08g45210* | *GmPHS* |
| 9 | 2595256 | 3393687 | q9-2, mQTL-F24 | Linolenic acid (T), palmitic acid |  |  |  |
| 9 | 3393432 | 3742851 | q9-3, mQTL-F25 | Oil content (T), palmitic acid |  | *Glyma09g04901* | *GmKCS* |
| 15 | 14006679 | 14286484 | q15-4, mQTL-F34 | Linolenic acid (T), FA(18:1), FA(20:0), FA(20:1), OAHFA(18:1/18:0), FA(16:0) |  |  |  |
| 18 | 59964661 | 60102913 | q18-2, mQTL-G22 | Linolenic acid (T), TG(18:1/18:2/18:3), TG(18:1/18:2/18:2) |  | *Glyma18g50580* | *GmKASI* |
| 20 | 39859038 | 40131751 | q20-4, mQTL-G33 | Oil content (T), DG(16:0/16:0), DG(18:0/18:0) | DG(16:0/16:0) and DG(18:0/18:0) | *Glyma20g31630* | *GmDHLAT* |
| 6 | 2234633 | 2615729 | q6-1, mQTL-G52 | Palmitic acids (T), TG(18:0/16:0/18:1), TG(18:2/18:2/18:2), TG(18:0/18:0/18:1) |  | *Glyma06g04230* | *GmPAH2* |
| 10 | 42523481 | 42892855 | q10-6, mQTL-G73 | Oil content (T), TG(20:2/18:2/18:2), DGDG(18:3/18:3) | Oil content (T) and DGDG(18:3/18:3) | *Glyma10g34150* | *GmMDH2* |
| 11 | 36022830 | 36594192 | q11-5, mQTL-G82 | Linolenic acid (T), TG(18:0/18:1/18:1) |  |  |  |
| 12 | 2149546 | 2596172 | q12-1, mQTL-G86 | Palmitic acids (T), TG(18:2/18:2/18:2) |  | *Glyma12g04150* | *GmFBPase* |
| 2 | 10942328 | 10995741 | q2-4, mQTL-O1 | Palmitic acids (T), 2-Oxoadipate |  | *Glyma02g12660* | *GmCHIA* |
| 14 | 6467666 | 6654071 | q14-10, mQTL-O24 | Linoleic acid (T), D-Glyceric acid |  | *Glyma14g08400* | *GmLPAAT4* |
| 2 | 14316826 | 15105561 | q2-9, mQTL-O3 | Oil content (T), Pyruvate |  | *Glyma02g16010* | *GmGSR* |
| 20 | 40131751 | 40547069 | q20-5, mQTL-GP19 | Linolenic acid (T), PC(16:0/18:3), PE(18:3/18:2), PE(20:0/18:3) |  | *Glyma20g31030* | *GmCK* |
| 6 | 11666413 | 12200628 | q6-9, mQTL-GP31 | Linolenic acid (T), PE(18:2/18:2), PE(16:0/18:2) |  | *Glyma06g14530* | *GmLPLA* |
| 7 | 8915752 | 9329473 | q7-12, mQTL-GP32 | Stearic acid (T), PI(16:0/18:2), LPC(18:2), PI(18:1/18:1) |  |  |  |
| 16 | 34849 | 88098 | q16-1, mQTL-GP60 | Linoleic acid (T), PI(18:1/18:1) |  | *Glyma16g00790* | *GmPDAT1* |
| 1 | 54812210 | 55262853 | q1-4, mQTL-GP9 | Linolenic acid (T), PI(16:0/18:3), PI(18:1/18:1) |  | *Glyma01g43470* | *GmLACS4* |
| 6 | 9162441 | 10926010 | q6-8, mQTL-P6 | Linolenic acid (T), proline |  | *Glyma06g11860* | *GmLACS9* |
| 9 | 1424169 | 2595496 | q9-1, mQTL-P9 | Linolenic acid (T), proline, oxoproline |  | *Glyma06g13210* | *GmSMS* |
| 9 | 2864240 | 3393687 | QE12, mQTL-F25 | Linolenic acid (T), palmitic acid |  | *Glyma09g04620* | *GmPLDζ3* |
| 5 | 38248264 | 39112357 | QE2, QE19, mQTL-O7 | Linoleic acid (T), Oleic acid (T), citric acid |  | *Glyma05g33790* | *GmPEAMT* |
| 5 | 8916061 | 9115095 | QE1, mQTL-GP27 | Linoleic acid (T), PC(16:0/18:2), PC(18:2/18:2), LPE(18:1), PE(18:1/18:1) |  | *Glyma05g09160* | *GmLTP* |
| 10 | 44154821 | 46094981 | QE27, mQTL-G74 | Palmitic acid (T), TG(16:0/18:1/18:1), TG(16:0/18:1/18:2), TG(18:1/18:1/18:2) | TG(18:1/18:1/18:2)=TG(16:0/18:1/18:2); Palmitic acid (T)=TG(16:0/18:1/18:1) | *Glyma10g35960* | *GmDHLAT* |
| 13 | 25826888 | 26293580 | QE8, QE14, QE21, mQTL-P13 | Oleic acid (T), Linoleic acid (T), Linolenic acid (T), proline |  | *Glyma13g22230* | *GmHACPS* |
| 13 | 20296075 | 20678080 | QE35, mQTL-G100, mQTL-O20 | Stearic acid (T), TG(16:0/18:2/18:3), TG(20:1/18:3/18:3), TG(18:3/18:3/18:3), TG(20:2/18:2/18:2), succinic acid, citric acid |  | *Glyma13g16560* | *GmDGAT1a* |
| 6 | 9162441 | 10195951 | QE16, mQTL-P6 | Linolenic acid (T), proline |  | *Glyma06g11650* | *GmPIPK-IB* |
| 14 | 3379092 | 3810849 | QE15, QE20, mQTL-G105 | Linolenic acid (T), Oleic acid (T), TG(16:0/16:0/18:3), TG(16:0/18:3/18:3), TG(18:0/16:0/18:1) | Oleic acid (T)=TG(18:0/16:0/18:1) | *Glyma14g05510* | *GmTAGL* |
| 14 | 6467405 | 6618161 | QE6, mQTL-O24 | Linoleic acid (T), D-Glyceric acid |  | *Glyma14g08400* | *GmLPAAT4* |
| 14 | 4928302 | 5504596 | QE5, mQTL-F31, mQTL-GP56 | Linoleic acid (T), FA(18:0), FA(20:0), CL(18:2/18:1/18:1/18:2), CL(18:2/18:2/18:2/18:1) | Linoleic acid (T)=CL(18:2/18:1/18:1/18:2) | *Glyma14g07290* | *GmGPAT5* |
| 2 | 44478668 | 45564581 | mQTL-C1, QE31, mQTL-GP24 | D-Glucose, mannose, Stearic acid (T), PC(16:0/18:2), PC(18:2/18:2) |  | *Glyma02g40280* | *GmHSI2/VAL1* |
| 2 | 13572614 | 14213831 | QE24, mQTL-F7, mQTL-G39 | Palmitic acid (T), OAHFA(18:1/18:0), Arachidic acid, TG(16:0/18:2/18:3), TG(16:0/18:3/18:3) | Palmitic acid (T)=TG(16:0/18:2/18:3) | *Glyma02g15600* | *GmFAB2* |
| 2 | 12620402 | 13008018 | QE30, mQTL-O2 | Palmitic acid (T), Pyruvate |  | *Glyma02g14211* | *GmAAPT* |
| 7 | 12129535 | 14979626 | QE17, mQTL-F20, mQTL-O9 | Oil content (T), linolenic acid, citric acid, alpha-ketoisocaproic acid |  | *Glyma07g13780* | *GmPAH* |
| 12 | 3316217 | 4493712 | QE4, mQTL-G87, mQTL-GP46 | Linoleic acid (T), TG(16:0/18:3/18:3), TG(18:0/16:0/18:1), TG(18:0/18:0/18:1), TG(20:0/18:1/18:2), TG(20:0/18:1/18:1), DGDG(16:0/18:2), CL(18:2/18:1/18:1/18:2), PE(18:0/18:1), PI(18:2/18:2) | Linoleic acid (T)=CL(18:2/18:1/18:1/18:2); Linoleic acid (T)=DGDG(16:0/18:2); Linoleic acid (T)=TG(18:0/16:0/18:1); DGDG(16:0/18:2)=CL(18:2/18:1/18:1/18:2) | *Glyma12g07230* | *GmDIR10* |
[truncated: 273,912 more chars]
